# Comprehensive genomics in androgen receptor-dependent castration-resistant prostate cancer identifies an adaptation pathway mediated by opioid receptor kappa 1

**DOI:** 10.1038/s42003-022-03227-w

**Published:** 2022-04-01

**Authors:** Yuki Makino, Yuki Kamiyama, J. B. Brown, Toshiya Tanaka, Ryusuke Murakami, Yuki Teramoto, Takayuki Goto, Shusuke Akamatsu, Naoki Terada, Takahiro Inoue, Tatsuhiko Kodama, Osamu Ogawa, Takashi Kobayashi

**Affiliations:** 1 Department of Urology, Kyoto University Graduate School of Medicine, Kyoto, Japan; 2 Laboratory of Molecular Biosciences, Kyoto University Graduate School of Medicine, Kyoto, Japan; 3 Center for Cancer Immunotherapy and Immunobiology, Kyoto University Graduate School of Medicine, Kyoto, Japan; 4 Research Center for Advanced Science and Technology, The University of Tokyo, Tokyo, Japan; 5 Department of Gynecology and Obstetrics, Kyoto University Graduate School of Medicine, Kyoto, Japan; 6 Department of Diagnostic Pathology, Kyoto University Graduate School of Medicine, Kyoto, Japan

**Keywords:** Cancer therapeutic resistance, Prostate

## Abstract

Castration resistance is a lethal form of treatment failure of prostate cancer (PCa) and is associated with ligand-independent activation of the androgen receptor (AR). It is only partially understood how the AR mediates survival and castration-resistant growth of PCa upon androgen deprivation. We investigated integrative genomics using a patient-derived xenograft model recapitulating acquired, AR-dependent castration-resistant PCa (CRPC). Sequencing of chromatin immunoprecipitation using an anti-AR antibody (AR-ChIP seq) revealed distinct profiles of AR binding site (ARBS) in androgen-dependent and castration-resistant xenograft tumors compared with those previously reported based on human PCa cells or tumor tissues. An integrative genetic analysis identified several AR-target genes associated with CRPC progression including OPRK1, which harbors ARBS and was upregulated upon androgen deprivation. Loss of function of OPRK1 retarded the acquisition of castration resistance and inhibited castration-resistant growth of PCa both in vitro and in vivo. Immunohistochemical analysis showed that expression of OPRK1, a G protein-coupled receptor, was upregulated in human prostate cancer tissues after preoperative androgen derivation or CRPC progression. These data suggest that OPRK1 is involved in post-castration survival and cellular adaptation process toward castration-resistant progression of PCa, accelerating the clinical implementation of ORPK1-targeting therapy in the management of this lethal disease.

## Abstract (web summary)

Through comparative genomics using PDX models of androgen-dependent (AD) and androgen-resistant (AR) tumors, Makino et al. identify opioid receptor kappa 1 (OPRK1) as being associated with castration-resistance. Loss of OPRK1 function delays castration-resistance and inhibits castration-resistant growth of prostate cancer cells in culture and in vivo, suggesting OPRK1 as a therapeutic target.

## Introduction

Prostate cancer (PCa) is the most frequently diagnosed cancer in men^1^. Although the majority of PCas are treated with an excellent survival outcome, a subset of patients develop an advanced form of the disease which is ultimately lethal. Since the most of treatment-naïve PCas are dependent on androgen receptor (AR) signaling, the standard treatment for advanced PCa is androgen deprivation and AR-targeting therapy^2,3^.

Clinically, the initial response rate of PCa to androgen deprivation and targeted AR inhibition therapy is very high. Upon androgen deprivation, AR activity is attenuated and PCa cells go into G1 arrest^4^. However, only a subset of cells undergoes apoptosis or other cell death processes whereas the rest of PCa cells undergo adaptive survival processes^5^. Biological mechanisms for anti-castration adaptive survival signaling have not been fully understood, although several previous studies reported implication of AKT^6^ and clusterin^7^ in this process, being characterized by upregulation upon androgen blockade.

PCa cells that survive under a castrated condition eventually acquire the ability of castration-resistant growth. Amongst various mechanisms previously reported for the development of castration-resistant PCa (CRPC), aberrant activation of AR has been considered as the most important driving force toward castration-resistant (CR) progression^8–12^. Using chromatin immunoprecipitation with anti-AR antibody (AR-ChIP), Wang and colleagues showed that AR regulated a distinct transcriptional program between androgen-independent and -dependent human PCa cells^13^. Since then, AR-ChIP using AR-expressing PCa cells has been demonstrated as a useful tool for the understanding of AR-mediated signals in PCa^14^. In another report by Sharma et al.^15^, AR-ChIP using human PCa tissues revealed that there were substantial discrepancies in the profiles of AR-binding sites (ARBS) between cell lines and tumor tissues. This indicates that to better recapitulate the processes leading to castration resistance, AR-ChIP for ARBS profiling should be performed using in vivo tumor tissues. However, it is very difficult to obtain sufficient amount of prostate cancer tissue from surgical specimens. Additionally, it is almost impossible to obtain pairwise fresh samples of androgen-dependent (AD) and CR tumors from the same patient in clinical practice.

Herein, to overcome the above-mentioned barriers in obtaining comparative genomics data (including gene expression and ARBS profiling) between AD and CR tumors, we used a patient-derived xenograft (PDX) of PCa that recapitulated acquired castration resistance in an AR-dependent manner. Whereas fresh AD and CR tumors from the same patient present a major obstacle, PDX models can circumvent this issue. This work provides useful information on comparative genomics data between AD and CR tumors from the same origin. Critically, the analyses herein have identified OPRK1 as a potential novel therapeutic target; notably, OPRK1 expression was upregulated upon castration, and its loss of function retarded acquisition of CR progression by blocking cell proliferation and tumor growth in multiple CRPC models.

## Results

### KUCaP2 as an AR-dependent CRPC model

**Fig. 1:**
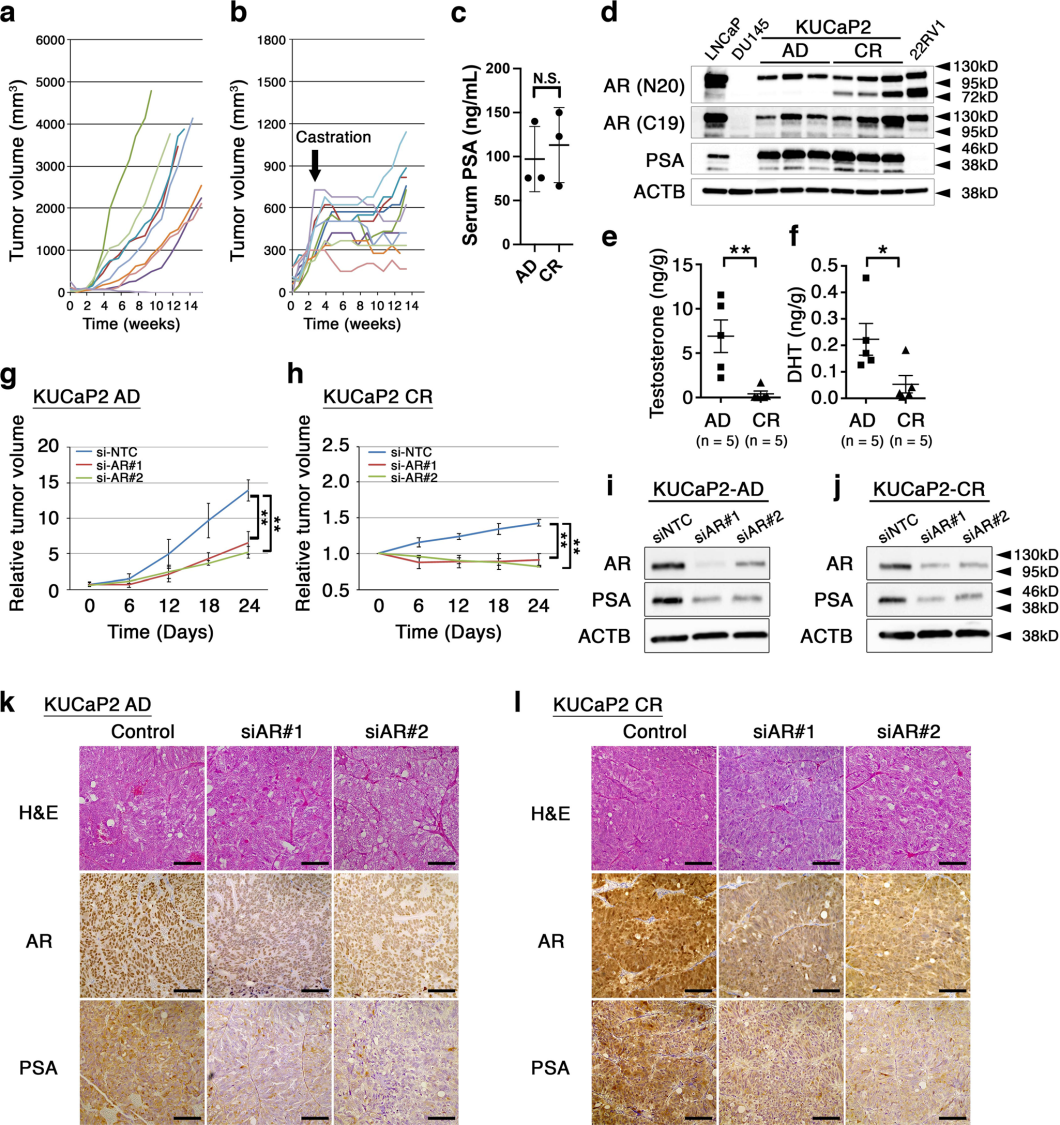
KUCaP2 is a PDX line as a model for androgen receptor-dependent castration-resistant prostate cancer. **a**, **b** Individual growth curves of KUCaP2 tumors in intact (**a**) and castrated (**b**) mice. Arrow indicates surgical castration. **c** Serum prostate-specific antigen (PSA) levels in androgen-dependent (AD) and castration-resistant (CR) tumor-bearing mice (*n* = 4 each). **d** Western blotting of indicated proteins in AD and CR KUCaP2 tumors. Two antibodies for androgen receptor (AR) recognizing distinct epitopes on N-terminus (N20) and C-terminus (C19) were used. LNCaP, PC3, and 22RV1 prostate cancer cell lines act as controls. ACTB; β-actin. **e**, **f** Serum testosterone (**e**) and dihydrotestosterone (DHT, **f**) levels in mice bearing AD and CR KUCaP2 tumors. **g**, **h** Growth curves of AD (**g**) and CR (**h**) KUCaP2 tumors treated with control (siNTC) or siRNAs for AR (siAR#1 and siAR#2). **P < 0.01 (ANOVA). **i**, **j** Western blotting of indicated proteins in AD (**i**) and CR (**j**) KUCaP2 tumors treated with control or siRNAs for AR. **k**, **l** Representative microphotographs of hematoxylin and eosin (H&E) stain and immunohistochemical stains for AR and PSA in AD (**k**) and CR (**l**) KUCaP2 tumors treated with control or siRNAs for AR. Bars indicate 50 μm.

We previously established a patient-derived xenograft line KUCaP2 that harbored wild-type AR and expressed PSA^16^. KUCaP2 tumors grow in intact male mice, stop growing or shrinking upon castration, and start re-growth after ~6 weeks. We confirmed the monotonic growth in non-castrated mice (Fig. 1a) as well as response to castration followed by acquisition of castration-resistant growth (Fig. 1b). In both statuses, mice bearing KUCaP2 tumors showed high serum levels of prostate-specific antigen (PSA, ~100 ng/ml, Fig. 1c).

Western blotting using whole lysate obtained from KUCaP2 tumors revealed that both androgen-dependent (AD) and castration-resistant (CR) tumors expressed signals detected by two distinct antibodies against N-terminus (N20) and C-terminus (C19) of AR at the level of 110 kD, which is equivalent to the molecular size of full-length AR (AR-FL, Fig. 1d). Notably, KUCaP2 CR tumors showed signals exclusively detected by antibody against N-terminus at the level of 75 kD, which is highly suspect of a truncated variant of AR including AR-V7^17,18^. Expression of AR-V7 in KUCaP2 CR tumors was confirmed using specific antibody to AR-V7 following immunoprecipitation using antibody to AR N-terminus (Supplementary Fig. 1a). Additionally, reverse transcriptase PCR (RT-PCR) using specific primers (Supplementary Fig. 1b) showed increased expression of AR-FL (Supplementary Fig. 1c) as well as expression of AR-V1 (Supplementary Fig. 1d) and AR-V7 (Supplementary Fig. 1e) at the mRNA level in KUCaP2 CR tumors. Moreover, we obtained consistent results from RNA sequence (Supplementary Fig. 1f). No single-nucleotide variation was detected in *AR* mRNA. We further investigated the DNA copy number of the *AR* gene using genome-based quantitative PCR. It successfully detected previously known copy number increase in exons 2b and 3 and cryptic exon 3 (CE3) of 22RV1 cells by approximately two fold^17,18^. We observed copy number increases up to 1.5 fold in some of KUCaP2 AD tumors while KUCaP2 CR tumors harbored 2–3-fold increases (Supplementary Fig. 1g), suggesting that some clones harboring *AR* copy number gain existed in KUCaP2 AD tumors, selectively grew in the absence of androgen, and became dominant in the process of castration-resistant progression^11,12^.

Since intratumoral androgen neo-synthesis and subsequent ligand-dependent activation of AR have been considered one of the major molecular mechanisms for CRPC progression^19^, we examined KUCaP2 CR tumors and determined intratumoral testosterone and dihydrotestosterone (DHT) using mass spectrometry. It was revealed that both testosterone (Fig. 1e) and DHT (Fig. 1f) were suppressed to sub-castration levels, indicating that AR in KUCaP2 CR tumors was activated at very low androgen levels.

We next asked whether growth of KUCaP2 AD and CR tumors was dependent on AR. We tried to silence *AR* using siRNA, using two distinct siRNAs for AR delivered with atelocollagen^20^. AR silencing effectively retarded tumor growth in both KUCaP2 AD (Fig. 1g) and CR (Fig. 1h) tumors. We confirmed the decreased abundance of AR and PSA using western blot (Fig. 1i, j) and immunohistochemistry (IHC, Fig. 1k, l). The expressions of other AR-regulated genes were also suppressed by AR knockdown in both KUCaP2 AD (Supplementary Fig. 1h) and CR tumors (Supplementary Fig. 1i).

Taken together, KUCaP2 is a model for AR-dependent CRPC which recapitulates castration-induced growth retardation followed by acquisition of castration-resistant growth ability. KUCaP2 CR tumors exhibited *AR* amplification and AR-V7 expression, which was reportedly observed in ~50% of CRPC^21^.

### Androgen-independent sublines derived from LNCaP cells as AR-expressing CRPC models

In addition to an in vivo KUCaP2 model, we established androgen-independent sublines of LNCaP cells as culture cell CRPC models. The parental LNCaP cells were cultured in charcoal-strip FBS media and four clones that grew up in the absence of androgen for 2–3 months were established as AI (androgen-independent) LNCaP1 to 4. AILNCaPs expressed 2–3-fold higher *AR* mRNA compared with the parental LNCaP (Supplementary Fig. 2a), whereas they scarcely expressed AR-related genes including *KLK2*, *KLK3*, *TMPRSS2*, *NKX3.1*, and *FKBP5* in the androgen-deprived condition (Supplementary Fig. 2b). These were reflected to protein expressions as confirmed by western blotting (Supplementary Fig. 2c). AILNCaP1 to 4 did not express AR truncated variant (Supplementary Fig. 2c). When the AILNCaP cells were treated with siRNA for AR, *AR* mRNA was efficiently suppressed in LNCaP and all AILNCaPs but AILNCaP1 (Supplementary Fig. 2d). AR silencing inhibited the proliferation of AILNCaP2 to 4 by 20–30% (Supplementary Fig. 2e), suggesting that the androgen-independent proliferation of the AILNCaPs was, at least partially, dependent on AR. These findings collectively suggested that AILNaP2 to 4 were also able to act as CRPC models expressing functional AR and we decided to use AILNaP2 to 4 thereafter.

### ChIP seq analysis using AR-expressing CRPC models

It has been reported that the AR is activated and acts as the driver in the majority of CRPC^4,8,11^. However, a previous report demonstrated that activated AR regulated a distinct transcriptional program from that in the presence of androgen^13^. Another previous report showed that there was a discrepancy between regulatory programs in cultured AR-driven prostate cancer cells and in vivo human prostate cancer tissues^22^. To address this issue, we investigated AR-binding sites (ARBS) in KUCaP2 AD and KUCaP2 CR tumors as well as LNCaP and AILNCaP cells using chromatin immunoprecipitation with anti-AR antibody (AR-ChIP).

Since AR ChIP using xenograft tumor tissue has not been reported elsewhere, we first optimized it in the following ways. First KUCaP2 AD and CR tumors (*n* = 3 each) were subject to ChIP using anti-AR (N-terminus) antibody, control normal IgG, and anti-histone H3 antibody and abundance of *KLK3* enhancer region in immunoprecipitated DNA samples were determined with quantitative PCR. It showed that ChIP using anti-AR antibody enriched *KLK3* enhancer region by ~15–80-fold compared with that using control IgG (Supplementary Fig. 3a). Immunoprecipitated DNA with anti-AR antibody was also subject to quantitative PCR using specific primers to *KLK3* enhancer region and *GAPDH*, which showed ~5–10-fold concentration in terms of percent input (Supplementary Fig. 3b). These results collectively indicated that our AR ChIP had been optimized for specific enrichment of ARBS using KUCaP2 xenograft tumor tissue. Moreover, we confirmed that our AR ChIP worked also on LNCaP and AILNCaP cells in the same manner (Supplementary Fig. 3c).

**Fig. 2:**
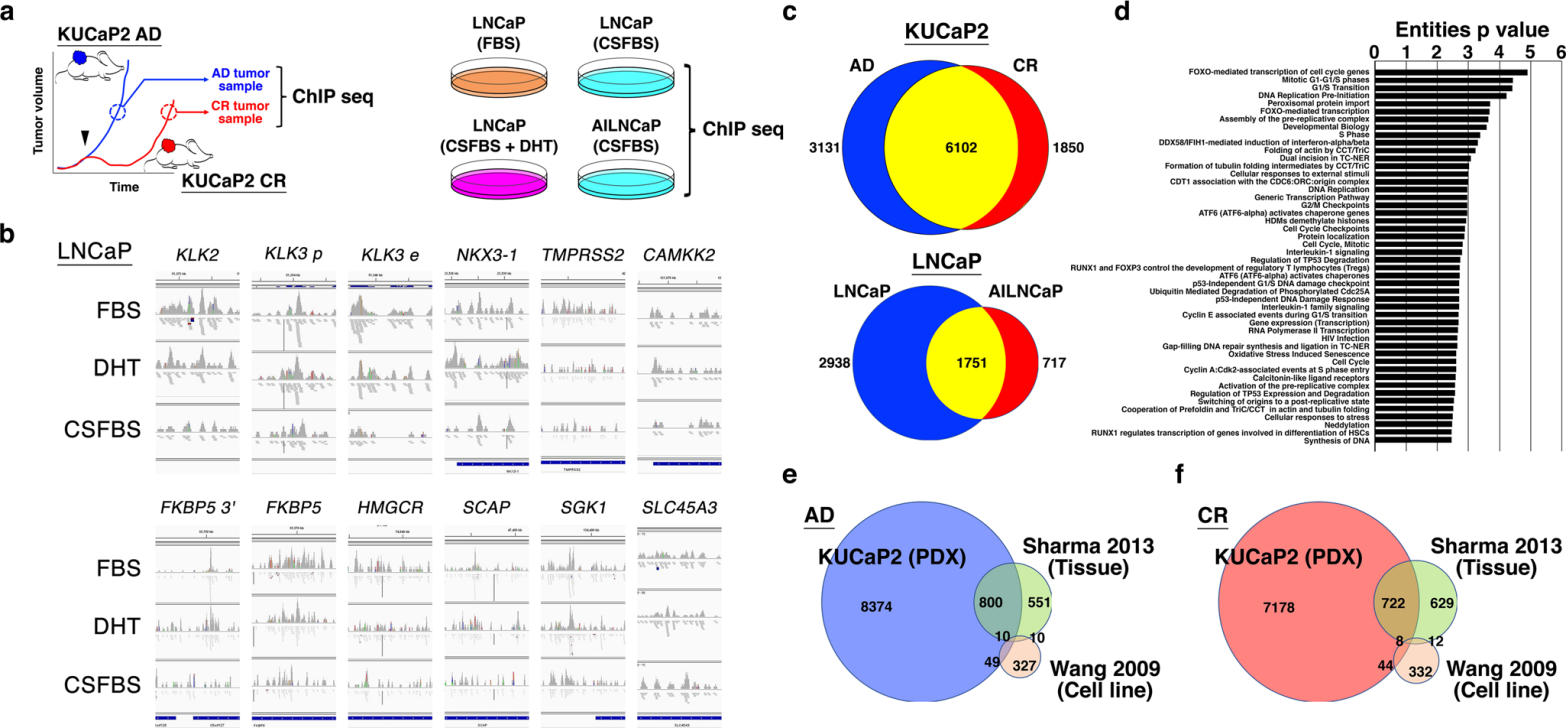
Chromatin-immunoprecipitation sequence (ChIP seq) using antibody against androgen receptor (AR). **a** Top: Experimental schemes. KUCaP tumors under androgen-dependent (AD) and castration-resistant (CR) growth were obtained. Bottom: LNCaP cells cultured in FBS, charcoal-strip FBS (CSFBS) and CSFBS supplemented with 1 nM dihydrotestosterone (CSFBS + DHT) as well as AILNCaP cultured in CSFBS were subject to CHiP seq. **b** Quality controls for ChIP seq binding data. Bar files were generated after MAT analysis of AR whole-genome ChIP seq raw data from LNCaP cells cultured in FBS, charcoal-strip FBS (CSFBS), and CSFBS supplemented with 1 nM dihydrotestosterone (DHT). AR-binding peaks at the promoter regions (unless otherwise indicated) of indicated genes^13^ are shown. *KLK3* p; *KLK3* (*PSA*) promoter, *KLK3* e; *KLK3* (*PSA*) enhancer, *FKBP5 3'*, 3' UTR region of *FKBP5*. **c** Venn diagrams showing AR-binding sites in KUCaP2 AD and CR tumors (top), and LNCaP and AILNCaP cells (bottom). We identified 3131 differential AR-binding sites for KUCaP AD tumors, 1850 for KUCaP2 CR tumors, 2,938 for LNCaP cells, and 717 for AILNCaP cells, which were defined as having fold change ≤ 0.5 or ≥2 compared with each counterpart. There were 6102 AR-binding sites commonly identified in KUCaP2 AD and CR tumors and 1751 in LNCaP and AILNCaP cells. **d** Reactome pathways for genes exclusively identified for KUCaP2 CR tumors annotated by AR-binding sites in AR-ChIP seq with regard to entities *p*-values (−log[*p*-value]). **e**, **f** Venn diagrams depicting differentially and commonly identified genes harboring AR-binding site in AD (**e**) and CR (**f**) models including PDX (the present study), human PCa tissue^22^, and cell lines^13^.

For AR-ChIP seq, we obtained KUCaP2 AD and CR tumors (*n* = 3 each) that were growing in intact and castrated mice, respectively (Fig. 2a). We also obtained LNCaP cells cultured in normal media (10% FBS), androgen-deprived media (10% charcoal-stripped FBS [CSFBS]) and androgen-deprived and DHT-supplemented media (CSFBS + DHT) as well as AILNCaPs (AILNCaP2, 3, and 4) cultured in androgen-deprived media. After sequence data for each AR ChIP sample were obtained, we determined the threshold of *q*-value as <0.129 so that peak calls of *KLK3* enhancer region could be specifically detected in LNCaP FBS and LNCaP CSFBS + DHT but not in LNCaP CSFBS. Under this condition, we confirmed that LNCaP FBS and LNCaP CSFBS + DHT showed stronger signals compared with LNCaP CSFBS in the previously reported ARBSs within *KLK2*, *KLK3* (promoter region), *NKX3.1*, *TMPRSS2*, *CAMKK2*, *FKBP5* (3' end region), *FKBP5*, *HMGCR*, *SCAP*, *SGK1*, and *SLC45A* as well as *KLK3* (enhancer region) (Fig. 2b, Supplementary Fig. 3d), suggesting that our AR-ChIP successfully enriched true ARBS.

Using this threshold, we analyzed ARBS detected in KUCaP2 AD and CR tumors. We defined ARBS of KUCaP2 tumors in each status as being called in at least two of the three samples. Then, each ARBS was annotated to a gene located within a 20-Kb range. A total of 11,083 genes were called; 3131 were exclusively for KUCaP2 AD, 1850 for KUCaP2 CR, and 6102 were shared with both AD and CR tumors (Fig. 2c top, Supplementary Data S1). For LNCaP cells and the androgen-independent sublines (AILNCaP), a total of 5406 genes were called; 2938 were exclusively for LNCaP, 717 for AILNCaP, and 1751 were shared with both LNCaP and AILNCaP cells (Fig. 2c bottom, Supplementary Data S2).

According to Reactome gene ontology analysis, many of genes harboring ARBS exclusively called in KUCaP2 CR tumors were related to cell cycle (Fig. 2d), which is consistent with a previous report showing that CRPC cells with or without treatment-induced AR variants activated a distinct expression signature enriched for cell-cycle genes^13,23^. On the other hand, genes harboring ARBS exclusively called in KUCaP2 AD tumors were related to transcription, translation elongation, and cell cycle (Supplementary Fig. 4a), while those commonly called in KUCaP2 AD and CR tumors were related to intracellular signal transduction including membrane trafficking, receptor tyrosine kinases, Rho GTPase signaling and SUMOylation (Supplementary Fig. 4b).

In terms of LNCaP and AILNCaPs, a total of 5406 genes were called; 2938 were exclusively for LNCaP, 717 for AILNCaPs, and 1751 were shared with both LNCaP and AILNCaP cells (Fig. 2c bottom). We defined ARBS of AILNCaP cells in each status as being called in at least two of the three AILNCaP sublines. These findings collectively indicate that as PCa progresses to CR disease, aberrantly activated AR regulates a distinct, CR-specific transcription program while maintaining, at least in part, a common transcription program with AR driving androgen-dependent PCa.

We further interrogated ARBS detected in KUCaP2 tumors in comparison with those reported by previous studies on AR ChIP (Fig. 2e, f)^13,22^. Among ARBS identified in human PCa tissues, 59.1% (810 of 1371) for AD and 53.2% (730 of 1371) for CR tumors were shared with PDX tumors, whereas only 14.9% (59 of 396) for AD cells and 13.1% (52 of 396) among ARBS identified in human PCa cells were shared with PDX tumors, highlighting the divergence between AR-targeting genes between in vivo tumors and cultured cells^22^.

### A search combining AR-ChIP seq, RNA seq, and public database identified candidates for therapeutic targets including OPRK1

We next investigated potential therapeutic targets in CRPC using KUCaP2, a model that well recapitulated the typical clinical behavior of PCa including androgen-dependent growth, response to castration and castration-resistant progression in vivo. Our AR-ChIP seq strongly indicated that KUCaP2 significantly altered the profile of genes that were transcriptionally regulated by AR in the process of progressing from AD to CR status. However, it did not seem to fully reflect expression of the screened genes in tumors. Therefore, we combined RNA seq analysis using KUCaP2 AD and CR tumors with the results from AR-ChIP seq to narrow candidate genes for a therapeutic target in CRPC. Furthermore, we searched publicly available databases as we considered it important to identify targets that are not specific to KUCaP2 but are more generalized for the treatment of CRPC.

**Fig. 3:**
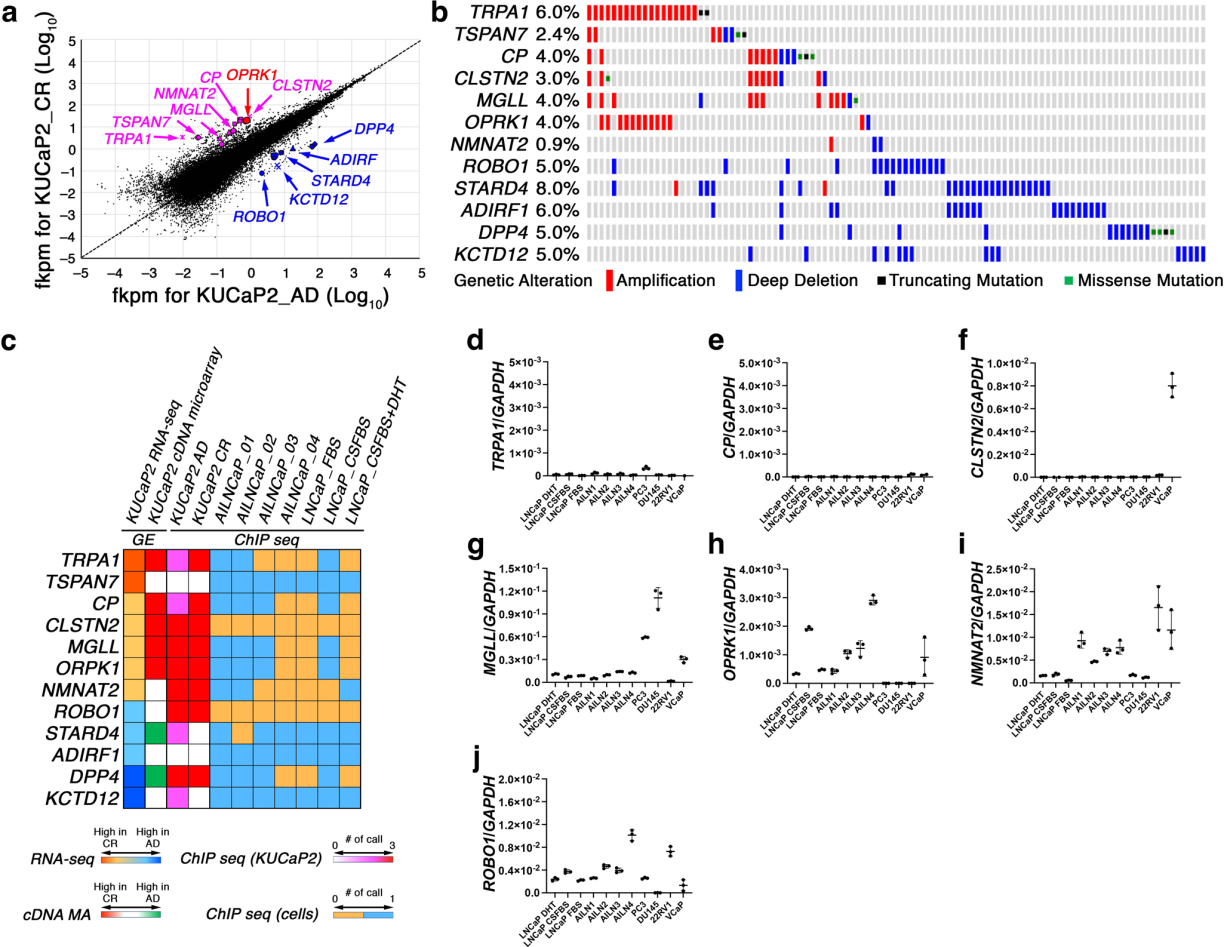
RNA sequence KUCaP2 AD and CR tumors. Among 30 most significantly upregulated genes in RNA seq of KUCaP2 CR compared with AD tumors, seven genes that were reported to be frequently amplified in PCa were picked up. Likewise, among 30 most significantly downregulated genes in KUCaP2 CR tumors, five genes that were reported to be frequently altered (deep deletion, and truncating and missense mutations) in CRPC were picked up. **a** Dot plot of log_10_[fkpm] indicating the 12 genes. **b** Landscape of alterations (amplification, deep deletion, and truncating and missense mutations) of the 12 genes primary PCa^24^. **c** Summarized results from integrated genomics including RNA seq of KUCaP2 tumors (AD vs CR), cDNA microarray of KUCaP2 tumors (AD vs CR)^16^, ChIP seq of KUCaP2 tumors (AD and CR), and ChIP seq of AILNCaPs (four independent sublines) and LNCaP cells cultured in FBS (LNCaP_FBS), charcoal-stripped FBS (LNCaP_CSFBS), and CSFBS supplemented with 1 nM dihydrotestosterone (LNCaP_CSFBS + DHT). **d**–**j** Quantitative RT-PCR in human PCa cell lines. Expressions of *TRPA1* (**d**), *CP* (**e**), *CLSTN2* (**f**), *MGLL* (**g**), *OPRK1* (**h**), *NMNAT2* (**i**), and *ROBO1* (**j**) normalized by that of *GAPDH* in the indicated PCa cell lines.

In RNA seq, 149 genes were identified as differentially upregulated in CR tumors with fkpm > 3.0 and fold change > 4.0 (Supplementary Data S3), while 79 were identified as differentially upregulated in AD tumors with fkpm > 3.0 and fold change > 4.0 (Supplementary Data S4). Additionally, we screened for frequently (5% or more) altered (amplified, deeply deleted, truncated, or missense-mutated) genes in either of the previously published data from large-scale next-generation sequencing (NGS) on human primary PCa^24^, metastatic PCa^25^, or metastatic CRPC^21,26^. Among the top 30 differentially expressed genes in RNA seq, we identified 12 frequently altered genes according to published NGS data, seven of which were upregulated in CR (*TRPA1*, *TSPAN7*, *CP*, *CLSTN2*, *MGLL*, *OPRK1*, *NMNAT2*) while five of which were downregulated in CR (*ROBO1*, *STARD4*, *ADRIF1*, *DPP4*, *KCTD12*) (Fig. 3a, b, Supplementary Fig. 5a–c). Of these 12 genes, *TSPAN7*, *STARD4*, *ADRIF1*, and *KCTD12* were not detected by AR-ChIP on KUCaP2 (Fig. 3c) and *DPP4* failed to reproduce the differential expression in quantitative reverse transcriptase PCR (RT-PCR), while the remaining seven genes (*TRPA1*, *CP*, *CLSTN2*, *MGLL*, *OPRK1*, *NMNAT2*, *ROBO1*) showed reproducible differential expressions (Supplementary Fig. 6). Of note, all seven genes were detected by AR-ChIP seq on both KUCaP2 AD and CR tumors, suggesting the AR-binding on those genes is, at least in part, maintained under a castrated condition.

Then we investigated expression of the seven genes in human PCa cell lines (LNCaP, AILNCaP, PC3, DU145, 22RV1, VCaP) using RT-PCR. Amongst six that were upregulated in KUCaP2 CR tumors, *TRPA1* (Fig. 3d) and *CP* (Fig. 3e) were scarcely expressed in the most of the PCa cell lines, while *CLSTN2* was expressed in almost exclusively in VCaP cells (Fig. 3f). *MGLL* (Fig. 3g), *OPRK1* (Fig. 3h), and *NMNAT2* (Fig. 3i) showed upregulation in androgen-independent cells. *ROBO1* (Fig. 3j), which showed downregulation in KUCaP2 CR tumors, did not show downregulation in androgen-independent cells. Therefore, *CLSTN2*, *MGLL*, *OPRK1*, and *NMNAT2* were selected for further investigation by functional assays as candidate genes for therapeutic targets of CRPC.

### Loss-of-function experiments using AILNCaP found potential for *OPRK1* and *NMNAT2* as therapeutic targets

We next asked whether the AR affected the expression of candidate genes and whether loss-of-function of those genes affected proliferation of PCa cells using siRNA silencing. As for *CLSTN2*, we used VCaP cells since *CLSTN2* was expressed almost exclusively in VCaP (Fig. 3f). Expression of *CLSTN2* was significantly suppressed in VCaP cells treated with siRNAs for AR (Supplementary Fig. 7a). When *CLSTN2* was silenced using two distinct siRNAs (Supplementary Fig. 7b), the cell proliferation was not significantly inhibited (Supplementary Fig. 7c). As for *MGLL*, siRNA knockdown of AR partially decreased *MGLL* expression in LNCaP, AILNCaP3, and AILNCaP4 (Supplementary Fig. 8a). Expression of *MGLL* was significantly suppressed in the three cell lines by two distinct siRNAs for *MGLL* (Supplementary Fig. 8b). However, there was no significant difference in the cell proliferation by *MGLL* knockdown (Supplementary Fig. 8c). On the other hand, AR silencing tended to decrease *NMNAT2* expression in LNCaP, AILNCaPs 2–4 (Supplementary Fig. 9a). When *NMNAT2* expression was significantly suppressed by two distinct siRNAs for *NMNAT2*, the four cell lines showed statistically significant retardation in cell proliferation (Supplementary Fig. 9b).

**Fig. 4:**
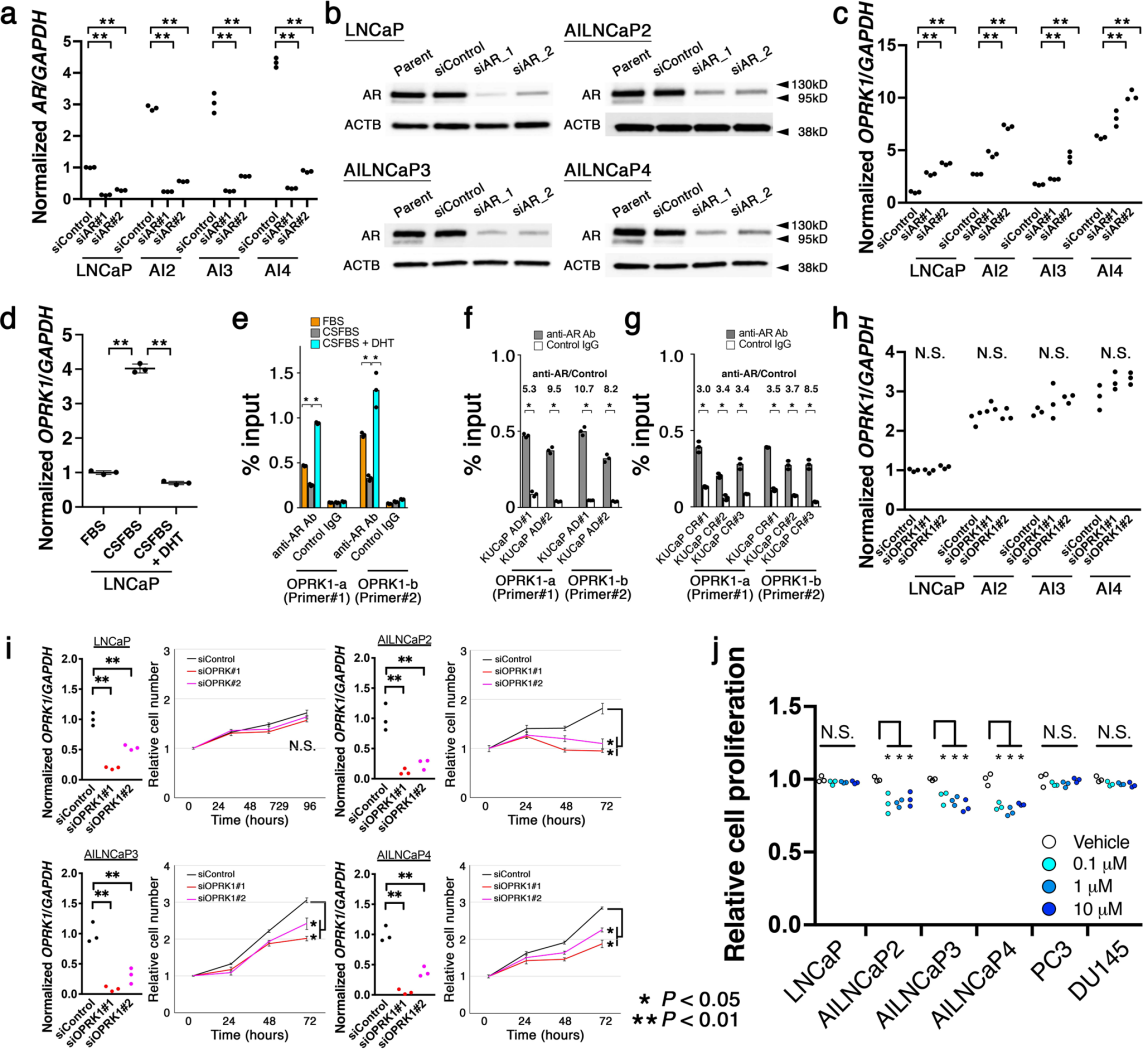
AR suppression upregulates OPRK1, which underpin androgen-independent growth of androgen-independent derivatives of LNCaP cells. **a**, **b** Expressions of *AR* in LNCaP, AILNCaP2 (AI2), AILNCaP3 (AI3), and AILNCaP4 (AI4) treated with siRNA for *GFP* (siControl) and *AR* (siAR#1 and siAR#2) assessed by quantitative RT-PCR (**a**) and western blot (**b**). **P < 0.01, Normalized by *GAPDH* (**a**) and β-actin (ACTB, **b**). **c** Expression of *OPRK1* in LNCaP, AILNCaP2 (AI2), AILNCaP3 (AI3), and AILNCaP4 (AI4) treated with siRNA for *GFP* (siControl) and *AR* (siAR#1 and siAR#2) assessed by quantitative RT-PCR. **P < 0.01, Normalized by *GAPDH*. **d** Expression of *AR* (left) and *OPRK1* (right) in LNCaP cultured in media supplemented with 10%FBS, charcoal-stripped FBS (CSFBS), and CSFBS plus 1 nM dihydrotestosterone. **P < 0.01, Normalized by *GAPDH*. **e** Chromatin immunoprecipitation (ChIP) using anti-AR antibody and control IgG followed by quantitative RT-PCR using two primers for *OPRK1* promoter (OPRK1-a, and -b) in LNCaP cells cultured as in **d**. **P < 0.01. **f**, **g** ChIP using anti-AR antibody and control IgG followed by quantitative RT-PCR using two primers for *OPRK1* promoter (OPRK1-a, and -b) in KUCaP2 AD (**f**) and CR (**g**) tumors. **P < 0.01. **h** Expression of *AR* in LNCaP, AILNCa*P*2 (AI2), AILNCaP3 (AI3), and AILNCaP4 (AI4) treated with siRNA for *GFP* (siControl) and *OPRK1* (siOPRK1#1 and siOPRK1#2) assessed by quantitative RT-PCR. Normalized by *GAPDH*. **i** Expression of *ORPK1* normalized by *GAPDH* (left) and cell proliferation (right) in LNCaP, AILNCaP2 (AI2), AILNCaP3 (AI3), and AILNCaP4 (AI4) treated with siRNA for *GFP* (siControl) and *OPRK1* (siOPRK1#1 and siOPRK1#2). *P < 0.05, **P < 0.01. **j** Summarized cell proliferation rates of indicated cells with regard to the concentration of supplemented nor-BNI, OPRK1 inhibitor. *P < 0.05.

As for *OPRK1*, we first investigated association with AR signaling using LNCaP, AILNCaP2, AILNCaP3, and AILNCaP4 cells. When we successfully silenced the AR expression using siRNA (Fig. 4a, b), expression of *OPRK1* was significantly upregulated in the four cell lines (Fig. 4c). In androgen-dependent LNCaP, cells cultured in CSFBS (LNCaP CSFBS) showed significant elevation of *OPRK1* expression compared with LNCaP cultured in 10%FBS (LNCaP FBS) and supplementation with 1 nM DHT (LNCaP DHT) counteracted it (Fig. 4d), suggesting that androgen deprivation induces *ORPK1* expression. To further investigate the involvement of AR and androgen, AR ChIP samples from LNCaP FBS, LNCaP CSFBS, and LNCaP DHT were subject to quantitative PCR using two distinct primers for *OPRK1*. As shown in Fig. 4e, ARBS on *OPRK1* was significantly more enriched in LNCaP cultured in the presence of androgen compared with those cultured in the absence of androgen. Our AR-ChIP seq showed enrichment of ARBS on *OPRK1* both in KUCaP2 AD and CR tumors (Fig. 3c). Additionally, an independent AR-ChIP followed by qRT-PCR confirmed that ARBS on *OPRK1* was enriched both in KUCaP2 AD (Fig. 4f) and CR (Fig. 4g) tumors, although the extent of enrichment was higher in AD than CR tumors. We also confirmed that siRNA silencing of *OPRK1* did not affect the expression of the *AR* (Fig. 4h).

Next, we looked at effect of siRNA silencing of *OPRK1* on the proliferation of LNCaP and AILNCaP cells. When *OPRK1* was knocked down by either of two distinct siRNAs, proliferation of AILNCaP2 to 4 was inhibited by 30–40%, whereas proliferation of LNCaP was not affected (Fig. 4i). Since OPRK1 is seven transmembrane G protein-coupled opioid receptor that functions as receptor for endogenous alpha-neoendorphins and dynorphins, we investigated whether pharmacological blockade of the receptor inhibited PCa cell proliferation. We used nor-Binaltorphimine (nor-BNI, ab120078, Abcam, Cambridge, UK), an OPRK1 antagonist, since it is known as the standard OPRK1 inhibitor and has been widely used for in vivo administration experiments. We observed that nor-BNI did not affect expressions of *AR* or *KLK3* in LNCaP or VCaP cells or AR or OPRK1 in AILNCaP or AIVCaP (described below) cells (Supplementary Fig. 10). Consistent with the results from siRNA knockdown, nor-BNI inhibited proliferation in AILNCaP2 to 4 but not in LNCaP (Fig. 4j). It did not affect proliferation of AR-negative PCa cell lines including PC3 and DU145. These results indicated that suppression of AR activity induced expression of *OPRK1*, which seemed promising as a therapeutic target based on the loss-of-function experiments using AILNCaP cells.

### OPRK1 is induced by androgen blockade and promising as a therapeutic target in a CRPC model of AIVCaP cells

**Fig. 5:**
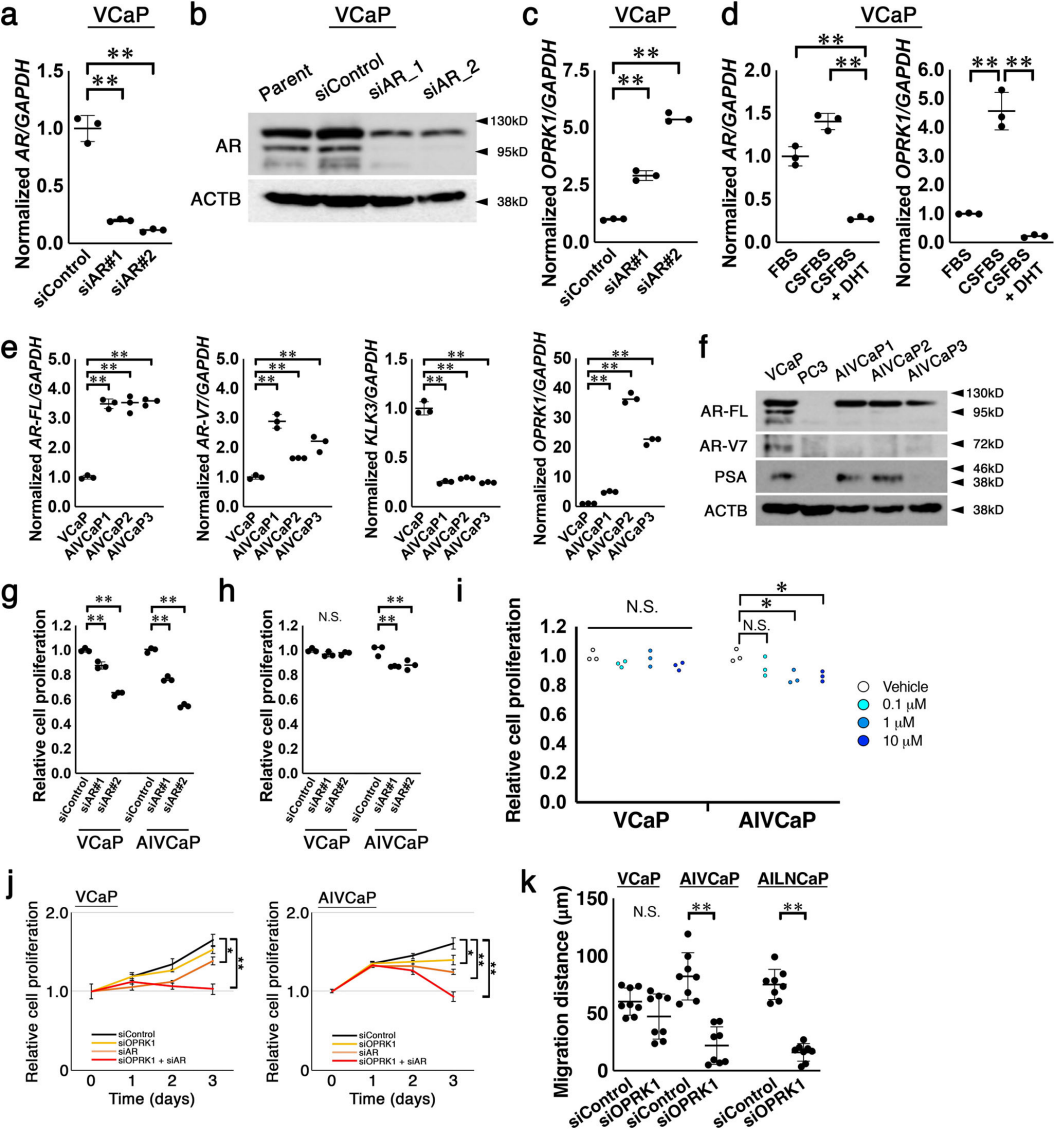
AR suppression upregulates OPRK1, which underpins androgen-independent growth of androgen-independent derivatives of VCaP cells. **a**, **b** Expressions of *AR* in VCaP treated with siRNA for *GFP* (siControl) and *AR* (siAR#1 and siAR#2) assessed by quantitative RT-PCR (**a**) and western blot (**b**). **P < 0.01, Normalized by *GAPDH* (**a**) and β-actin (ACTB, **b**). **c** Expression of *OPRK1* in VCaP treated with siRNA for *GFP* (siControl) and *AR* (siAR#1 and siAR#2) assessed by quantitative RT-PCR. **P < 0.01, Normalized by *GAPDH*. **d** Expression of *AR* (left) and *OPRK1* (right) in VCaP cultured in media supplemented with 10% FBS, charcoal-stripped FBS (CSFBS), and CSFBS plus 1 nM dihydrotestosterone (DHT) assessed by quantitative RT-PCR. **P < 0.01, Normalized by *GAPDH*. **e** Summarized results of quantitative RT-PCR for full-length *AR* (*AR-FL*), *AR* variant 7 (*AR-V7*), *KLK3*, and *OPRK1* normalized by *GAPDH* in indicated cells. **P < 0.01. **f** Western blot of indicated proteins in VCaP, PC3, AIVCaP1, AIVCaP2, and AIVaP3. β-actin (ACTB) acted as loading control. **g** Summarized results of proliferation rates of VCaP and AIVCaP2 (AIVCaP) treated with siRNA for *GFP* (siControl) and *AR* (siAR#1 and siAR#2). **h** Summarized results of proliferation rates of VCaP and AIVCaP2 treated with siRNA for *GFP* (siControl) and *OPRK1* (siOPRK1#1 and siOPRK1#2). **i** Summarized cell proliferation rates of VCaP and AIVCaP2 (AIVCaP) with regard to the concentration of supplemented nor-BNI, OPRK1 inhibitor. *P < 0.05. **j** Cell proliferation in VCaP (left) and AIVCaP2 (AIVCaP) treated with siRNA for *GFP* (siControl), *AR* (siAR) *OPRK1* (siOPRK1), or siAR plus siOPRK1. *P < 0.05, **P < 0.01. **k** Summarized results of migration (scratch) assay with greater distance indicating higher migration ability. VCaP, AIVCaP, and AILNCaP cells were treated with indicated siRNA and subject to the assay. **P < 0.01.

To further investigate the potential opportunity of OPRK1 as a therapeutic target in CRPC, we adopted VCaP cells as another experimental model since VCaP cells express the wild-type AR and OPRK1 and were known to phenotypically recapitulate acquired castration resistance under low androgen culture condition^27^. When we silenced expression of AR using either of two distinct siRNAs (Fig. 5a, b), expression of *OPRK1* was upregulated VCaP cells (Fig. 5c), whereas expressions of *NMNAT2* (Supplementary Fig. 11) and *CLSTN2* (Supplementary Fig. 7a) were unchanged and downregulated, respectively, in consistent with LNCaP cells. It was also consistent with LNCaP cells that androgen deprivation-induced *OPRK1* expression in VCaP cells as well (Fig. 5d). Then we established androgen-independent sublines of VCaP, namely AIVCaP (androgen-independent VCaP) 1, AIVCaP2, and AIVCaP3, according to a previous report^27^. AIVCaPs expressed 3–4-fold of full-length *AR*, 2–3-fold of *AR-V7*, one-fifth of *KLK3*, and 5–40-fold of *OPRK1* in the mRNA level (Fig. 5e). In the protein level, AIVCaPs expressed at least equivalent abundance of AR protein compared with the parental VCaP cells (Fig. 5f), although the OPRK1 expression, proliferation and migration of those cells were not affected by androgen manipulation (Supplementary Fig. 12a).

Since AIVCaP cells express AR-FL as well as AR-V7, we knocked down each of AR-FL or AR-V7 using siRNAs that specifically recognize each variant to dissociate the effect of the two transcriptional variants on the expression of *OPRK1*. We confirmed that treatment with each siRNA successfully knocked down target AR variants (Supplementary Fig. 12b). *OPRK1* mRNA expression was elevated by knocking down AR-FL, whereas it was not affected by knocking down AR-V7 (Supplementary Fig. 12c), suggesting that AR-FL, but not AR-V7 has the suppressing effect on *OPRK1* expression. Although the authors wanted to clarify whether the differential effects between the two AR variants on *OPRK1* expression are attributed to difference in the binding ability to ARBS around *OPRK1* or in the inhibitory ability after binding to the ARBS, it could not be concluded in the present study since we failed to optimally perform ChIP using the variant-specific antibodies.

Silencing AR by siRNAs suppressed the cell proliferation of VCaP and AIVCaP cells by 20–40% (Fig. 5g), suggesting that AIVCaP was at least partially dependent on AR to a similar degree to VCaP cells. On the other hand, genetic silencing of *OPRK1* inhibited the cell proliferation of AIVCaP but not VCaP (Fig. 5h). Pharmacological blockade of OPRK1 also suppressed the cell proliferation of AIVCaP in a dose-dependent manner but not VCaP (Fig. 5i). Accordingly, combined knockdown of *AR* and *OPRK1* effectively suppressed cell proliferation of both VCaP and AIVCaP (Fig. 5j). Additionally, OPRK1 knockdown decreased cell migration in AIVCaP and AILNCaP cells but not LNCaP cells (Fig. 5k). These results collectively suggest that OPRK1 can be a therapeutic target at both settings in castration-sensitive PCa upon castration and in castration-resistant PCa with or without AR axis blockade.

### Pharmacological blockade of OPRK1 retards acquisition of castration-resistant progression and slows pre-acquired castration-resistant tumor growth in in vivo preclinical mouse models of CRPC

We investigated in vivo efficacy of OPRK1 blockade using preclinical mouse models. To ensure safety, non-tumor-bearing mice were castrated and then treated with vehicle or nor-BNI (10 mg/kg i.p. 3 days a week). We found no significant difference in body weight at least up to 10 weeks after the treatment initiation (Supplementary Fig. 13).

**Fig. 6:**
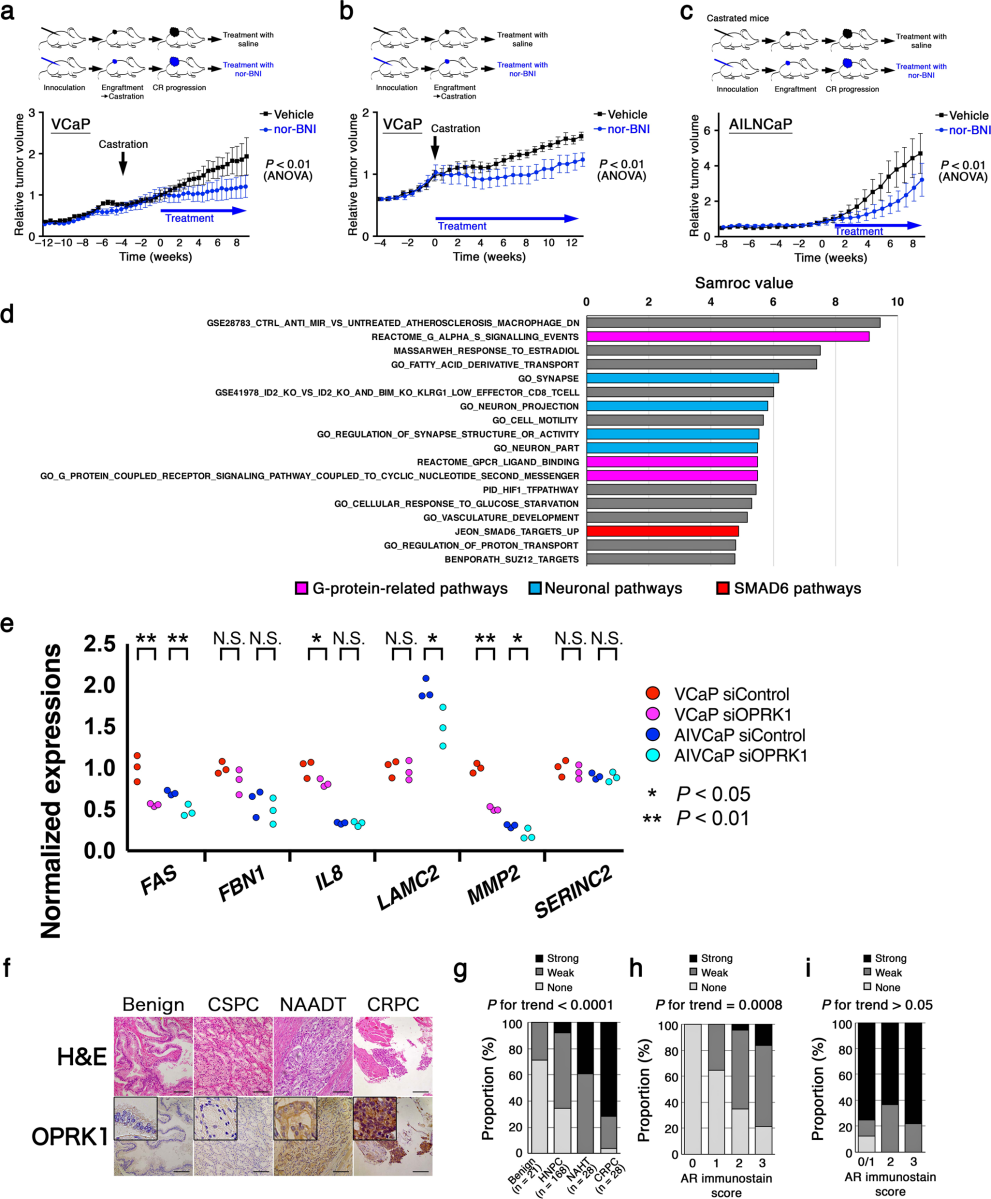
Pharmacological inhibition of OPRK1 suppresses tumor growth in multiple in vivo castration-resistant prostate cancer models. **a** Mice bearing VCaP cell-derived xenograft were castrated. Four weeks later, when the tumor started castration-resistant growth, mice were untreated or treated with OPRK1 inhibitor nor-BNI (*n* = 5 each). **b** Mice bearing VCaP cell-derived xenograft were castrated and untreated or treated with nor-BNI (*n* = 5 each) at the same time. **c** AILNCaP cells were inoculated to castrated mice. When the cell-derived xenograft tumors were engrafted and started growing, mice were untreated or treated with nor-BNI (*n* = 5 each). **d** Single-sample GSEA (ssGSEA) showing differentially enriched gene sets in VCaP cells treated with siRNA for *AR* (siAR) alone vs those treated with siAR and siRNA for *OPRK1* (siORPK1). A gene set involved in SMAD6 pathway (red column) is enriched as well as some gene sets involved in neuronal pathways (light blue) and G-protein-related pathways (magenta). **e** Expressions of six genes involved in the SMAD6 pathway (JEON_SMAD6_TARGETS_UP) were evaluated using quantitative RT-PCR in VCaP or AIVCaP cells under AR signal suppression between siNTC and siOPRK1 treatments. **f** Representative photomicrograph images of hematoxylin and eosin (H&E) and immunohistochemical stains for OPRK1 in non-cancer prostate (benign), castration-sensitive (CSPC) prostate cancer, prostate cancer treated with neoadjuvant androgen deprivation therapy (NAADT), and castration-resistant prostate cancer (CRPC) tissues. **g** Immunostainability of OPRK1 classified into negative (none), weakly (weak), and strongly (strong) positive in benign, hormone-naïve (HNPC), PCa after neoadjuvant hormone therapy (NAHT), and CRPC. **h** Immunostainability of OPRK1 in HNPC tissues with regard to AR immunostainability. **i** Immunostainability of OPRK1 in CRPC tissues with regard to AR immunostainability.

Next, we established VCaP xenograft that temporarily stopped growing upon castration and started re-growing in about 4 weeks, mimicking acquired castration resistance of PCa. When the tumors started castration-resistant growth, tumor-bearing mice were treated with vehicle or nor-BNI (*n* = 5 each). Tumors on mice treated with nor-BMI showed significant growth retardation compared with those on vehicle-treated mice (*P* < 0.01, ANOVA, Fig. 6a). Then we interrogated the effect of nor-BNI when used upon castration. Mice bearing VCaP xenograft were castrated and administered with vehicle or nor-BNI (*n* = 5 each). Tumors on vehicle-treated mice acquired the ability to grow under castrated condition in about 4 weeks, whereas those on nor-BNI-treated mice took about 8 weeks (*P* < 0.01, ANOVA, Fig. 6b). We further evaluated the efficacy of nor-BNI using another CRPC model based on AILNCaP cells. Cells were inoculated on castrated mice. When we confirmed the engraftment and castration-resistant growth of the tumor about 6 weeks after inoculation, we treated the mice with vehicle or nor-BNI (*n* = 5 each). We observed similar significant growth retardation in tumors on nor-BNI-treated mice compared with those on vehicle-treated mice (*P* < 0.01, ANOVA, Fig. 6c) in this CRPC model as well.

To gain further insight with regard to biological function of OPRK1 under androgen-depleted condition, we set up two distinct comparisons using VCaP and AIVCaP cells. VCaP or AIVCaP were treated with siRNA for *AR* plus siRNA for *OPRK1* (combo-siRNA) or siRNA for *AR* alone (single siRNA) (*n* = 4 each), and then expression profile of the respective cell line treated with combo-siRNA was compared with that of cells treated with single siRNA. Based on gene expression clustering, cells treated with combo-siRNA and single siRNA were well discriminated (Supplementary Fig. 14a, b), suggesting that knockdown of *OPRK1* affected substantially biological process of VCaP and AIVCaP cells with *AR* knockdown. Of note, there are markedly more differentially expressed genes in AIVCaP than in VCaP; 51 upregulated and 69 downregulated genes for VCaP compared with 243 upregulated and 601 downregulated genes for AIVCaP with absolute fold change ≥1.5 (Supplementary Fig. 14c–f), suggesting the larger impact of *OPRK1* knockdown in AIVCaP cells than in VCaP cells.

To interrogate biological pathways affected by OPRK1 under castrated condition, we performed single-sample gene set enrichment analysis (ssGSEA)^28^ and samroc analysis^29^ using gene expression profiles obtained from VCaP and AIVCaP as above. Pathways commonly identified in VCaP (FDR < 0.075) and in AIVCaP (FDR < 0.001) treated with combo-siRNA or single siRNA were screened. As anticipated, there were several gene sets related to G protein-coupled receptor signaling and neuronal signaling, suggesting that *OPRK1* expression was effectively altered by siRNA silencing. Among commonly altered pathways, we identified a gene set related to the SMAD6 signaling pathway (samroc value 4.89, Fig. 6d). We confirmed some of the involved genes were downregulated by knocking down *OPRK1* under AR signal suppression (Fig. 6e), suggesting the SMAD6 pathway was affected by OPRK1 blockade under castrated conditions.

We then asked clinical relevance of OPRK1 expression using immunohistochemistry (IHC) on human benign prostate and PCa tissues. OPRK1 antibody for IHC was validated by differential stainability between KUCaP2 AD and KUCaP2 CR tissues (Supplementary Fig. 15). In accordance with the forementioned results from PCa cells, OPRK1 expression was upregulated in PCa tissue obtained after neoadjuvant hormone therapy (NAHT) compared with benign prostate and castration-naïve PCa tissues (*P* for trend < 0.0001, Fig. 6f, g). Importantly, OPRK1 expression was even higher in CRPC tissues (Fig. 6f, g). OPRK1 expression showed a positive correlation with that of AR in castration-naïve PCa (*P* for trend = 0.0008, Fig. 6h), whereas the correlation was lost in CRPC tissues (*P* for trend > 0.05, Fig. 6i). Moreover, we evaluated the survival of patients with advanced PCa with regard to *OPRK1* amplification status using c-BioPortal and found that patients with *OPRK1* amplification had significantly shorter survival compared with those without *OPRK1* amplification (*P* = 5.823 × 10^−4^, log-rank test, Supplementary Fig. S5d, e). Taken together, OPRK1 is induced by androgen deprivation therapy (ADT) and can be a promising therapeutic target of human prostate cancer treated with ADT.

## Discussion

The lack of faithful disease models has hampered translational and preclinical research on CRPC. The present study has demonstrated unique characteristics of the KUCaP2 xenograft, particularly its response to androgen deprivation followed by acquisition of castration resistance accompanied with amplification of *AR*. This model can be considered as an improvement in recapitulating AR-dependent CRPC, in which overexpressed AR confers castration resistance^8,11,12^.

It was reported that aberrantly activated AR in CRPC regulates a distinct transcriptional program^13^. On the other hand, it was also reported that the AR transcription program in cell lines was different from that determined using tumor tissue samples^22^. However, AR-ChIP using human PCa tissue is technically challenging due to the difficulty to obtain a sufficient volume of replicate samples from the same patient. Moreover, it is usually difficult to obtain matched tissue samples of castration-sensitive and resistant tumors. In order to overcome these problems, we have utilized tumors obtained from our xenograft models, which have been limited in the literature with respect to PCa. Repeated sampling of tissues from castration-sensitive and -resistant tumors enabled an efficient comparative analysis.

OPRK1 is a G-protein-coupled receptor (GPCR) that is widely expressed throughout the nervous system and is physiologically activated by endogenous opioid peptide agonists derived from prodynorphin^30,31^. Functional analyses in the present study revealed that OPRK1 has strong potential to be a therapeutic target for PCa. OPRK1 expression was enhanced by androgen deprivation in multiple AR-expressing PCa cell lines, whereas the expression of many other genes harboring ARBS was positively regulated by androgens. Our observation is consistent with previous reports^27,32,33^ showing that AR negatively regulated *OPRK1* expression. Those studies suggested that *OPRK1* was upregulated when AR was unbound from AR-responsive elements nearby the gene upon androgen deprivation. However, the present study has identified *OPRK1* as a gene located close to an ARBS commonly enriched in KUCaP2 AD and CR tumors. Indeed, in vivo AR-ChIP and RNA seq in the present study showed that KUCaP2 CR tumors overexpressed *OPRK1* despite AR remained bound to the ARBS nearby the gene. It is known that AR recruits various co-factors differentially between AD and CR status^11,12^. In this regard, it was reported that recruitment of lysine-specific demethylase-1 mediated transcriptional suppression of AR-targeted genes including OPRK1^27^. Another explanation is that a specific variant of overexpressed AR in CR tumors binds *OPRK1* without ligand biding^8,11,12^, which could attenuate the suppressing function of OPRK1 expression by AR. It has been reported that AR-V7 differentially represses several genes in CRPC^34^. It is also possible that AR-V7 harbors weaker ability to suppress OPRK1 expression compared with full-length AR, as suggested by our variant-specific knockdown experiments. In that case, it is possible AR-V7 even may play a role as a decoy hindering suppressing function of full-length AR, although we could not evaluate differential binding ability to ARBS around *OPRK1* by variant-specific ChIP assays. Moreover, we did not observe negative correlations between AR and OPRK1 expressions in IHC using CSPC or CRPC tissues, suggesting that AR expression may not be a direct indicator of AR activity in regulating *OPRK1* expression. Thus, there may be distinct, complex mechanisms for transcriptional regulation of *OPRK1* expression between AD and CR tumors, or between in vitro and in vivo. However, multiple studies using multiple models consistently showed that OPRK1 is induced by androgen deprivation, suggesting the potential of ORPK1 as a therapeutic target in CRPC.

Further functional analyses revealed that upregulation of OPRK1 facilitates the survival and progression of PCa under very low androgen conditions. In this regard, it is considered that OPRK1 is induced in response to castration and appears to play a purposeful role as a mechanism of adaptation to the attenuated androgen environment. Indeed, we have demonstrated that pharmacological inhibition of OPRK1 prolonged time to acquisition of castration resistance and retarded castration-resistant tumor growth in multiple in vivo preclinical models of CRPC, suggesting clinical efficacy of OPRK1-targeting treatment for prolonging time to castration-resistant progression of advanced PCa. Furthermore, our RNA seq and IHC analyses showed an increasing trend of OPRK1 expression as PCa progressed from castration-naïve to castration-resistant diseases, which was consistent with the findings from cultured PCa cells^27^ and with multiple databases for advanced PCa showing upregulation of OPRK1 in advanced PCa, particularly in those at high risk for death^21,25,26^.

As a consequence of applying ssGSEA and samroc to gene expression profile in castration-sensitive and -resistant VCaP cells with AR ± OPRK1 silencing, it was suggested that OPRK1 upregulated the SMAD6 signaling pathway under suppression of AR activity. It has been reported that SMAD6 plays critical roles in supporting lung cancer cell growth and survival^35^ and in enhancing the aggressiveness of breast cancer^36^ as a downstream effector of the transforming growth factor-β (TGF-β) signaling pathway. As for downstream effectors of the SMAD6 signaling pathways that were shown to be positively regulated by OPRK1, IL8 has been implicated in castration-resistant progression of PCa^37^ as well as LAMC2 reported to be involved in progression, migration, and invasion of multiple types of cancer^38^. It has been reported that IL8 was also negatively regulated by AR binding to the *IL8* promoter^39^, which is consistent with our observation of IL8 upregulation in androgen-independent PCa cells. The present study may provide another mechanism mediated by OPRK1 to upregulate IL8 expression in PCa cells. In line with the reported functions of potential downstream pathways, OPRK1 knockdown decreased both proliferation and migration of CRPC cells including AIVCaP and AILNCaP. We have not elucidated the underlying link between OPRK1 and SMAD6 pathway, which seems to be subject to future studies. Nonetheless, these results collectively strengthen the potential of OPRK1 as a therapeutic target for the prevention of castration-resistant progression and treatment of CRPC.

In terms of clinical relevance, the importance of delaying castration-resistant progression in the management of advanced, castration-sensitive PCa has been emphasized in recent years^40,41^. It is well known that time to CRPC progression is correlated with OS^42^ and progression to CRPC is associated with a deterioration in quality of life^43^. Our findings indicate that OPRK1 inhibition is expected to prolong time to CRPC progression of hormone-sensitive PCa when used in addition to a potent androgen blockade. OPRK1 inhibition can also be expected to suppress tumor growth after CRPC progression.

Recently, as a result of efforts that have been made in clinical and research fields to subtype metastatic CRPCs, a new subtype concept called “double-negative PC (DNPC)”, characterized by the lack of AR pathway activation or neuroendocrine (NE) traits, has emerged^44,45^. The main models used in the present study represented AR-dependent CRPC (ARPC) and OPRK1 blockade did not seem effective for DNPC as evident by inhibitory effect on the proliferation of PC3 and DU145, which are recognized as models of DNPC. Therefore, in the future clinical use of OPRK-targeted therapy, it will be important to distinguish ARPC, DNPC, and NEPC. In this regard, a previous report by Bluemn et al.^44^ indicated that DNPC could not be distinguished by unique genomic aberrations but could be characterized by FGF and MAPK pathway activation. Additionally, Su et al.^45^ reported that DNPC could be characterized by CCL2 expression driven by Polycomb Repressor Complex 1 (PRC1) and recruiting M2-like tumor-associated macrophages and regulatory T cells. These unique characteristics will be useful for distinguishing DNPC from ARPC.

There are, of course, several limitations in the present study. AR-ChIP was not analyzed in combination with mapping of histone methylation or acetylation markers including H3K9me3, H3K27me3, H3K9ac, and H3K27ac. The number of ARBS defined in this study was slightly higher than previous reports due to the lack of narrowing by such markers. We believe that this could be complemented by other integrated omics analyses such as RNA seq. Expression regulation, associated transcriptional programs, and functional relationships between full-length and treatment-induced variant of AR were not dissociated. Since western blotting using anti-OPRK1 antibody could not be optimized by any means, the quantification of OPRK1 protein by gel analysis or proteomics was not available for the present study, and instead, a complimentary approach by extensive qRT-PCR was executed. Anti-tumor effects of nor-BNI could not be confirmed using KUCaP2 since the PDX line was exterminated after repeat passages. We believe that this could be complemented by extensive experiments using multiple other in vivo preclinical models, which rather proved the generalizability of the anti-tumor effect of nor-BNI as a therapeutic agent for CRPC. Genetic knockdown of OPRK1 was not successfully performed in vivo, which we wish to add to pharmacological blockade. Thus, despite several limitations the conclusions of this study have a potential impact on the development of novel treatment strategies for advanced PCa, proposing a new concept of OPRK1 inhibition to block the adaptation pathway for PCa toward CRPC progression. Given those small molecule inhibitors of OPRK1 such as buprenorphine have already been approved by regulatory agencies, novel pharmaceutical advancements or drug repurposing efforts for PCa can be placed on the short-term horizon.

## Methods

### Reagents, oligos, and antibodies

OPRK1 inhibitor nor-Binaltorphimine (nor-BNI) was purchased from Abcam (ab120078, Cambridge, UK). Primers used for PCR in the study are described in Supplementary Data S5. siRNAs used in the study is listed in Supplementary Data S6. Antibodies used in the study are shown in Supplementary Data S7.

### Cell lines

Human prostate cancer cell lines LNCaP, DU145, PC3, 22RV1, and VCaP were purchased from American Type Culture Collection (Manassas, VA). LNCaP, DU145, PC3, and 22RV1 were cultured in RPMI 1640, while VCaP was cultured in DMEM, supplemented with 10% fetal bovine serum (FBS), 12.5 mM HEPES, penicillin, and streptomycin at 37 °C in humidified air containing 5% CO_2_. AILNCaP1 to 4, androgen-independent sublines of LNCaP were established as described elsewhere^15^ and they were cultured in phenol-red-free RPMI 1640 supplemented with 10% charcoal/dextran-stripped FBS (CSFBS). AIVCaPs were established from the parental VCaP cultured in phenol-red-free DMEM medium with 8% CSFBS plus 2% FBS as reported previously^27^.

### Animal experiments

All experiments involving laboratory animals were conducted in accordance with policies of the Guideline for Animal Experiments of Kyoto University and Fundamental Guidelines for Proper Conduct of Animal Experiment and Related Activities in Academic Research Institutions by the Ministry of Education, Culture, Sports, Science and Technology of Japan. The experimental protocols were approved by the Animal Research Committee at Kyoto University Graduate School of Medicine (MedKyo13551, MedKyo14316, MedKyo15290, MedKyo16161, MedKyo17207, MedKyo18238).

BALB/cAJcl-nu/nu mice were purchased from CLEA Japan, Inc. KUCaP2 was previously established from a locally recurrent tumor (CRPC) after radical prostatectomy^16^. The tumor was passaged to BALB/cAJcl-nu/nu nude male mice (CLEA Japan, Inc., Tokyo, Japan) before the tumor size exceeded 2 cm in diameter. For cell-based xenografts (CDX), VCaP 2.0 × 10^6^ cells in 150 μL of DMEM medium and Matrigel^®^ (Corning Glendale, AZ) (1:1) were subcutaneously injected to the right flank of BALB/cAJcl-nu/nu nude male mice (6–7 weeks old). AILNCaP xenograft model was prepared in a similar way with 3.0 × 10^6^ cells in RPMI medium and Matrigel® (1:1) to the mice under castration at the same time.

For surgical castration, mice were anesthetized with inhaled isoflurane and both testes were gently mobilized to the scrotum, and 7 mm skin incision was made along the midline of the scrotum. The testes were pulled out through the incision and dissected away. The spermatic cord was ligated and the skin incision was closed with 4–0 absorbable suture. Mice in the sham-operation control group were similarly anesthetized, incisions were made, testes were pulled out and put back in the scrotum followed by wound closure.

Blood sampling of xenograft mice was taken from the heart under anesthesia and the serum was gathered by centrifugation. PSA in the serum was estimated by chemiluminescent immunoassay (CLIA) method using Architect® PSA (Abbott, Tokyo, Japan) at FALCO biosystems, Ltd (Kyoto, Japan). Intratumoral androgens were quantified by liquid chromatography tandem mass spectrometry (ASKA Pharma Medical Co., Ltd., Fujisawa, Kanagawa, Japan).

For RNA interference in vivo, the siRNA was delivered using the AteloGene® Local Use in vivo siRNA Transfection Kit (KOKEN, Tokyo, Japan), and administrated around and intra-tumors following the supplier’s protocol under anesthesia every 6 days for a total of four times.

Treatment with nor-BNI in vivo was conducted by intraperitoneal administration (i.p.) to the mice, at a dose of 10 mg/kg for three times a week. Nor-BNI was dissolved in normal saline at a concentration of 1 μg/μL and injected. The control group was treated with vehicle (normal saline). Tumor growth was monitored once or twice a week by caliper measurements. The tumor volume was calculated according to the formula: *V* = *L* × *W*^2^ × 0.5 (mm^3^), where *L* is the largest and *W* is the orthogonal diameter of the tumor.

### siRNA treatment and WST-8 assay

For in vitro siRNA silencing, the cells were transfected with specific siRNA oligos (Supplementary Data S6) using Lipofectamine RNAiMAX (Thermo Fisher Scientific K.K., Tokyo, Japan) according to the manufacturer’s instruction. Specific gene knockdown was confirmed by qRT-PCR. The effect of siRNA or nor-BNI (0.1 mM unless otherwise indicated) treatment on cell proliferation was assessed by WST-8 cell proliferation assay. Cells were seeded to 96-well plates in triplicate and incubated for 24 h and then each siRNA was transfected. Cells were cultured for another 72–96 h and proliferation was assessed every 24 h. Cell Counting Kit-8 (Dojin East, Tokyo, Japan) was added to each well and the absorbance of the formazan product was measured at 450 nm with a spectrophotometric plate reader after 2-h incubation.

### AR-chromatin immunoprecipitation (AR-ChIP)

AR-ChIP was done using SimpleChIP® Plus Enzymatic Chromatin IP Kit (#9005; Cell Signaling Technology) according to manufacturer’s instruction. For ChIP using tissue samples we preferably used fresh tissues, not frozen. The antibodies used in the experiments are listed in Supplementary Data 7. For the quality control of AR-ChIP, the immunoprecipitated DNA samples were validated by quantitative PCR using specific primers to *KLK3* enhancer region (as a positive control locus) and *GAPDH* promoter lesion (as a negative control locus) before sequencing. Library construction (TruSeq ChIP Sample Prep Kit, Illumina) and high-throughput sequencing using an Illumina Hiseq (paired end, 100 bp/read) and mapping of the resulting reads by Bowtie were performed by Hokkaido System Science Co., Ltd. Japan.

Mapped reads were analyzed for peak calls via the MACS software (version 2.0.10.20131216). *q*-value analysis based on KLK3 controls yielded a threshold (*q* = 0.129) which defined genes as ARBS. This threshold was applied genome-wide to each sample, identifying target genes based on either in-gene AR-ChIP peaks or peaks in gene promoter regions, defined as being within 20 kb upstream of a gene. The BEDtools package^46^ was employed to handle the genome arithmetic logic. KUCaP2 AD and CR genes identified through this process were filtered to those present in multiple samples, and then compared to LNCaP-ablated (abl) basal upregulated genes reported in Wang et al.^13^, and the CRPC tissue upregulated genes reported in Sharma et al.^22^.

The abl expression data (GSE11428) was analyzed using the microarray probe value matrix format provided by NCBI GEO, where genes were assigned the maximum expression value of constituent probes, and abl-vehicle expression was compared to AR siRNA-treated cell lines (*n* = 3 per cell line). Three hundred sixty-two genes were obtained by applying the filter criteria of a one-way ANOVA having a maximum *p*-value of 0.001, an average expression fold change of >2.5 in abl lines, and no >10% overlap in the 95% expression level confidence intervals estimated. For the comparison with the genes reported in Sharma et al.^22^, a direct comparison was performed using the gene list provided in the article’s supplementary data.

### Single-sample gene set enrichment analysis (ssGSEA)

In ssGSEA a separate enrichment score for each pairing of sample and gene set is calculated independent of phenotype labeling, while GSEA generates a gene set’s enrichment scores for phenotypic differences across a collection of samples within a dataset. In other words, ssGSEA transforms a single sample’s gene expression profile to a gene sets enrichment profile. The gene set enrichment score represents the activity level of a biological process in which members of the gene sets are coordinately up- or downregulated.

ssGSEA was performed as reported elsewhere^28,47,48^. First, we cleaned our gene expression microarray datasets (58,201 gene probes) up by filtering out those with a poor probe quality flag of A (probe signal intensity over the background) and M (high background probe), which resulted in 20,969 flag P (quality good) probes retained. Then we downloaded all gene sets version 6.2 (*n* = 20,938) from the Molecular Signatures Database (http://www.broadinstitute.org/gsea/msigdb, last accessed January 10, 2019). Using these data and gene sets, we performed ssGSEA to generate scores for predefined signature gene sets, as described (GenePattern version 3.5.0; Broad institute; http://www.broadinstitute.org/cancer/software/genepattern, last accessed July 10, 2018). For data analyses, the ssGSEA scores were normalized from 0 to 1. Samroc^29^ was used for detecting significant differential pathways between combo-siRNA and single siRNA. FDR *q*-values were calculated from Samroc *p*-values using the R library “*p*-adjust”.

### RNA sequence, cRNA microarray, quantitative PCR

Total RNA was extracted using an RNeasy Mini kit (QIAGEN, Hilden, Germany). RNA purity and integrity were evaluated by ND-1000 Spectrophotometer (NanoDrop, Wilmington, USA), Agilent 2100 Bioanalyzer (Agilent Technologies, Palo Alto, USA). RNA-sequencing (RNA seq) libraries were prepared by TruSeq Rapid PE Cluster Kit and TruSeq Rapid SBS kit (Illumina, San Diego, CA). The libraries were sequenced on Illumina HiSeq 2500 using a read length of 2 × 150 bp. RNA seq reads were demultiplexed using CASAVA v1.8.2 and aligned to human transcriptome (UCSC gene) and genome (GRCh37/hg19) references respectively using Burrows–Wheeler Aligner^49^. After transcript coordinate was converted to genomic positions, an optimal mapping result was selected either from transcript or genome mapping by comparing the minimal edit distance to the reference. Local realignment was performed within in-house short reads aligner with a smaller k-mer size (*k* = 11). Finally, fragments per kilobase of exon per million fragments mapped (fpkm) values were calculated for each UCSC gene while considering strand-specific information.

For cRNA microarray, RNA labeling and hybridization were performed by using the Agilent One-Color Microarray-Based Gene Expression Analysis protocol (Agilent Technology, V 6.5, 2010). Briefly, 100 ng of total RNA from each sample was linearly amplified and labeled with Cy3-dCTP. The labeled cRNAs were purified by RNAeasy Mini Kit (Qiagen). The concentration and specific activity of the labeled cRNAs (pmol Cy3/μg cRNA) were measured by NanoDrop ND-1000 (NanoDrop, Wilmington, USA). Then 600 ng of each labeled cRNA was fragmented by adding 5 μl 10x blocking agent and 1 μl of 25x fragmentation buffer and then heated at 60 °C for 30 min. Finally, 25 μl 2x GE hybridization buffer was added to dilute the labeled cRNA. 40 μl of hybridization solution was dispensed into the gasket slide and assembled to the Agilent SurePrint G3 Human GE 8X60K, V3 Microarrays (Agilent®). The slides were incubated for 17 h at 65 °C in an Agilent hybridization oven. then washed at room temperature by using the Agilent One-Color Microarray-Based Gene Expression Analysis protocol (Agilent Technology, V 6.5, 2010). The hybridized array was immediately scanned with an Agilent Microarray Scanner D (Agilent Technologies, Inc.). Microarray results were extracted using Agilent Feature Extraction software v11.0 (Agilent Technologies).

For quantitative RT-PCR, RNA was reverse-transcribed into complementary DNA using a ReverTra Ace qPCR RT Kit (TOYOBO, Osaka, Japan) according to instructions from each manufacturer. Genome DNA was extracted using QIAamp DNA Mini Kit (Qiagen, Hilden, Germany) and subject to genome-based PCR. Quantitative PCR was performed as reported elsewhere^4^ using primers shown in Supplementary Data 5.

### Western blotting and immunoprecipitation

Whole-cell extracts were prepared as previously described^4^. For western blot analysis, aliquots of proteins were separated by SDS–PAGE and transferred to nitrocellulose membrane (Bio-Rad Laboratories). Immunodetection was carried out with the indicated antibodies (Supplementary Data 7) and bound antibodies were visualized with peroxidase-conjugated affinity-purified donkey anti-mouse or anti-rabbit IgG using ECL Plus (Amersham Biosciences), and luminescence images were analyzed by ImageQuant LAS 4000 min, (Fuji Film, Tokyo, Japan). Uncropped images are shown in Supplementary Fig. 13.

### Pathological evaluation

Paraffin sections were cut (5-μm thick), dewaxed in xylene, brought to water down an ethanol gradient, and then subject to hematoxylin and eosin (H&E) or immunohistochemical (IHC) stains. IHC assays for AR and PSA were performed as described previously^4^. For the IHC assay of OPRK1, AR, and PSA, a trained pathologist (Y. T.) and two authors (Y. M. and T.K.) independently scored each staining intensity and discrepant cases were resolved by discussion with all observers.

### Statistics and reproducibility

All experiments are triplicate and the data are presented as mean ± S.E.M. unless otherwise indicated. Student’s *t*-test was used for the comparison of numerical data while ANOVA was used for the comparison of repeated numerical data. Chi-square of Fisher’s exact tests were used for contingency analyses. Kruskal–Wallis test was also used for non-parametric comparison between three groups. All statistical analyses were performed using commercially available software (SPSSII, SPSS Japan Inc.). All tests were two-sided and *P*-values < 0.05 were considered statistically significant.

### Reporting summary

Further information on research design is available in the Nature Research Reporting Summary linked to this article.

## Supplementary information

Supplementary Information

Description of Additional Supplementary Files

Supplementary Data S1

Supplementary Data S2

Supplementary Data S3

Supplementary Data S4

Supplementary Data S5

Supplementary Data S6

Supplementary Data S7

Reporting Summary

## Data Availability

ChIP sequence data that support the findings of this study have been deposited in DDBJ Sequence Read Archive (DRA) with the accession codes DRA013524 and DRA013551. RNA sequence data that support the findings of this study have been deposited in DDBJ Sequence Read Archive (DRA) with the accession code DRA013525. cDNA microarray data have been deposited in GEO repository with the accession codes GSE194248.
