# Author Correction: Comprehensive genomics in androgen receptor-dependent castration-resistant prostate cancer identifies an adaptation pathway mediated by opioid receptor kappa 1

**DOI:** 10.1038/s42003-022-03707-z

**Published:** 2022-07-19

**Authors:** Yuki Makino, Yuki Kamiyama, J. B. Brown, Toshiya Tanaka, Ryusuke Murakami, Yuki Teramoto, Takayuki Goto, Shusuke Akamatsu, Naoki Terada, Takahiro Inoue, Tatsuhiko Kodama, Osamu Ogawa, Takashi Kobayashi

**Affiliations:** 1 Department of Urology, Kyoto University Graduate School of Medicine, Kyoto, Japan; 2 Laboratory of Molecular Biosciences, Kyoto University Graduate School of Medicine, Kyoto, Japan; 3 Center for Cancer Immunotherapy and Immunobiology, Kyoto University Graduate School of Medicine, Kyoto, Japan; 4 Research Center for Advanced Science and Technology, The University of Tokyo, Tokyo, Japan; 5 Department of Gynecology and Obstetrics, Kyoto University Graduate School of Medicine, Kyoto, Japan; 6 Department of Diagnostic Pathology, Kyoto University Graduate School of Medicine, Kyoto, Japan

**Keywords:** Cancer therapeutic resistance, Prostate

Correction to: *Communications Biology* 10.1038/s42003-022-03227-w, published online 01 April 2022.

In the original version of the Supplementary information associated with this Article, Supplementary Figure panels S1h, S1i, S5d, S5e were left out.

This has now been corrected.

## Supplementary information

Updated Supplementary information

