# Supplementary material for: Comprehensive genomics in androgen receptor-dependent castration-resistant prostate cancer identifies an adaptation pathway mediated by opioid receptor kappa 1
Source: Commun Biol. 2022 Apr 1;5:299. doi: 10.1038/s42003-022-03227-w (PMC8976065; doi:10.1038/s42003-022-03227-w)
Supplement: Supplementary file 1 — Supplementary Information [file 42003_2022_3227_MOESM1_ESM.pdf]

Supplementary information

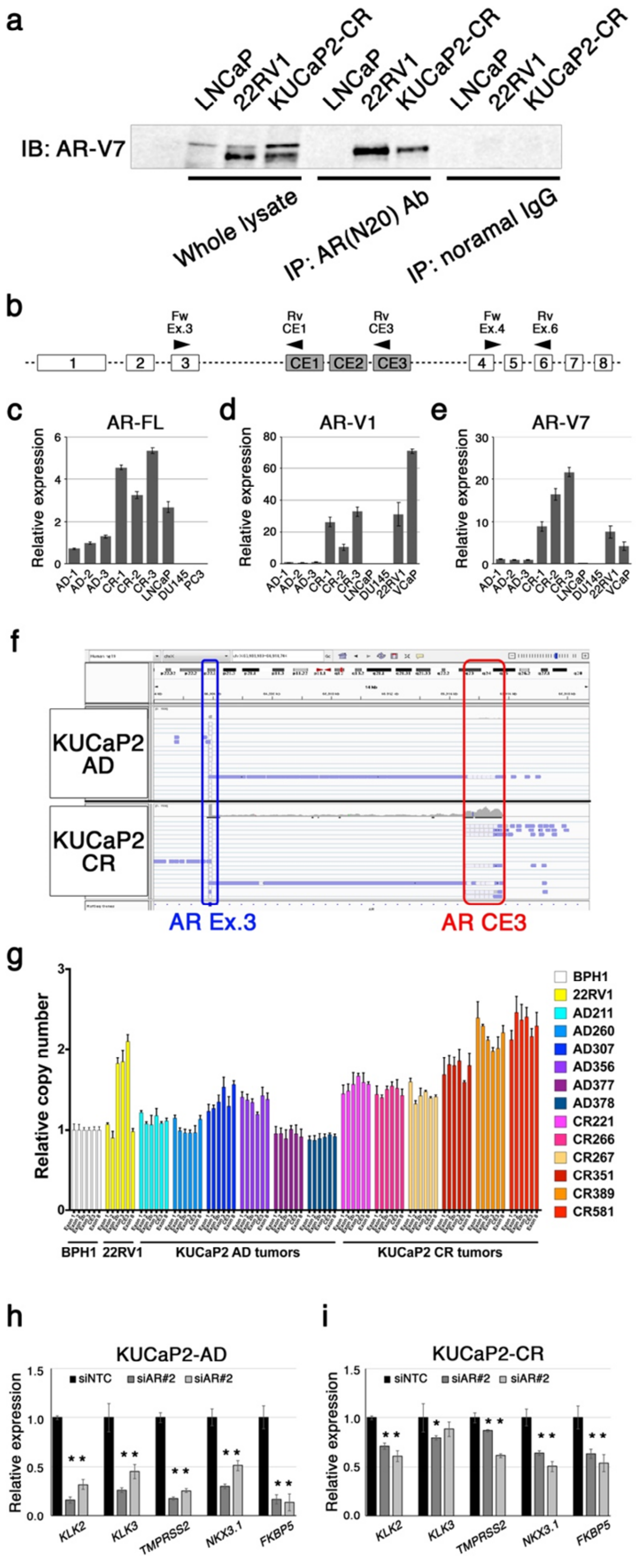
a
LNCaP
22RV1
KUCaP2-CR
IB: AR-V7
Whole lysate
IP: AR(N20) Ab
IP: noramal IgG
b
Fw Ex.3
Rv CE1
Rv CE3
Fw Ex.4
Rv Ex.6
1
2
3
CE1
CE2
CE3
4
5
6
7
8
c
AR-FL
d
AR-V1
e
AR-V7
Relative expression
AD-1
AD-2
AD-3
CR-1
CR-2
CR-3
LNCaP
DU145
PC3
22RV1
VCaP
f
KUCaP2 AD
KUCaP2 CR
AR Ex.3
AR CE3
g
Relative copy number
BPH1
22RV1
AD211
AD260
AD307
AD356
AD377
AD378
CR221
CR266
CR267
CR351
CR389
CR581
KUCaP2 AD tumors
KUCaP2 CR tumors
h
KUCaP2-AD
i
KUCaP2-CR
siNTC
siAR#2
siAR#2
KLK2
KLK3
TMPRSS2
NKX3.1
FKBP5

Supplementary Fig. S1. *AR* amplification and AD-V7 expression in KUCaP2 CR tumors. **a** Expression of AR-V7 in KUCaP2 was evaluated by immunoprecipitation (IP) using an antibody to N-terminus of AR (AR N20) followed by immunoblotting (IB) using an specific antibody to AR-V7. **b** Schematic design of primers used for RT-PCR for the detection of full-length AR (AR-FL, FwEx.4 and RvEx.6), AR-V1 (FwEx.3 and RvCE1) and AR-V7 (FwEx.3 and RvCE3). **c–e** Charts for relative expression of AR-FL (**c**), AR-V1 (**d**) and AR-V7 (**e**) determined by RT-PCR. **f** RNA sequence showing signals for AR exon 3 (Ex.3) in both androgen-dependent (KUCaP AD, top) and castration-resistant KUCaP2 (KUCaP2 CR, bottom) and for cryptic exon 3 (AR CE3) exclusively in KUCaP2 CR. **g** Relative copy numbers of indicated exons of AR using genome-based quantitative PCR in BPH1 and 22RV1 cells, KUCaP AD tumors (n = 6) and KUCaP CR tumors (n = 6). **h, i** Relative expression of indicated AR-regulated genes in KUCaP AD (**h**) and CR (**i**) tumors treated with or without siRNA for AR.

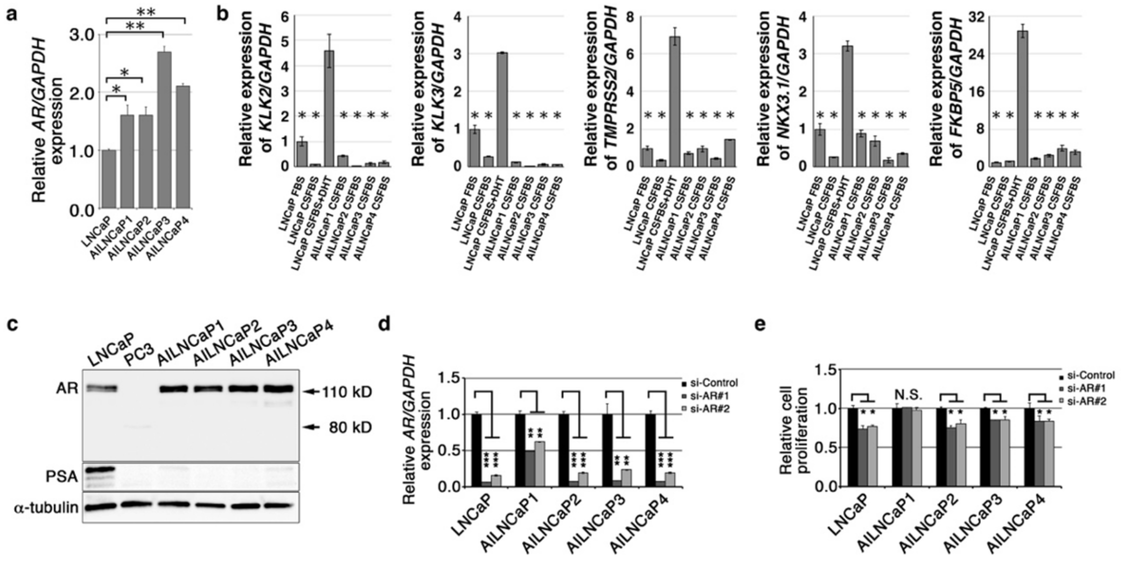

Supplementary Fig. S2. AILNCaP cells as experimental models for AR-expressing CRPC. **a** Expression of *AR* normalized by *GAPDH* for indicated cells using quantitative RT-PCR. *P < 0.05, **P < 0.01. **b** Expression of indicated AR-related genes normalized by *GAPDH* for indicated cells using quantitative RT-PCR. *P < 0.01 for the comparison with “LNCaP CSFBS + DHT”. **c** Abundance of AR and PSA proteins was evaluated using western blotting. α-tubulin acts as loading control. **d, e** Relative expression of *AR* (**d**) and cell proliferation (**c**) for indicated cells treated with control siRNA (si-Ctl) and two distinct siRNAs for AR (si-AR#1 and si-AR#2).

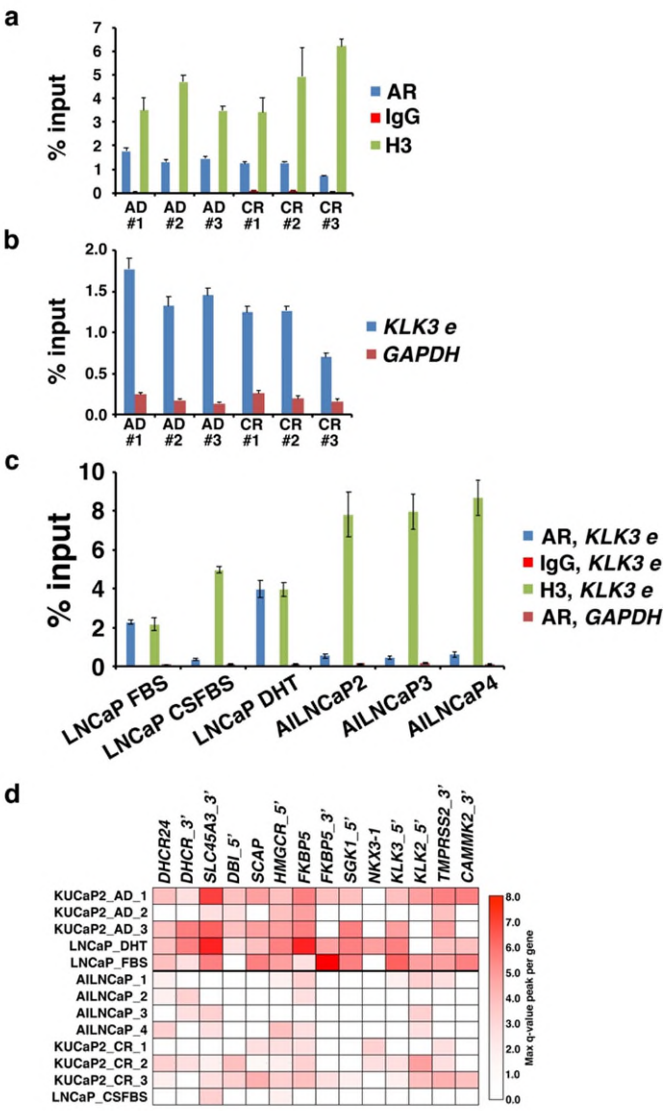

Supplementary Fig. S3. a Immunoprecipitated samples by anti-AR antibody (AR), control normal IgG (IgG), and anti-histone H3 antibody (H3) were subject to quantitative PCR using primers for *KLK3* enhancer. **b** Immunoprecipitated samples by anti-AR antibody were subject to quantitative PCR using primers for *KLK3* enhancer (*KLK3 e*) or negative control locus (*GAPDH* promoter). **c** LNCaP cells were cultured in media containing 10% FBS (LNCaP FBS), 10% charcoal-strip FBS (LNCaP CSFBS), and 10%

charcoal-strip FBS supplemented with 1 nM DHT (LNCaP DHT), while AILNCaP2, 3, and 4 were cultured in media containing 10% CSFBS. Nuclear extracts from indicated cells were immunoprecipitated by anti-AR antibody (AR, *KLK3 e*), control normal IgG (IgG, *KLK3 e*), anti-histone H3 (H3, *KLK3 e*), and then subject to quantitative PCR using primers for *KLK3* enhancer while samples immunoprecipitated by anti-AR antibody were subject to quantitative PCR using primers for *GAPDH* (AR, *GAPDH*). **d** Heatmap of max q-value peak obtained from AR-ChIP for indicated gene sites that were previously reported as target of AR.

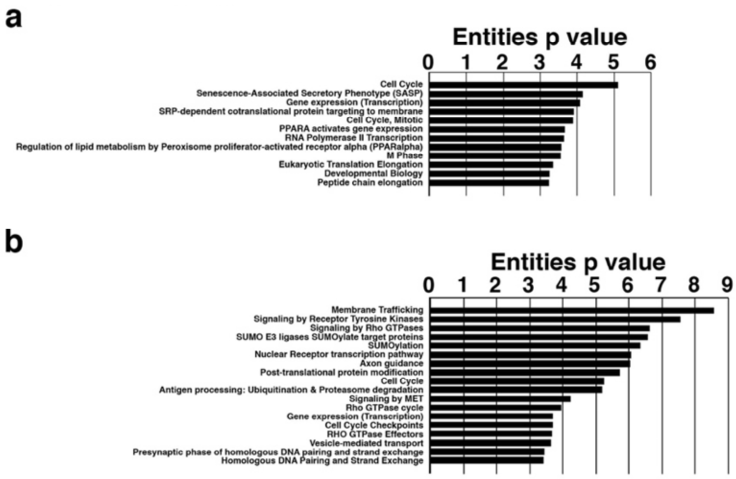

Supplementary Fig. S4. a Reactome pathways for genes commonly identified for KUCaP2 AD and CR tumors annotated by AR-binding sites in AR-ChIP seq with regard to entities p values (–log[p-value]). **b** Reactome pathways for genes exclusively identified for KUCaP2 AD tumors annotated by AR-binding sites in AR-ChIP seq with regard to entities p values (–log[p-value]).

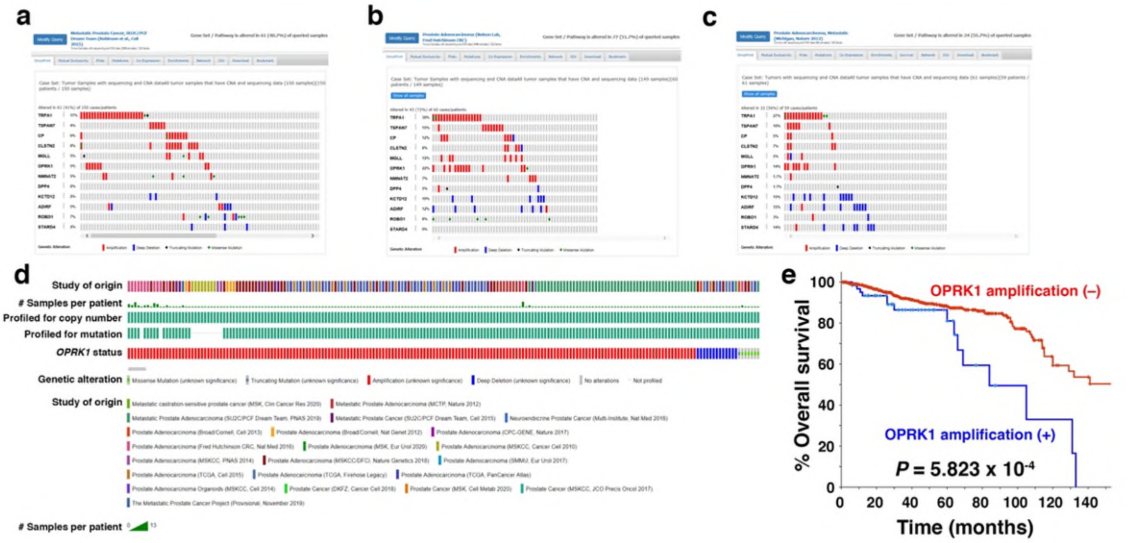

Supplementary Fig. S5. a–c Outputs of C-bioportal in terms of frequently altered genes in metastatic CRPC (**a**, Robinson et al. 2015[21]), metastatic PCa (**b**, Kumar et al. 2016[25]) and lethal CRPC (**c**, Grasso et al. 2012[26]). **d, e**. Outputs of C-BioPortal in terms of *OPRK1* gene alteration status (**d**) and overall survival with regard to amplification of *OPRK1* (**e**).

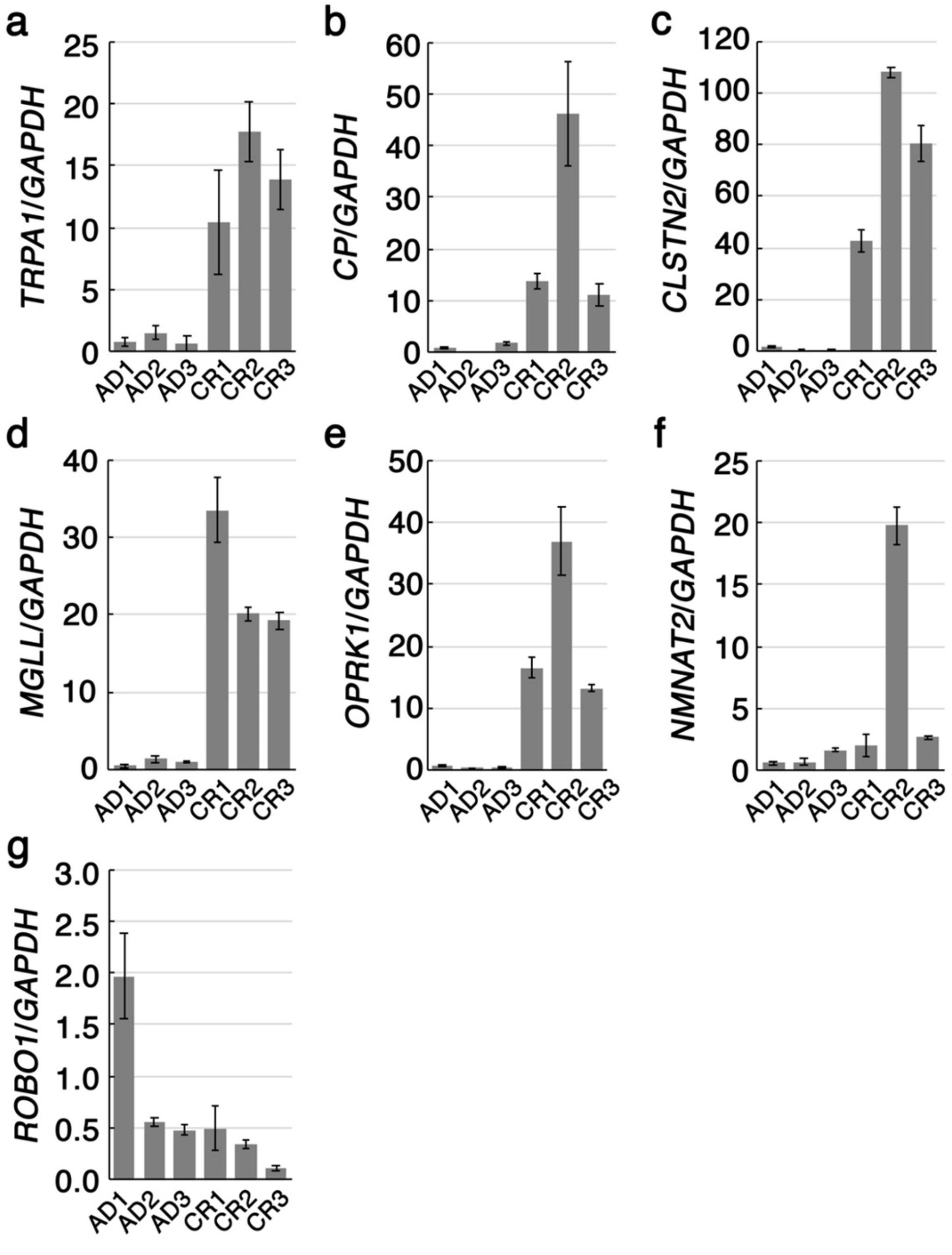

Supplementary Fig. S6. a–h Expressions of indicated genes normalized by *GAPDH* in KUCaP2 AD (AD1–3) and CR (CR1–3) tumors (n = 3 each) using quantitative RT-PCR.

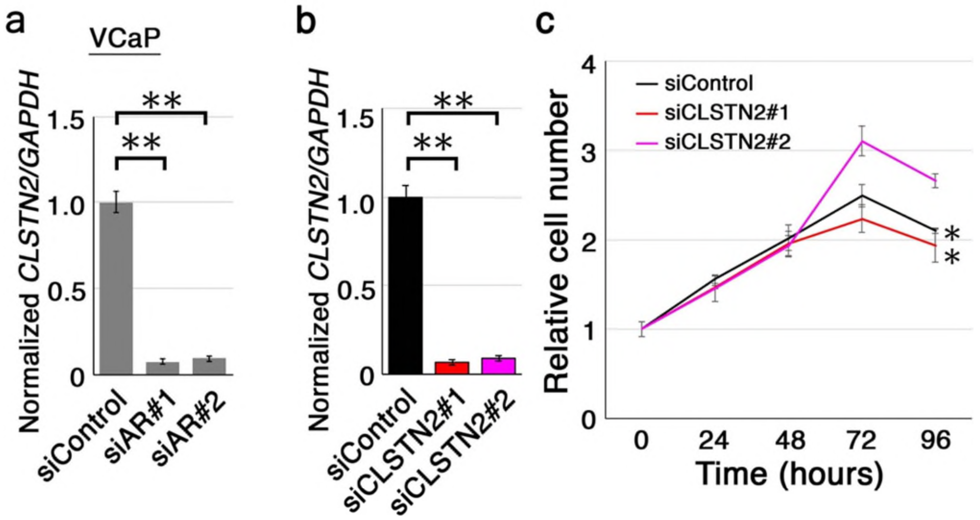

Supplementary Fig. S7. a Expression of *CLSTN2* normalized by *GAPDH* in VCaP cells treated with control siRNA (siControl) or two distinct siRNA for *AR* (siAR#1 and #2) using RT-PCR. **b** Expression of *CLSTN2* normalized by *GAPDH* in indicated cells treated with control siRNA (siControl) or two distinct siRNA for *CLSTN2* (siCLSTN2#1 and #2) using RT-PCR (left) and relative cell proliferation for 96 hours after the treatment (right).

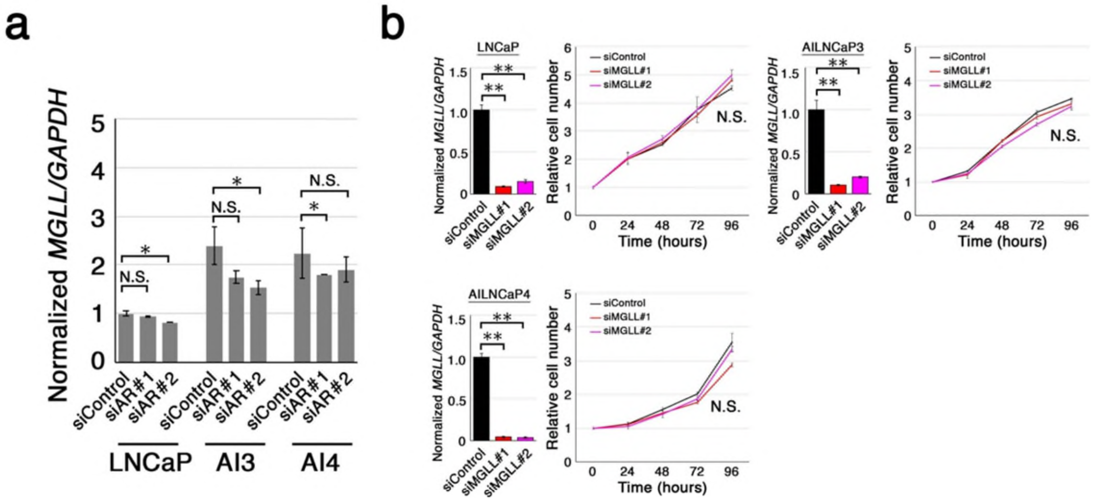

Supplementary Fig. S8. a Expression of *MGLL* normalized by *GAPDH* in indicated cells treated with control siRNA (siControl) or two distinct siRNA for *AR* (siAR#1 and #2) using RT-PCR. **b** Expression of *MGLL* normalized by *GAPDH* in indicated cells treated with control siRNA (siControl) or two distinct siRNA for *MGLL* (siMGLL#1 and #2) using RT-PCR (left) and relative cell proliferation for 96 hours after the treatment (right).

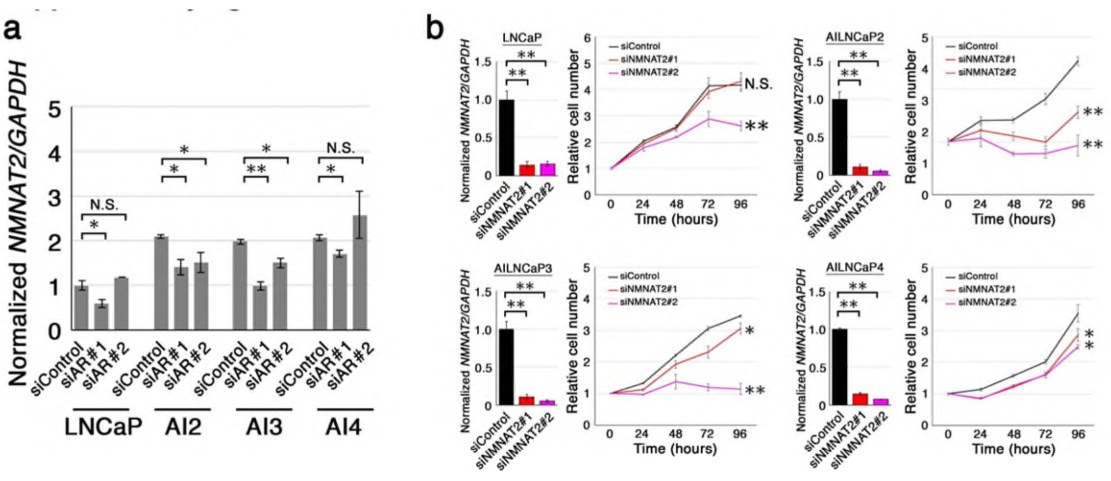

Supplementary Fig. S9. a Expression of *NMNAT2* normalized by *GAPDH* in indicated cells treated with control siRNA (siControl) or two distinct siRNA for *AR* (siAR#1 and #2) using RT-PCR. **b** Expression of *NMNAT2* normalized by *GAPDH* in indicated cells treated with control siRNA (siControl) or two distinct siRNA for *NMNAT2* (siNMNAT2#1 and #2) using RT-PCR (left) and relative cell proliferation for 96 hours after the treatment (right).

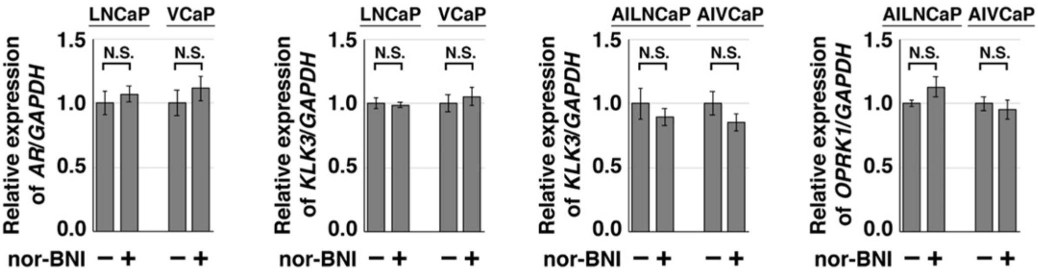

Supplementary Fig. S10. Expression of indicated genes normalized by *GAPDH* in indicated cells treated with vehicle (–) or 0.1 mM nor-BNI (+) for 48 hr using RT-PCR.

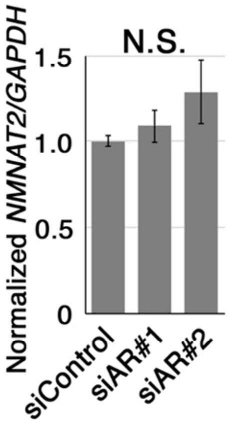

Supplementary Fig. S11. Expression of *NMNAT2* normalized by *GAPDH* in VCaP cells treated with control siRNA (siControl) or two distinct siRNA for *AR* (siAR#1 and #2) using RT-PCR.

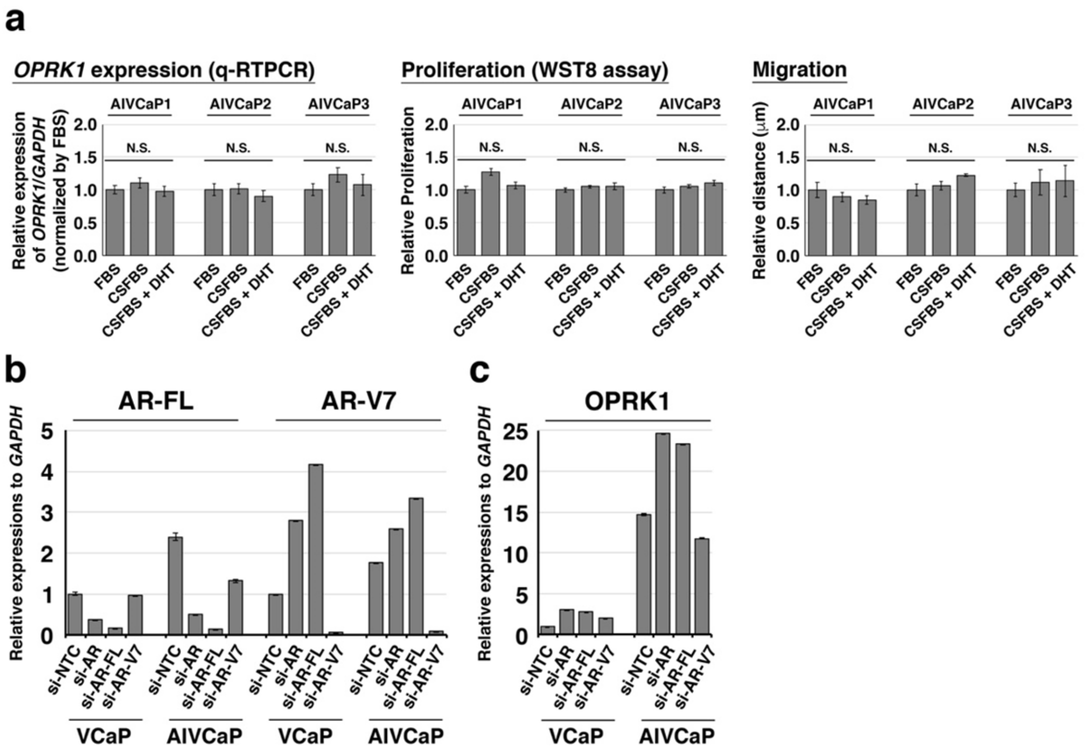

Supplementary Fig. S12. a Expression of *OPRK1*, proliferation and migration of AIVCaP cells cultured in the indicated conditions. **b** Expression of *AR-FL* and *AR-V7* normalized by *GAPDH* in VCaP or AIVCaP cells treated with control siRNA (si-NTC), siRNA for AR-FL and AR-V7 (si-AR), siRNA for *AR-FL* (siAR-FL) or siRNA for AR-V7 (si-AR-V7) using RT-PCR. **c** Expression of *OPRK1* normalized by *GAPDH* in VCaP or AIVCaP cells treated with siRNAs indicated as in **b**.

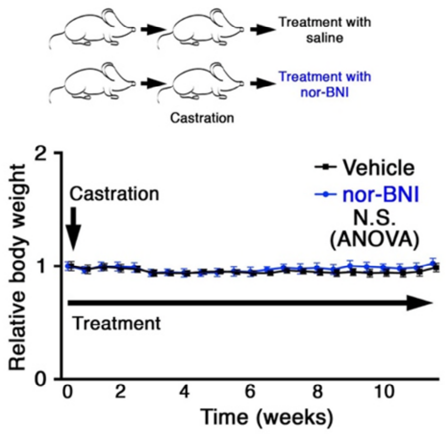

Supplementary Fig. S13. a Experimental scheme of pilot treatment of mice with vehicle or OPRK1 inhibitor nor-BNI following surgical castration. **b** Changes in relative body weight for 10 weeks after treatment initiation (n = 3 each).

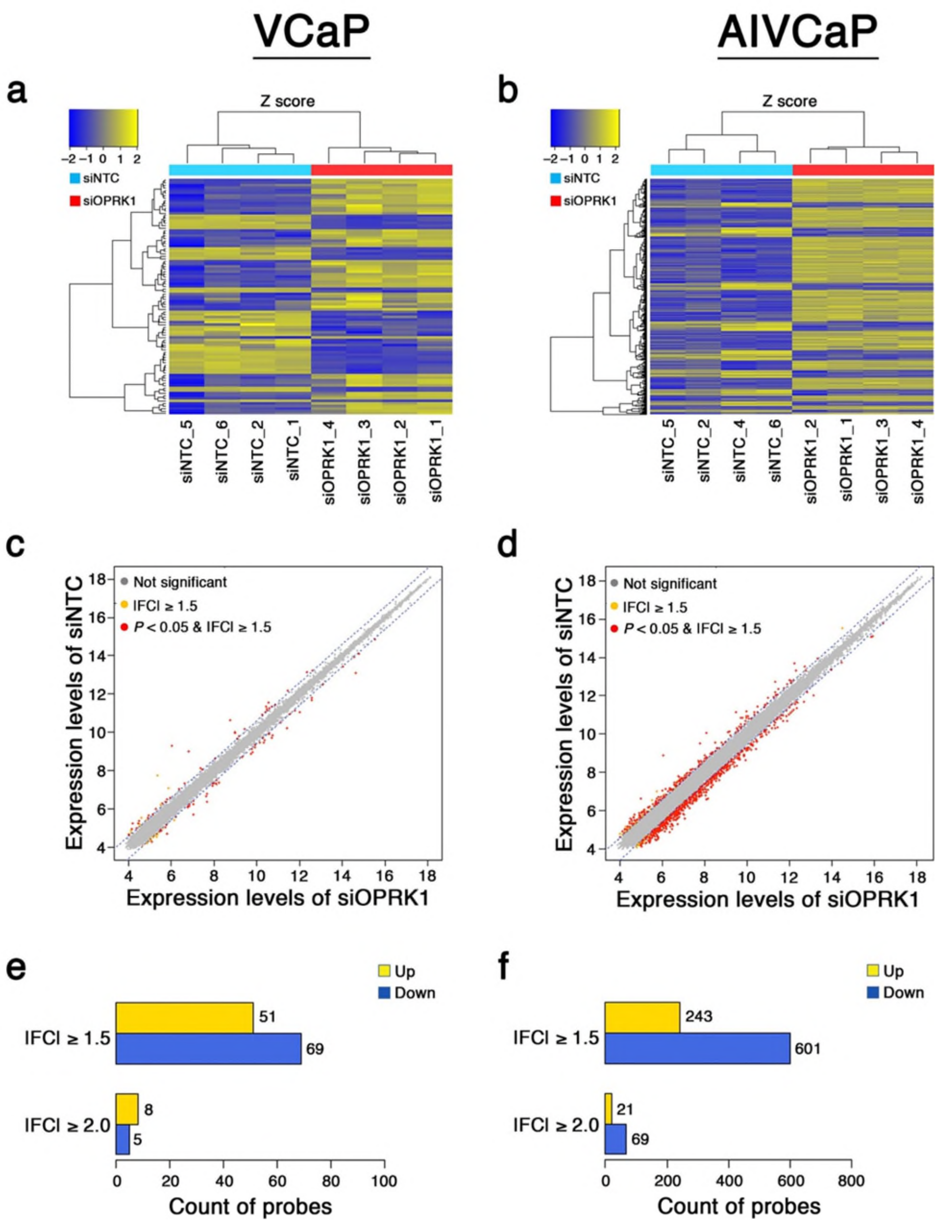

Supplementary Fig. S14. a, b Hierarchial clustering of VCaP (**a**, n = 4) and AIVCaP (**b**, n = 4) cells treated with siRNA for *AR* (siAR) alone or treated with siAR and siORPK1. **c**, **d** Scatter plots showing differentially expressed genes in VCaP (**c**) and AIVCaP (**d**) cells treated with siRNA for *AR* (siAR) alone or treated with siAR and siORPK1. Gray dots indicate genes not significantly changed and yellow dots indicate genes with absolute

fold change (|FC|) ≥ 1.5 but *P* value ≥ 0.05, while red dots indicate genes with |FC| ≥ 1.5 and *P* value < 0.05. **e**, **f** Numbers of significantly changed genes with |FC| ≥ 1.5 (top) and |FC| ≥ 2.0 (bottom) in VCaP (**e**) and AIVCaP (**f**) cells.

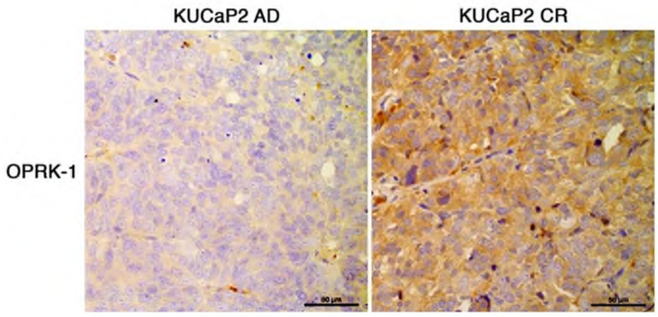

Supplementary Fig. S15. a Representative photomicrograph images of immunohistochemical stainings for OPRK-1 in KUCaP AD and KUCaP2 CR tumors.

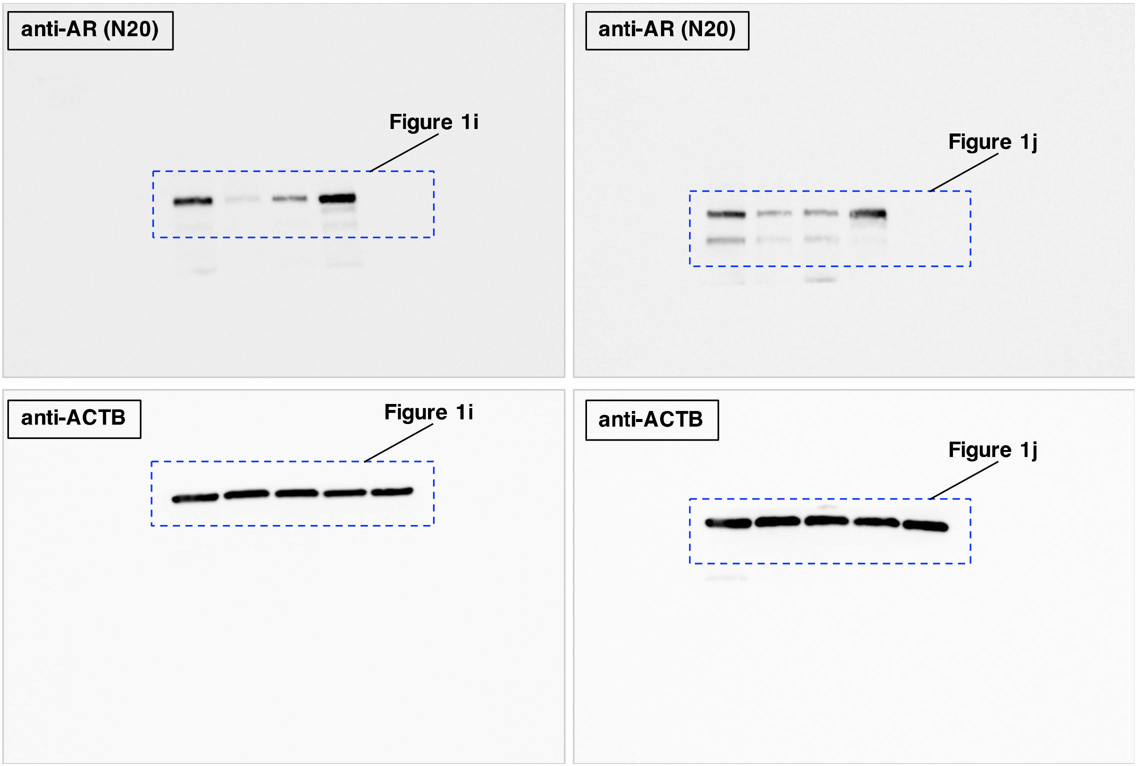

Supplementary Fig. S16. Uncropped gel images for **Figure 1d**

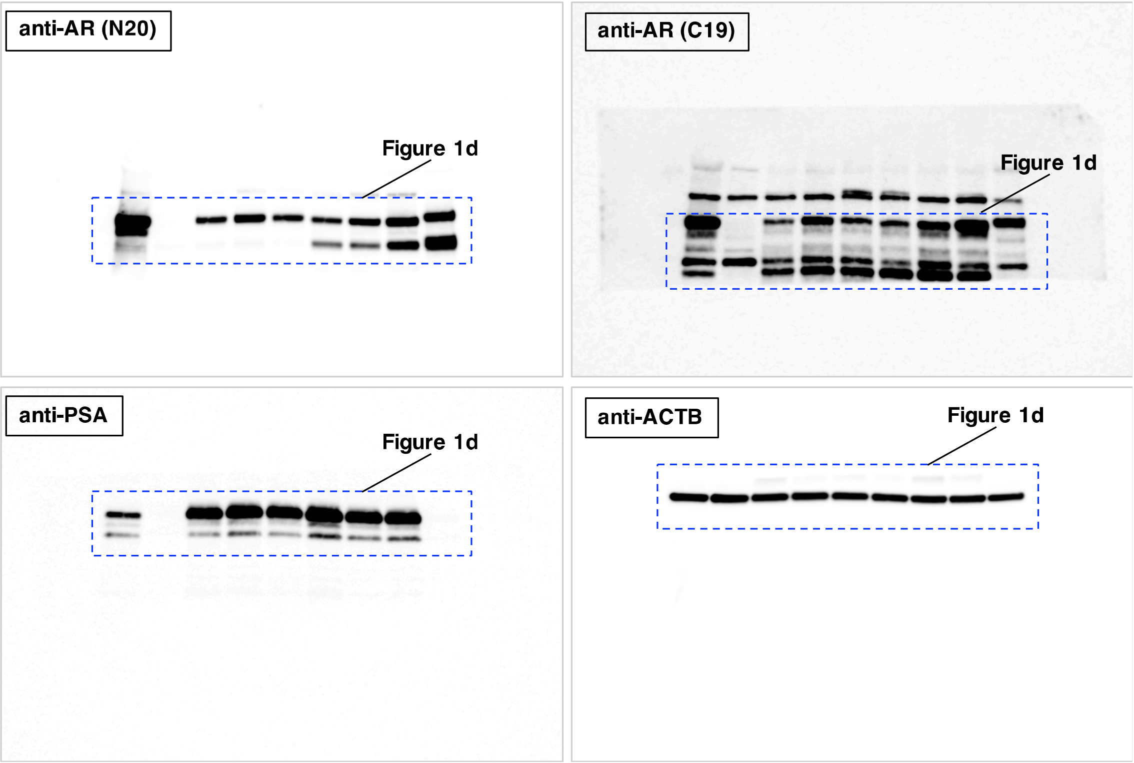

Supplementary Fig. S17. Uncropped gel images for **Figures 1i and 1j**

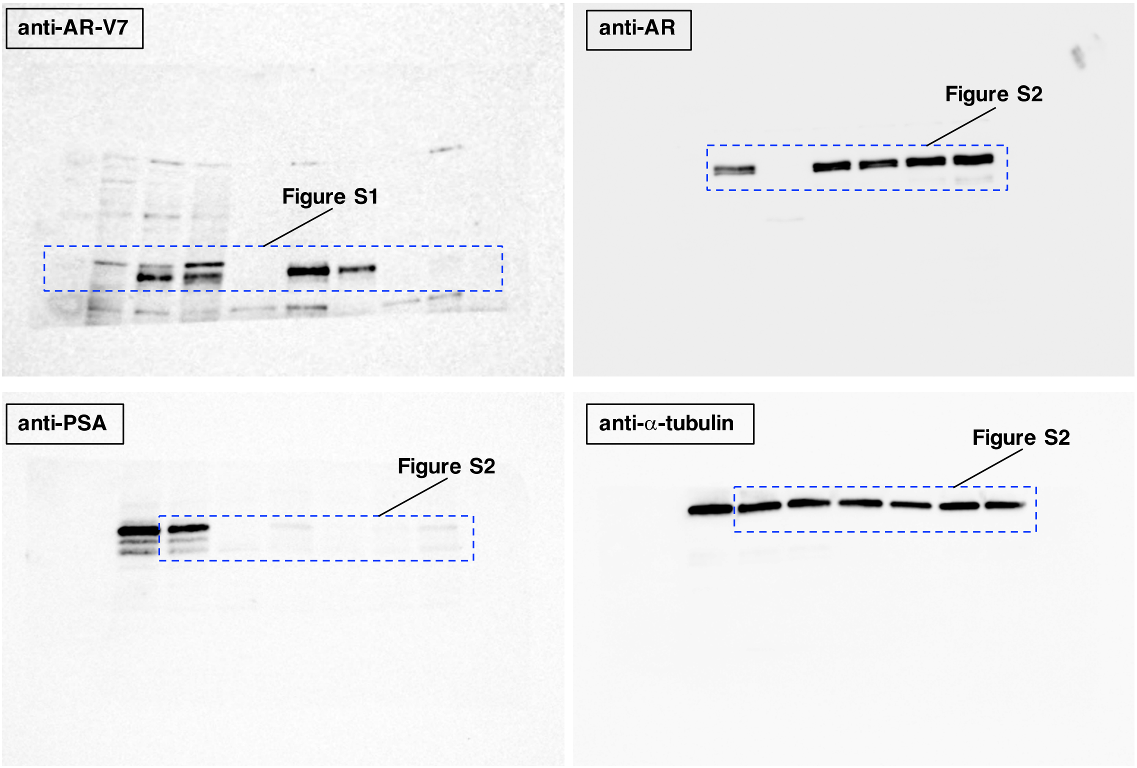

Supplementary Fig. S18. Uncropped gel images for **Supplementary figures S1 and S2**

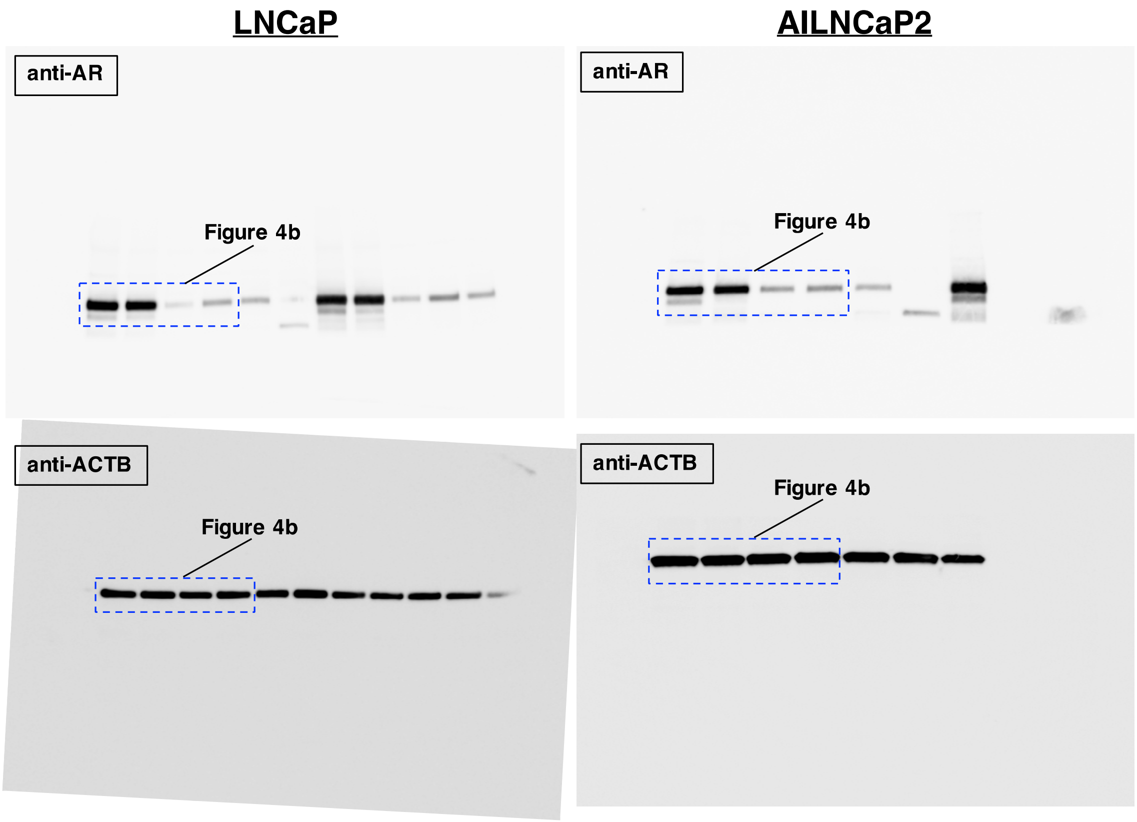

Supplementary Fig. S19. Uncropped gel images for **Figure 4b** (LNCaP, left and AILNCaP2, right)

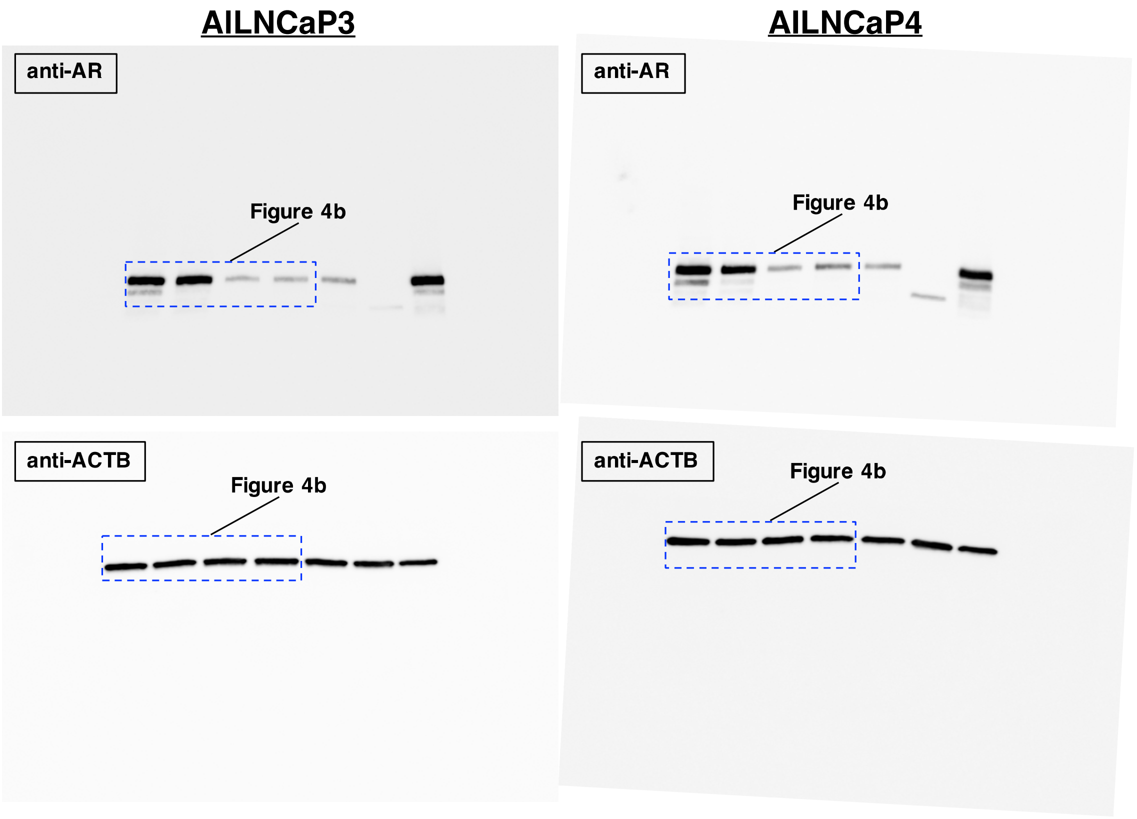

Supplementary Fig. S20. Uncropped gel images for **Figure 4b** (AILNCaP3, left and AILNCaP4, right)

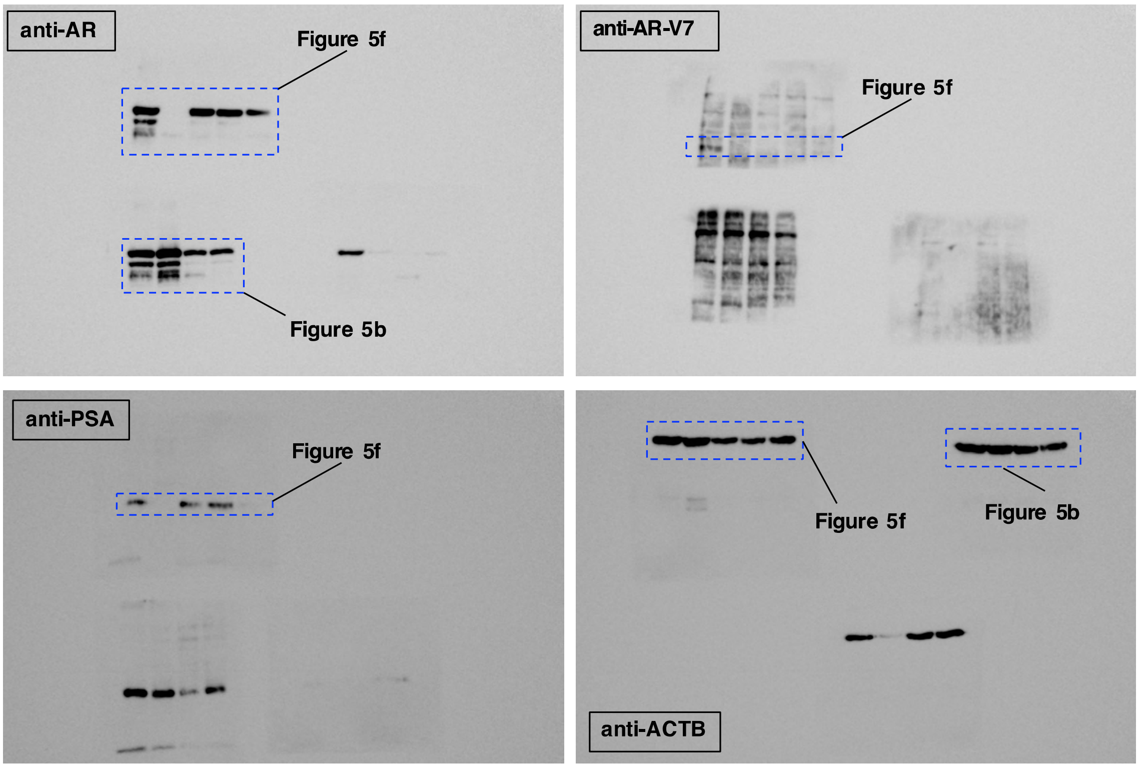

Supplementary Fig. S21. Uncropped gel images for **Figures 5b and 5f**
