# Supplementary material for: Comprehensive genomics in androgen receptor-dependent castration-resistant prostate cancer identifies an adaptation pathway mediated by opioid receptor kappa 1
Source: Commun Biol. 2022 Apr 1;5:299. doi: 10.1038/s42003-022-03227-w (PMC8976065; doi:10.1038/s42003-022-03227-w)
Supplement: Supplementary file 2 — Description of Additional Supplementary Files [file 42003_2022_3227_MOESM2_ESM.pdf]

Description of Additional Supplementary Files

File name: Supplementary Data S1
Description: List of genes called in the ChIP sequence experiments including 3,131 called exclusively for KUCaP2 AD, 1,850 for KUCaP2 CR, and 6,102 shared with both AD and CR tumors.

File name: Supplementary Data S2
Description: List of genes called in the ChIP sequence experiments including 2,938 called exclusively for LNCaP, 717 for AILNCaP, and 1,751 shared with both LNCaP and AILNCaP cells.

File name: Supplementary Data S3
Description: Differentially upregulated genes in KUCaP2 CR tumors compared with KUCaP2 AD tumors in RNA seq (fkpm > 3.0 and fold change > 4.0.

File name: Supplementary Data S4
Description: Differentially downregulated genes in KUCaP2 CR tumors compared with KUCaP2 AD tumors in RNA seq (fkpm > 3.0 and fold change < 0.25).

File name: Supplementary Data S5
Description: List of primers used in the present study.

File name: Supplementary Data S6
Description: List of siRNA oligos used in the present study.

File name: Supplementary Data S7
Description: List of antibodies used in the prenset study.
