# Supplementary material for: Comprehensive genomics in androgen receptor-dependent castration-resistant prostate cancer identifies an adaptation pathway mediated by opioid receptor kappa 1
Source: Commun Biol. 2022 Apr 1;5:299. doi: 10.1038/s42003-022-03227-w (PMC8976065; doi:10.1038/s42003-022-03227-w)
Supplement: Supplementary file 3 — Supplementary Data S1 [file 42003_2022_3227_MOESM3_ESM.pdf]

Supplementary data S1. List of genes called in the ChIP sequence experiments including 3,131 called exclusively for KUCaP2 AD, 1,850 for KUCaP2 CR, and 6,102 shared with both AD and CR tumors

KUCaP2 AD	KUCaP2 CR	Common
AADAC	A2ML1	A1CF
ABCC1	A2MP1	A2M
ABCG2	AAGAB	AAA1
ABHD11	AARSD1	AADACL2
ABHD12	AASDH	AADAT
ABHD13	ABCA8	AAK1
ABLIM3	ABCC3	AARS2
ABRA	ABHD10	AASS
ACE3	ABHD5	AATF
ACP1	ABHD6	ABCA1
ACP6	ABI1P1	ABCA10
ACRC	ACAA1	ABCA12
ACSBG1	ACAD10	ABCA13
ACSF2	ACCSL	ABCA5
ACSL1	ACE2	ABCB1
ACSM1	ACO1	ABCB10
ACSM3	ACOX1	ABCB11
ACTBP11	ACOX2	ABCB4
ACTBP8	ACTR2	ABCB5
ACTC1	ACTR3P3	ABCC10
ACTG1P1	ACTR6	ABCC13
ACVR2B	ADAM30	ABCC2
ADAD1	ADAMTS19	ABCC4
ADAL	ADCK3	ABCC5
ADAM32	ADCYAP1	ABCC9
ADAM3A	ADD1	ABCD2
ADAMTSL4	ADIPOQ	ABCF1
ADAP2	AGBL2	ABHD12B
ADCY3	AGGF1	ABHD2
ADCY5	AGPAT3	ABHD3
ADCYAP1R1	AGPAT9	ABI1
ADH1B	AGR3	ABI2
ADH1C	AICDA	ABI3BP
ADH5P4	AIDA	ABL1
ADH7	AIMP1	ABL2
ADO	AK4P3	ABO
ADORA2B	AKAP11	ABP1
ADRA2A	AKAP3	ABTB2
ADRBK2	AKAP8L	ACAA2
AFM	AKR1B15	ACACA

AGPAT5	AKR1C4	ACAD11
AGPHD1	AKR1E2	ACADL
AHNAK	ALB	ACADSB
AK1	ALG10B	ACAP2
AK2P2	ALLC	ACAT1
AK4P5	ALOX12	ACBD3
AK4P6	ALOX15B	ACBD5
AKR1B1	ALX1	ACBD6
AKR1B10	ANAPC16	ACCN1
AKR1B1P1	ANKDD1B	ACER2
AKR1C2	ANKLE2	ACER3
ALDH1A2	ANKRD13D	ACHE
ALDH1B1	ANKRD30BP	ACIN1
ALDH7A1P3	ANKRD34A	ACMSD
ALKBH1	ANKRD40	ACN9
ALKBH3	ANKRD52	ACOT12
ALPK3	ANKUB1	ACOT13
AMELY	AP1AR	ACOXL
AMICA1	AP1S1	ACPL2
AMIGO2	APEX1	ACPP
AMTN	APOF	ACSL3
ANAPC10	ARF1P1	ACSM4
ANAPC4	ARF4P1	ACSS3
ANAPC5	ARG1	ACTG1P9
ANAPC7	ARHGAP17	ACTN2
ANGEL2	ARHGAP28	ACTR3
ANGPTL4	ARHGAP35	ACTR3B
ANGPTL5	ARHGDIB	ACTR3C
ANK1	ARHGEF35	ACTR3P1
ANKDD1A	ARID3B	ACVR1
ANKRA2	ARMC1	ACVR1C
ANKRD1	ARMCX3-AS	ACVR2A
ANKRD10	ARMCX5	ACYP2
ANKRD20A4	ARMS2	ADAM10
ANKRD20A6	ARV1	ADAM12
ANKRD20A7	ASB4	ADAM17
ANKRD26P1	ASCL1	ADAM18
ANKRD30BP	ASPM	ADAM2
ANKRD31	ATF1P1	ADAM22
ANKRD34B	ATG12	ADAM23
ANKRD6	ATG16L1	ADAM5P
ANO5	ATP2B2	ADAM7
ANP32AP1	ATP5A1P2	ADAM9
ANP32BP2	ATP5C1P1	ADAMTS12
ANP32D	ATP5F1P4	ADAMTS16

ANPEP	ATP5G2P1	ADAMTS17
ANTXRL	ATP5S	ADAMTS18
ANXA4	ATP6V0A2	ADAMTS20
ANXA7	ATP8A2P1	ADAMTS3
APCDD1	ATPAF1	ADAMTS6
APH1A	ATXN7L3B	ADAMTS9
APIP	AZI2	ADAMTS9-AS2
APLNR	BAMBI	ADAMTSL1
APOA5	BAZ2A	ADAMTSL3
APOBEC1	BCCIP	ADAP1
APOO	BCL2L11	ADAR
APPBP2	BCL2L2-PAB	ADARB1
AQP9	BCOR	ADARB2
AQPEP	BCORP1	ADAT2
ARGLU1	BDH2	ADCY1
ARHGAP16P	BECN1	ADCY2
ARHGAP29	BEND6	ADCY8
ARHGAP40	BHLHE40	ADD3
ARHGEF5	BIVM-ERCC5	ADH5
ARHGEF7	BMP3	ADHFE1
ARL1	BNIPL	ADI1
ARL14	BRAP	ADIPOR1
ARL15	BRI3P2	ADK
ARL2BPP7	BROX	ADNP
ARL5B	BTF3L4	ADPRH
ARL6	BTG1	ADRB2
ARL6IP5	BUTR1	ADSS
ARMC9	C10orf113	AEBP2
ARPC3	C10orf140	AFF1
ARPC3P2	C10orf67	AFF3
ARPC3P4	C10orf92	AFF4
ARPC4	C11orf30	AFG3L2
ARPM1	C11orf71	AFTPH
ARRB1	C12orf23	AGAP1
ARSA	C12orf24	AGAP7
ARSG	C12orf41	AGAP9
ARSK	C12orf65	AGBL1
ART2P	C12orf66	AGBL3
AS3MT	C12orf69	AGBL4
ASAH2B	C12orf74	AGFG1
ASB17	C13orf44-AS	AGFG2
ASB5	C14orf102	AGGF1P3
ASIP	C14orf166B	AGK
ASNS	C14orf167	AGMO
ASPA	C14orf39	AGPS

ASS1P11	C15orf38	AGR2
ASS1P13	C18orf42	AGTPBP1
ASS1P7	C18orf62	AGTR1
ATF7IP	C18orf8	AGXT2
ATOH7	C1orf185	AHCTF1
ATP10A	C1orf198	AHCTF1P1
ATP12A	C1orf220	AHCYL2
ATP1A1OS	C1orf227	AHI1
ATP1B3	C1orf43	AHR
ATP5A1P5	C1orf49	AHRR
ATP5A1P8	C1orf56	AIG1
ATP5F1P2	C1QTNF6	AIM1
ATP5G1P4	C1QTNF9B	AK3
ATP5G3	C1RL	AK4P2
ATP7B	C1S	AK5
ATP8B2	C20orf26	AK7
ATRIP	C21orf88	AK8
ATRNL1	C2orf27A	AKAP10
ATXN10	C2orf63	AKAP12
AURKA	C2orf73	AKAP13
AURKB	C2orf80	AKAP2
AVPI1	C2orf88	AKAP6
AVPR1A	C3orf33	AKAP7
AZFP	C3orf49	AKAP9
AZGP1	C4BPA	AKD1
B2M	C5orf24	AKIRIN2
B3GALT1	C5orf30	AKNA
B3GAT3	C5orf32	AKR1B1P3
B3GNT1	C6orf123	AKR1C1
bA255A11.4	C6orf204	AKR1CL1
BACE1	C6orf226	AKR1D1
BACE2	C6orf94	AKT3
BAG1	C7orf16	ALCAM
BAHCC1	C7orf40	ALDH18A1
BAHD1	C7orf69	ALDH1A1
BAK1P2	C7orf70	ALDH1A3
BARD1	C8G	ALDH1L1
BARX1	C8orf84	ALDH1L2
BATF3	C9orf116	ALDH3A2
BBIP1	C9orf123	ALDH5A1
BBS4	C9orf146	ALG2
BBS7	C9orf150	ALG8
BCAP29	C9orf156	ALG9
BCAS2P1	C9orf40	ALK
BCAS4	C9orf47	ALKBH8

BCL2L1	C9orf57	ALMS1
BCL2L10	CAB39	ALOX15P2
BCL6	CABIN1	ALOX5
BCL7B	CABYR	ALPK2
BEND2	CABYRP1	ALS2
BEND7	CALB1	ALS2CR11
BEX2	CALCA	ALS2CR12
BFAR	CALM2P3	ALS2CR8
BHLHE22	CAPN9	AMBP
BLVRB	CAPS2	AMBRA1
BMI1	CASK	AMD1
BMP5	CASKP1	AMDHD1
BNIP1	CAV2	AMN1
BOC	CAV3	AMOTL1
BRCA2	CBLN2	AMPH
BRI3	CCBP2	ANAPC1
BRP44L	CCDC115	ANGPT1
BSN	CCDC144NL	ANK2
BTBD16	CCDC160	ANK3
BTC	CCDC164	ANKAR
BTF3P7	CCDC165P1	ANKH
BTF3P9	CCDC169-S(	ANKHD1
BTG3	CCDC39	ANKHD1-EIF4EBP3
BTNL3	CCDC89	ANKIB1
BVES	CCL18	ANKMY2
C10orf103	CCL21	ANKRD12
C10orf108	CCL28	ANKRD13A
C10orf128	CCNB1IP1	ANKRD13C
C10orf131	CCNDBP1	ANKRD16
C10orf53	CCNG1	ANKRD17
C10orf58	CCNG2	ANKRD18A
C10orf88	CCNH	ANKRD19P
C11orf24	CCNT2	ANKRD20A5P
C11orf63	CCR2	ANKRD20A8P
C11orf82	CCR5	ANKRD26
C12orf68	CCT4	ANKRD28
C12orf70	CCT5	ANKRD29
C12orf73	CCT6A	ANKRD30A
C13orf26	CCT8	ANKRD30B
C14orf101	CD27	ANKRD30BL
C14orf126	CD34	ANKRD32
C14orf135	CD3D	ANKRD33B
C14orf166	CD69	ANKRD35
C14orf183	CDC16	ANKRD36
C14orf19	CDC20B	ANKRD36B

C14orf23	CDC25A	ANKRD36BP2
C14orf38	CDK5PS	ANKRD42
C15orf23	CDK8	ANKRD44
C15orf40	CDKL5	ANKRD45
C15orf42	CDKN2C	ANKRD46
C15orf44	CDR2	ANKRD57
C15orf48	CDS1	ANKRD62
C17orf104	CDV3P1	ANKRD7
C17orf39	CEBPD	ANKS1A
C17orf42	CECR7	ANKS1B
C17orf75	CELF3	ANKS4B
C17orf76	CENPA	ANKS6
C17orf76-AS	CEP55	ANLN
C17orf78	CEP95	ANO10
C17orf81	CERS5	ANO2
C18orf12	CETN1	ANO3
C1orf15-NBL	CHCHD2P10	ANO4
C1orf151	CHCHD2P9	ANO6
C1orf65	CHFR	ANP32A
C1orf95	CHI3L1	ANP32B
C1QL3	CHIC1	ANP32E
C1QTNF3	CHIC2	ANTXR1
C1QTNF5	CHORDC2P	ANTXR2
C20orf194	CICP4	ANUBL1
C20orf72	CIZ1	ANXA10
C21orf37	CLDN1	ANXA13
C21orf91	CLDN16	ANXA2
C2orf27B	CLDN8	ANXA3
C2orf48	CLEC12B	AOAH
C2orf50	CLEC1A	AOX1
C2orf65	CLEC2D	AOX2P
C2orf71	CLEC4E	AP1S3
C2orf72	CLEC7A	AP2B1
C2orf84	CLEC9A	AP3B1
C3orf17	CLIC2	AP3M1
C3orf24	CLSPN	AP3S1
C3orf35	CMPK2	AP3S2
C3orf56	CNGA3	AP4E1
C3orf58	CNIH	AP4S1
C3orf64	CNKSR3	APAF1
C3orf70	CNPY1	APBA1
C3orf78	CNRIP1	APBA2
C4orf21	CNTD1	APBB1IP
C4orf27	COL3A1	APBB2
C4orf34	COL4A5	APC

C4orf43	COL6A3	APH1B
C4orf51	COPZ1	API5
C5orf13	COX15	APLF
C5orf27	COX6CP2	APLP2
C5orf39	COX7B	APP
C5orf41	CPB2	APPL1
C5orf46	CPNE3	APPL2
C5orf51	CPO	APTX
C6orf115	CROCCP2	AQP11
C6orf136	CROT	AQR
C6orf145	CRYM	AR
C6orf154	CS	ARAP2
C6orf165	CSRP1	ARCN1
C6orf186	CTH	ARF1
C6orf191	CTLA4	ARF1P3
C6orf201	CTSL1P6	ARF4
C6orf222	CTXN2	ARF5
C6orf58	CXorf59	ARF6
C7orf23	CYBB	ARFGEF1
C7orf28B	CYC1	ARFIP1
C7orf30	CYCSP51	ARHGAP10
C7orf36	CYLD	ARHGAP12
C7orf55	CYP2J2	ARHGAP15
C7orf57	CYP7A1	ARHGAP18
C7orf71	DAB2	ARHGAP19
C8orf22	DACT2	ARHGAP19-SLIT1
C8orf37	DBX2	ARHGAP20
C8orf38	DCAF7	ARHGAP21
C8orf4	DCD	ARHGAP22
C8orf59	DCLRE1C	ARHGAP24
C8orf75	DCTN6	ARHGAP26
C9orf103	DDHD2	ARHGAP31
C9orf117	DDX23	ARHGAP32
C9orf131	DDX39BP2	ARHGAP39
C9orf142	DDX3YP3	ARHGAP42
C9orf147	DEFB127	ARHGAP44
C9orf153	DEPDC4	ARHGAP5
C9orf50	DEPDC7	ARHGAP6
C9orf51	DERL1	ARHGEF12
C9orf71	DHCR7	ARHGEF26
C9orf72	DHRS13	ARHGEF26-AS1
C9orf95	DHRS4L2	ARHGEF3
CA14	DIRC1	ARHGEF37
CAD	DLX5	ARHGEF38
CALM1P2	DMC1	ARHGEF4

CALM2	DMD	ARID1A
CALM2P1	DMRT3	ARID1B
CALML4	DNAJA2	ARID2
CAMK1	DNAJC7	ARID4A
CAMTA1	DNAJC9	ARID4B
CAP1P2	DND1P1	ARID5B
CAP2	DNM3	ARIH1
CAPN1	DPY19L2P1	ARIH2
CAPSL	DSCAML1	ARL13B
CAPZB	DSCR3	ARL5A
CARD11	DSG3	ARL8B
CARD16	DTD1	ARMC2
CARTPT	DUPD1	ARMC3
CASC5	DUSP19	ARMC4
CASP10	DUSP5P	ARMC8
CBX1P3	DYDC2	ARMCX5-GPRASP2
CBX1P5	DYRK4	ARNT
CBX3P5	E2F3	ARNT2
CBY3	E2F6	ARNTL2
CC2D2A	E2F7	ARPC1A
CCDC104	EBF1	ARPP19
CCDC107	ECE1	ARPP21
CCDC109B	EDDM3B	ARRDC3
CCDC110	EEF1A1P33	ARSJ
CCDC111	EEF1A1P8	ART3
CCDC12	EEF1A1P9	ART4
CCDC13	EFHC1	ASAP1
CCDC144B	EFHD1	ASAP2
CCDC149	EFTUD2	ASB13
CCDC158	EGF	ASB14
CCDC162P	EHD3	ASB15
CCDC29	EIF2AK3	ASB3
CCDC47	EIF2B3	ASB7
CCDC54	EIF2S2	ASCC1
CCDC57	EIF2S2P6	ASCC3
CCDC58	EIF4A1P13	ASH1L
CCDC80	EIF4BP3	ASNSD1
CCDC84	ENC1	ASPH
CCDC85A	ENTPD2	ASPN
CCDC92	ENTPD8	ASRGL1
CCL1	ENY2	ASS1P12
CCL26	EP400	ASS1P3
CCNB2	EPCAM	ASTN1
CCNC	EPHA4	ASTN2
CCND2	EPPK1	ASXL1

CCNYL1	ERBB2	ASXL2
CCP110	ERCC5	ASXL3
CCR3	ERCC8	ASZ1
CCR8	ERN1	ATAD2
CCRL2	ERP27	ATAD2B
CCT2	ERP29	ATAD5
CCT6P1	ERRFI1	ATAT1
CCT6P2	ESF1	ATF2
CCT6P4	ESYT1	ATF3
CCT6P5	ETFDH	ATF7
CCT7P2	EWSR1	ATG10
CD101	F10	ATG13
CD109	FABP4	ATG2B
CD160	FAM104A	ATG3
CD163	FAM108B1	ATG4C
CD2	FAM111A	ATG5
CD200R1L	FAM113B	ATG7
CD302	FAM133A	ATL1
CD38	FAM133B	ATL2
CD58	FAM134C	ATL3
CD59	FAM138C	ATM
CD82	FAM138F	ATN1
CD99	FAM157B	ATP10B
CDADC1	FAM166B	ATP10D
CDC123	FAM179A	ATP11B
CDC23	FAM186A	ATP13A3
CDC27P1	FAM200A	ATP13A4
CDC37L1	FAM200B	ATP13A5
CDC40	FAM205B	ATP2A2
CDC42BPB	FAM24B	ATP2B1
CDC42P2	FAM27L	ATP2B4
CDH23	FAM43B	ATP2C1
CDH4	FAM49A	ATP5A1
CDK1	FAM54A	ATP5B
CDK2AP2	FAM58BP	ATP5F1P6
CDK2AP2P1	FAM71D	ATP5LP5
CDKL3	FAM82B	ATP6V0A4
CDKN1B	FAM83A	ATP6V0D2
CDKN2A	FAM83B	ATP6V0E1
CDKN2AIPNI	FAM84B	ATP6V0E2
CDKN2B	FAM8A2P	ATP6V1A
CDO1	FAM91A1	ATP6V1C1
CDV3	FARSB	ATP6V1C2
CDY10P	FBXO15	ATP6V1G1
CDY11P	FBXO45	ATP6V1G3

CDY3P	FBXO8	ATP6V1H
CECR5	FBXO9	ATP7A
CELF6	FBXW12	ATP8A1
CELP	FBXW2	ATP8A2
CENPJ	FCGR1C	ATP8B1
CENPL	FDPS	ATP8B4
CENPV	FEM1AP2	ATP8B5P
CEP135	FEM1B	ATP9A
CEP164	FETUB	ATP9B
CEP170P1	FGB	ATPBD4
CEP76	FGF2	ATR
CFHR2	FGF7P1	ATRN
CFHR4	FKBP1AP4	ATRX
CFHR5	FLJ12334	ATXN1
CFI	FLJ20712	ATXN2
CFL2	FLJ37453	ATXN7
CHAF1A	FLJ39061	ATXN7L1
CHCHD5	FLJ40288	ATXN8OS
CHDH	FLJ40852	AUH
CHRM5	FLJ41649	AUTS2
CHRNA3	FLJ42280	AVEN
CHRNA5	FLJ42351	AVL9
CHRNB4	FLJ42969	AVPR1B
CHST11	FLJ44054	AXDND1
CHST2	FNDC3CP	AZIN1
CHST3	FOXB2	B3GALNT1
CHTOP	FOXG1	B3GALNT2
CHURC1-FN	FOXJ2	B3GNT6
CIB4	FOXL2	B4GALT1
CICP14	FRA10AC1	B4GALT4
CISD2	FSHB	B4GALT5
CITED2	GABRA4	B4GALT6
CKAP2L	GAL3ST2	B7H6
CKS1B	GAPDHP30	BAALC
CLDN10	GAPDHP58	BAAT
CLDN11	GBA3	BAATP1
CLDN12	GBX1	BACH1
CLDN14	GCA	BACH2
CLDN25	GCK	BAGE2
CLEC12A	GDAP1	BAI3
CLEC2L	GDF6	BAIAP2L1
CLEC6A	GDNF	BANK1
CLGN	GFOD1	BARX2
CLINT1	GFPT1	BASP1
CLIP4	GGT1	BATF

CLLU1OS	GIGYF2	BAZ1A
CLNK	GIMAP1-GIM.	BAZ1B
CLNS1A	GIMAP5	BAZ2B
CLRN1	GIMAP8	BBOX1
CMAS	GKN1	BBS5
CMPK1	GLO1	BBS9
CMYA5	GLRX3	BBX
CNDP2	GLTP	BCAS1
CNGA1	GLYATL1P2	BCAS3
CNIH3	GLYATL1P3	BCAT1
CNN2P9	GML	BCHE
CNNM1	GMNN	BCKDHB
CNNM3	GNA13	BCL11A
CNNM4	GOLT1B	BCL2
CNTFR	GPR125	BCL9
CNTNAP3	GPR85	BCLAF1
COCH	GPX8	BCO2
COG6	GRAMD1B	BDAG1
COL12A1	GREM1	BDH1
COL15A1	GRIK1-AS2	BDNF
COL20A1	GRPEL2P3	BDNF-AS1
COL23A1	GSTA4	BDP1
COL4A2	GTF2H1	BEST3
COL4A4	GTF3C5	BET3L
COL5A1	GUF1	BEX4
COL9A1	GYG2P2	BFSP2
COMMD1	H2AFV	BHLHE41
COMMD3-BM	H2AFZP2	BICC1
COMMD5	HADHAP2	BICD1
COMMD8	HAO2	BICD2
COMMD9	HAR1B	BIN1
COPS3	HAS2-AS1	BIRC2
COPS7A	HAUS4	BIRC3
COQ5	HCG4P4	BIRC6
COX18	HCG9	BLM
COX19	HCRTR2	BLMH
COX5B	HDLBP	BLNK
COX5BP4	HDX	BLVRA
COX6B1P5	HEATR2	BMP2K
COX7B2	HECA	BMPER
COX7CP1	HEXIM2	BMPR1B
CPA4	HFE2	BMPR2
CPA5	HHLA2	BMS1P4
CPN1	HIN1L	BMS1P6
CPNE1	HINT3	BNC1

CPSF7	HIST1H2BPS	BNC2
CPXM2	HIST1H3D	BNIP2
CREBZF	HIST1H3E	BNIP3P4
CRHR1	HIST1H4C	BOLA1
CRISP3	HIST3H2BB	BOLL
CRNN	HLA-DMA	BPGM
CROCC	HLA-E	BPNT1
CROCCP3	HMGB1P15	BPTF
CRTAC1	HMGB1P27	BRAF
CRTAM	HMGB1P46	BRCA1
CRY2	HMGN1P11	BRD2
CRYBA1	HMGN2P13	BRD9
CRYGFP	HNF1A	BRE
CRYZL1	HNF1B	BRIP1
CSN2	HNRNPA1P8	BRMS1L
CSNK1A1P1	HNRPUP	BRWD1
CSNK2A1	HOMER1	BSPRY
CSRNP1	HOXB13	BTAF1
CTAGE1	HOXB13-AS1	BTBD1
CTAGE13P	HOXC10	BTBD11
CTBP2P1	HOXC13	BTBD9
CTCFL	HRASLS5	BTD
CTGLF11P	HSD17B7P1	BTLA
CTNNB1	HSD3B2	BTNL8
CTSL1P4	HSD3BP4	BUB1B
CTSL3	HSD3BP5	BUD13
CTXN3	HSF2BP	BZW1
CUZD1	HSP90AA1	BZW2
CWC22	HSP90AA5P	C10orf107
CWH43	HSP90B1	C10orf11
CXCL13	HSPA5	C10orf112
CYB5A	HSPE1	C10orf120
CYB5AP2	HTN3	C10orf122
CYB5R1	HTR1F	C10orf137
CYCS	HTR3C	C10orf28
CYCSP16	HUWE1	C10orf32-AS3MT
CYCSP20	HVCN1	C10orf40
CYCSP39	HYAL1	C10orf47
CYCSP42	IFNA12P	C10orf68
CYCSP7	IFNA2	C10orf90
CYFIP1	IFNA4	C11orf2
CYFIP2	IFNG	C11orf41
CYorf15B	IFNW1	C11orf44
CYP11A1	IGHV1OR15-	C11orf49
CYP11B2	IGLJCOR18	C11orf53

CYP19A1	IKZF4	C11orf54
CYP1B1-AS1	IL10	C11orf58
CYP24A1	IL17RA	C11orf61
CYP2B7P1	IL18R1	C11orf65
CYP2E1	IL1RN	C11orf67
CYP3A7	IL22	C11orf70
CYP4V2	IL31RA	C11orf73
CYP4X1	IL36B	C11orf74
CYP4Z1	IL36G	C11orf75
CYP51A1	ILF3	C11orf80
CYTH1	IMPAD1	C11orf93
CYYR1	INCENP	C12orf11
DAAM1	INSIG2	C12orf26
DAD1	INTS12	C12orf34
DAG1	IQCD	C12orf35
DAOA-AS1	IRAK4	C12orf4
DARS	ISCA1P2	C12orf40
DAZAP2	ISCU	C12orf42
DBF4	ISY1	C12orf45
DBF4B	ISY1-RAB43	C12orf48
DBIP2	ITGA5	C12orf49
DCAF13	ITM2A	C12orf5
DCAF6	ITPKB-IT1	C12orf50
DCHS2	ITPRIP	C12orf51
DCLK3	IVL	C12orf55
DCN	JAGN1	C12orf56
DCP2	JAK1	C12orf60
DCPS	JTB	C12orf63
DCT	JUN	C12orf75
DDB1	KATNAL1	C14orf182
DDIT4	KCNA1	C14orf28
DDX17	KCNG2	C14orf37
DDX18	KCNG3	C14orf56
DDX21	KCNS3	C14orf93
DDX39BP1	KCTD14	C15orf24
DEFB126	KCTD7	C15orf27
DEFB132	KDM1B	C15orf29
DEPDC1	KDM3B	C15orf33
DEPDC1B	KDM6A	C15orf38-AP3S2
DHCR24	KDM6B	C15orf41
DHFRL1	KIAA0020	C15orf43
DHRS7	KIAA0196	C15orf60
DHX29	KIAA0226	C16orf52
DIP2A	KIAA0922	C16orf61
DIS3L	KIAA1009	C17orf57

DKFZP434F1	KIAA1614	C17orf63
DLEC1	KIAA1737	C18orf1
DLEU1	KIAA1919	C18orf19
DLX2	KIAA1984	C18orf21
DLX3	KIF18B	C18orf25
DMP1	KIF2A	C18orf34
DMTF1	KIF4CP	C18orf45
DNAAF2	KIF5A	C18orf63
DNAJA4	KIF9	C19orf2
DNAJB13	KIT	C19orf48
DNAJC16	KLC4	C1GALT1
DNAJC25	KLF10	C1orf100
DNAJC27-AS	KLF14	C1orf101
DNAJC3	KLF4	C1orf105
DNAJC8P1	KLHL23	C1orf106
DNAL1	KLHL25	C1orf115
DNER	KLHL38	C1orf116
DNM1P5	KLK15	C1orf124
DOCK11	KLK2	C1orf131
DOCK11P1	KLK3	C1orf150
DOCK2	KLRG2	C1orf168
DOCK5	KRBA1	C1orf186
DOK5	KRR1	C1orf21
DPM3	KRT16P4	C1orf229
DPP7	KRT18P22	C1orf27
DPRX	KRT18P24	C1orf31
DPY30	KRT18P30	C1orf53
DPYSL3	KRT18P32	C1orf55
DSC2	KRT18P55	C1orf87
DSC3	KRT24	C1orf9
DUOXA1	KRT25	C1orf96
DUSP14	KRTAP13-4	C1orf98
DUSP22	KRTAP19-9P	C1QTNF3-AMACR
DUSP5	KRTAP2-4	C20orf108
DUSP6	KRTAP2-5P	C20orf177
DVL2	KRTAP4-1	C20orf197
DYDC1	KRTAP4-16P	C20orf94
DYNC2LI1	KRTAP9-1	C21orf7
DYNLL1	LARS	C22orf26
DYRK2	LATS1	C2CD3
DYSF	LBR	C2orf29
DYX1C1	LCE1A	C2orf3
E2F8	LCE1C	C2orf43
ECD	LCE1D	C2orf55
ECE2	LCE2A	C2orf67

ECHDC3	LCE2B	C2orf76
ECM2	LCE4A	C2orf77
EDA	LCN1	C3orf15
EDF1	LCN1P2	C3orf19
EEF1A1	LDHB	C3orf20
EEF1A1P14	LGMNP1	C3orf21
EEF1A1P25	LIMK1	C3orf23
EEF1A1P36	LINC00108	C3orf26
EEF1A1P6	LINC00162	C3orf38
EEF1B2P2	LINC00238	C3orf52
EEF1E1	LINC00251	C3orf55
EEF1E1-MUT	LINC00264	C3orf67
EFCAB10	LINC00308	C3orf77
EFCAB7	LINC00326	C4orf19
EFEMP1	LINC00371	C4orf22
EFNA1	LINC00410	C4orf32
EGLN3	LINC00466	C4orf37
EGR2	LINC00485	C4orf41
EIF1B	LMAN2L	C4orf45
EIF2A	LMCD1	C5
EIF2B5	LMF1	C5orf17
EIF2D	LNPEP	C5orf22
EIF3EP2	LOC1001254	C5orf28
EIF3FP1	LOC1001280	C5orf34
EIF4A1P1	LOC1001280	C5orf38
EIF4A1P12	LOC1001280	C5orf42
EIF4A1P6	LOC1001281	C5orf44
EIF4BP4	LOC1001281	C6
ELF2	LOC1001282	C6orf1
ELF5	LOC1001284	C6orf10
ELOVL4	LOC1001284	C6orf103
ELOVL5	LOC1001285	C6orf105
EMB	LOC1001287	C6orf106
EML1	LOC1001287	C6orf108
ENG	LOC1001288	C6orf138
ENHO	LOC1001289	C6orf141
ENOX2	LOC1001290	C6orf168
ENPP3	LOC1001290	C6orf170
ENTPD6	LOC1001292	C6orf174
EOMES	LOC1001292	C6orf183
EP400NL	LOC1001292	C6orf218
EPAS1	LOC1001295	C6orf223
EPDR1	LOC1001295	C6orf228
EPHB4	LOC1001295	C6orf52
EPN2	LOC1001297	C7

EPS15P1	LOC1001297	C7orf10
EPT1	LOC1001297	C7orf31
ERCC3	LOC1001299	C7orf33
EREG	LOC1001299	C7orf41
ERV3-1	LOC1001299	C7orf42
ESYT3	LOC1001300	C7orf44
ETAA1	LOC1001300	C7orf46
ETNK1	LOC1001300	C7orf49
ETV5	LOC1001300	C7orf50
EVX1	LOC1001301	C7orf58
EXOC5	LOC1001301	C7orf60
EXOC8	LOC1001301	C7orf63
EXOG	LOC1001301	C7orf68
EXOSC3	LOC1001302	C7orf72
F13B	LOC1001302	C7orf74
F2RL1	LOC1001302	C8A
F5	LOC1001302	C8orf34
FABP5	LOC1001303	C8orf44
FABP5P5	LOC1001305	C8orf44-SGK3
FABP9	LOC1001305	C8orf45
FAH	LOC1001307	C8orf46
FAHD2B	LOC1001308	C8orf47
FAM108C1	LOC1001309	C8orf76
FAM114A1	LOC1001310	C8orf83
FAM115C	LOC1001310	C8orf85
FAM115D	LOC1001312	C9
FAM120B	LOC1001312	C9orf102
FAM123C	LOC1001312	C9orf11
FAM131B	LOC1001312	C9orf129
FAM149A	LOC1001313	C9orf135
FAM150A	LOC1001313	C9orf148
FAM151B	LOC1001314	C9orf152
FAM154B	LOC1001314	C9orf170
FAM169B	LOC1001314	C9orf171
FAM171A1	LOC1001314	C9orf174
FAM172BP	LOC1001315	C9orf21
FAM173B	LOC1001315	C9orf25
FAM174A	LOC1001315	C9orf29
FAM177A2	LOC1001316	C9orf3
FAM180A	LOC1001316	C9orf30
FAM183B	LOC1001317	C9orf30-TMEFF1
FAM184A	LOC1001320	C9orf4
FAM184B	LOC1001321	C9orf41
FAM189A2	LOC1001321	C9orf46
FAM193A	LOC1001322	C9orf5

FAM203A	LOC1001323	C9orf53
FAM205A	LOC1001323	C9orf64
FAM205CP	LOC1001323	C9orf68
FAM24A	LOC1001327	C9orf80
FAM27B	LOC1001329	C9orf82
FAM3B	LOC1001333	C9orf84
FAM3C	LOC1001345	C9orf85
FAM47E	LOC1001445	C9orf86
FAM55A	LOC1002717	C9orf89
FAM65B	LOC1002867	C9orf91
FAM71F2	LOC1002868	C9orf93
FAM73B	LOC1002869	CA1
FAM75B	LOC1002869	CA10
FAM75D1	LOC1002870	CA12
FAM81B	LOC1002871	CA13
FAM82A2	LOC1002871	CA8
FAM84A	LOC1002872	CAB39L
FAM87B	LOC1002874	CABLES1
FAM8A3P	LOC1002874	CACHD1
FANCI	LOC1002875	CACNA1B
FARP1	LOC1002878	CACNA1D
FARP2	LOC1002878	CACNA1E
FASTKD2	LOC1002878	CACNA2D1
FAUP1	LOC1002879	CACNA2D3
FAUP2	LOC1002884	CACNB2
FBLN1	LOC1002885	CACNB4
FBXL18	LOC1002885	CADM1
FBXL3	LOC1002885	CADM2
FBXL5	LOC1002886	CADPS
FBXO25	LOC1002888	CADPS2
FBXO4	LOC1002891	CALCOCO2
FBXO41	LOC1002891	CALCR
FBXO43	LOC1002891	CALCRL
FBXO5	LOC1002892	CALD1
FBXW10	LOC1002892	CALML3
FCGBP	LOC1002894	CALML5
FCN1	LOC1002895	CALN1
FCN2	LOC1002924	CALU
FDXACB1	LOC1002937	CAMK1D
FER1L5	LOC1002939	CAMK1G
FERD3L	LOC1004188	CAMK2B
FERMT1	LOC1004188	CAMK2D
FEZ1	LOC1004188	CAMK4
FFAR2	LOC1004189	CAMKMT
FGF10	LOC1004189	CAMSAP1

FGF13	LOC1004195	CAMSAP2
FGF6	LOC1004196	CAND1
FGF7	LOC1004197	CANX
FGFR1OP	LOC1004197	CAPN11
FGFR3P2	LOC1004197	CAPN13
FGL2	LOC1004198	CAPN14
FH	LOC1004198	CAPN2
FHL2	LOC1004199	CAPN5
FHL5	LOC1004199	CAPN7
FIBIN	LOC1004199	CAPN8
FIGNL1	LOC1004200	CAPRIN1
FKBP14	LOC1004201	CAPRIN2
FKBP6	LOC1004202	CAPZA2
FLG2	LOC1004202	CARD17
FLI1	LOC1004202	CARM1P1
FLJ10038	LOC1004204	CASC1
FLJ37644	LOC1004205	CASC3
FLJ39653	LOC1004205	CASC4
FLJ43826	LOC1004205	CASD1
FLJ43860	LOC1004205	CASP2
FLJ44881	LOC1004205	CASP4
FLJ45139	LOC1004206	CASP8
FLT1	LOC1004206	CASQ2
FLT3	LOC1004206	CASR
FNBP1L	LOC1004208	CAST
FNTB	LOC1004209	CASZ1
FOLR2	LOC1004209	CAT
FOLR4	LOC1004209	CATSPERB
FOS	LOC1004210	CAV1
FOXO1	LOC1004210	CBFA2T2
FREM3	LOC1004211	CBL
FRG2C	LOC1004213	CBLB
FRMPD2	LOC1004213	CBLL1
FRMPD4	LOC1004214	CBR4
FRZB	LOC1004214	CBWD1
FSCN1	LOC1004214	CBWD3
FSTL4	LOC1004215	CBX3
FTH1	LOC1004215	CBX5
FTMT	LOC1004217	CCAR1
FUBP3	LOC1004221	CCBE1
FUT4	LOC1004223	CCBL1
FXYD4	LOC1004223	CCDC102B
FXYD6	LOC1004224	CCDC11
FXYD6-FXYD	LOC1004226	CCDC122
FYCO1	LOC1004227	CCDC126

FYN	LOC1004629	CCDC127
FYTTD1	LOC1004994	CCDC129
GABARAPL1	LOC1004995	CCDC132
GABRA2	LOC1005054	CCDC136
GABRA6	LOC1005055	CCDC138
GABRB1	LOC1005055	CCDC14
GABRG1	LOC1005056	CCDC141
GAD1	LOC1005056	CCDC146
GAD2	LOC1005056	CCDC147
GALNT1	LOC1005057	CCDC148
GALNT5	LOC1005057	CCDC15
GAN	LOC1005057	CCDC150
GAPDHP23	LOC1005057	CCDC152
GAPDHP39	LOC1005059	CCDC165
GAPDHP63	LOC1005059	CCDC3
GAPDHP70	LOC1005059	CCDC34
GAS5	LOC1005059	CCDC36
GAS7	LOC1005060	CCDC38
GBAS	LOC1005060	CCDC41
GCET2	LOC1005060	CCDC50
GCFC1	LOC1005060	CCDC53
GCH1	LOC1005060	CCDC6
GCLC	LOC1005061	CCDC60
GCM1	LOC1005061	CCDC64
GCNT1P5	LOC1005062	CCDC66
GDPD5	LOC1005062	CCDC67
GEM	LOC1005062	CCDC68
GEMIN2	LOC1005063	CCDC7
GEMIN5	LOC1005063	CCDC73
GEN1	LOC1005063	CCDC81
GFM2	LOC1005063	CCDC82
GFRAL	LOC1005063	CCDC83
GGTA1P	LOC1005064	CCDC88A
GHRHR	LOC1005064	CCDC90B
GIGYF1	LOC1005064	CCDC91
GIN1	LOC1005065	CCDC93
GJC1	LOC1005066	CCL15
GJC3	LOC1005066	CCL19
GJD2	LOC1005066	CCL23
GLCE	LOC1005066	CCM2
GLIPR1	LOC1005067	CCNE2
GLIS1	LOC1005067	CCNT1
GLS2	LOC1005067	CCNY
GLT6D1	LOC1005067	CCPG1
GLTPP1	LOC1005067	CCR7

GLUD1P5	LOC1005068	CCRL1
GLULP4	LOC1005069	CCT6B
GLYAT	LOC1005069	CCT6P3
GLYATL1P4	LOC1005069	CD163L1
GM2AP1	LOC1005069	CD164
GNAL	LOC1005069	CD200R1
GNAQP1	LOC1005070	CD226
GNB5	LOC1005070	CD274
GNG10	LOC1005070	CD28
GNGT1	LOC1005071	CD2AP
GNPAT	LOC1005071	CD36
GNPDA2	LOC1005071	CD3E
GNS	LOC1005072	CD3G
GOLGA2P5	LOC1005072	CD44
GOLGA6L2	LOC1005073	CD46
GOLGA8A	LOC1005073	CD46P1
GOT2P2	LOC1005074	CD47
GPCRLTM7	LOC1005074	CD55
GPER	LOC1005074	CD72
GPR111	LOC1005074	CD80
GPR113	LOC1005075	CD86
GPR133	LOC1005075	CD9
GPR148	LOC1005076	CD96
GPR68	LOC1005076	CD99P1
GPR79	LOC1005098	CDC14A
GPRC6A	LOC1005279	CDC14B
GPRIN2	LOC1005279	CDC14BL
GPX1P2	LOC1005336	CDC27
GRIA3	LOC1005336	CDC42
GRIN1	LOC1005336	CDC42BPA
GRINL1A	LOC1005336	CDC42SE2
GRPR	LOC1005336	CDC5L
GSN	LOC1005336	CDC6
GSPT1	LOC1005336	CDC73
GSTM2P1	LOC1005336	CDCA7L
GSTM5P1	LOC1005337	CDH10
GSTO1	LOC1005337	CDH12
GSTO2	LOC1005337	CDH13
GTF2A2	LOC1005337	CDH17
GTF2H5	LOC1006527	CDH18
GTF2IRD1	LOC1006527	CDH19
GTF2IRD2	LOC1006527	CDH2
GTF3C4	LOC1006527	CDH20
GZMH	LOC1006528	CDH26
H2AFVP1	LOC1006528	CDH6

HAPLN1	LOC1006528	CDH7
HAUS1	LOC1006528	CDH9
HAUS3	LOC1006529	CDHR3
HBZ	LOC1006529	CDK12
HCG4P7	LOC1006529	CDK13
HCLS1	LOC1006529	CDK14
HCP5P6	LOC1006529	CDK15
HDAC4	LOC1006530	CDK17
HDAC5	LOC121296	CDK19
HDC	LOC128136	CDK2AP2P3
HDGFRP3	LOC129560	CDK5RAP2
HDHD2	LOC131055	CDK6
HEATR3	LOC133789	CDK7
HEATR7B1	LOC138412	CDKAL1
HECW1-IT1	LOC138971	CDKL1
HELQ	LOC144383	CDKL2
HEMGN	LOC148709	CDKN2B-AS
HEPACAM2	LOC151658	CDKN3
HEPHL1	LOC153684	CDON
HERC6	LOC153910	CDRT15
HESX1	LOC200149	CDYL
HEXA	LOC201617	CDYL2
HEXB	LOC253039	CELF1
HEY1	LOC283172	CELF2
HHEX	LOC283398	CENPC1
HHIP	LOC283588	CENPE
HHIPL2	LOC285033	CENPF
HIGD1AP10	LOC285232	CENPK
HINFP	LOC285375	CENPO
HINT1	LOC285401	CENPP
HIST1H1E	LOC285456	CENPQ
HIST1H2BF	LOC285634	CEP112
HIST1H4D	LOC286238	CEP120
HIST1H4F	LOC286467	CEP128
HIST2H2AC	LOC338579	CEP152
HIST2H3D	LOC339505	CEP170
HIST2H3PS2	LOC339535	CEP192
HIST2H4A	LOC339843	CEP290
HIVEP1	LOC339966	CEP350
HIVEP3	LOC340073	CEP57
HKDC1	LOC344653	CEP57L1
HMG20A	LOC347097	CEP63
HMGB1P25	LOC353010	CEP70
HMGB1P37	LOC388942	CEP72
HMGB1P43	LOC389465	CEP78

HMGB2	LOC390298	CERKL
HMGB3P15	LOC390586	CERS3
HMGCR	LOC390617	CERS6
HMGCS1	LOC390846	CFH
HMGN1P31	LOC391048	CFHR1
HMOX2	LOC391073	CFHR3
HN1	LOC391092	CFLAR
HNRNPA0	LOC391359	CFLAR-AS1
HNRNPA1L2	LOC391465	CFTR
HNRNPA1P1	LOC391556	CGGBP1
HNRNPD	LOC391562	CGN
HNRNPKP3	LOC391578	CGNL1
HOMER2	LOC391600	CGRRF1
HORMAD1	LOC391741	CHCHD3
HOXA11-AS1	LOC392027	CHCHD6
HOXA4	LOC392232	CHD1
HOXD13	LOC392268	CHD1L
HPS1	LOC392352	CHD2
HPVC1	LOC392368	CHD6
HRASLS	LOC400026	CHD7
HRH4	LOC400061	CHD8
HRSP12	LOC400769	CHEK1
HS3ST3A1	LOC400927	CHIT1
HSD11B1	LOC401180	CHKA
HSD17B13	LOC401321	CHL1
HSD3BP2	LOC401767	CHMP2B
HSPA4L	LOC402216	CHMP3
HSPD1P17	LOC402360	CHMP4C
HSPD1P4	LOC440917	CHMP5
HSPD1P9	LOC440973	CHN1
HTR1A	LOC441009	CHN2
HTR3D	LOC441698	CHP
HTR5BP	LOC442060	CHPT1
HUS1	LOC442097	CHRM1
HYLS1	LOC442098	CHRM2
IAPP	LOC442132	CHRM3
IBSP	LOC442292	CHRNA7
ICK	LOC442459	CHST10
IDO1	LOC442668	CHST9
IDO2	LOC503540	CHST9-AS1
IFIH1	LOC574080	CHSY1
IFNA16	LOC641746	CHSY3
IFNA20P	LOC642361	CHUK
IFNA21	LOC642554	CIR1
IFNA5	LOC642969	CIT

IFNAR1	LOC643037	CKAP5
IFNGR1	LOC643058	CKS2
IFNNP1	LOC643072	CLASP1
IFT172	LOC643085	CLASP2
IGFBP2	LOC643401	CLCN1
IGHMBP2	LOC643623	CLCN3
IGHV1OR15-	LOC643733	CLDN18
IGJP1	LOC643916	CLDN4
IGKV1OR2-1	LOC644006	CLEC2B
IGSF10	LOC644060	CLEC4A
IL18RAP	LOC644160	CLEC5A
IL1B	LOC644233	CLECL1
IL1F10	LOC644265	CLIC5
IL1R1	LOC644387	CLIP1
IL1R2	LOC644659	CLIP2
IL1RL1	LOC644681	CLK1
IL2	LOC644684	CLK2P
IL20	LOC644776	CLMP
IL36A	LOC644877	CLN6
IL6	LOC645266	CLOCK
IMPA2	LOC645297	CLPB
IMPACT	LOC645328	CLPTM1L
IMPDH1	LOC645544	CLPX
ING3	LOC645571	CLRN1-AS1
INO80C	LOC645763	CLRN3
INSIG1	LOC645862	CLSTN1
INSL6	LOC646044	CLSTN2
INTS2	LOC646168	CLTA
INTS3	LOC646392	CLTC
INTS6	LOC646719	CLUL1
INTU	LOC646730	CLVS1
IPCEF1	LOC647002	CLYBL
IPMK	LOC647169	CMA1
IPO11	LOC647302	CMAHP
IPO5	LOC647503	CMBL
IQCA1	LOC647532	CMC1
IQCF5	LOC648987	CMTM6
IRX4	LOC650157	CMTM7
ISX	LOC653175	CMTM8
ITGA10	LOC678655	CNBD1
ITGA11	LOC724034	CNBP
ITGB3	LOC724084	CNDP1
IVD	LOC727849	CNGB3
IVNS1ABP	LOC728247	CNIH4
IWS1	LOC728344	CNNM2

KALP	LOC728537	CNOT10
KAT7	LOC728613	CNOT2
KAZN	LOC728622	CNOT4
KBTBD10	LOC728723	CNOT6L
KBTBD3	LOC728947	CNR1
KBTBD5	LOC730129	CNST
KCMF1	LOC731308	CNTLN
KCNIP1	LOXHD1	CNTN1
KCNJ1	LPA	CNTN3
KCNJ5	LPAR6	CNTN4
KCNJ6	LPCAT1	CNTN5
KCNN3	LRRC10	CNTN6
KCP	LRRC37A2	CNTNAP2
KCTD12	LRRC37BP1	CNTNAP3B
KCTD20	LSM6P1	CNTNAP4
KCTD6	LYG1	CNTNAP5
KDM4A	LYNX1	CNTRL
KDM4B	LYPLAL1	COBL
KDM4DL	LYSMD3	COBLL1
KDR	MADD	COG3
KERA	MAGEF1	COG5
KHNYN	MAGEL2	COL14A1
KIAA0040	MANBA	COL19A1
KIAA0087	MANSC4	COL1A2
KIAA0226L	MAP1LC3B2	COL21A1
KIAA0247	MAP2K6	COL22A1
KIAA0408	MAPK6PS2	COL25A1
KIAA0415	MARS	COL27A1
KIAA0430	MATN3	COL28A1
KIAA1024	MCART3P	COL2A1
KIAA1045	MCART5P	COL4A3
KIAA1143	MCART6	COL4A3BP
KIAA1199	MCM6	COL5A2
KIAA1310	MCRS1	COL6A4P1
KIAA1462	MDH2	COL6A4P2
KIAA1522	MECP2	COL6A5
KIAA1524	MEGF11	COL6A6
KIAA1841	MEIG1	COL8A1
KIAA1908	MEIS1	COLEC10
KIF16B	MEP1B	COLEC11
KIF1B	METTL15P2	COLEC12
KIF23	METTL16	COMMD10
KIRREL3	METTL6	COPG2
KLF5	MEX3B	COPS2
KLF6	MFSD2B	COPS5

KLHL2	MFSD5	COQ10B
KLHL32	MGC16075	COQ3
KLHL5	MICD	CORIN
KLHL9	MIER2	CORO1C
KLK4	MIER3	CORO2A
KLRAQ1	MIR129-1	COX10
KLRC4-KLRI	MIR206	COX5AP1
KLRD1	MIR216A	COX6B1P1
KNTC1	MIR3118-1	COX6C
KPNA5	MIR3150A	CP
KPNA7	MIR3686	CPA2
KRBOX1	MIR3688-1	CPA6
KREMEN1	MIR3910-1	CPB1
KRT18P19	MIR3927	CPD
KRT18P2	MIR3975	CPE
KRT18P43	MIR4430	CPEB1
KRT18P45	MIR4443	CPEB2
KRT18P8	MIR4464	CPEB3
KRT19P3	MIR4491	CPEB4
KRT222	MIR4494	CPHL1P
KRT223P	MIR4495	CPM
KRT34	MIR4508	CPNE4
KRT40	MIR4510	CPNE8
KRT8P1	MIR4757	CPS1
KRT8P16	MIR548AE1	CPSF3
KRT8P18	MIR548X2	CPSF6
KRT8P2	MIR550B2	CPVL
KRT8P25	MKI67IP	CR1
KRT8P40	MKRN9P	CR1L
KRTAP13-1	MMP27	CR2
KRTAP19-7	MMRN1	CRADD
KRTAP21-2	MNS1	CRB1
KRTAP25-1	MOSC1	CRBN
KRTAP27-1	MPP4	CRCP
KRTAP4-5	MPZL2	CREB1
KRTAP7-1	MR1	CREB3L1
LACE1	MRPL11P3	CREB3L2
LACTB	MRPL15P1	CREB5
LACTB2	MRPL2P1	CREBBP
LALBA	MRPL36	CREM
LAMB3	MRPL45P2	CRIM1
LAMC2	MRPS29P2	CRIP3
LAMC3	MRPS31P1	CRISP2
LANCL1	MRPS36P1	CRISPLD1
LATS2	MRS2P1	CRLF3

LAX1	MS4A3	CRTAP
LCE1F	MS4A7	CRTC3
LCE5A	MSMB	CRY1
LCN1P1	MSNP1	CRYBG3
LCT	MSRB2	CSAD
LCTL	MST1P2	CSDA
LDHA	MSTN	CSDE1
LECT2	MTFMT	CSGALNACT1
LEFTY1	MTL5	CSGALNACT2
LEFTY2	MTMR6	CSMD1
LEFTY3	MTNR1B	CSMD2
LEO1	MTOR	CSMD3
LHFPL2	MTRNR2L12	CSNK1A1
LHFPL5	MTRNR2L5	CSNK1G1
LHX2	MTRR	CSNK1G3
LHX4	MYL12A	CSPP1
LILRB1	MYL6P3	CSRNP3
LIMS3	MYNN	CSRP2
LIMS3-LOC4	MYO5BP1	CSTA
LIN28AP1	MZF1	CSTF3
LIN52	NAA30	CTAGE5
LIN54	NAA38	CTBP2
LINC00114	NACA	CTDSPL
LINC00115	NACAD	CTDSPL2
LINC00116	NADSYN1	CTGLF10P
LINC00161	NANS	CTIF
LINC00173	NARS	CTNNA1
LINC00202	NASP	CTNNA2
LINC00256B	NAT10	CTNNA3
LINC00273	NBPF18P	CTNNAL1
LINC00277	NBR1	CTNNBL1
LINC00290	NDUFB6	CTNND1
LINC00303	NDUFS6	CTNND2
LINC00314	NECAP1	CTSL1
LINC00317	NEDD1	CTSL1P2
LINC00427	NEK6	CTSL2
LINS	NETO2	CTSS
LIPT1	NF1P5	CTTNBP2
LMO2	NFATC1	CUBN
LMOD1	NFU1	CUL1
LOC1001011	NFYC	CUL2
LOC1001012	NGDN	CUL3
LOC1001278	NGFRAP1	CUL4B
LOC1001279	NHLH2	CUL5
LOC1001279	NICN1	CUL9

LOC1001280	NKX2-8	CUX1
LOC1001280	NKX3-1	CUX2
LOC1001280	NMI	CWC27
LOC1001281	NMS	CWF19L2
LOC1001282	NMT1	CXADR
LOC1001282	NOBOX	CXCL1P
LOC1001282	NOX3	CXXC1
LOC1001284	NPBWR1	CYB5R4
LOC1001284	NPM1P14	CYBRD1
LOC1001284	NPM1P21	CYCSP30
LOC1001284	NPM1P22	CYCSP55
LOC1001285	NPM1P4	CYCTP
LOC1001286	NPM1P6	CYLC2
LOC1001286	NR1I2	CYP20A1
LOC1001287	NT5C1B-RDH	CYP27C1
LOC1001287	NUAK1	CYP2C18
LOC1001288	NUDT2	CYP2C19
LOC1001288	NUDT5	CYP2C8
LOC1001289	NUDT6	CYP2C9
LOC1001289	NUDT9	CYP39A1
LOC1001289	NUP37	CYP3A4
LOC1001290	NUP54	CYP3A43
LOC1001290	NXN	CYP7B1
LOC1001291	OAS1	CYSLTR1
LOC1001292	OGN	CYTH3
LOC1001292	OIT3	DAB1
LOC1001292	OMD	DAB2IP
LOC1001293	OR10A7	DACH1
LOC1001293	OR10U1P	DAGLB
LOC1001293	OR10Y1P	DAP
LOC1001294	OR11H12	DAPK1
LOC1001294	OR11L1	DAPK2
LOC1001295	OR13F1	DAPL1
LOC1001295	OR14A2	DARS2
LOC1001296	OR14C36	DAZ1
LOC1001296	OR14K1	DAZL
LOC1001297	OR1J1	DBC1
LOC1001298	OR2AL1P	DBR1
LOC1001299	OR2C3	DCAF10
LOC1001299	OR2G3	DCAF12
LOC1001299	OR2K2	DCAF17
LOC1001300	OR2M3	DCAF4L2
LOC1001300	OR2M7	DCAF5
LOC1001300	OR2Q1P	DCBLD2
LOC1001301	OR2T10	DCC

LOC1001301	OR2T11	DCDC1
LOC1001302	OR2T3	DCDC2
LOC1001302	OR2T34	DCDC2C
LOC1001302	OR4C10P	DCDC5
LOC1001302	OR4C3	DCK
LOC1001304	OR4F6	DCLK1
LOC1001304	OR4G3P	DCLK2
LOC1001305	OR4K12P	DCP1A
LOC1001305	OR4K13	DCUN1D1
LOC1001305	OR4K16P	DCUN1D4
LOC1001305	OR4K2	DCUN1D5
LOC1001306	OR4K3	DDAH1
LOC1001307	OR4K7P	DDC
LOC1001307	OR4S1	DDHD1
LOC1001309	OR5AC1	DDI2
LOC1001311	OR5AN2P	DDX1
LOC1001311	OR5BC1P	DDX10
LOC1001312	OR5F1	DDX11L2
LOC1001312	OR5G4P	DDX31
LOC1001313	OR5H5P	DDX4
LOC1001313	OR5H6	DDX46
LOC1001314	OR5K2	DDX50
LOC1001314	OR5M10	DDX52
LOC1001315	OR6C3	DDX58
LOC1001315	OR6C4	DDX59
LOC1001316	OR6C5P	DDX6
LOC1001316	OR6C6	DDX60
LOC1001316	OR6C71P	DDX60L
LOC1001316	OR6C72P	DEC1
LOC1001316	OR6D1P	DECR1
LOC1001317	OR6F1	DEGS1
LOC1001317	OR6L1P	DEK
LOC1001317	OR7E102P	DENND1A
LOC1001319	OR7E104P	DENND1B
LOC1001319	OR7E105P	DENND2A
LOC1001319	OR7E159P	DENND3
LOC1001322	OR8A1	DENND4A
LOC1001322	OR8A2P	DENND4C
LOC1001322	OR8B8	DENND5B
LOC1001325	OR9A4	DEPTOR
LOC1001326	ORC3	DERA
LOC1001326	ORC6	DFNA5
LOC1001326	OS9	DFNB31
LOC1001326	OSGEPL1	DFNB59
LOC1001327	OSTBETA	DGKB

LOC1001327	OSTF1P1	DGKG
LOC1001327	OTOG	DGKH
LOC1001330	OTOL1	DGKI
LOC1001330	OXNAD1	DHFR
LOC1001332	OXTR	DHRS7B
LOC1001333	PAAF1	DHRS9
LOC1001333	PABPC1P10	DHRSX
LOC1001333	PAICSP5	DHX15
LOC1001333	PAK1IP1	DHX16
LOC1001347	PAPOLA	DHX32
LOC1001350	PAPOLG	DHX36
LOC1001877	PARP11	DHX40
LOC1002163	PARP6	DHX8
LOC1002164	PAX8	DHX9
LOC1002332	PAXIP1	DIAPH1
LOC1002706	PC	DIAPH2
LOC1002869	PCDH10	DIAPH3
LOC1002869	PCGEM1	DICER1
LOC1002870	PCGF2	DIDO1
LOC1002870	PCGF6	DIEXF
LOC1002871	PCNPP2	DIP2B
LOC1002872	PCSK2	DIP2C
LOC1002873	PDCD2	DIRAS2
LOC1002874	PDE5A	DIRC2
LOC1002875	PDHA2	DIRC3
LOC1002875	PDK1	DIS3L2
LOC1002878	PECR	DISC1
LOC1002878	PELI2	DISP1
LOC1002878	PEMT	DIXDC1
LOC1002880	PEX12P1	DKFZP564C196
LOC1002880	PEX2	DKFZp686O1327
LOC1002880	PEX3	DKK2
LOC1002881	PEX5L-AS2	DLAT
LOC1002881	PGAM1P13	DLD
LOC1002881	PGAM1P2	DLEU2
LOC1002881	PGBD3P3	DLEU7
LOC1002881	PHB	DLG1
LOC1002885	PHC1	DLG2
LOC1002885	PHF10	DLG5
LOC1002885	PHLDB1	DLGAP1
LOC1002885	PI4K2A	DLGAP5
LOC1002886	PIGX	DLK2
LOC1002887	PILRB	DLX1
LOC1002889	PIM1	DLX6-AS1
LOC1002889	PIPOX	DMGDH

LOC1002890	PITRM1	DMRT1
LOC1002890	PKD2L2	DMRT2
LOC1002891	PKIG	DMRTA1
LOC1002892	PLA2G1B	DMXL1
LOC1002892	PLCXD1	DMXL2
LOC1002892	PLEKHA1	DNA2
LOC1002893	PLEKHA3	DNAH10
LOC1002893	PLEKHG7	DNAH11
LOC1002893	PLRG1	DNAH12
LOC1002894	PM20D1	DNAH14
LOC1003037	PMP2	DNAH5
LOC1003139	PMPCA	DNAH6
LOC1004187	POLA1	DNAH7
LOC1004187	POLA2	DNAH8
LOC1004187	POLE2	DNAH9
LOC1004189	POLK	DNAI1
LOC1004191	POLR2B	DNAJA1
LOC1004193	POLR2D	DNAJA1P2
LOC1004193	POLR3B	DNAJB6
LOC1004194	PON2	DNAJC1
LOC1004195	POTEC	DNAJC10
LOC1004195	PP2672	DNAJC12
LOC1004196	PPAPDC2	DNAJC13
LOC1004196	PPATP1	DNAJC15
LOC1004197	PPIA	DNAJC17
LOC1004197	PPIAP14	DNAJC2
LOC1004198	PPIAP18	DNAJC21
LOC1004198	PPIL1P1	DNAJC24
LOC1004199	PPIL3	DNAJC25-GNG10
LOC1004200	PPIP5K1	DNAJC5
LOC1004200	PPP1R12BP	DNAJC5B
LOC1004200	PPP1R2	DNM1L
LOC1004200	PPP1R3D	DNM1P46
LOC1004201	PPP6R2	DNMBP
LOC1004201	PRCP	DNMBP-AS1
LOC1004203	PRDM13	DNTT
LOC1004203	PRDM2	DOCK1
LOC1004204	PRDM9	DOCK10
LOC1004204	PREX1	DOCK3
LOC1004204	PRKAB1	DOCK4
LOC1004204	PROK2	DOCK7
LOC1004204	PROSER1	DOCK8
LOC1004205	PRPF4B	DOCK9
LOC1004207	PRPS1L1	DOK6
LOC1004207	PRPSAP2	DOPEY1

LOC1004207	PRSS37	DPAGT1
LOC1004208	PRSS44	DPM1
LOC1004208	PRUNE	DPP10
LOC1004208	PSAT1	DPP4
LOC1004209	PSMA2	DPP6
LOC1004209	PSMA4	DPY19L1
LOC1004209	PSMB3	DPY19L1P1
LOC1004210	PSMB3P	DPY19L2
LOC1004211	PSMB5	DPY19L2P2
LOC1004211	PSMC1P2	DPY19L2P3
LOC1004211	PSMC1P8	DPY19L2P4
LOC1004211	PSMC6	DPY19L4
LOC1004212	PSMD4	DPYS
LOC1004212	PSPH	DPYSL5
LOC1004212	PTCD3	DRAM1
LOC1004212	PTCRA	DRD3
LOC1004213	PTGS1	DRD5P2
LOC1004213	PYGO1	DRGX
LOC1004213	RAB12	DROSHA
LOC1004213	RAB13	DSC1
LOC1004213	RAB35	DSCAM
LOC1004214	RAB40B	DSCC1
LOC1004214	RAB7L1	DSCR6P1
LOC1004214	RACGAP1P	DSE
LOC1004215	RAD1	DSG2
LOC1004215	RAD21L1	DSG4
LOC1004215	RALB	DST
LOC1004216	RAMP2	DSTN
LOC1004216	RANP1	DSTYK
LOC1004216	RARG	DTHD1
LOC1004216	RARRES1	DTL
LOC1004216	RARS	DTNA
LOC1004216	RASSF9	DTNB
LOC1004216	RAVER2	DTWD2
LOC1004217	RAX	DUOX1
LOC1004217	RBBP4P5	DUSP10
LOC1004217	RBM26	DUT
LOC1004218	RBM27	DUXAP2
LOC1004219	RBM28	DYM
LOC1004220	RBM4B	DYNC1I1
LOC1004221	RBM5	DYNC1I2
LOC1004222	RBPJP6	DYNC1LI1
LOC1004222	RCHY1	DYNC2H1
LOC1004222	RCN2	DYRK1A
LOC1004223	RDH12	DYRK3

LOC1004224	RDH16	DYTN
LOC1004224	REG4	DYX1C1-CCPG1
LOC1004225	RELA	DZIP1
LOC1004225	REREP3	DZIP3
LOC1004226	RFESD	E2F5
LOC1004991	RGS17	EAPP
LOC1004991	RGS18	EBAG9
LOC1005054	RHEBL1	ECHDC1
LOC1005054	RILPL2	ECT2
LOC1005055	RNF11	ECT2L
LOC1005055	RNF114	EDAR
LOC1005055	RNF128	EDARADD
LOC1005055	RNF138P1	EDC3
LOC1005055	RNF139	EDEM1
LOC1005056	RNF20	EDEM3
LOC1005056	RNF34	EDIL3
LOC1005056	RNPEP	EDN1
LOC1005057	RNU1-2	EDNRA
LOC1005057	RNU105C	EEA1
LOC1005057	RNU7-14P	EED
LOC1005057	RNU7-15P	EEF1A1P13
LOC1005058	RNU7-17P	EEF1A1P17
LOC1005058	RNU7-22P	EEF1A1P27
LOC1005058	RNU7-2P	EEF1A1P28
LOC1005058	RNU7-30P	EEF1A1P37
LOC1005058	RNU7-34P	EEF1DP3
LOC1005058	RNU7-53P	EEF1G
LOC1005058	RNY3	EEFSEC
LOC1005058	RNY4P18	EEPD1
LOC1005059	RNY5	EFCAB1
LOC1005059	ROPN1L	EFCAB11
LOC1005059	RPIA	EFCAB2
LOC1005059	RPL10AP9	EFCAB5
LOC1005059	RPL10P1	EFCAB6
LOC1005059	RPL10P13	EFHA1
LOC1005059	RPL10P4	EFHB
LOC1005059	RPL12P17	EFNA5
LOC1005060	RPL12P25	EFR3A
LOC1005061	RPL13AP22	EFTUD1
LOC1005061	RPL13AP23	EGFEM1P
LOC1005061	RPL13P8	EGFLAM
LOC1005062	RPL14	EGFR
LOC1005062	RPL14P1	EGLN1
LOC1005063	RPL15P3	EHBP1
LOC1005063	RPL17P19	EHD4

LOC1005064	RPL17P23	EHF
LOC1005064	RPL17P31	EHHADH
LOC1005064	RPL17P38	EHMT1
LOC1005064	RPL18AP8	EI24
LOC1005064	RPL18P4	EIF2AK1
LOC1005064	RPL18P6	EIF2AK2
LOC1005064	RPL18P9	EIF2AK4
LOC1005065	RPL21P106	EIF2C2
LOC1005065	RPL21P17	EIF2C3
LOC1005065	RPL21P43	EIF2S1
LOC1005065	RPL21P44	EIF2S2P4
LOC1005065	RPL21P56	EIF3B
LOC1005066	RPL21P59	EIF3E
LOC1005066	RPL21P66	EIF3H
LOC1005066	RPL21P68	EIF3J
LOC1005066	RPL21P92	EIF4B
LOC1005067	RPL22P19	EIF4BP8
LOC1005067	RPL23AP15	EIF4E
LOC1005068	RPL23AP19	EIF4E3
LOC1005068	RPL23AP22	EIF4G3
LOC1005068	RPL23AP23	EIF4H
LOC1005068	RPL23AP38	EIF5A2
LOC1005068	RPL23AP4	EIF5B
LOC1005068	RPL23AP40	ELAC1
LOC1005069	RPL23AP53	ELAVL2
LOC1005069	RPL23AP61	ELF1
LOC1005069	RPL23P5	ELK1P1
LOC1005069	RPL26P2	ELK3
LOC1005069	RPL26P31	ELK4
LOC1005070	RPL26P32	ELL2
LOC1005070	RPL27AP6	ELMO1
LOC1005070	RPL30P10	ELMOD1
LOC1005070	RPL30P12	ELOVL2
LOC1005070	RPL31	ELOVL6
LOC1005070	RPL31P39	ELOVL7
LOC1005071	RPL31P40	ELP2
LOC1005071	RPL31P43	ELP4
LOC1005071	RPL31P48	EMBP1
LOC1005071	RPL31P51	EMCN
LOC1005071	RPL31P59	EMID2
LOC1005072	RPL32P25	EMILIN2
LOC1005072	RPL32P4	EML4
LOC1005072	RPL35AP9	EML5
LOC1005072	RPL36AP30	EML6
LOC1005072	RPL36AP40	ENAH

LOC1005072	RPL36AP6	ENDOD1
LOC1005073	RPL37	ENKUR
LOC1005073	RPL37P10	ENOSF1
LOC1005073	RPL37P20	ENOX1
LOC1005073	RPL39P22	ENPP1
LOC1005073	RPL39P23	ENPP2
LOC1005074	RPL4P5	ENPP5
LOC1005074	RPL5P20	ENSA
LOC1005074	RPL5P24	ENTHD1
LOC1005074	RPL6P18	ENTPD1
LOC1005075	RPL6P21	ENTPD3
LOC1005075	RPL7AP28	ENTPD3-AS1
LOC1005075	RPL7AP38	ENTPD5
LOC1005075	RPL7AP52	ENTPD7
LOC1005075	RPL7P4	EP300
LOC1005075	RPL9P15	EPB41
LOC1005075	RPL9P24	EPB41L2
LOC1005075	RPL9P26	EPB41L3
LOC1005076	RPL9P28	EPB41L4A
LOC1005076	RPLP0P6	EPB41L4B
LOC1005076	RPLP2P3	EPB41L5
LOC1005076	RPS12P28	EPC1
LOC1005076	RPS12P4	EPC2
LOC1005094	RPS15AP27	EPG5
LOC1005268	RPS15AP35	EPHA1
LOC1005268	RPS15AP4	EPHA3
LOC1005336	RPS15P4	EPHA5
LOC1005336	RPS17P14	EPHA6
LOC1005336	RPS17P15	EPHA7
LOC1005336	RPS18P6	EPHB1
LOC1005336	RPS23P1	EPHB6
LOC1005337	RPS26P30	EPHX1
LOC1005337	RPS26P35	EPM2A
LOC1005337	RPS27	EPRS
LOC1005338	RPS27AP5	EPS15
LOC1005338	RPS27L	EPS8
LOC1005338	RPS27P21	EPSTI1
LOC1006527	RPS28P7	EPYC
LOC1006527	RPS29P17	ERAP1
LOC1006527	RPS29P5	ERAP2
LOC1006527	RPS29P9	ERBB2IP
LOC1006527	RPS2P31	ERBB4
LOC1006528	RPS3AP23	ERC1
LOC1006528	RPS4XP11	ERC2
LOC1006528	RPS4XP18	ERCC6

LOC1006528	RPS4XP9	ERG
LOC1006528	RPS6P12	ERGIC2
LOC1006528	RPS6P19	ERMP1
LOC1006528	RPSAP1	ERO1L
LOC1006528	RPSAP10	ERO1LB
LOC1006529	RQCD1	ERP44
LOC1006529	RRP12	ESCO1
LOC1006529	RSAD2	ESPNP
LOC1006529	RSL24D1	ESR1
LOC1006529	RSPH3	ESRP1
LOC1006529	RTN4IP1	ESRRG
LOC1006529	RUNX1	ESYT2
LOC1006529	RWDD4P2	ETF1
LOC1006529	RXFP2	ETFA
LOC1006530	RYBP	ETS1
LOC1006530	S100A11P3	ETV1
LOC1006530	S100A2	EXD1
LOC121014	S100B	EXD3
LOC127841	SACM1L	EXO1
LOC134997	SALL4	EXOC1
LOC138864	SAMD9	EXOC2
LOC145694	SAMD9L	EXOC3
LOC145845	SAMSN1	EXOC4
LOC146880	SCAND3	EXOC6
LOC147804	SCML4	EXOC6B
LOC149773	SCN2A	EXPH5
LOC150381	SCNN1A	EXT1
LOC150577	SCRN2	EXT2
LOC154872	SCRN3	EYA1
LOC157381	SDF2	EYA2
LOC157667	SDR16C5	EYA3
LOC158435	SDR39U1	EYA4
LOC158948	SEC24D	EYS
LOC1720	SECISBP2	EZH1
LOC200726	SELT	EZH2
LOC202319	SEMA5B	EZR
LOC219731	SEPHS1P7	F13A1
LOC220077	SEPT10P1	F2
LOC220729	SEPT15	FAAH2
LOC220980	SEPT2	FABP7
LOC255308	SERTAD4	FAF1
LOC283014	SFTA1P	FAIM
LOC283033	SGOL1	FAIM3
LOC283050	SGPP2	FAM105A
LOC283688	SGSM1	FAM105B

LOC283731	SH3PXD2B	FAM107B
LOC284998	SHB	FAM108A7P
LOC285084	SIGMAR1	FAM110B
LOC285103	SIKE1	FAM110C
LOC285286	SKA2L	FAM111B
LOC285326	SLC17A4	FAM115A
LOC285359	SLC20A2	FAM116A
LOC285740	SLC23A2	FAM117A
LOC285878	SLC24A1	FAM117B
LOC285943	SLC25A31	FAM118B
LOC286094	SLC25A5P8	FAM120A
LOC286177	SLC25A5P9	FAM125B
LOC286184	SLC28A2	FAM126A
LOC286190	SLC29A2	FAM126B
LOC338739	SLC2A2	FAM129A
LOC339240	SLC2A8	FAM129B
LOC340107	SLC35A1	FAM134B
LOC341965	SLC35C1	FAM135A
LOC342784	SLC40A1	FAM135B
LOC343052	SLC46A2	FAM138D
LOC344978	SLC5A9	FAM138E
LOC345016	SLC6A20	FAM13A
LOC347674	SLC7A13	FAM13B
LOC348958	SLC9B1P2	FAM13C
LOC375295	SLFN5	FAM149B1
LOC387646	SLIT1	FAM150B
LOC387647	SMARCA5	FAM154A
LOC387820	SMARCE1P3	FAM155A
LOC387869	SMARCE1P5	FAM160A1
LOC388387	SMC3	FAM162A
LOC388553	SMEK2	FAM163A
LOC388579	SMG1	FAM164A
LOC388692	SNORD59A	FAM168A
LOC389043	SNPH	FAM168B
LOC389137	SNX18P7	FAM169A
LOC389473	SNX19P3	FAM171B
LOC389602	SOBP	FAM172A
LOC389768	SP2	FAM174B
LOC390233	SP5	FAM175B
LOC390250	SPCS1	FAM177A1
LOC390251	SPCS2	FAM179B
LOC390311	SPINK1	FAM185A
LOC390332	SPINK6	FAM185BP
LOC390340	SPON2	FAM186B
LOC390358	SPRY1	FAM188A

LOC390363	SPTLC2	FAM188B
LOC390572	SREBF2	FAM189A1
LOC390618	SRRT	FAM190A
LOC390858	SRSF9	FAM194A
LOC390860	SS18L1	FAM196A
LOC391405	SSBP1	FAM198B
LOC391436	SSR1	FAM19A1
LOC391526	ST13P1	FAM19A2
LOC391559	ST13P3	FAM19A4
LOC391566	ST13P7	FAM208A
LOC391642	ST20	FAM208B
LOC391686	ST7-AS2	FAM20B
LOC391744	STAG3L4	FAM24B-CUZD1
LOC391747	STARD6	FAM36A
LOC392226	STRAP	FAM40B
LOC392264	STX11	FAM41C
LOC392266	STX19	FAM46C
LOC399715	STX2	FAM48A
LOC399939	SUCLA2P1	FAM49B
LOC400654	SULT1C3	FAM50B
LOC401134	SUPT16HP	FAM53B
LOC401164	SUV39H2	FAM55B
LOC401324	SUZ12P	FAM55C
LOC401463	SYN3	FAM55D
LOC401533	TAAR9	FAM59A
LOC401677	TACR1	FAM5B
LOC402096	TAF1L	FAM5C
LOC402112	TAF9P3	FAM60A
LOC402279	TAS2R38	FAM63B
LOC402644	TBK1	FAM71F1
LOC407835	TBL2	FAM75D5
LOC439953	TBPL2	FAM81A
LOC440063	TC2N	FAM82A1
LOC440311	TCEA1	FAM83H
LOC440742	TCEAL5	FAM89A
LOC440993	TCEB2P2	FAM91A3P
LOC441204	TCP11L1	FAM92A1
LOC441416	TCTN1	FAM96A
LOC441454	TDRG1	FAM98B
LOC441601	TDRKH	FAN1
LOC441915	TFDP1	FANCC
LOC442075	TFDP1P	FANCD2
LOC442087	TGFBR2	FANCF
LOC442156	TGIF2P1	FANCL
LOC442308	TGM3	FANCM

LOC442318	TGS1	FANK1
LOC442389	THEM5	FAP
LOC442427	THOC7	FAR1P1
LOC442713	TIA1	FAR2
LOC442727	TIGD3	FARS2
LOC51145	TM4SF19	FASTKD1
LOC641456	TMBIM4	FAT1
LOC641924	TMC5	FAT3
LOC642461	TMCO5A	FAT4
LOC642474	TMED7	FBLN7
LOC642590	TMED8	FBN1
LOC642890	TMEM136	FBN2
LOC642897	TMEM141	FBP1
LOC642935	TMEM14B	FBXL13
LOC642943	TMEM165	FBXL17
LOC643064	TMEM168	FBXL2
LOC643126	TMEM18	FBXL20
LOC643276	TMEM22	FBXL4
LOC644192	TMEM26	FBXL7
LOC644242	TMEM30A	FBXO10
LOC644390	TMEM37	FBXO11
LOC644425	TMEM5	FBXO16
LOC644436	TMEM50A	FBXO18
LOC644482	TMEM50B	FBXO21
LOC644566	TMEM59	FBXO22
LOC644589	TMEM66	FBXO28
LOC644662	TMEM68	FBXO32
LOC644667	TMEM70	FBXO33
LOC644714	TMEM8B	FBXO34
LOC644871	TMEM9	FBXO38
LOC645065	TMEM97	FBXO42
LOC645086	TMEM98	FBXO47
LOC645177	TMLHE	FBXW11
LOC645233	TMTC4	FBXW7
LOC645355	TMX2	FBXW8
LOC645397	TMX3	FCAMR
LOC645405	TOMM20L	FCGR1A
LOC645415	TPBG	FCHO2
LOC645503	TPD52L3	FCHSD2
LOC645529	TPTE2P3	FDX1
LOC645551	TRAPPC6B	FECH
LOC645598	TRBV21OR9	FEM1C
LOC645900	TRHR	FER
LOC645937	TRIB2	FER1L6
LOC645949	TRIB3	FER1L6-AS1

LOC646021	TRIM50	FERMT2
LOC646096	TRIM52	FGD3
LOC646114	TRIM54	FGD4
LOC646214	TRIM60P18	FGD5
LOC646227	TRIM64C	FGD6
LOC646278	TRIP11	FGF12
LOC646324	TRNAA20	FGF14
LOC646388	TRNAC10	FGFR1OP2
LOC646505	TRNAC26	FGGY
LOC646614	TRNAC4	FHIT
LOC646674	TRNAE21	FHOD3
LOC646801	TRNAE27P	FIG4
LOC646873	TRNAE28P	FIGN
LOC647012	TRNAE32P	FILIP1
LOC647013	TRNAG27	FILIP1L
LOC647323	TRNAI25	FIP1L1
LOC647996	TRNAK16	FKBP15
LOC649024	TRNAM11	FKBP1A-SDCBP2
LOC651714	TRNAM16	FKBP5
LOC653739	TRNAN11	FKBP7
LOC653895	TRNAQ3	FKBP9
LOC654340	TRNAQ36P	FKTN
LOC727944	TRNAR30P	FLG
LOC728064	TRNAT12	FLJ13197
LOC728066	TRNAT19	FLJ20444
LOC728095	TRNAV22	FLJ22447
LOC728333	TRNAY2	FLJ22763
LOC728477	TSC22D4	FLJ25363
LOC728586	TSHB	FLJ30838
LOC728606	TSPAN3	FLJ31306
LOC728667	TSPAN5	FLJ32255
LOC728675	TSPEAR	FLJ32955
LOC728724	TSRM	FLJ33065
LOC728739	TTC19	FLJ33630
LOC728996	TTC3	FLJ34208
LOC729082	TTC30A	FLJ34690
LOC729101	TTC4P1	FLJ35024
LOC729141	TUBA1C	FLJ35282
LOC729200	TUBBP5	FLJ37396
LOC729366	TULP4	FLJ37786
LOC729466	TXN	FLJ39080
LOC729532	TXNDC12	FLJ39534
LOC729570	TXNDC5	FLJ39739
LOC729686	TXNL1	FLJ41278
LOC729739	TYW5	FLJ42709

LOC729911	UAP1L1	FLJ43663
LOC729960	UBA5	FLJ45872
LOC730076	UBA52P1	FLJ45974
LOC730086	UBC	FLJ46010
LOC730098	UBE2D2	FLJ46066
LOC730101	UBE2D3	FLJ46361
LOC730144	UBE2QL1	FLNB
LOC731789	UBFD1P1	FLOT1
LOC767850	UBR2	FLVCR1
LOC81691	UBTFL7	FLYWCH1P1
LOC93432	UFM1	FMN1
LPP-AS2	UGT2B10	FMN2
LRRC20	UGT8	FMNL2
LRRC23	UNC50	FMO5
LRRC27	UNC79	FNBP1
LRRC34	UNG	FNBP4
LRRC36	UPK3A	FNDC3A
LRRC40	UQCRQ	FNDC3B
LRRC59	USP16	FNIP1
LRRC63	USP22	FNIP2
LRRIQ3	USP37	FOLH1
LRRIQ4	USP4	FOLH1B
LRRN3	UTY	FONG
LRSAM1	VDAC2	FOXA1
LRTOMT	VGLL3	FOXH1
LSAMP-AS3	VN1R17P	FOXJ3
LSG1	VN1R5	FOXK1
LSS	VN1R54P	FOXN2
LTF	VN2R9P	FOXN3
LUM	VPS37A	FOXN4
LUZP1	VPS39	FOXO3
LY6H	VPS72	FOXP1
LY6K	VSIG10	FOXP2
LYG2	VSTM5	FPGT-TNNI3K
LYRM4	VTI1B	FRAS1
LYRM7	WDFY3-AS2	FRAT2
LYZL4	WDR45L	FREM1
LYZL6	WDR61	FREM2
MAB21L3	WDR93	FRG1
MACF1	WEE1	FRG1B
MAK16	WEE2	FRK
MALL	WIPI2	FRMD3
MAN1A1	XRCC6	FRMD4A
MAN1C1	YBX1P2	FRMD4B
MANBAL	YIPF1	FRMD5

MANEA	YRDCP2	FRMD6
MAP3K2	YTHDF2	FRMPD1
MAP4K3	YWHAQP7	FRS2
MAP9	YWHAZP3	FRY
MAPK1	ZBED3	FRYL
MAPK14	ZBTB26	FSD1L
MAPKBP1	ZBTB34	FSD2
MAPRE1	ZBTB7B	FSHR
MARCH10	ZBTB8A	FSIP1
MARCH3	ZC3H12C	FSIP2
MARCKS	ZC3H15	FSTL1
MARK2P5	ZC3H7A	FSTL5
MARK3	ZCCHC16	FTH1P12
MAST1	ZCCHC9	FTH1P20
MAX	ZDHHC23	FTH1P22
MBLAC2	ZDHHC3	FTLP12
MBOAT1	ZEB1-AS1	FURIN
MBTPS1	ZFC3H1	FUT8
MC3R	ZFP62	FUT9
MCART2	ZFP64	FXN
MDH1B	ZFP64P1	FXR1
MDM1	ZFY	FYB
MED14P1	ZFY-AS1	FZD1
MED24	ZFYVE9P2	FZD3
MED27	ZGPAT	FZD5
MED30	ZKSCAN5	FZD6
MESP2	ZMIZ1	G2E3
MEST	ZNF107	G3BP1
MESTP3	ZNF12	G3BP2
MESTP4	ZNF235	GAB1
METTL21A	ZNF274	GAB2
MFAP1	ZNF322	GABBR2
MFAP5	ZNF331	GABPA
MFHAS1	ZNF34	GABPB1
MFSD8	ZNF396	GABPB2
MIPEPP2	ZNF410	GABRA5
MIR1202	ZNF444P1	GABRB2
MIR1208	ZNF445	GABRB3
MIR122	ZNF496	GABRG3
MIR148A	ZNF503	GABRR3
MIR1827	ZNF513	GADD45G
MIR21	ZNF541	GADL1
MIR30C2	ZNF572	GALC
MIR3144	ZNF623	GALK2
MIR3153	ZNF718	GALNT11

MIR31HG	ZNF720	GALNT12
MIR331	ZNF732	GALNT13
MIR3679	ZNF77	GALNT14
MIR3688-2	ZNF831	GALNT2
MIR378D2	ZNF860	GALNT3
MIR3911	ZNF891	GALNT7
MIR3916	ZNF90	GALNT8
MIR4273	ZP2	GALNTL1
MIR4277	ZSWIM5	GALNTL2
MIR4289	ZYG11B	GALNTL5
MIR4302		GALNTL6
MIR4327		GALR1
MIR4424		GALT
MIR4442		GANC
MIR4445		GAP43
MIR4447		GAPDHP15
MIR4478		GAPDHP50
MIR4500HG		GAPDHP64
MIR4527		GAPDHP68
MIR4532		GAPVD1
MIR4693		GARNL3
MIR4703		GARS
MIR4719		GAS2
MIR4783		GAS2L3
MIR4792		GATA6
MIR548A2		GATAD2B
MIR548AI		GATM
MIR548G		GBA2
MIR570		GBE1
MIR720		GCC2
MIRLET7A1		GCG
MKRN3		GCM2
MLH3		GCNT1
MLLT11		GCNT2
MMP1		GCNT6
MMP28		GCOM1
MMP7		GDA
MOGAT1		GDAP2
MOGAT2		GDI2
MON1B		GDPD4
MORF4L2		GFM1
MPEG1		GGCT
MPRIP		GGH
MPZL3		GGNBP2
MRGPRD		GGPS1

MRP63P10	GHR
MRPL13	GIMAP2
MRPL16	GIMAP4
MRPL3	GIMAP6
MRPL33	GIT2
MRPL35	GJA3
MRPL42P4	GK5
MRPL44	GKAP1
MRPS14	GKN2
MRPS17	GLB1
MRPS18AP1	GLB1L2
MRPS23P1	GLB1L3
MRPS27	GLCCI1
MRPS31	GLDC
MRPS5	GLE1
MS4A15	GLI3
MS4A4A	GLIPR2
MS4A5	GLIS3
MS4A6E	GLRA3
MSH2	GLRX2
MST1P9	GLS
MST4	GLT1D1
MT1P2	GLT25D2
MT2P1	GLT8D2
MTAP	GLUL
MTERF	GLYATL1
MTERFD1	GLYATL2
MTERFD3	GLYATL3
MTHFD1	GLYCTK
MTND5P28	GMCL1
MTPN	GMDS
MTTP	GMNC
MTUS1	GMPS
MTUS2	GNA12
MUC17	GNA14
MUC3A	GNAI1
MUC4	GNAQ
MUCL1	GNAS
MUTED	GNAT3
MYCN	GNB4
MYF5	GNE
MYH1	GNG2
MYH13	GNG4
MYH2	GNL1
MYL4	GNMT

MYO1A	GNN
MYO1H	GNPNAT1
MYO5BP2	GNPTAB
MYO5C	GOLGA1
MYOZ2	GOLGA4
N4BP1	GOLGB1
N4BP2L1	GOLIM4
N6AMT2	GOLM1
NAA15	GOLPH3
NAA50	GOLPH3L
NAB2	GOLT1A
NACC2	GOPC
NAMPT	GORASP2
NANOGP3	GOSR1
NANOGP8	GOT1
NASPP1	GPATCH2
NBPF13P	GPATCH8
NBPF8	GPBP1
NCK2	GPBP1L1
NCOA4	GPC5
NCOA6	GPC6
NCOR2	GPCPD1
NDFIP2	GPD1L
NDUFA4	GPD2
NDUFB9	GPHN
NDUFV2	GPLD1
NEFHP1	GPM6A
NEFLP1	GPNMB
NEIL3	GPR1
NEK4P1	GPR107
NEK4P2	GPR110
NEK4P3	GPR116
NEU3	GPR126
NFE2	GPR128
NFE2L1	GPR137B
NFE2L3P1	GPR137C
NFKB1	GPR149
NFKBIE	GPR155
NFRKB	GPR156
NFYAP1	GPR158
NHEJ1	GPR160
NHS	GPR176
NID2	GPR182
NIF3L1	GPR37
NIPA1	GPR75-ASB3

NIPA2	GPR89A
NIPA2P1	GPR98
NIPA2P2	GPRIN3
NIPAL1	GRAMD1C
NKAIN1	GRAMD2
NKX2-3	GRAMD3
NLGN4Y	GRB10
NLRC3	GRB14
NLRP2P	GRB2
NLRP9	GREB1
NMD3	GREB1L
NOC3L	GREM2
NOP56P1	GRHL1
NOP58	GRHL2
NOS1	GRIA2
NOS2	GRIA4
NOV	GRID2
NPFFR2	GRIK1
NPM1P2	GRIK2
NPNT	GRIK4
NPRL3	GRIN3A
NPY	GRIP1
NR4A3	GRM1
NRARP	GRM3
NRBF2	GRM4
NRBP1	GRM5
NT5E	GRM7
NTF3	GRM8
NUDCD2	GRXCR1
NUDT12	GSDMC
NUDT15	GSK3B
NUFIP1	GSTCD
NUPL1	GSTK1
NXF2B	GSTP1
NXNL2	GSTP1P1
OBP2B	GTDC1
OBSCN	GTF2A1L
OCA2	GTF2E1
OCIAD1	GTF2E2
ODF2	GTF2F2
OFD1P3Y	GTF2I
OFD1P6Y	GTF3C3
OLIG3	GTPBP10
OLR1	GTSF1
OPN5	GUCA1C

OPRK1	GUCY1A2
OPRM1	GUCY1A3
OR10D5P	GUCY1B2
OR10G1P	GUCY1B3
OR10G7	GUK1
OR10Q2P	GULP1
OR11H7	GUSBP1
OR11K2P	GUSBP4
OR11M1P	GXYLT1
OR13A1	GXYLT2
OR13C4	GYG1
OR13C8	GYG1P1
OR13C9	GYS2
OR13D3P	GZMB
OR13I1P	H2AFJ
OR14A16	H2AFY
OR1AC1P	H2AFY2
OR1C1	H3.X
OR1H1P	H3.Y
OR1J2	H3F3A
OR1N1	H3F3B
OR1N2	HABP4
OR1S2	HACE1
OR2A14	HACL1
OR2A25	HADHA
OR2A3P	HADHB
OR2A42	HAL
OR2AJ1	HAS2
OR2AP1	HAT1
OR2E1P	HAUS2
OR2F1	HAUS6
OR2M1P	HBS1L
OR2M4	HCFC2
OR2T2	HCG27
OR2T33	HCGVIII-2
OR2T6	HCN1
OR2W5	HDAC9
OR4A17P	HDHD3
OR4A40P	HEATR1
OR4C4P	HEATR5A
OR4C5	HEATR5B
OR4K1	HEATR7A
OR4L1	HEATR7B2
OR4M1	HEBP2
OR4N1P	HECTD1

OR4N3P	HECTD2
OR4X1	HECW1
OR5AC2	HECW2
OR5AK3P	HEG1
OR5AL1	HELB
OR5AZ1P	HELLS
OR5B15P	HELZ
OR5BA1P	HERC1
OR5BK1P	HERC2
OR5BR1P	HERC2P3
OR5G5P	HERC3
OR5J2	HERC4
OR5K1	HERPUD2
OR5M1	HEXIM1
OR5M12P	HEY2
OR5M8	HFM1
OR5T2	HGD
OR5T3	HGF
OR6Q1	HHAT
OR6W1P	HHLA1
OR7E136P	HIATL1
OR7E13P	HIATL2
OR7E14P	HIBADH
OR7E7P	HIBCH
OR7E89P	HIF1A
OR8B7P	HIGD1A
OR8J3	HIP1
OR8K5	HIPK2
OR8Q1P	HIPK3
OR8T1P	HIST1H2BG
OR9G2P	HIST2H2AB
OR9G4	HIST4H4
OR9H1P	HIVEP2
OR9N1P	HK1
OR9R1P	HLA-V
ORMDL1	HLCS
OSBP	HLTF
OSBP2	HLX
OTUD1	HMBOX1
OTUD4	HMCN1
OTUD6B	HMGA1
OTUD7A	HMGA1P7
OTX2OS1	HMGA2
OXA1L	HMGB1P26
P2RY1	HMGB3P20

P2RY2	HMGB3P24
P5-05	HMGCLL1
PA2G4P5	HMGCS2
PABPC1P4	HMGN2P46
PABPC4L	HMGN2P6
PACRGL	HNF4G
PAICS	HNMT
PAIP2B	HNRNPA1P23
PAN2	HNRNPA2B1
PANK2	HNRNPA3
PANK3	HNRNPC
PAR5	HNRNPF
PARD6B	HNRNPH3
PARP1	HNRNPK
PARP16	HNRNPR
PARP3	HNRNPU
PARP9	HNRNPU-AS1
PATL2	HNRNPUL2
PAX3	HNRNPUL2-BSCL2
PAX4	HNRPLL
PBX2P1	HOOK1
PCBD2	HOOK3
PCBP3	HOPX
PCDH11Y	HOTAIR
PCDH17	HOTAIRM1
PCDH8P1	HOTTIP
PCDHAC1	HOXA10
PCDHGA10	HOXA10-HOXA9
PCDHGA6	HOXA11
PCDHGA7	HOXA13
PCDHGA8	HOXA3
PCDHGA9	HOXA9
PCDHGB3	HPDL
PCDHGB4	HPGD
PCDHGB5	HPGDS
PCDHGB6	HPS3
PCID2	HPS5
PCSK1	HPSE2
PCSK6	HRASLS2
PDCD10	HRCT1
PDCD11	HRH1
PDCD7	HS3ST4
PDCL2	HS6ST3
PDCL3	HSD17B11
PDE6A	HSD17B12

PDE6C	HSD17B3
PDE8A	HSD17B4
PDGFA	HSD17B6
PDGFRA	HSD3B1
PDIA3	HSD3BP1
PDK4	HSD3BP3
PDLIM3	HSDL2
PDPN	HSP90AB1
PEBP1	HSPA14
PENK	HSPA4
PER1	HSPA9
PER2	HSPB1P1
PEX14	HSPBAP1
PFKFB2	HSPD1P19
PFN4	HSPE1-PHOCN
PGAM2	HTR1E
PGBD3P1	HTR3B
PGDP1	HTR4
PGDP2	HTR7
PGGT1B	HYAL4
PGLYRP3	HYALP1
PGLYRP4	HYDIN
PGPEP1L	IARS
PHC1P1	IARS2
PHF17	IBTK
PHF19	ICA1
PHF20L1	ICA1L
PHF8	ICOS
PHKG1P3	ID2
PHOSPHO2	IDE
PHOX2B	IDH1
PI4KB	IDH3A
PIEZO1P1	IDI1
PIGS	IDI2-AS1
PIGU	IER3
PIP	IER5
PIPSL	IFLTD1
PITPNB	IFNA6
PITPNM2	IFRD1
PKNOX2	IFT122
PLA2G2A	IFT57
PLA2G4E	IFT74
PLA2G7	IFT80
PLAC1L	IFT81
PLCG2	IFT88

PLEKHD1	IGF1
PLK1S1	IGF1R
PLXNA2	IGF2BP1
PLXNC1	IGF2BP2
PMCH	IGF2BP3
PML	IGF2R
PMVK	IGFN1
PNKD	IGH@
PNP	IGHV2OR16-5
PNPLA4P1	IGK@
PNRC2	IGL@
POC1A	IGSF11
PODXL	IGSF3
POLD3	IKBIP
POLG	IKBKAP
POLM	IKZF1
POLR1C	IKZF2
POLR3C	IKZF3
POM121C	IL15
POM121L12	IL15RA
POMC	IL16
POP7	IL17RD
POSTN	IL19
POTEF	IL1RAP
POU1F1	IL1RAPL1
POU5F1B	IL1RAPL2
POU6F1	IL1RL2
PPARGC1A	IL20RA
PPARGC1B	IL20RB
PPAT	IL22RA2
PPBPL1	IL23R
PPFIA1	IL24
PPFIA4	IL2RA
PPFIBP2	IL33
PPIAP22	IL5RA
PPIEL	IL6R
PPIP5K2	IL6RP1
PPM1A	IL6ST
PPM1D	IL7
PPM1G	IL7R
PPM1K	IL9R
PPP1R14BP5	IL9RP2
PPP1R14C	IL9RP4
PPP2CA	ILDR1
PPP2CB	IMMP1L

PPP2R2D	IMMP2L
PPP2R3B	IMMT
PPP2R5C	IMPA1
PPP4R4	IMPA1P
PPPDE2	IMPDH1P3
PRAMEL	IMPG1
PRDM1	IMPG2
PRDM11	INA
PRDM5	INADL
PRDM7	INHBC
PRELP	INMT-FAM188B
PREP	INO80
PRKAB2	INO80D
PRKACG	INPP1
PRKCQ	INPP4A
PRKG2	INPP4B
PRKRA	INPP5A
PRKRIR	INSC
PRKRIRP3	INSM2
PRMT2	INTS4
PROM1	INTS4L1
PROSP	INTS4L2
PRPF18	INTS7
PRPF40A	INTS8
PRR14L	INTS9
PRR15	INVS
PRR23B	IP6K1
PRR3	IP6K2
PRR5L	IP6K3
PRSS23	IPO8
PRSS3	IPO9
PRSS43	IPPK
PRSS58	IQCB1
PSAP	IQCE
PSAT1P1	IQCF2
PSCA	IQCF4
PSEN2	IQCG
PSMA6	IQCH
PSMB1	IQCJ
PSMB3P2	IQCJ-SCHIP1
PSMC1P10	IQGAP1
PSMC1P12	IQGAP2
PSMD12	IQSEC1
PSMD12P	IQSEC3
PSMD5	IQUB

PSMF1	IRAK3
PTGDR	IREB2
PTGIS	IRF2BP2
PTHLH	IRF5
PTP4A2	IRF6
PTPN1	IRS1
PTPN11	IRS2
PTPN9	IRX2
PTPRA	ISCA1
PTPRT	ISPD
PTS	ITCH
PURB	ITFG1
PURG	ITGA1
PVRL3	ITGA2
PVRL3-AS1	ITGA4
PWP1	ITGA6
PYCR2	ITGA8
PYGB	ITGA9
PYROXD1	ITGAV
QRICH1	ITGB1
QRSL1	ITGB3BP
RAB11FIP3	ITGB5
RAB19	ITGB6
RAB28	ITGB8
RAB33B	ITGBL1
RAB39	ITIH5
RAB40A	ITPKB
RAB40AL	ITPR1
RAB6C	ITPR2
RAB9BP1	ITSN1
RABGGTB	ITSN2
RAC1P5	JAK2
RACGAP1	JAKMIP2
RAD50	JAM3
RAD51AP2	JAZF1
RAE1	JHDM1D
RAG1	JMJD1C
RAMP3	JMY
RANBP20P	JPH1
RAP1GDS1	KALRN
RAP2A	KANK1
RAP2B	KAT2B
RARA	KAT6A
RARS2	KAT6B
RASGEF1A	KBTBD12

RASGEF1C	KBTBD2
RASGRF1	KC6
RASGRP3	KCNAB1
RASSF5	KCNB2
RBBP6	KCNC2
RBFOX1	KCND2
RBM22P1	KCNH1
RBM4	KCNH5
RBM45	KCNH7
RBM46	KCNH8
RBMXP3	KCNIP4
RBMY2JP	KCNJ15
RBMY2MP	KCNJ3
RBP4	KCNK1
RBPJ	KCNK2
RBX1	KCNK9
RCC1	KCNMA1
RCC2P5	KCNMB2
RCC2P8	KCNMB3
RCOR1	KCNMB4
RDM1	KCNN2
REEP1	KCNQ3
REG1A	KCNQ5
REG1B	KCNT2
REG3A	KCNU1
RELL1	KCNV1
REN	KCNV2
RERGL	KCTD1
RETNLB	KCTD16
REXO2	KCTD3
RFC1	KCTD8
RFPL4A	KDELC2
RFX5	KDELR2
RFX8	KDM1A
RGPD4	KDM2A
RGS13	KDM3A
RGS16	KDM4C
RGS21	KDM4D
RGS6	KDM5B
RGS7BP	KDM5D
RHCG	KDSR
RHOBTB3	KEL
RHOQP2	KGFLP2
RHOQP3	KHDRBS2
RINT1	KHDRBS3

RLF	KIAA0100
RN7SL2	KIAA0101
RNASE1	KIAA0146
RNASE13	KIAA0232
RNASE4	KIAA0319
RNASE8	KIAA0319L
RNASE9	KIAA0355
RNASEH1P2	KIAA0368
RNASEH2C	KIAA0391
RNF121	KIAA0528
RNF135	KIAA0564
RNF157	KIAA0586
RNF217	KIAA0748
RNF220	KIAA0825
RNF4	KIAA0895
RNFT2	KIAA0947
RNU1-15P	KIAA1033
RNU4-1	KIAA1109
RNU4-5P	KIAA1147
RNU5A-1	KIAA1211
RNU5B-1	KIAA1217
RNY4	KIAA1239
ROPN1	KIAA1244
RPA2	KIAA1267
RPH3A	KIAA1324L
RPL12P4	KIAA1328
RPL12P6	KIAA1370
RPL13AP21	KIAA1377
RPL13P7	KIAA1407
RPL15	KIAA1429
RPL15P11	KIAA1430
RPL15P15	KIAA1432
RPL17	KIAA1468
RPL17-C18ORF32	KIAA1486
RPL17P32	KIAA1539
RPL17P35	KIAA1549
RPL17P37	KIAA1715
RPL18P11	KIAA1731
RPL19P15	KIAA1797
RPL21P101	KIAA1804
RPL21P107	KIAA1958
RPL21P116	KIAA2018
RPL21P117	KIAA2026
RPL21P122	KIDINS220
RPL21P38	KIF11

RPL21P40	KIF12
RPL21P41	KIF13A
RPL21P42	KIF13B
RPL21P84	KIF14
RPL21P86	KIF15
RPL21P89	KIF18A
RPL21P93	KIF21A
RPL21P98	KIF24
RPL22P11	KIF26B
RPL22P12	KIF27
RPL23A	KIF3A
RPL23AP16	KIF5B
RPL23AP29	KIF5C
RPL23AP41	KIF6
RPL23AP44	KIN
RPL23AP56	KITLG
RPL23AP68	KL
RPL23P7	KLB
RPL24	KLF12
RPL26	KLF3
RPL26P17	KLF7
RPL29P26	KLF7P1
RPL29P29	KLF9
RPL30P3	KLHDC1
RPL31P13	KLHDC10
RPL31P33	KLHDC2
RPL31P35	KLHDC5
RPL31P37	KLHDC8A
RPL31P41	KLHL1
RPL31P42	KLHL12
RPL31P44	KLHL14
RPL31P53	KLHL18
RPL32	KLHL20
RPL32P9	KLHL24
RPL34P26	KLHL28
RPL34P7	KLHL29
RPL35AP30	KLHL6
RPL35P1	KLHL7
RPL36AP21	KLHL8
RPL36AP31	KLKP1
RPL36AP47	KLRC1
RPL36P10	KLRC2
RPL37AP7	KLRC3
RPL37P16	KLRF1
RPL37P21	KLRF2

RPL37P5	KLRG1
RPL38P3	KMO
RPL39L	KNG1
RPL39P10	KPNA1
RPL39P24	KPNA4
RPL3P4	KPNB1
RPL3P8	KRAS
RPL6P3	KRIT1
RPL7A	KRT14
RPL7AP18	KRT18P28
RPL7AP2	KRT18P39
RPL7AP50	KRT18P46
RPL7AP58	KRT18P6
RPL7L1P4	KRT18P9
RPL7L1P8	KRT81
RPL7P27	KRT86
RPL7P33	KRT8P20
RPLP0P2	KRT8P28
RPLP0P5	KRT8P29
RPLP1	KRTAP6-3
RPLP1P4	KRTAP9-6
RPS10P11	KSR2
RPS10P12	KTN1
RPS12P15	KYNU
RPS12P24	KYNUP1
RPS13P7	L2HGDH
RPS14P7	L3MBTL3
RPS14P9	L3MBTL4
RPS15AP20	LAMA1
RPS17P10	LAMA2
RPS19P7	LAMA3
RPS20	LAMA4
RPS20P14	LAMB1
RPS20P19	LAMB4
RPS23	LAMC1
RPS24P13	LAMP3
RPS24P4	LANCL2
RPS25P6	LAPTM4A
RPS26P40	LAPTM4B
RPS26P54	LARGE
RPS26P9	LARP1B
RPS27AP14	LARP4
RPS27P22	LARP4B
RPS27P23	LARS2
RPS27P4	LBH

RPS27P5	LCA5
RPS2P18	LCEP2
RPS2P22	LCLAT1
RPS2P3	LCN2
RPS2P38	LCOR
RPS2P6	LCORL
RPS3	LCP1
RPS3A	LDHAP4
RPS3AP18	LDHC
RPS3AP24	LDLRAD3
RPS3AP49	LEF1
RPS4XP6	LEKR1
RPS4Y1	LEMD1
RPS6	LEMD3
RPS6P17	LEPR
RPS6P22	LEPREL1
RPS6P3	LEPROTL1
RPS6P4	LGALS3
RPS7P5	LGALS8
RPSAP11	LGI1
RPSAP17	LGR4
RPSAP42	LGR5
RPSAP44	LGR6
RPSAP52	LHFP
RPSAP59	LHFPL3
RRH	LHX9
RRM2	LIFR
RRN3	LIMA1
RRP15	LIMCH1
RSPH1	LIMD1
RSRC2	LIMS1
RTF1	LIMS3L
RTP4	LIN28B
RUFY3	LIN7A
RUNDC1	LIN9
RXRA	LINC00032
S100PBP	LINC00174
SACS	LINC00184
SAE1	LINC00189
SAFB	LINC00200
SAFB2	LINC00265
SALL4P5	LINC00271
SAMD5	LINC00272
SAP130	LINC00299
SARDH	LINC00301

SASH1	LINC00305
SCARB2	LINC00340
SCD5	LINC00461
SCFD2	LINC00467
SCG3	LINC00470
SCGB2A1	LINC00475
SCGN	LINC00476
SCLY	LINC00477
SCN11A	LINC00478
SCO1	LINC00486
SCP2	LINC00487
SDCCAG3P1	LINC00488
SDCCAG3P2	LINGO2
SDHAF2	LIPC
SDHDP3	LIPG
SDK2	LIPH
SDR16C6P	LIPI
SEC14L1	LIX1
SEC14L2	LIX1L
SEC23IP	LLPH
SEC61A1	LMAN1
SEC61A2	LMBR1
SEC61B	LMBRD1
SEC62	LMBRD2
SEL1L2	LMLN
SELENBP1	LMNB1
SELPLG	LMO3
SENP2	LMO7
SEPHS1	LMOD3
SEPHS1P1	LMTK2
SEPHS1P6	LNP1
SEPSECS	LNX1
SEPT11	LNX2
SEPT7P7	LOC100124332
SERINC1	LOC100124692
SERINC5	LOC100127907
SERPINA6	LOC100127982
SERPINB10	LOC100127989
SERPINB11	LOC100128052
SERPINB13	LOC100128095
SERPINB2	LOC100128126
SERPINB3	LOC100128164
SERPINB4	LOC100128179
SERPINB5	LOC100128324
SERPINB9	LOC100128335

SERPINE2	LOC100128360
SERPINI2	LOC100128365
SERTAD2	LOC100128441
SETD3	LOC100128542
SETP14	LOC100128685
SETP19	LOC100128790
SETP2	LOC100128803
SETP22	LOC100128822
SF3A2	LOC100128909
SFR1	LOC100129009
SFTPA2	LOC100129100
SGCG	LOC100129148
SH3BP5	LOC100129321
SH3BP5L	LOC100129480
SH3RF2	LOC100129601
SHF	LOC100129664
SHOC2	LOC100129667
SIGLEC15	LOC100129716
SIL1	LOC100129772
SIPA1L1	LOC100129830
SIPA1L3	LOC100129854
SIRPB2	LOC100129858
SIRT1	LOC100129923
SIRT4	LOC100129961
SKA3	LOC100129989
SKOR2	LOC100130018
SKP1	LOC100130039
SLAIN1	LOC100130137
SLC10A4	LOC100130301
SLC11A2	LOC100130331
SLC12A9	LOC100130466
SLC17A5	LOC100130480
SLC17A6	LOC100130485
SLC22A20	LOC100130487
SLC22A7	LOC100130522
SLC25A17	LOC100130618
SLC25A20	LOC100130691
SLC25A28	LOC100130801
SLC25A33	LOC100130890
SLC25A40	LOC100130923
SLC25A5P5	LOC100130963
SLC27A6	LOC100131013
SLC28A1	LOC100131060
SLC30A5	LOC100131234
SLC31A1P1	LOC100131257

SLC33A1	LOC100131320
SLC35D2	LOC100131528
SLC35E1P1	LOC100131842
SLC35G1	LOC100132080
SLC36A1	LOC100132146
SLC37A2	LOC100132215
SLC38A2	LOC100132242
SLC39A1	LOC100132247
SLC39A6	LOC100132310
SLC43A1	LOC100132352
SLC45A3	LOC100132354
SLC47A2	LOC100132482
SLC4A8	LOC100132507
SLC5A1	LOC100132524
SLC6A11	LOC100132698
SLC7A1	LOC100132733
SLC7A2	LOC100132735
SLC8A1	LOC100132762
SLC9A3R1	LOC100132781
SLCO2A1	LOC100132858
SLFN11	LOC100132891
SLIRP	LOC100132909
SLITRK3	LOC100133005
SLMO2	LOC100133007
SLMO2-ATP5E	LOC100133047
SLN	LOC100133091
SMAD5	LOC100133097
SMAD9	LOC100133112
SMARCC2	LOC100133203
SMARCD3	LOC100133317
SMARCE1	LOC100133669
SMG6	LOC100144602
SMNP	LOC100188947
SMO	LOC100192378
SMOC2	LOC100192389
SMPDL3B	LOC100216545
SMR3B	LOC100216546
SMTNL1	LOC100270648
SNAR-I	LOC100271832
SNCAIP	LOC100287072
SNHG13	LOC100287225
SNHG3	LOC100287313
SNHG5	LOC100287505
SNHG6	LOC100287567
SNHG8	LOC100287651

SNORD50B	LOC100287663
SNORD56B	LOC100287708
SNORD59B	LOC100287715
SNORD93	LOC100287718
SNRNP27	LOC100287723
SNRPA1	LOC100287765
SNRPD2P2	LOC100287922
SNRPF	LOC100287944
SNURFL	LOC100288037
SNX24	LOC100288077
SNX25	LOC100288097
SNX32	LOC100288181
SNX8	LOC100288268
SOCS2	LOC100288283
SORD	LOC100288366
SORD2	LOC100288428
SOX9	LOC100288449
SP100	LOC100288470
SP110	LOC100288524
SP140	LOC100288570
SPA17	LOC100288628
SPATA7	LOC100288637
SPC25	LOC100288663
SPDYC	LOC100288708
SPECC1L	LOC100288798
SPESP1	LOC100288844
SPG20	LOC100289211
SPG21	LOC100289350
SPO11	LOC100289351
SPOCD1	LOC100289383
SPPL2A	LOC100289473
SPRED2	LOC100289488
SPRR2E	LOC100289542
SPSB2	LOC100289561
SPTBN2	LOC100289584
SPTLC3	LOC100289607
SQLE	LOC100294406
SQRDL	LOC100302640
SRD5A1	LOC100418719
SRD5A3	LOC100418730
SREK1	LOC100418814
SREK1IP1	LOC100418940
SRFBP1	LOC100418942
SRGN	LOC100419642
SRI	LOC100419763

SRP72	LOC100419772
SRRM1	LOC100419777
SRRM3	LOC100419812
SRSF3	LOC100419824
SRSF6	LOC100419873
SS18	LOC100419877
SSFA2	LOC100419911
SSR3	LOC100420070
SSTR1	LOC100420073
ST13P2	LOC100420215
ST13P4	LOC100420257
ST13P6	LOC100420260
ST14	LOC100420331
ST3GAL3	LOC100420355
ST6GAL2	LOC100420548
ST6GALNAC1	LOC100420742
ST8SIA2	LOC100420743
ST8SIA3	LOC100420787
STARD4	LOC100420853
STAT1	LOC100420879
STELLAR	LOC100420927
STK17B	LOC100421197
STRADBP1	LOC100421267
STT3A	LOC100421363
STX16	LOC100421411
STX16-NPEPL1	LOC100421587
STX3	LOC100421599
STX7	LOC100421633
STXBP4	LOC100421692
SUCLA2	LOC100421751
SUCLA2P2	LOC100422076
SUCLG1	LOC100422265
SUGT1	LOC100422272
SUGT1P1	LOC100422324
SULT1B1	LOC100422352
SULT1C2P1	LOC100422478
SULT2B1	LOC100422527
SUMF2	LOC100422564
SUMO1	LOC100422580
SUN3	LOC100422614
SUPT5H	LOC100423044
SV2A	LOC100462648
SV2B	LOC100499227
SYNE1	LOC100499484
SYNM	LOC100500773

SYT15	LOC100500934
SYTL3	LOC100505498
TAB3P1	LOC100505505
TACR2	LOC100505532
TAF1D	LOC100505549
TAF5L	LOC100505562
TAF9B	LOC100505566
TAGLN3	LOC100505609
TAMM41	LOC100505613
TAS2R16	LOC100505634
TAS2R39	LOC100505650
TAS2R4	LOC100505678
TAS2R5	LOC100505695
TBC1D2	LOC100505718
TBC1D22A	LOC100505736
TBC1D9	LOC100505739
TBCA	LOC100505774
TBCD	LOC100505794
TBKBP1	LOC100505817
TBRG1	LOC100505832
TBX10	LOC100505836
TCEAL3	LOC100505854
TCEAL7	LOC100505862
TCEB1P3	LOC100505872
TCERG1	LOC100505875
TCP1P1	LOC100505902
TDO2	LOC100505933
TDP1	LOC100505938
TDRD9	LOC100505960
TECPR1	LOC100505964
TEKT4P1	LOC100505966
TERF1	LOC100505989
TESK2	LOC100506022
TEX2	LOC100506023
TF	LOC100506025
TFAM	LOC100506049
TGFA	LOC100506070
TGFBR3	LOC100506128
TGM6	LOC100506136
TH1L	LOC100506176
THAP9	LOC100506178
THBS4	LOC100506189
THOC1	LOC100506190
THUMPD1	LOC100506206
THUMPD2	LOC100506207

TIAM2	LOC100506214
TIMD4	LOC100506217
TIMELESS	LOC100506220
TIMM10	LOC100506226
TIMM17A	LOC100506236
TIMMDC1	LOC100506246
TINAG	LOC100506267
TKTL2	LOC100506272
TLR1	LOC100506339
TLR5	LOC100506342
TM4SF18	LOC100506354
TM9SF2	LOC100506368
TMC2	LOC100506379
TMCO4	LOC100506380
TMED10	LOC100506393
TMED11P	LOC100506422
TMED2	LOC100506446
TMEM119	LOC100506462
TMEM128	LOC100506474
TMEM132C	LOC100506487
TMEM140	LOC100506495
TMEM144	LOC100506497
TMEM154	LOC100506516
TMEM176B	LOC100506575
TMEM192	LOC100506586
TMEM207	LOC100506606
TMEM215	LOC100506639
TMEM220	LOC100506652
TMEM229B	LOC100506660
TMEM233	LOC100506682
TMEM31	LOC100506686
TMEM39A	LOC100506687
TMEM53	LOC100506689
TMEM55B	LOC100506700
TMEM57	LOC100506710
TMEM74	LOC100506725
TMEM85	LOC100506762
TMEM99	LOC100506776
TMPRSS11A	LOC100506810
TMPRSS11B	LOC100506831
TMPRSS11CP	LOC100506860
TMPRSS11D	LOC100506869
TMPRSS11E	LOC100506922
TMPRSS11F	LOC100506927
TMPRSS12	LOC100506929

TMPRSS4	LOC100506936
TMTC3	LOC100506939
TMX4	LOC100506941
TNFAIP3	LOC100506980
TNFAIP8	LOC100507053
TNFAIP8L3	LOC100507056
TNFRSF19	LOC100507065
TNFRSF21	LOC100507145
TNFSF11	LOC100507163
TNK2	LOC100507175
TNKS2	LOC100507186
TNNI1	LOC100507193
TNRC6A	LOC100507205
TOMM5	LOC100507217
TOMM7	LOC100507258
TOP3A	LOC100507299
TOR1B	LOC100507332
TPD52L1	LOC100507377
TPH2	LOC100507389
TPM3P2	LOC100507391
TPTE2P1	LOC100507421
TPTE2P6	LOC100507452
TRAF2	LOC100507466
TRAF3IP1	LOC100507474
TRAF6	LOC100507481
TRAM2	LOC100507489
TRAPPC2P1	LOC100507495
TRAT1	LOC100507500
TRBV24OR9-2	LOC100507508
TREM1	LOC100507521
TRGC1	LOC100507531
TRIL	LOC100507540
TRIM23	LOC100507557
TRIM42	LOC100507642
TRIM46	LOC100507650
TRIM49L1	LOC100507651
TRIM59	LOC100508736
TRIM62	LOC100509105
TRIM63	LOC100526771
TRIM67	LOC100533635
TRIP12	LOC100533661
TRMT12	LOC100533664
TRMT1L	LOC100533667
TRMT5	LOC100533677
TRNAA-AGC	LOC100533707

TRNAA30	LOC100533719
TRNAA38	LOC100533853
TRNAA4	LOC100616530
TRNAC18	LOC100628307
TRNAC28	LOC100630923
TRNAE7	LOC100631247
TRNAG18	LOC100631269
TRNAI-AAU	LOC100652735
TRNAK11	LOC100652747
TRNAK34	LOC100652753
TRNAL12	LOC100652757
TRNAL15	LOC100652761
TRNAL46P	LOC100652766
TRNAL47P	LOC100652767
TRNAM15	LOC100652770
TRNAN2	LOC100652787
TRNAN29	LOC100652789
TRNAN36P	LOC100652796
TRNAP17	LOC100652804
TRNAQ2	LOC100652810
TRNAQ38P	LOC100652812
TRNAQ54P	LOC100652823
TRNAQ8	LOC100652827
TRNAR14	LOC100652843
TRNAR24	LOC100652845
TRNAR27	LOC100652857
TRNAR6	LOC100652860
TRNAS17	LOC100652863
TRNAS31P	LOC100652887
TRNAV24	LOC100652891
TRNAV25	LOC100652893
TRPC7	LOC100652900
TRPM8	LOC100652903
TRPV5	LOC100652913
TSKU	LOC100652914
TSPAN14	LOC100652919
TSPAN15	LOC100652924
TSPAN2	LOC100652927
TSPAN33	LOC100652937
TSPY23P	LOC100652946
TSPYL5	LOC100652953
TSSC1	LOC100652971
TTC12	LOC100652973
TTC21A	LOC100652979
TTC28-AS1	LOC100653008

TTC7A	LOC100653017
TTC8	LOC124685
TTI1	LOC126860
TTK	LOC131185
TUBA1B	LOC145783
TUBB	LOC145820
TUBB8	LOC145837
TUBD1	LOC150568
TUBE1	LOC150622
TUBGCP4	LOC151760
TUFMP1	LOC152225
TWIST1	LOC152586
TWISTNB	LOC153469
TXK	LOC154092
TXNDC8	LOC154761
TYRP1	LOC154822
UAP1	LOC154860
UBASH3B	LOC219347
UBB	LOC253573
UBE2B	LOC254128
UBE2D1	LOC255025
UBE2F	LOC255411
UBE2F-SCLY	LOC256374
UBE2HP1	LOC260334
UBE2Q1	LOC283038
UBE2Q2P1	LOC283177
UBL3	LOC283194
UBN1	LOC283547
UCHL3	LOC283553
UCK1	LOC283683
UCP2	LOC283711
UFL1	LOC284100
UGP2	LOC284294
UGT2A1	LOC284561
UGT2A2	LOC284576
UGT2A3	LOC285141
UGT2B24P	LOC285205
UGT2B25P	LOC285419
UGT2B28	LOC285423
UIMC1	LOC285501
UNC5D	LOC285577
UPP1	LOC285692
UQCRBP3	LOC285696
UQCRH	LOC285768
UQCRHP4	LOC285889

URGCP	LOC285954
URGCP-MRPS24	LOC285965
USP1	LOC285972
USP10	LOC286135
USP20	LOC286149
USP30	LOC286178
USP38	LOC286186
USP9Y	LOC286189
USP9YP8	LOC286370
USPL1	LOC338591
UST	LOC338799
UTP18	LOC338862
UTP23	LOC339400
VAT1L	LOC339529
VDAC2P2	LOC339788
VIM	LOC339822
VN1R108P	LOC339874
VN1R16P	LOC339894
VN1R25P	LOC339926
VN1R48P	LOC339975
VN1R51P	LOC340094
VN1R63P	LOC340113
VN2R1P	LOC340268
VPS36	LOC340508
VPS37D	LOC340515
VPS53	LOC343165
VRK1	LOC343508
VRK2	LOC344595
VSTM2A	LOC344887
VTRNA2-1	LOC347193
VWA3A	LOC347281
VWA5A	LOC348840
VWA5B1	LOC359819
WASF1	LOC375190
WBP11P1	LOC387895
WDFY1	LOC388734
WDR16	LOC388996
WDR20	LOC389676
WDR27	LOC389705
WDR31	LOC389765
WDR36	LOC390101
WDR66	LOC390600
WDR75	LOC391040
WDR85	LOC391174
WDR96	LOC391470

WFDC9	LOC391472
WHSC1	LOC391718
WNT9A	LOC391742
WRNIP1	LOC391764
WSB1	LOC392285
WTAP	LOC392787
XPO6	LOC399708
YIPF3	LOC400084
YPEL5	LOC400604
YTHDC2	LOC400655
YWHAZP5	LOC400986
YWHAZP6	LOC401068
ZBTB1	LOC401177
ZBTB24	LOC401316
ZBTB8B	LOC401320
ZC3H11B	LOC401679
ZC3H13	LOC401703
ZC3H14	LOC401805
ZC3H3	LOC401980
ZC3H8	LOC402076
ZC3HAV1L	LOC440040
ZCCHC24	LOC440173
ZCCHC8	LOC440313
ZCRB1	LOC440434
ZCWPW1	LOC440704
ZDBF2	LOC440925
ZDHHC20	LOC440970
ZDHHC20P4	LOC441242
ZDHHC5	LOC441461
ZFAND1	LOC441736
ZFYVE20	LOC441806
ZFYVE28	LOC441899
ZG16	LOC441907
ZIC4	LOC442006
ZMIZ2	LOC442028
ZNF117	LOC442063
ZNF124	LOC442131
ZNF22	LOC442161
ZNF226	LOC442239
ZNF248	LOC442320
ZNF25	LOC442421
ZNF273	LOC442497
ZNF281	LOC442517
ZNF366	LOC442707
ZNF384	LOC442710

ZNF510	LOC493754
ZNF563	LOC554201
ZNF577	LOC613126
ZNF596	LOC619207
ZNF608	LOC63930
ZNF620	LOC641298
ZNF626	LOC641364
ZNF648	LOC641365
ZNF669	LOC641695
ZNF672	LOC642236
ZNF708	LOC642355
ZNF727	LOC642414
ZNF736P1Y	LOC642441
ZNF738	LOC642496
ZNF761	LOC642550
ZNF774	LOC642791
ZNF775	LOC642846
ZNF777	LOC642924
ZNF785	LOC643255
ZNF789	LOC643339
ZNF815	LOC643373
ZNF839P1	LOC643542
ZNF841	LOC643579
ZNF847P	LOC643582
ZNF92	LOC643634
ZNHIT3	LOC643650
ZNRF2P1	LOC643723
ZPBP2	LOC643770
ZSCAN12	LOC643827
ZSCAN21	LOC643962
ZWILCH	LOC643997
	LOC644277
	LOC644495
	LOC644616
	LOC644632
	LOC644661
	LOC644794
	LOC644838
	LOC645166
	LOC645206
	LOC645314
	LOC645324
	LOC645434
	LOC645482
	LOC645749

LOC645877
LOC645954
LOC646012
LOC646030
LOC646103
LOC646120
LOC646203
LOC646213
LOC646218
LOC646329
LOC646360
LOC646616
LOC646639
LOC646745
LOC646762
LOC646828
LOC646999
LOC647051
LOC647107
LOC647145
LOC647589
LOC647946
LOC648262
LOC648532
LOC648809
LOC648947
LOC649395
LOC653458
LOC653513
LOC653653
LOC654342
LOC723809
LOC727677
LOC727751
LOC727805
LOC727924
LOC727982
LOC728012
LOC728034
LOC728056
LOC728065
LOC728175
LOC728323
LOC728377
LOC728558

LOC728611
LOC728730
LOC728755
LOC728875
LOC728989
LOC729126
LOC729156
LOC729178
LOC729222
LOC729305
LOC729409
LOC729506
LOC729852
LOC729862
LOC729915
LOC729941
LOC729950
LOC729974
LOC730021
LOC730091
LOC730102
LOC730190
LOC730974
LOC91948
LOH12CR1
LONP2
LONRF2
LONRF2P1
LONRF2P2
LPAR1
LPCAT3
LPGAT1
LPHN3
LPIN1
LPIN2
LPP
LPPR1
LPXN
LRBA
LRCH1
LRCH3
LRFN5
LRGUK
LRIG1
LRIG3

LRMP
LRP11
LRP12
LRP1B
LRP2
LRP4
LRPPRC
LRR1
LRRC1
LRRC16A
LRRC17
LRRC2
LRRC28
LRRC31
LRRC37B
LRRC3B
LRRC49
LRRC4C
LRRC55
LRRC6
LRRC67
LRRC69
LRRC7
LRRC9
LRRCC1
LRRD1
LRRFIP1
LRRFIP2
LRRIQ1
LRRK1
LRRK2
LRRN1
LRRTM3
LRRTM4
LSAMP
LSM14A
LSM5
LTA4H
LTBP1
LTN1
LUC7L2
LUC7L3
LUZP2
LUZP6
LY75

LY75-CD302
LY86-AS1
LY96
LYN
LYPD6
LYPD6B
LYPLA1
LYST
LYZ
LYZL1
LYZL2
LZTFL1
M1
M6PR
MACC1
MACROD2
MAD1L1
MAD2L1BP
MAGI1
MAGI1-IT1
MAGI2
MAGOHB
MAGT1
MAK
MAL2
MALAT1
MALT1
MAMDC2
MAML2
MAML3
MAN1A2
MAN2A1
MAOA
MAP2
MAP2K4
MAP2K5
MAP3K1
MAP3K13
MAP3K14
MAP3K4
MAP3K5
MAP3K7
MAP4
MAP4K4
MAP4K5

MAP6
MAP7
MAPK10
MAPK4
MAPK6
MAPK8
MAPKAP1
MAPKAPK2
MAPKAPK5
MAPRE2
MAPRE3
MAPT
MARCH1
MARCH11
MARCH5
MARCH6
MARCH7
MARCH8
MARCO
MARK1
MARK2
MARK2P10
MARK2P9
MARS2
MASP1
MAST2
MAST4
MASTL
MAT1A
MATN2
MATR3
MB21D2
MBD2
MBD5
MBIP
MBNL1
MBNL2
MBOAT2
MBP
MBTD1
MC2R
MC5R
MCART1
MCC
MCCC1

MCCC2
MCF2L2
MCHR2
MCL1
MCM4
MCM7
MCM8
MCM9
MCTP1
MCTP2
MCU
MDC1
MDFIC
MDGA2
MDM2
MDM4
MDN1
ME1
ME2
ME3
MEA1
MEAF6
MECOM
MED10
MED12L
MED13
MED13L
MED17
MED23
MEF2A
MEF2C
MEGF10
MEGF9
MEIS2
MELK
MEMO1
MEOX2
MEP1A
MERTK
MET
METAP1D
METAP2
METTL10
METTL14
METTL15

METTL15P1
METTL20
METTL2B
METTL4
METTL5
METTL7B
METTL8
METTL9
MEX3C
MFN1
MFSD1
MFSD4
MFSD6
MFSD9
MGA
MGAM
MGAT4A
MGAT4C
MGAT5
MGC14436
MGC21881
MGLL
MGMT
MGST1
MGST2
MIA2
MIA3
MIB1
MICU1
MIER1
MINA
MIOS
MIPEP
MIPEPP1
MIPOL1
MIR100HG
MIR1279
MIR1302-4
MIR181A2HG
MIR30D
MIR3174
MIR3910-2
MIR4454
MIR4455
MIR4456

MIR4471
MIR4477A
MIR4477B
MIR4662A
MIR4698
MIR607
MIRLET7BHG
MIS18BP1
MITF
MKI67
MKL1
MKL2
MKLN1
MKRN1
MKRN2
MKX
MLF1
MLH1
MLIP
MLK7-AS1
MLL
MLL3
MLL5
MLLT10
MLLT3
MLLT4
MLPH
MMAA
MMADHC
MME
MMP13
MMP16
MMP20
MMS22L
MNAT1
MND1
MNX1
MOB3B
MOB4
MOBP
MON2
MORC1
MORC3
MORF4L1
MORN5

MOSC2
MOXD1
MPDZ
MPHOSPH9
MPP5
MPP6
MPP7
MPPED2
MRE11A
MRO
MRPL1
MRPL14
MRPL15
MRPL35P2
MRPL42
MRPL47
MRPL48
MRPL48P1
MRPS16P1
MRPS18A
MRPS21
MRPS28
MRPS33
MRPS35
MRPS9
MRRF
MRS2
MS4A13
MS4A4E
MSH3
MSH4
MSL1
MSL2
MSL3P1
MSMP
MSR1
MSRB3
MT1P1
MTA3
MTBP
MTCH2
MTDH
MTFR1
MTHFD1L
MTHFD2L

MTHFS
MTMR10
MTMR12
MTMR2
MTMR3
MTPAP
MTR
MTSS1
MTX2
MUC13
MUSK
MUT
MUTED-TXNDC5
MXI1
MYB
MYBL1
MYBPC1
MYC
MYCBP2
MYEF2
MYH10
MYH15
MYH4
MYH7
MYH8
MYLK
MYLK-AS1
MYLK4
MYO10
MYO16
MYO1B
MYO1D
MYO1E
MYO3A
MYO3B
MYO5A
MYO5B
MYO6
MYO7B
MYO9A
MYOCD
MYOF
MYOM1
MYPN
MYRIP

MYT1
MYT1L
N4BP2
N4BP2L2
NAA16
NAA25
NAA35
NAALAD2
NAALADL2
NAB1
NACAP1
NADKD1
NALCN
NAMPTL
NAP1L1
NAPEPLD
NARG2
NARS2
NAV1
NAV2
NAV3
NBAS
NBEA
NBEAL1
NBEAP1
NBEAP4
NBN
NBPF1
NBPF10
NBPF14
NBPF15
NBPF17P
NBPF7
NCALD
NCAM1
NCAM2
NCAPD2
NCAPD3
NCAPG2
NCAPH
NCBP1
NCEH1
NCF2
NCK1
NCKAP1

NCKAP1L
NCKAP5
NCOA1
NCOA2
NCOA3
NCOA7
NCOR1
NCOR1P2
NDC80
NDRG1
NDST3
NDST4
NDUFA12
NDUFA5
NDUFA9
NDUFAF2
NDUFB2
NDUFB8P3
NDUFC2
NDUFC2-KCTD14
NDUFS1
NDUFS4
NEAT1
NEB
NEBL
NECAB1
NEDD4
NEDD4L
NEDD9
NEGR1
NEK1
NEK10
NEK11
NEK2
NEK4
NEK7
NEK9
NELL1
NELL2
NEMF
NEO1
NET1
NETO1
NEURL
NEUROD1

NF1
NF1P6
NF1P8
NFASC
NFATC2
NFE2L2
NFE2L3
NFIA
NFIB
NFIL3
NFIX
NFKBIZ
NFX1
NFXL1
NFYB
NGF
NGLY1
NHP2P2
NHSL1
NID1
NIM1
NIN
NIPAL2
NIPBL
NKAIN1P1
NKAIN2
NKAIN3
NKIRAS1
NKTR
NLGN1
NLK
NLRC4
NLRP3
NME9
NMNAT2
NMNAT3
NMT2
NMU
NNMT
NNT
NOD1
NOL10
NOL4
NOL6
NOL8

NOM1
NOSTRIN
NOTCH2
NOTCH2NL
NOVA1
NOX4
NOX5
NPAS2
NPAS3
NPAT
NPC1L1
NPEPPS
NPHP1
NPHP3
NPHP3-ACAD11
NPHP3-AS1
NPHS2
NPL
NPM1P18
NPR2
NPR3
NPSR1
NPTN
NPTX2
NPVF
NPY6R
NR1D1
NR1D2
NR1H4
NR1H5P
NR2C1
NR2C2
NR2F2
NR3C1
NR3C2
NR4A2
NR5A2
NR6A1
NRBP2
NRCAM
NRD1
NRF1
NRG1
NRG3
NRG4

NRIP1
NRK
NRM
NRN1
NRP1
NRP2
NRSN1
NRXN1
NRXN3
NSD1
NSF
NSL1
NSMAF
NSMCE2
NSRP1
NSUN2
NSUN6
NSUN7
NT5C1B
NT5C2
NT5C3
NT5C3L
NT5DC1
NT5DC3
NTM
NTN4
NTNG2
NTPCR
NTRK2
NTRK3
NTS
NUB1
NUBPL
NUCB2
NUCKS1
NUDCD3
NUDT13
NUDT3
NUDT4
NUDT4P1
NUFIP2
NUMA1
NUMB
NUP107
NUP133

NUP153
NUP155
NUP160
NUP188
NUP205
NUP210
NUP210L
NUP214
NUP35
NUPL2
NUSAP1
NVL
NWD1
NXF1
NXPH1
NXPH2
OAT
OBFC1
OBP2A
OC90
OCIAD2P1
ODC1
ODF1
ODZ2
ODZ3
ODZ4
OFCC1
OGDH
OLA1
OMA1
ONECUT2
OPA1
OPCML
OPHN1
OPN1SW
OPN3
OPTN
OR10AD1
OR11G1P
OR11H2
OR13C3
OR13G1
OR14I1
OR1L4
OR1L8

OR1S1
OR2A1
OR2A12
OR2A20P
OR2A5
OR2B11
OR2L13
OR2M5
OR2T12
OR2T27
OR2T29
OR2T4
OR2T5
OR2T7
OR2T8
OR4F28P
OR4G6P
OR4K15
OR4K5
OR4K8P
OR4Q2
OR4Q3
OR5AP2
OR5AR1
OR5B12
OR5BQ1P
OR5H8P
OR5J1P
OR6B1
OR6C68
OR6C70
OR6C76
OR6L2P
OR6U2P
OR6V1
OR7E108P
OR7E31P
OR9A1P
OR9A2
OR9A3P
OR9K1P
ORC2
ORC4
ORC5
OSBPL10

OSBPL11
OSBPL1A
OSBPL3
OSBPL6
OSBPL8
OSBPL9
OSGIN2
OSMR
OSR1
OSR2
OSTF1
OSTN
OTOGL
OTUD7B
OVCH1
OVOS
OVOS2
OXCT1
OXR1
OXSR1
P2RY12
P2RY14
P4HA1
P4HA3
PABPC1
PABPC1P2
PACRG
PACS1
PACSIN1
PAFAH1B2
PAG1
PAH
PAICSP1
PAIP1
PAK1
PAK2
PAK6
PAK7
PALLD
PALM2
PALM2-AKAP2
PAM
PAMR1
PAN3
PANX1

PAPD4
PAPD5
PAPD7
PAPPA
PAPPA2
PAPSS1
PAQR5
PAQR9
PARD3
PARD3B
PARD6G
PARG
PARK2
PARL
PARM1
PARN
PARP10
PARP12
PARP14
PARP15
PARP2
PARP4P1
PARP8
PART1
PATE2
PATL1
PAWR
PAX5
PAX9
PBLD
PBRM1
PBX3
PCA3
PCBP2
PCCA
PCCB
PCDH15
PCDH7
PCDH9
PCDHA@
PCDHA1
PCDHA10
PCDHA11
PCDHA12
PCDHA13

PCDHA2
PCDHA3
PCDHA4
PCDHA5
PCDHA6
PCDHA7
PCDHA8
PCDHA9
PCDHB@
PCDHB7
PCDHGA1
PCDHGA2
PCDHGA3
PCDHGA4
PCDHGA5
PCDHGB1
PCDHGB2
PCDP1
PCF11
PCGF5
PCLO
PCMT1
PCMTD1
PCMTD1P2
PCMTD2
PCNP
PCNX
PCNXL2
PCOLCE2
PCSK5
PDCD1LG2
PDCD4
PDCD6
PDCD6IP
PDCL
PDE10A
PDE11A
PDE1A
PDE1C
PDE3A
PDE4B
PDE4D
PDE4DIP
PDE7A
PDE7B

PDE8B
PDE9A
PDGFC
PDGFD
PDHX
PDIA4
PDIA5
PDIA6
PDLIM1
PDLIM5
PDP1
PDS5A
PDS5B
PDSS1
PDSS2
PDZD2
PDZK1
PDZRN3
PDZRN4
PEAK1
PEG10
PELI1
PERP
PEX1
PEX5
PEX5L
PEX6
PEX7
PFDN1
PGAM1P4
PGAM1P5
PGAP1
PGBD2
PGCP
PGK1
PGM2L1
PGM3
PGM5
PGR
PHACTR1
PHACTR2
PHACTR3
PHACTR4
PHBP7
PHC3

PHF12
PHF14
PHF2
PHF21A
PHF3
PHGDH
PHIP
PHKB
PHLDB2
PHLPP1
PHOSPHO2-KLHL23
PHTF2
PHYHIPL
PI15
PIAS1
PIAS2
PIBF1
PICALM
PIEZO2
PIGB
PIGK
PIGL
PIGN
PIGR
PIK3AP1
PIK3C2A
PIK3C2B
PIK3C2G
PIK3C3
PIK3CA
PIK3CB
PIK3CG
PIK3R1
PIK3R3
PIK3R4
PIKFYVE
PILRA
PION
PIP4K2A
PIP5K1A
PIP5K1B
PIP5K1P2
PITPNC1
PIWIL4
PJA2

PKD1L1
PKD2
PKHD1
PKHD1L1
PKIA
PKIB
PKP1
PKP2
PKP4
PLA2G12B
PLA2G16
PLA2G4A
PLA2G4D
PLA2R1
PLAA
PLAG1
PLAGL1
PLB1
PLCB1
PLCB4
PLCE1
PLCH1
PLCL1
PLCL2
PLCXD2
PLCXD3
PLCZ1
PLD1
PLD5
PLDN
PLEKHA5
PLEKHA6
PLEKHA7
PLEKHA8
PLEKHA8P1
PLEKHB2
PLEKHF2
PLEKHG1
PLEKHG4B
PLEKHH1
PLEKHH2
PLEKHM3
PLOD2
PLS1
PLSCR1

PLSCR2
PLSCR4
PLSCR5
PLXDC2
PLXNA4
PM20D2
PMEPA1
PMP22
PMPCB
PMS1
PMS2
PNISR
PNN
PNPLA7
PNPLA8
PNPT1
POC1B
POGLUT1
POGZ
POLD2
POLH
POLI
POLN
POLQ
POLR1B
POLR1D
POLR3A
POLR3G
POLR3GL
POM121
PON1
PON3
POP1
POR
POT1
POTED
POTEE
POTEKP
POU5F1P2
POU6F2
PP2D1
PPA1
PPA2
PPAP2A
PPAP2B

PPAPDC1B
PPARG
PPFIA2
PPFIBP1
PPHLN1
PPIAL4D
PPIAL4E
PPIG
PPIL4
PPIL6
PPM1B
PPM1H
PPM1L
PPME1
PPP1CB
PPP1CC
PPP1R10
PPP1R12A
PPP1R12B
PPP1R13B
PPP1R15B
PPP1R16A
PPP1R1C
PPP1R3A
PPP1R3C
PPP1R9A
PPP2R1B
PPP2R2B
PPP2R3A
PPP2R3C
PPP2R5A
PPP2R5D
PPP2R5E
PPP3CA
PPP3CB
PPP3R1
PPP4R1
PPP4R1L
PPP4R2
PPP6C
PPP6R3
PPPDE1
PPTC7
PPYR1
PQLC1

PRC1
PRDM10
PRDM14
PRDX3P3
PRELID2
PREPL
PREX2
PRG4
PRICKLE1
PRICKLE2
PRIM1
PRIM2
PRKAA1
PRKAA2
PRKAG2
PRKAR1B
PRKAR2A
PRKAR2B
PRKCA
PRKCE
PRKCH
PRKCI
PRKD1
PRKD3
PRKDC
PRKG1
PRKRIRP5
PRLR
PRMT10
PRMT3
PROC
PROS1
PROX1
PRPF3
PRPF39
PRPF4
PRPF40B
PRPF6
PRR13
PRR16
PRRC1
PRRC2B
PRRG4
PRSS12
PRSS35

PRSS38
PRSS42
PRTFDC1
PRTG
PRUNE2
PSD3
PSD4
PSIP1
PSKH2
PSMA3
PSMA7P
PSMA8
PSMB7
PSMC2
PSMC6P1
PSMD1
PSMD11
PSMD14
PSMD6
PSME4
PSMG2
PSORS1C3
PSPC1
PSTK
PSTPIP2
PTAR1
PTCH1
PTDSS1
PTER
PTGER2
PTGER3
PTGER4
PTGES2
PTGES3
PTGFRN
PTGR1
PTGR2
PTGS2
PTH2R
PTK2
PTK7
PTN
PTPDC1
PTPLA
PTPLAD1

PTPLAD2
PTPLB
PTPN12
PTPN13
PTPN14
PTPN18
PTPN2
PTPN21
PTPN3
PTPN4
PTPN5
PTPRB
PTPRC
PTPRD
PTPRE
PTPRG
PTPRJ
PTPRK
PTPRM
PTPRN2
PTPRO
PTPRQ
PTPRR
PTPRZ1
PUM1
PUM2
PURA
PUS10
PUS7
PUS7L
PVT1
PXDN
PXDNL
PXK
PYGL
PZP
QKI
QSER1
QSOX1
QSOX2
R3HDM1
R3HDM2
RAB10
RAB11A
RAB11FIP4

RAB14
RAB18
RAB1C
RAB21
RAB22A
RAB23
RAB27A
RAB27B
RAB2A
RAB2B
RAB30
RAB31
RAB3B
RAB3C
RAB3GAP1
RAB3GAP2
RAB3IP
RAB4A
RAB5A
RAB6A
RAB7A
RAB8B
RABEP1
RABGAP1
RABGAP1L
RABGEF1
RABL3
RABL5
RAC1
RAC1P6
RAD17
RAD18
RAD21
RAD23B
RAD51
RAD51AP1
RAD51B
RAD51L3-RFFL
RAD54B
RAD54L2
RAD9B
RAF1
RAI14
RALA
RALBP1

RALGAPA1
RALGAPA2
RALGAPB
RALGPS1
RALGPS2
RALYL
RAMP1
RANBP17
RANBP2
RANBP3L
RANBP9
RAP1B
RAPGEF1
RAPGEF2
RAPGEF4
RAPGEF5
RAPGEF6
RAPH1
RARB
RARRES2
RASA1
RASA2
RASAL2
RASEF
RASGRF2
RASGRP1
RASSF3
RASSF6
RASSF8
RB1
RB1CC1
RBAK
RBAK-LOC389458
RBBP4P4
RBBP5
RBBP8
RBFOX2
RBKS
RBM12B
RBM14-RBM4
RBM17
RBM20
RBM25
RBM33
RBM34

RBM44
RBM47
RBM6
RBM8A
RBMS1
RBMS2
RBMS3
RBP2
RBPMS
RC3H1
RC3H2
RCAN2
RCBTB1
RCL1
RCOR3
RDH10
RDX
RECK
REEP3
REL
RELN
REPIN1
REPS1
RERE
RERG
REV1
REV3L
REXO1L1
RFC2
RFC3
RFFL
RFK
RFT1
RFTN1
RFTN1P1
RFTN2
RFWD2
RFX3
RFX4
RFX6
RFX7
RG9MTD1
RG9MTD3
RGL1
RGMB

RGNEF
RGS20
RGS22
RGS3
RGS7
RGS8
RGSL1
RHAG
RHBDD1
RHEB
RHOA
RHOBTB1
RHOH
RHOT1
RHOU
RHPN2P1
RIC8B
RICTOR
RIF1
RIMKLB
RIMS1
RIMS2
RIOK3
RIPK2
RIT2
RMI1
RMST
RN7SL7P
RNASE6
RNASEH1
RND3
RNF10
RNF103-VPS24
RNF111
RNF115
RNF11P1
RNF125
RNF13
RNF138
RNF144A
RNF144B
RNF145
RNF146
RNF149
RNF150

RNF152
RNF165
RNF168
RNF169
RNF17
RNF175
RNF180
RNF19A
RNF2
RNF214
RNF216
RNF216P1
RNF24
RNF32
RNF38
RNF41
RNGTT
RNLS
RNMT
RNU1-4
RNU1-5
RNU6ATAC5P
RNU7-54P
RNU7-82P
ROBO1
ROBO2
ROCK1
ROCK1P1
ROCK2
ROD1
ROR1
ROR2
RORA
RORB
ROS1
RP9
RP9P
RPA3
RPAP3
RPGRIP1
RPL10P11
RPL12P10
RPL12P35
RPL13AP18
RPL13P11

RPL21P102
RPL21P128
RPL21P31
RPL21P61
RPL21P75
RPL21P76
RPL22L1
RPL22P20
RPL22P7
RPL23AP21
RPL23AP25
RPL23AP27
RPL23AP7
RPL23P8
RPL24P7
RPL26P23
RPL27P10
RPL29P16
RPL29P19
RPL29P8
RPL30
RPL31P10
RPL31P9
RPL32P20
RPL32P30
RPL32P8
RPL34P14
RPL34P18
RPL34P31
RPL35AP19
RPL35AP8
RPL35P2
RPL35P4
RPL36AP28
RPL39P11
RPL39P2
RPL39P27
RPL3P11
RPL3P9
RPL5P3
RPL6P8
RPL7AP41
RPL7AP54
RPL7P16
RPL7P28

RPL7P37
RPL7P40
RPL7P6
RPL9P14
RPN1
RPP30
RPRD1A
RPRD2
RPRM
RPRML
RPS10
RPS10-NUDT3
RPS10P13
RPS10P27
RPS11P3
RPS12P11
RPS12P17
RPS15P2
RPS16P2
RPS16P4
RPS17P12
RPS20P23
RPS20P25
RPS24
RPS24P10
RPS24P15
RPS25P2
RPS25P4
RPS25P7
RPS26P32
RPS26P6
RPS27AP11
RPS27AP6
RPS27P16
RPS27P20
RPS29
RPS2P33
RPS2P34
RPS2P40
RPS3AP13
RPS3AP47
RPS4XP1
RPS4XP12
RPS5P5
RPS5P6

RPS6KA5
RPS6KB1
RPS6KC1
RPS6P10
RPS6P13
RPS7
RPSAP12
RPSAP47
RPSAP57
RPTOR
RREB1
RRM2B
RSBN1L
RSF1
RSL24D1P5
RSPH9
RSPO2
RSPO3
RSRC1
RSU1
RTKN2
RTN1
RTN3
RTN4
RTTN
RUFY2
RUNDC3B
RUNX1T1
RUNX2
RUSC2
RUVBL1
RWDD1P3
RXFP1
RYK
RYR2
RYR3
S100A10
S100A11
SAAL1
SAMD12
SAMD12-AS1
SAMD4A
SAMD7
SAMD8
SAR1B

SARNP
SART3
SATB1
SATB2
SAV1
SBF2
SBNO1
SC5DL
SCAF11
SCAF4
SCAF8
SCAI
SCAMP1
SCAP
SCAPER
SCCPDH
SCEL
SCFD1
SCG5
SCHIP1
SCIN
SCLT1
SCMH1
SCN10A
SCN1A
SCN3A
SCN5A
SCN7A
SCN8A
SCN9A
SCOC
SCRN1
SCTR
SCYL2
SDC1
SDC2
SDCBP
SDCCAG8
SDHA
SDK1
SEC11A
SEC11C
SEC16A
SEC16B
SEC22A

SEC22B
SEC22C
SEC23A
SEC24A
SEC24B
SEC61G
SEC63
SECISBP2L
SEH1L
SEMA3A
SEMA3C
SEMA3D
SEMA3E
SEMA4D
SEMA5A
SEMA6A
SEMA6D
SENP1
SENP5
SENP6
SENP7
SENP8
SEPHS1P5
SEPP1
SEPT10
SEPT14
SEPT7
SEPT7P2
SEPT7P3
SERAC1
SERGEF
SERP2
SERPINB7
SERPINI1
SESN1
SESTD1
SET
SETBP1
SETD2
SETD5
SETD7
SETDB1
SETX
SF3B1
SF3B5

SFMBT1
SFMBT2
SFSWAP
SFTA3
SGCD
SGCE
SGIP1
SGK1
SGK3
SGMS1
SGMS2
SGOL2
SGPL1
SH2D4B
SH3D19
SH3GL2
SH3GL3
SH3PXD2A
SH3RF1
SH3RF3
SH3TC2
SH3YL1
SHANK2
SHANK3
SHC3
SHC4
SHCBP1L
SHFM1
SHISA6
SHPRH
SHQ1
SHROOM3
SHROOM4
SI
SIAH2
SIDT1
SIK2
SIK3
SIM2
SIN3A
SIPA1L2
SIX1
SKA1
SKAP1
SKAP2

SKIL
SKIV2L2
SKP2
SLA
SLAIN2
SLC10A7
SLC12A1
SLC12A2
SLC12A6
SLC12A7
SLC12A8
SLC13A1
SLC13A4
SLC14A2
SLC15A2
SLC15A5
SLC16A10
SLC16A7
SLC16A9
SLC17A1
SLC17A2
SLC17A8
SLC1A1
SLC1A2
SLC1A3
SLC20A1
SLC22A10
SLC22A15
SLC22A16
SLC22A2
SLC22A23
SLC22A24
SLC22A25
SLC22A3
SLC22A9
SLC23A4P
SLC24A2
SLC24A3
SLC24A5
SLC25A12
SLC25A13
SLC25A16
SLC25A21
SLC25A25
SLC25A26

SLC25A3
SLC25A32
SLC25A36
SLC26A3
SLC26A4
SLC26A5
SLC26A7
SLC27A2
SLC28A3
SLC29A1
SLC2A12
SLC2A13
SLC30A1
SLC30A10
SLC30A4
SLC30A6
SLC30A8
SLC30A9
SLC31A1
SLC35B4
SLC35D1
SLC35E3
SLC35F1
SLC35F2
SLC35F3
SLC35F4
SLC35F5
SLC36A4
SLC37A1
SLC37A3
SLC38A1
SLC38A11
SLC38A4
SLC38A6
SLC38A9
SLC39A10
SLC39A12
SLC39A8
SLC39A9
SLC3A1
SLC3A2
SLC41A2
SLC41A3
SLC44A1
SLC44A5

SLC45A2
SLC4A10
SLC4A1AP
SLC4A4
SLC4A7
SLC5A12
SLC5A8
SLC6A15
SLC6A18
SLC6A19
SLC6A3
SLC7A11
SLC7A14
SLC7A8
SLC8A3
SLC9A10
SLC9A11
SLC9A2
SLC9A3
SLC9A4
SLC9A7
SLC9A8
SLC9A9
SLC9B1
SLC9B2
SLCO1A2
SLCO1B1
SLCO1B3
SLCO1B7
SLCO1C1
SLCO4C1
SLCO5A1
SLCO6A1
SLIT2
SLIT3
SLK
SLMAP
SLTM
SMAD1
SMAD2
SMAD3
SMAD4
SMAD7
SMAP1
SMAP2

SMARCA2
SMARCAD1
SMARCC1
SMC1B
SMC2
SMC4
SMC5
SMC6
SMCHD1
SMEK1
SMG7
SMNDC1
SMOC1
SMU1
SMURF1
SMURF2
SMYD2
SMYD3
SNAP23
SNAP91
SNAPC3
SNAR-B1
SNCA
SND1
SNHG1
SNORD3A
SNRK
SNRNP40
SNRPBP1
SNRPC
SNRPD1
SNRPE
SNRPGP2
SNRPN
SNTB1
SNTG1
SNTG2
SNTN
SNX1
SNX10
SNX13
SNX14
SNX16
SNX18P10
SNX18P2

SNX18P21
SNX19
SNX2
SNX27
SNX3
SNX30
SNX31
SNX4
SNX5
SNX6
SNX9
SOAT1
SOAT2
SOCS4
SOCS5
SOCS6
SON
SORBS1
SORBS2
SORCS1
SORCS3
SORL1
SOS1
SOS2
SOX11
SOX13
SOX17
SOX2-OT
SOX4
SOX5
SOX6
SP1
SP3
SP3P
SP4
SP8
SPAG1
SPAG16
SPAG17
SPAG6
SPAG9
SPAM1
SPARCL1
SPAST
SPATA13

SPATA16
SPATA17
SPATA19
SPATA5
SPATA5L1
SPATA6
SPATA9
SPATC1
SPATS1
SPATS2
SPATS2L
SPDEF
SPDYA
SPECC1
SPEF2
SPEN
SPG11
SPHKAP
SPIC
SPICE1
SPIN1
SPINK14
SPINK5
SPINK8
SPIRE1
SPOCK1
SPOCK3
SPOP
SPOPL
SPPL3
SPRED1
SPRNP1
SPRR2B
SPRR2F
SPRY3
SPSB4
SPTAN1
SPTBN1
SPTLC1
SPTSSB
SRBD1
SRD5A2
SRF
SRGAP1
SRGAP2

SRGAP2P1
SRGAP2P2
SRGAP3
SRP14P1
SRP54
SRP9
SRPK1
SRPK2
SRPRB
SRRM1P1
SRRM4
SRSF10
SRSF11
SRSF12
SSB
SSBP2
SSH2
SSPN
ST13
ST13P13
ST13P8
ST18
ST20-MTHFS
ST3GAL1
ST3GAL6
ST6GAL1
ST6GALNAC3
ST6GALNAC5
ST7
ST7-OT3
ST8SIA1
ST8SIA4
ST8SIA5
ST8SIA6
STAB2
STAC
STAG1
STAG3
STAM
STAM2
STAP1
STARD13
STARD3NL
STARD7
STARD9

STAT4
STAT5B
STAU2
STEAP1
STEAP1B
STEAP2
STEAP4
STIL
STIM2
STK17A
STK3
STK31
STK32A
STK38L
STK39
STK4
STMN2
STOM
STON1-GTF2A1L
STON2
STOX1
STRADB
STRBP
STRN
STRN3
STT3B
STX17
STX6
STXBP1
STXBP5
STXBP5L
STXBP6
STYK1
STYX
SUB1
SUCLG2
SULF1
SULT1E1
SUMF1
SUN1
SUPT16H
SUPT3H
SUPV3L1
SUSD1
SUSD4

SUSD5
SUV420H1
SUZ12
SV2C
SVEP1
SVIL
SVOP
SVOPL
SWT1
SYBU
SYCP1
SYCP2
SYCP2L
SYCP3
SYK
SYN2
SYNCRIP
SYNE2
SYNGR2P2
SYNJ1
SYNJ2
SYNPO2
SYNPR
SYNRG
SYPL1
SYT1
SYT10
SYT14
SYT16
SYT2
SYTL2
TAB2
TAC1
TACC1
TACR3
TADA2A
TAF15
TAF1A
TAF1B
TAF2
TAF3
TAF4B
TAG
TAL2
TANC1

TANC2
TANK
TAOK1
TAOK3
TAPT1
TARBP1
TARP
TARS
TARSL2
TASP1
TATDN1
TATDN3
TAX1BP1
TBC1D1
TBC1D12
TBC1D14
TBC1D15
TBC1D19
TBC1D23
TBC1D2B
TBC1D3
TBC1D30
TBC1D3F
TBC1D4
TBC1D5
TBC1D8
TBCE
TBCEL
TBCK
TBL1XR1
TBL1Y
TBPL1
TBX15
TBX20
TBX3
TBXAS1
TCEA1P1
TCEA1P3
TCEAL1
TCEANC2
TCEB1
TCERG1L
TCF12
TCF24
TCF4

TCFL5
TCHHL1
TCP11L2
TCP1P2
TCTE1
TCTN2
TCTN3
TDG
TDGF1P4
TDP2
TDPX2
TDRD10
TDRD3
TDRD5
TDRD7
TEC
TECRL
TECTA
TEK
TEKT3
TEKT4
TEKT4P2
TEP1
TERT
TES
TESK1
TET1
TET2
TEX10
TEX9
TFAP2A
TFB1M
TFB2M
TFCP2
TFCP2L1
TFDP2
TFEC
TFG
TFPI
TFRC
TG
TGFB2
TGFBR1
TGFBRAP1
TGIF1

TGM5
THBS1
THEM4
THEMIS
THRAP3
THRB
THSD1
THSD4
THSD7A
THSD7B
THUMPD3
TIAM1
TIMM8AP1
TIPARP
TJAP1
TJP1
TJP2
TLE1
TLE3
TLE4
TLK1
TLK2
TLL1
TLL2
TLN2
TLR3
TM2D1
TM4SF1
TM4SF4
TM7SF3
TM9SF3
TMC1
TMC3
TMCC1
TMCC2
TMCC3
TMED10P1
TMED4
TMEFF1
TMEFF2
TMEM106B
TMEM108
TMEM11
TMEM111
TMEM116

TMEM117
TMEM123
TMEM131
TMEM132B
TMEM132D
TMEM135
TMEM139
TMEM14C
TMEM150C
TMEM156
TMEM161B
TMEM163
TMEM170B
TMEM178
TMEM182
TMEM183A
TMEM194A
TMEM194B
TMEM196
TMEM2
TMEM202
TMEM206
TMEM209
TMEM229A
TMEM232
TMEM38B
TMEM45A
TMEM45B
TMEM48
TMEM55A
TMEM62
TMEM63B
TMEM65
TMEM67
TMEM71
TMEM86A
TMEM87A
TMEM87B
TMF1
TMIGD1
TMOD1
TMOD2
TMOD3
TMPO
TMPRSS15

TMPRSS2
TMPRSS7
TMTC1
TMTC2
TMX1
TMX2-CTNND1
TNC
TNFAIP6
TNFRSF11A
TNFRSF11B
TNFRSF13B
TNFSF10
TNFSF15
TNFSF18
TNFSF4
TNFSF8
TNIK
TNN
TNNI3K
TNPO1
TNPO3
TNR
TNRC18
TNRC6B
TNRC6C
TNS3
TOB1
TOM1L2
TOMM20
TOMM70A
TOP1
TOP2A
TOP2B
TOPBP1
TOR1AIP1
TOR1AIP2
TOX
TOX4
TP53AIP1
TP53BP1
TP53BP2
TP53INP1
TP53TG1
TP63
TPCN1

TPD52
TPH1
TPI1P1
TPK1
TPM1
TPM3
TPM3P4
TPO
TPP2
TPPP
TPR
TPRG1
TPRXL
TPST1
TPTE
TPX2
TRA@
TRA2A
TRA2B
TRAF1
TRAF3IP2-AS1
TRAF3IP3
TRAF5
TRAK1
TRAK2
TRAM1
TRANK1
TRAPPC8
TRAPPC9
TRB@
TRBV20OR9-2
TRBV30
TRD@
TRDMT1
TRDN
TRG@
TRGC2
TRHDE
TRIB1
TRIM13
TRIM14
TRIM2
TRIM24
TRIM32
TRIM36

TRIM4
TRIM43B
TRIM44
TRIM49
TRIM55
TRIM58
TRIM69
TRIM71
TRIM9
TRIO
TRIP13
TRIP4
TRMT11
TRMT61B
TRNAA14
TRNAA32
TRNAA7
TRNAC11
TRNAC2
TRNAC20
TRNAC21
TRNAC25
TRNAC27
TRNAC6
TRNAE38P
TRNAG10
TRNAG19
TRNAH5
TRNAI12
TRNAL19
TRNAM-CAU
TRNAM5
TRNAN3
TRNAQ32
TRNAQ51P
TRNAR32P
TRNAY17P
TRNT1
TROVE2
TRPA1
TRPC1
TRPC3
TRPC4
TRPC4AP
TRPC6

TRPC6P
TRPM1
TRPM3
TRPM6
TRPM7
TRPS1
TRPV6
TRRAP
TSC1
TSC22D1
TSC22D2
TSG1
TSG101
TSGA10
TSGA13
TSGA14
TSHR
TSHZ1
TSHZ2
TSNAX
TSNAX-DISC1
TSPAN11
TSPAN12
TSPAN13
TSPAN8
TSTD2
TTBK1
TTBK2
TTC1
TTC13
TTC15
TTC17
TTC18
TTC21B
TTC23
TTC23L
TTC26
TTC27
TTC28
TTC29
TTC33
TTC35
TTC37
TTC39B
TTC39C

TTC6
TTC7B
TTF1
TTF2
TTL
TTLL11
TTLL5
TTLL6
TTN
TTPA
TTR
TTTY15
TUBA1A
TUBAL3
TUBBP1
TUBGCP5
TUFT1
TUSC3
TUT1
TWSG1
TXLNB
TXNDC11
TXNDC16
TXNDC3
TXNIP
TXNL4A
TXNRD1
TXNRD3
TXNRD3NB
TYR
TYW1
TYW1B
U2SURP
UACA
UBA6
UBAC2
UBAP1
UBAP2
UBAP2L
UBE2CBP
UBE2D3P4
UBE2D4
UBE2E1
UBE2E2
UBE2E3

UBE2G1
UBE2H
UBE2J1
UBE2K
UBE2N
UBE2Q2
UBE2R2
UBE2T
UBE2V2
UBE2W
UBE3A
UBE3B
UBE3C
UBE4A
UBE4B
UBN2
UBP1
UBQLN1
UBR1
UBR3
UBR4
UBR5
UBXN2A
UBXN2B
UBXN4
UBXN7
UCHL5
UEVLD
UGCG
UGDH
UGGT1
UGGT2
UGT1A10
UGT1A3
UGT1A4
UGT1A5
UGT1A6
UGT1A7
UGT1A8
UGT1A9
UGT2B15
UGT2B4
UGT3A1
UGT3A2
UHRF1BP1

UHRF1BP1L
UHRF2
ULK2
ULK4
UNC13B
UNC13C
UNC5C
UNC80
UNQ6494
UNQ6975
UPF2
UPF3A
UPK1B
UPP2
UQCC
UQCRB
UQCRC1
URB2
USH2A
USO1
USP13
USP14
USP15
USP24
USP25
USP28
USP3
USP31
USP32
USP33
USP34
USP40
USP42
USP43
USP44
USP45
USP48
USP50
USP53
USP54
USP6NL
USP8
USP9YP3
UTP20
UTP6

UTRN
UTS2D
UVRAG
VAMP7
VANGL1
VAPA
VAPB
VASH2
VAV2
VCAN
VCL
VCPIP1
VDAC1P11
VDR
VEGFA
VEGFC
VEPH1
VEZT
VGLL4
VIPR1
VIPR2
VKORC1L1
VMP1
VN1R40P
VN1R47P
VN1R59P
VN1R74P
VN1R7P
VNN1
VOPP1
VPRBP
VPS13A
VPS13B
VPS13C
VPS13D
VPS26A
VPS29
VPS37C
VPS41
VPS45
VPS4B
VPS54
VPS8
VSNL1
VSTM4

VTA1
VTCN1
VWA3B
VWC2
VWC2L
VWDE
VWF
WAC
WARS2
WASL
WBP4
WBSCR17
WDFY2
WDFY3
WDFY4
WDHD1
WDPCP
WDR12
WDR17
WDR19
WDR26
WDR33
WDR35
WDR37
WDR43
WDR48
WDR49
WDR52
WDR60
WDR64
WDR67
WDR7
WDR70
WDR72
WDR76
WDR78
WDR82
WDR86
WDR89
WDSUB1
WDYHV1
WHAMM
WHAMMP3
WHSC1L1
WIBG

WIF1
WIPF1
WIPF2
WIPF3
WISP1
WNK2
WNT16
WNT2
WNT3A
WRN
WTH3DI
WWC1
WWC2
WWOX
WWP1
WWTR1
XCR1
XDH
XIRP2
XKR4
XKR9
XPA
XPO1
XPO4
XPO5
XPOT
XPR1
XRCC2
XRCC4
XRCC5
XRN1
XRN2
XRRA1
XYLB
YAF2
YAP1
YARS2
YBX1
YBX1P10
YBX1P6
YEATS2
YEATS4
YES1
YIPF4
YME1L1

YOD1
YPEL1
YSK4
YTHDC1
YTHDF1
YTHDF3
YWHAE
YWHAG
YWHAQ
YWHAQP3
YWHAZ
YWHAZP4
ZAK
ZBBX
ZBED1
ZBTB10
ZBTB11
ZBTB16
ZBTB20
ZBTB20-AS1
ZBTB37
ZBTB38
ZBTB43
ZBTB44
ZBTB5
ZBTB7C
ZC3H11A
ZC3H4
ZC3H6
ZC3HAV1
ZC3HC1
ZCCHC11
ZCCHC2
ZCCHC4
ZCCHC6
ZCCHC7
ZCWPW2
ZDHHC11
ZDHHC11B
ZDHHC13
ZDHHC14
ZDHHC17
ZDHHC21
ZEB1
ZEB2

ZER1
ZFAND3
ZFAND5
ZFAND6
ZFAT
ZFHX4
ZFP106
ZFP91
ZFP91-CNTF
ZFPM2
ZFR
ZFYVE16
ZFYVE9
ZHX1
ZHX1-C8ORF76
ZHX2
ZKSCAN1
ZMAT1
ZMAT3
ZMAT4
ZMPSTE24
ZMYM2
ZMYM4
ZMYND11
ZNF10
ZNF131
ZNF138
ZNF140
ZNF148
ZNF16
ZNF169
ZNF18
ZNF197
ZNF207
ZNF212
ZNF217
ZNF227
ZNF234
ZNF236
ZNF24
ZNF250
ZNF251
ZNF252
ZNF26
ZNF268

ZNF277
ZNF280D
ZNF282
ZNF292
ZNF318
ZNF32-AS3
ZNF33A
ZNF33B
ZNF365
ZNF367
ZNF385B
ZNF385D
ZNF397
ZNF398
ZNF407
ZNF425
ZNF438
ZNF451
ZNF462
ZNF483
ZNF484
ZNF503-AS1
ZNF507
ZNF512
ZNF516
ZNF519
ZNF521
ZNF532
ZNF589
ZNF592
ZNF595
ZNF605
ZNF609
ZNF618
ZNF619
ZNF624
ZNF638
ZNF639
ZNF652
ZNF664-FAM101A
ZNF670
ZNF670-ZNF695
ZNF678
ZNF680
ZNF692

ZNF695
ZNF697
ZNF704
ZNF706
ZNF713
ZNF717
ZNF736
ZNF746
ZNF767
ZNF780B
ZNF782
ZNF783
ZNF800
ZNF804A
ZNF804B
ZNF827
ZNF84
ZNF862
ZNF876P
ZNRD1-AS1
ZNRF2
ZPBP
ZPLD1
ZRANB1
ZRANB3
ZSCAN30
ZSWIM2
ZSWIM6
ZUFSP
ZW10
ZZZ3
tAKR
