# Supplementary material for: Comprehensive genomics in androgen receptor-dependent castration-resistant prostate cancer identifies an adaptation pathway mediated by opioid receptor kappa 1
Source: Commun Biol. 2022 Apr 1;5:299. doi: 10.1038/s42003-022-03227-w (PMC8976065; doi:10.1038/s42003-022-03227-w)
Supplement: Supplementary file 4 — Supplementary Data S2 [file 42003_2022_3227_MOESM4_ESM.pdf]

Supplementary data S2. List of genes called in the ChIP sequence experiments including 2,938 called exclusively for LNCaP, 717 for AILNCaP, and 1,751 shared with both LNCaP and AILNCaP cells

LNCaP	AILNCaP	Common
A1CF	AADACL2	AAA1
A2M	AASS	ABCA12
AADAT	AATF	ABCA13
AAGAB	ABCB5	ABCA17P
AAK1	ABCC12	ABCA5
ABCA1	ABRA	ABCA6
ABCA4	ACSL6	ABCA8
ABCA9	ACSM1	ABCB1
ABCB4	ACTR5	ABCB10
ABCC1	ACVR2A	ABCB11
ABCC4	ADAM23	ABCD1P3
ABCC9	ADAM28	ABHD2
ABCD2	ADIPOR1	ABL2
ABCD3	ADRA1B	ABLIM1
ABHD12	AGBL3	ACACA
ABI1	AGGF1	ACAP2
ABI2	AGGF1P3	ACBD6
ABL1	AGPS	ACCN1
ABTB2	AIDA	ACER3
ACAD11	AK4P5	ACOXL
ACADL	AKR1E2	ACPP
ACADSB	ALDH5A1	ACSL3
ACBD3	ALOX5AP	ACSL5
ACBD5	ANKRD20A8	ADAM10
ACMSD	ANKRD29	ADAM12
ACN9	ANKRD35	ADAM2
ACP6	ANXA3	ADAM22
ACSM2A	AOAH	ADAM32
ACSM4	AP2B1	ADAM7
ACSS3	APOB	ADAMTS12
ACTR3	ARHGAP17	ADAMTS16
ACTR3B	ARHGAP19-[illegible]	ADAMTS19
ACTR3P1	ARHGAP20	ADAMTS20
ACVR1	ARHGAP22	ADAMTS3
ACVR1C	ARHGAP30	ADAMTS6
ACYP2	ARHGAP44	ADAMTS9
ADAD1	ARHGAP6	ADAMTS9-AS2
ADAM17	ARHGEF11	ADAMTSL1
ADAM18	ARL13B	ADAMTSL3
ADAM5P	ASPH	ADARB2

ADAM9	ASS1P10	ADCY10
ADAMTS17	ATP1B1	ADCY2
ADAMTS18	ATP5G2P2	ADCY8
ADAR	ATP6V0A4	ADD3
ADARB1	ATP6V0E1	ADK
ADCK1	B4GALT4	ADSS
ADCK3	B4GALT5	AFF3
ADH1C	BCL2L1	AFF4
ADH5P2	BCL6	AGAP9
ADI1	BCL9	AGBL1
ADORA3	BDAG1	AGBL4
ADRA1A	BMP6	AGK
ADRB1	BRD4	AGMO
ADRBK2	BTBD8	AHCTF1
AFF1	BTF3	AHI1
AFF2	BTNL2	AK4P2
AFG3L2	BVES	AK5
AGAP1	C10orf81	AKAP2
AGFG1	C11orf46	AKAP6
AGPAT4	C14orf39	AKAP7
AGPAT6	C16orf62	AKAP9
AGR2	C18orf42	AKD1
AGTPBP1	C1orf106	AKT3
AGTR1	C1orf227	ALCAM
AHCYL2	C1QTNF2	ALK
AHNAK	C20orf194	ALOX5
AIG1	C3orf80	AMD1
AIM2	C4BPA	AMPH
AK4	C6orf186	ANK2
AKAP1	C7orf46	ANK3
AKAP12	C8A	ANKFN1
AKAP13	C8orf42	ANKH
AKR1B1P3	C9orf174	ANKRD28
AKR1D1	C9orf46	ANKRD31
ALDH18A1	CA13	ANKRD44
ALDH1A2	CA2	ANKRD45
ALDH1A3	CAGE1	ANKRD7
ALDH9A1	CALB1	ANKS1B
ALG6	CALCRL	ANO10
ALG8	CAPRIN1	ANO3
ALG9	CATSPERB	ANO4
ALKBH1	CCDC122	ANO5
ALMS1	CCDC14	ANP32E
ALPK2	CCDC149	ANTXR2
ALS2CR11	CCDC18	AOX1

AMOTL1	CCDC50	AP3B1
AMPD3	CCDC73	APBA1
AMTN	CCNH	ARHGAP15
AMY1B	CCT6P5	ARHGAP18
AMY2A	CD34	ARHGAP21
AMY2B	CDK17	ARHGAP24
ANAPC5	CDK5RAP1	ARHGAP26
ANGEL1	CDK5RAP2	ARHGAP32
ANGEL2	CDK6	ARHGEF26
ANGPT1	CFHR2	ARHGEF26-AS1
ANGPT2	CFHR3	ARHGEF3
ANKFY1	CGN	ARID1B
ANKIB1	CHP2	ARID4B
ANKRD11	CHRNA7	ARL15
ANKRD13C	CLCN3	ARSB
ANKRD18A	CLEC16A	ASCC1
ANKRD26	CLMP	ASCC3
ANKRD26P1	CLTCL1	ASH1L
ANKRD30A	CLVS2	ASTN1
ANKRD30BL	CNOT10	ASTN2
ANKRD30BP	CNTRL	ASXL2
ANKRD36	COBL	ASZ1
ANKRD36B	COG2	ATF6
ANKRD36BP	COIL	ATG10
ANKRD36BP	COL28A1	ATP10B
ANKRD42	COL4A3BP	ATP10D
ANKRD55	COL6A4P1	ATP11B
ANKRD62	COLEC12	ATP2B1
ANKRD64	COPS3	ATP6V1A
ANO2	CPXM2	ATP6V1H
ANO6	CREB5	ATP8A2
ANP32BP2	CRTC3	ATR
ANUBL1	CSNK1G3	ATRNL1
ANXA4	CTAGE4	ATXN1
ANXA7	CTNNA1	ATXN7L1
AOX2P	CTNND1	AUH
AP1AR	CXCL13	AUTS2
AP1G1	CYB5R4	AXDND1
AP3S1	CYCSP3	B3GAT2
AP4E1	CYFIP2	BAGE2
AP4S1	CYP2C19	BAI3
APAF1	D21S2088E	BANK1
APBA2	DAAM2	BBS9
APBB1IP	DAPP1	BBX
APBB2	DARS2	BCAS3

APC	DCDC1	BCKDHB
APCDD1	DCDC2	BECN1P1
APP	DCLK3	BEND7
APPBP2	DCP2	BFSP2
APPL1	DEFA4	BICC1
APPL2	DEFB118	BMP5
AQP10	DEFB123	BMPER
AQR	DHRS7	BMPR1B
ARF1	DIP2C	BMPR2
ARFGEF1	DIRC1	BNC2
ARFGEF2	DLGAP2	BNIP3P4
ARHGAP12	DNAH11	BRD2
ARHGAP42	DND1P1	BRE
ARHGEF35	DPT	BRIP1
ARHGEF37	DPY19L1	BTAF1
ARHGEF38	DPY19L2P3	BTBD11
ARID2	DPYSL2	BTBD9
ARIH2	DROSHA	BTRC
ARL2BPP4	DSTYK	C10orf107
ARMC2	DTNBP1	C10orf11
ARMC3	DUX4L16	C10orf112
ARMC4	DUX4L17	C10orf68
ARMC8	DYRK2	C11orf44
ARMC9	ECM2	C11orf49
ARNT2	ECT2L	C12orf55
ARNTL2	EEF1A1P28	C12orf63
ARPC5	EEF1DP4	C15orf33
ARPP21	EFCAB2	C15orf41
ARSG	EFHA2	C15orf60
ARSK	EFTUD1	C18orf34
ASAP1	EHF	C1orf100
ASB13	EIF4A1	C1orf101
ASB14	EIF4A1P11	C1orf105
ASB5	EIF4A2	C1orf110
ASCC2	ELOVL4	C1orf116
ASCL4	ELP3	C1orf129
ASPM	EMID2	C1orf21
ASPSCR1	EMX2OS	C1orf27
ASRGL1	ENPP5	C1orf65
ASS1P13	EP300	C1orf9
ASS1P14	EPHX4	C20orf26
ASS1P7	EPM2A	C20orf61
ASXL1	EVI5	C22orf26
ATAD2	F13A1	C3orf26
ATAD2B	F8	C3orf67

ATE1	FAM102B	C4BPAP2
ATF2	FAM120B	C4orf22
ATF3	FAM126A	C4orf37
ATF7	FAM160B1	C5
ATF7IP	FAM66E	C5orf13
ATG2B	FAM92A1	C5orf46
ATG4C	FASTKD1	C6orf10
ATG7	FBXO25	C6orf103
ATIC	FBXO5	C6orf138
ATL3	FCAMR	C6orf170
ATM	FCGR2A	C6orf174
ATP13A3	FDPS	C6orf204
ATP13A4	FGF10	C7orf10
ATP13A5	FH	C7orf58
ATP1A1OS	FLJ34747	C7orf63
ATP1A2	FLJ36000	C8orf38
ATP1A4	FLJ46361	C8orf44-SGK3
ATP2B4	FOXA1	C9orf30-TMEFF1
ATP2C1	FRDAP	C9orf84
ATP6AP1L	FREM2	CA10
ATP6V0D2	FREM3	CA14
ATP6V1C1	FRG1	CACNA1A
ATP8A1	FRMPD2	CACNA1B
ATP8B1	FYB	CACNA1C
ATP8B4	FYN	CACNA1D
ATP8B5P	GALNT14	CACNA1E
ATP9B	GAPDHP28	CACNA2D1
ATPBD4	GAPDHP36	CACNA2D3
ATRN	GAPDHP63	CACNB2
ATXN10	GAPDHP64	CACNB4
ATXN2	GBP5	CADM1
ATXN7	GFOD1	CADM2
AURKA	GFRA1	CADPS
AVEN	GGT1	CADPS2
AZFP	GLIPR1	CALN1
B3GALT1	GLIS1	CAMK1D
B3GALTL	GLMN	CAMK4
B3GNT6	GLRA3	CAMSAP2
B4GALT1	GLS	CAMTA1
BAALC	GNAS	CAP2
BAIAP2L1	GNB1	CAPN2
BARD1	GNPAT	CAPN8
BATF	GOLGA6L2	CBFA2T2
BAZ1B	GOLIM4	CBLB
BAZ2B	GOLPH3L	CCDC102B

BBS4	GPBP1L1	CCDC129
BCAP29	GPR110	CCDC141
BCAS1	GPR128	CCDC150
BCAT1	GPR137B	CCDC158
BCL10	GPR139	CCDC19
BCL2	GPR53P	CCDC30
BDNF	GPR87	CCDC53
BDNF-AS1	GTF3C1	CD109
BDP1	GUCY1A3	CD247
BECN1	GUCY2C	CD55
BEND4	GYPA	CD86
BEND6	HACE1	CD96
BEST3	HCRTR2	CDC14A
BFAR	HECTD1	CDC14B
BICD1	HEPHL1	CDC23
BIRC2	HIBADH	CDC42BPA
BIRC6	HIRA	CDH10
BLM	HIST3H2BB	CDH11
BLVRB	HIST3H3	CDH12
BNIP2	HK1	CDH13
BNIP3P3	HLF	CDH18
BOLA1	HMGCR	CDH19
BOLL	HMGCS2	CDH2
BPIFA4P	HMGXB3	CDH4
BRAF	HNRNPA2B1	CDH8
BRP44	HNRNPU-AS	CDH9
BTBD10	HOXA4	CDK14
BTLA	HS2ST1	CDK19
BTNL3	HS3ST2	CDKAL1
BUB1B	HSPA4	CDKN2B-AS
BZW1	IER5	CDON
C10orf118	IFRD1	CDV3
C10orf128	IGSF3	CELF2
C10orf46	IKZF3	CENPF
C10orf67	IL1RL1	CENPP
C10orf90	IL2RA	CEP112
C10orf92	IL36B	CEP120
C10orf96	ILDR2	CEP128
C11orf30	INA	CEP170
C11orf41	INPP5A	CEP350
C11orf63	IRAK3	CEP63
C11orf74	IRF6	CERS6
C11orf75	ISCUP1	CFH
C11orf80	KBTBD2	CFHR1
C11orf85	KCNJ6	CFTR

C11orf92	KCNU1	CHCHD6
C11orf93	KIAA0319L	CHD2
C12orf4	KIAA0907	CHD6
C12orf40	KIAA1245	CHL1
C12orf42	KIAA1279	CHN2
C12orf51	KIAA1549	CHRM2
C12orf68	KIAA1804	CHRM3
C13orf41	KIAA1958	CHST9
C14orf105	KIF13B	CHSY3
C14orf159	KIR2DL4	CHURC1-FNTB
C14orf166	KLF7P1	CLASP2
C14orf37	KLHL31	CLIP1
C14orf56	KLHL7	CLNK
C15orf29	KLK2	CLRN1-AS1
C15orf32	KLK7	CLSTN2
C15orf57	KRT18P16	CLVS1
C16orf46	KRT18P36	CLYBL
C16orf61	KRT18P50	CMTM8
C16orf72	KRTAP19-10	CNBD1
C17orf48	KYNU	CNGB3
C17orf57	LCP2	CNIH3
C17orf63	LCT	CNST
C17orf67	LINC00158	CNTLN
C17orf81	LINC00238	CNTN1
C18orf1	LINC00301	CNTN3
C18orf12	LINC00488	CNTN4
C19orf2	LOC1001280	CNTN5
C1orf111	LOC1001281	CNTN6
C1orf112	LOC1001284	CNTNAP2
C1orf115	LOC1001287	CNTNAP4
C1orf124	LOC1001287	CNTNAP5
C1orf173	LOC1001288	COBLL1
C1orf192	LOC1001290	COG5
C1orf31	LOC1001290	COL11A1
C1orf61	LOC1001292	COL14A1
C1orf87	LOC1001293	COL19A1
C1orf95	LOC1001295	COL22A1
C1QTNF7	LOC1001295	COL23A1
C20orf103	LOC1001296	COL24A1
C20orf132	LOC1001297	COL25A1
C20orf197	LOC1001299	COL5A2
C20orf94	LOC1001300	COL6A6
C22orf34	LOC1001302	COL8A1
C2CD3	LOC1001302	COL9A1
C2orf43	LOC1001306	COMMD3-BMI1

C2orf55	LOC1001310	COPA
C2orf67	LOC1001314	CORIN
C2orf80	LOC1001315	CPA6
C3orf33	LOC1001330	CPEB4
C3orf70	LOC1001333	CPNE4
C3orf77	LOC1002164	CPNE8
C4orf43	LOC1002879	CPS1
C5orf17	LOC1002887	CPVL
C5orf25	LOC1002887	CR1L
C5orf27	LOC1002893	CRADD
C5orf42	LOC1002895	CRB1
C5orf50	LOC1002896	CSGALNACT1
C5orf51	LOC1002944	CSMD1
C6	LOC1004197	CSMD2
C6orf106	LOC1004197	CSMD3
C6orf127	LOC1004199	CSPP1
C6orf183	LOC1004199	CSRNP3
C6orf191	LOC1004199	CSRP1
C6orf201	LOC1004200	CTNNA2
C6orf222	LOC1004202	CTNNA3
C6orf62	LOC1004205	CTNND2
C6orf89	LOC1004210	CTSL1P2
C6orf97	LOC1004213	CTTNBP2
C7orf31	LOC1004214	CUBN
C7orf42	LOC1004215	CYP1B1-AS1
C7orf60	LOC1004216	CYP3A43
C7orf64	LOC1004217	CYP4Z1
C7orf74	LOC1004220	DAAM1
C8orf34	LOC1004222	DAB1
C8orf83	LOC1004994	DAPK1
C8orf84	LOC1005054	DBC1
C9orf150	LOC1005055	DCAF5
C9orf153	LOC1005056	DCAF6
C9orf3	LOC1005057	DCBLD2
C9orf5	LOC1005058	DCC
C9orf72	LOC1005058	DCDC5
C9orf91	LOC1005058	DCHS2
C9orf93	LOC1005059	DCLK1
CA12	LOC1005060	DDAH1
CA8	LOC1005061	DDR2
CAB39	LOC1005064	DDX10
CAB39L	LOC1005067	DDX60L
CACHD1	LOC1005068	DENND1A
CACNA2D4	LOC1005072	DENND1B
CACNG2	LOC1005074	DENND2C

CACNG3	LOC1005338	DEPDC1B
CADM3	LOC1006528	DEPTOR
CALD1	LOC1006529	DGKB
CALM1	LOC1006529	DGKG
CALM1P2	LOC1006530	DGKI
CALM2P1	LOC147670	DHX32
CALR4P	LOC150622	DIAPH3
CALU	LOC153910	DICER1
CAMK1G	LOC220980	DISC1
CAMK2B	LOC283050	DISP1
CAMK2D	LOC283299	DKFZp686O1327
CAMKK2	LOC283856	DKK2
CAMKMT	LOC284441	DLC1
CAPG	LOC285286	DLG2
CAPN14	LOC285889	DLGAP1
CAPN5	LOC339166	DMD
CAPSL	LOC343508	DMXL2
CAPZA1	LOC347097	DNAH12
CAPZA2	LOC388780	DNAH14
CASC2	LOC390099	DNAH3
CASC4	LOC390858	DNAH5
CASC5	LOC392268	DNAH6
CASP7	LOC401074	DNAH7
CASR	LOC401847	DNAH8
CAST	LOC440970	DNAH9
CASZ1	LOC553103	DNAJC1
CAT	LOC642461	DNAJC13
CBWD3	LOC642659	DNAJC3
CBX1P3	LOC643438	DNAJC5B
CBX3P8	LOC643441	DNAJC6
CCAR1	LOC643623	DNER
CCBE1	LOC643714	DNM3
CCBL2	LOC643884	DOCK1
CCBP2	LOC643916	DOCK10
CCDC109B	LOC644681	DOCK3
CCDC132	LOC645415	DOCK4
CCDC136	LOC645598	DOCK5
CCDC144B	LOC647979	DPP10
CCDC146	LOC648442	DPP6
CCDC148	LOC653458	DPY19L1P1
CCDC15	LOC727713	DPYD
CCDC38	LOC728012	DRD5P2
CCDC39	LOC729296	DSCAM
CCDC40	LOC729852	DSE
CCDC41	LOC93432	DST

CCDC57	LRRC37A	DTL
CCDC6	LRRC52	DTNA
CCDC60	LRRN2	DTWD2
CCDC64	LUC7L3	DUSP22
CCDC66	MAN1A1	DUX4L19
CCDC67	MAP1B	DUX4L9
CCDC68	MARK3	DYNC1I1
CCDC81	MBOAT1	DZIP1
CCDC82	MCHR2	EBF1
CCDC85A	MDM4	EBF2
CCDC88A	MED15P1	EDARADD
CCDC88C	MED18	EDEM3
CCDC91	MEOX1	EDIL3
CCDC93	METTL16	EEF1E1
CCL1	METTL9	EEF1E1-MUTED
CCL13	MFSD4	EEFSEC
CCL8	MGAT5	EFCAB1
CCNC	MINA	EFCAB11
CCNJ	MIR3924	EFCAB6
CCNY	MIR4273	EFNA5
CCNYL1	MIR548AE1	EGFEM1P
CCNYL2	MLIP	EHMT1
CCT6B	MLLT11	EIF2C3
CCT6P3	MMP8	EIF2S1
CD1C	MOBP	EIF4BP8
CD2	MORF4L1P7	EIF4H
CD244	MRPL48	ELMO1
CD2AP	MRPS21	ELOVL7
CD44	MYBPC1	EML5
CD46	MYH11	ENAH
CD47	MYL1	ENOX1
CD48	MYL6P3	ENPP1
CD5L	MYO18B	ENPP3
CD84	NBPF1	EPB41
CDC42EP3	NCEH1	EPB41L2
CDC42SE2	NCK2	EPB41L4A
CDC73	NCOA7	EPB41L4B
CDH23	NDUFA10	EPHA3
CDH26	NES	EPHA4
CDH6	NFATC2	EPHA5
CDK2AP2P3	NGLY1	EPHA6
CDKL1	NID2	EPHA7
CDKL3	NIPA2P1	EPHB1
CDYL	NIPAL2	EPHX1
CDYL2	NLRP5	EPN2

CEBPA	NNMT	EPS15
CEBPD	NOVA1	ERBB2IP
CENPJ	NPM1	ERBB4
CENPL	NPSR1	ERC2
CEP135	NT5DC3	ERCC6
CEP152	NT5M	ESR1
CEP192	NTN1	ESRRG
CEP290	NTRK1	ETV6
CEP70	OBSCN	EXOC2
CEP76	OPA1	EXOC4
CEP78	OR13G1	EXOC6B
CEP95	OR13I1P	EYA2
CERKL	OR2AQ1P	EYA3
CERS3	OR2B11	EYA4
CES2	OR2I1P	EYS
CFHR4	OR2T27	F13B
CFL1P2	OR2T4	F5
CFTRP1	OR51F4P	FABP5P5
CGNL1	OR51I1	FAM107B
CHCHD2P3	OR52J2P	FAM110B
CHCHD3	OR56A4	FAM117B
CHCHD5	OR5B15P	FAM129A
CHCHD7	OR5H14	FAM135A
CHD7	OR5H4P	FAM135B
CHD9	OR6L1P	FAM138D
CHEK1	OR6N2	FAM138E
CHKA	OR7A18P	FAM13A
CHN1	OR8A1	FAM13C
CHODL	OSBPL3	FAM155A
CHP	OTX2OS1	FAM164A
CHST11	OVCH1	FAM172A
CHST13	P2RY12	FAM184A
CHST8	P4HA3	FAM190A
CHST9-AS1	PALB2	FAM190B
CHSY1	PCBP2P3	FAM19A1
CIB4	PCCB	FAM19A2
CIT	PCDP1	FAM19A4
CKAP5	PCGF5	FAM36A
CLASP1	PCP4L1	FAM5C
CLCN1	PDCD2	FAM65B
CLDN16	PDCD4	FAM66A
CLDN18	PFDN1	FAM66B
CLEC1A	PFKFB2	FAM66D
CLEC4E	PHACTR4	FAM69A
CLIC5	PHBP3	FAM78B

CLINT1	PHF21A	FAM83B
CLIP4	PHTF1	FAM87B
CLNS1AP1	PI4KB	FAM90A10
CLRN1	PIGK	FAM91A3P
CLTC	PIGN	FANK1
CLUL1	PIGR	FARS2
CMAHP	PIP5K1A	FAT3
CMPK1	PIWIL1	FBN1
CNKSR2	PKD1L1	FBN2
CNKSR3	PKIG	FBXL17
CNNM1	PKLR	FBXL2
CNNM2	PLSCR4	FBXL7
CNOT4	PMF1	FBXO28
CNTNAP3	PMF1-BGLAI	FBXO38
COG3	POGK	FBXW7
COG6	POLB	FCHO2
COL12A1	POLR3GL	FCHSD2
COL21A1	PON3	FEM1C
COL4A4	PPAP2A	FER
COL4A5	PPAP2B	FER1L6
COL6A3	PPP2R3A	FER1L6-AS1
COL6A4P2	PPP4R1L	FGF12
COL6A5	PRDM6	FGF14
COMMD10	PRDM9	FGF14-IT1
COPG2	PRR23C	FGGY
COPS5	PRRX1	FHIT
COPS8	PSIP1	FILIP1L
COX10	PTGS2	FKBP5
COX6B1P5	PTPN7	FLJ20444
COX7B2	PTX3	FLJ21408
CPA1	PURA	FLJ25363
CPA5	PWWP2AP1	FLJ30838
CPD	PXT1	FLJ32955
CPEB1	PYGO1	FLJ33065
CPEB3	RAB2A	FLJ34690
CPO	RAB4A	FLJ35282
CPSF7	RABGAP1	FLJ37396
CR1	RAD21	FLJ37786
CR2	RALA	FLJ39080
CREB1	RAPGEF1	FLJ42709
CREB3L2	RASSF5	FLJ45872
CREBBP	RBFOX3	FLJ45974
CREBL2	RBM39	FLJ46010
CREG1	RFX3	FLNB
CRH	RGS18	FLVCR1

CRIM1	RGS4	FMN1
CRKL	RGS5	FMN2
CRPP1	RIMBP2	FMNL2
CRTAM	RIN3	FNBP1L
CRY1	RN7SL7P	FNDC3A
CRYBG3	RNF170	FNDC3B
CRYM	RNF217	FNIP1
CSAD	RNF220	FOXJ3
CSDA	RNPC3	FOXN3
CSE1L	RPL10AP9	FOXO3
CSGALNACT	RPL10P1	FOXP1
CSNK1A1	RPL13P7	FOXP2
CSNK1G1	RPL18P5	FPGT-TNNI3K
CSRP2BP	RPL21P24	FRG1B
CTAGE13P	RPL21P40	FRMD3
CTBP2	RPL23AP28	FRMD4A
CTBP2P1	RPL23AP60	FRMD4B
CTDSPL2	RPL23P9	FRMD5
CTGLF10P	RPL29P18	FRS2
CTGLF11P	RPL34P1	FRYL
CTNNBL1	RPL39P39	FSIP2
CTR9	RPL3P9	FSTL4
CTSK	RPL7AP17	FSTL5
CTSS	RPL7P9	FTO
CTTNBP2NL	RPL9P21	FUT8
CUL1	RPS15AP20	FZD3
CUL3	RPS15AP27	GABBR2
CUL5	RPS19P5	GABRA2
CUX1	RPS24P4	GABRB1
CUX2	RPS27P17	GABRB2
CWC22	RPS29P24	GABRB3
CWC27	RPS3AP34	GABRG3
CWF19L2	RPS4XP11	GADL1
CWH43	RPS4XP9	GALNT13
CXorf59	RPS6P14	GALNT2
CYB5B	RPS7P4	GALNT7
CYCSP30	RPSAP35	GALNTL6
CYCSP39	RPSAP7	GALR1
CYCSP53	RRP15	GAP43
CYP11A1	RSRC2	GAS2
CYP24A1	RSU1	GATAD2B
CYP2C18	RTN1	GBE1
CYP4X1	SAMHD1	GCM1
CYP4Z2P	SAR1AP3	GHR
CYP7B1	SAR1B	GIN1

DAP3	SCGB3A2	GLI3
DAPL1	SCN3A	GLIS3
DARS	SCNN1B	GLRA1
DAZL	SCPEP1	GLT25D2
DBF4	SDPR	GMDS
DBT	SEC11B	GNAI1
DCAF10	SECISBP2	GNAQ
DCAF8	SELP	GNG12
DCLK2	SENP3-EIF4/	GNG4
DCP1A	SENP6	GOLGA4
DDC	SEPT14	GOLGB1
DDHD1	SERPINI2	GOLPH3
DDX1	SESN1	GON4L
DDX18P6	SGMS2	GPAM
DDX31	SGTB	GPATCH2
DDX4	SH2D4B	GPC5
DDX59	SH3BP5L	GPC6
DDX6	SH3PXD2A	GPHN
DDX60	SIM1	GPLD1
DEC1	SKP1	GPM6A
DEFB126	SLAMF6	GPR156
DENND4A	SLC25A12	GPR158
DENND4C	SLC25A48	GPR161
DENND5B	SLC35A3	GPR39
DERA	SLC37A2	GPR89B
DGKD	SLC39A11	GPR98
DGKH	SLC4A10	GPRC6A
DHCR24	SLC9A2	GRAMD3
DHRS2	SLC9A4	GRB10
DHRS7B	SLIT1	GREB1
DHX15	SMEK1	GREM2
DHX36	SMG6	GRHL2
DHX9	SMOC2	GRIA1
DIP2A	SMYD2	GRIA4
DIP2B	SNAP47	GRID2
DIRAS2	SNORD116@	GRIK1
DIRC2	SNRPGP2	GRIK2
DIRC3	SNTN	GRIK4
DIS3L2	SNX7	GRIN2A
DKFZP564C'	SORT1	GRIN2B
DLG1	SOX14	GRIN3A
DLX6-AS1	SP140L	GRIP1
DMXL1	SPIN1	GRM1
DNA2	SPTLC3	GRM5
DNAI1	SRRM2	GRM7

DNAJC10	SRSF3	GRM8
DNAL1	SS18	GSG1L
DNASE1	ST13P12	GSK3B
DNASE2B	ST3GAL6	GTDC1
DNHD1	ST8SIA4	GUCY1A2
DNM1L	STK32B	GUSBP1
DNM1P46	STX19	HAPLN1
DOCK2	SUCLA2P2	HBS1L
DOCK7	SYCP2	HCN1
DOCK8	SYT17	HDAC9
DOK5	SYT6	HECTD2
DOK6	TBCD	HECW1
DPP4	TDRKH	HECW2
DPY19L4	TEAD1	HELZ
DPYS	TESK2	HERC2P3
DPYSL3	TET1	HERC3
DSC2	TF	HFM1
DSC3	TFDP1P	HHAT
DSG4	TGFBR1	HIPK2
DSTN	TGFBRAP1	HIVEP2
DTNB	TGM4	HIVEP3
DUSP16	TIAM1	HLA-DMA
DUSP19	TIGIT	HMCN1
DUSP23	TMBIM6	HMG20A
DUSP27	TMCC2	HMGA2
DUSP6	TMCO5A	HMGB1P18
DYM	TMEM111	HMGCLL1
DYNC1H1	TMEM116	HOOK3
DYNC2H1	TMEM45B	HPS3
DYRK3	TMPRSS11F	HPSE2
DZIP3	TMX2-CTNNI	HS3ST3A1
EAPP	TNFRSF10B	HS3ST4
EBF3	TNFSF18	HS6ST3
ECI2	TNNI1	HSD17B12
EDA	TPR	HSD17B7P1
EDAR	TRAF5	HSF5
EEA1	TRIM66	HTR4
EEF2K	TRIM71	HYDIN
EEPD1	TRNAC25	IARS2
EFCAB5	TRNAC6	IFLTD1
EFCAB7	TRNAK34	IFT80
EFCAB8	TRNAQ12	IGF1R
EFHC1	TRNAR32P	IGF2BP3
EFR3A	TRPC7	IGH@
EGF	TSC22D2	IGHV2OR16-5

EGFLAM	TSNAX	IGK@
EGFR	TTC18	IGKV1OR2-118
EGLN1	TTC29	IGL@
EHBP1	TTC34	IGSF11
EIF2AK4	TTLL7	IKZF2
EIF2B3	TYR	IL1RAP
EIF2D	UBBP4	IMMP2L
EIF3E	UBE2D3	IMPG1
EIF3EP2	UBE2U	INPP4B
EIF3FP1	UBE4B	INTS7
EIF3H	UBR5	INVS
EIF4BP4	UPB1	IP6K2
EIF4E3	UPK1B	IPO11
ELAVL4	UQCC	IPP
ELK3	UQCRBP2	IQCJ
ELK4	UQCRFS1P3	IQCJ-SCHIP1
ELMOD1	URB2	IQGAP2
ELOVL5	USP17L3	IQSEC3
ELOVL6	USP24	IQUB
ELP4	VDAC1P10	IRAK1BP1
ELTD1	VIT	ISPD
EMB	VN1R47P	ITFG1
EMBP1	VN1R54P	ITGA1
EMCN	VOPP1	ITGA9
EML1	VSTM2B	ITGAM
ENDOD1	VWC2	ITGB6
ENTHD1	VWDE	ITPR1
ENTPD1	WARS2	ITPR2
ENTPD5	WDFY3	ITSN1
ENTPD6	WDR16	JAKMIP2
EPB41L1	WDR63	JMJD1C
EPB41L3	WDR96	JMY
EPC1	WNT3A	KALRN
EPC2	XKR6	KAT2B
EPG5	ZCCHC24	KAT6B
EPHB6	ZHX3	KCNA3
EPN1	ZNF311	KCNAB1
EPRS	ZNF486	KCNB2
EPS8	ZNF569	KCNC2
EPSTI1	ZNF672	KCND2
EPT1	ZNF729	KCND3
ERAP2	ZNF738	KCNH1
ERC1	ZNF788	KCNH5
ERCC4	ZNF83	KCNH7
ERI1	ZNRD1-AS1	KCNH8

ERO1L
ERO1LB
ERP44
ESCO1
ESF1
ESRP1
ESRRB
ETFA
ETS1
ETV1
ETV5
EXD1
EXD3
EXOC6
EXOSC7
EXPH5
EXT1
EXT2
EYA1
F11R
F2R
FABP7
FADS1
FADS2
FAF1
FAF2
FAM108C1
FAM114A2
FAM115A
FAM118B
FAM120A
FAM131B
FAM134B
FAM13B
FAM149B1
FAM151B
FAM154B
FAM159B
FAM160A1
FAM162A
FAM163A
FAM168A
FAM169A
FAM169B
FAM171A1
ZRANB1
tAKR
KCNIP1
KCNIP4
KCNK13
KCNK2
KCNMA1
KCNMB2
KCNN2
KCNQ5
KCNT2
KCTD16
KDM4B
KDM4C
KDM5B
KDM6A
KHDRBS2
KHDRBS3
KIAA0146
KIAA0564
KIAA0825
KIAA1109
KIAA1217
KIAA1239
KIAA1244
KIAA1324L
KIAA1486
KIF13A
KIF26B
KIF5C
KIF6
KIFAP3
KIRREL
KIRREL3
KITLG
KLHL12
KLHL32
KRT18P28
KSR2
L3MBTL3
L3MBTL4
LACE1
LAMA2
LAMB4
LAMC1
LARGE
LARP1

FAM174A	LARS2
FAM174B	LCLAT1
FAM175B	LDB2
FAM176A	LEKR1
FAM177B	LEPR
FAM179B	LEPREL1
FAM188A	LGALS8
FAM189A1	LGR5
FAM189A2	LGR6
FAM205CP	LHFPL3
FAM208A	LHX8
FAM208B	LIN9
FAM20B	LINC00032
FAM24B	LINC00271
FAM24B-CUZD1	LINC00273
FAM32E	LINC00299
FAM46A	LINC00340
FAM46C	LINC00466
FAM49A	LINC00467
FAM49B	LINC00473
FAM55A	LINC00478
FAM58BP	LINGO2
FAM59A	LIX1
FAM5B	LMBRD1
FAM73A	LMCD1
FAM81B	LOC100124692
FAM82B	LOC100127907
FAM91A1	LOC100128095
FAM98B	LOC100128811
FAR2	LOC100129100
FARP1	LOC100129620
FAT2	LOC100130039
FAT4	LOC100130260
FBXL13	LOC100131060
FBXL14	LOC100131208
FBXL5	LOC100132247
FBXO15	LOC100132482
FBXO16	LOC100132507
FBXO47	LOC100132570
FBXW11	LOC100132617
FBXW2	LOC100132698
FBXW8	LOC100132733
FCGBP	LOC100132858
FCRL2	LOC100133097
FCRL5	LOC100133112

FCRLA	LOC100133203
FECH	LOC100133308
FERMT1	LOC100188947
FERMT2	LOC100287651
FGD4	LOC100287663
FGF13	LOC100287814
FGF2	LOC100287944
FGFR2	LOC100288268
FHL5	LOC100288428
FHOD3	LOC100288470
FIG4	LOC100288728
FIGN	LOC100288798
FILIP1	LOC100289178
FKTN	LOC100289211
FLG2	LOC100289350
FLI1	LOC100302640
FLJ33630	LOC100419319
FLJ35024	LOC100421094
FLJ37644	LOC100421363
FLJ41278	LOC100422478
FLJ42280	LOC100422737
FLJ43663	LOC100423044
FLJ43860	LOC100462648
FLJ46257	LOC100505566
FLT1	LOC100505718
FLVCR2	LOC100505821
FMO1	LOC100505836
FMO10P	LOC100505872
FMO11P	LOC100505875
FMO7P	LOC100505933
FN1	LOC100506022
FNIP2	LOC100506023
FNTB	LOC100506032
FOLH1	LOC100506128
FOLH1B	LOC100506187
FOLR4	LOC100506207
FONG	LOC100506246
FOXG1	LOC100506267
FOXN2	LOC100506288
FOXQ1	LOC100506867
FRAS1	LOC100506869
FREM1	LOC100506944
FRG2C	LOC100507115
FRK	LOC100507145
FRMD6	LOC100507193

FRMPD4	LOC100507221
FRY	LOC100507421
FSD1L	LOC100507466
FSHR	LOC100507633
FSIP1	LOC100507651
FTH1P20	LOC100508120
FTH1P21	LOC100533667
FUT9	LOC100533705
FXR1	LOC100533707
GAB2	LOC100616530
GAB4	LOC100628307
GABPA	LOC100631248
GABPB1	LOC100652810
GABRA1	LOC100652887
GABRA4	LOC100652924
GABRA5	LOC100652953
GABRR3	LOC100652979
GAL3ST2	LOC200149
GALK2	LOC283033
GALNT1	LOC283194
GALNT11	LOC283547
GALNT8	LOC283867
GALNTL4	LOC284294
GALNTL5	LOC284395
GAN	LOC285423
GAPDHP68	LOC285692
GAPVD1	LOC285768
GAS2L3	LOC339529
GATM	LOC339788
GBP1	LOC340357
GBX1	LOC344595
GCC2	LOC345576
GCFC1	LOC348840
GCNT1	LOC388692
GCNT2	LOC388996
GCOM1	LOC400794
GDE1	LOC400986
GDPD1	LOC401164
GDPD4	LOC440040
GDPD5	LOC440157
GEMIN8P2	LOC641298
GFM1	LOC642236
GGNBP2	LOC642441
GIPC2	LOC642924
GJB5	LOC643255

GK5	LOC643339
GKN2	LOC643542
GLB1	LOC643579
GLCE	LOC643650
GLO1	LOC643723
GLRA2	LOC643827
GLRB	LOC645166
GLRX2	LOC645206
GLULP4	LOC647107
GLYATL1P3	LOC647211
GNA14	LOC647946
GNAI2P1	LOC653557
GNAL	LOC654342
GNAO1	LOC727751
GNB4	LOC728034
GNG2	LOC728558
GNN	LOC728587
GNPDA1	LOC728611
GNPDA2	LOC728755
GNRHR2	LOC728875
GORASP2	LOC729178
GPATCH4	LOC729444
GPBP1	LOC91948
GPC3	LPGAT1
GPD2	LPHN2
GPHB5	LPHN3
GPR126	LPP
GPR135	LPPR1
GPR137C	LRBA
GPR155	LRGUK
GPR160	LRP1B
GPR89A	LRRC16A
GPRC5A	LRRC6
GPT2	LRRC69
GPX1P2	LRRC7
GRAMD1C	LRRC9
GRB14	LRRIQ3
GREB1L	LRRK2
GRIA2	LRRTM4
GRIA3	LSAMP
GRK5	LUZP2
GRM3	LY86-AS1
GRXCR1	LYRM4
GSTCD	LYST
GSTK1	MACF1

GSTO2	MACROD2
GSTO3P	MAGI1
GTF2E1	MAGI1-IT1
GTF2E2	MAGI2
GTF2I	MAGI3
GTF3C6	MAMDC2
GTSF1	MAML2
GUCY1B2	MAN1A2
GUCY1B3	MAN2A1
GUCY2GP	MAP2
GULP1	MAP2K5
GXYLT1	MAP3K13
GYS2	MAP3K5
H2AFY	MAP4K4
HAPLN2	MAP7
HAT1	MAPK10
HAUS3	MAPK14
HDAC4	MAPKAP1
HDAC5	MARCH3
HDDC2	MASP1
HEATR5A	MAST2
HEATR5B	MAST4
HEATR7B2	MB21D2
HEBP2	MBD5
HEG1	MBNL1
HELLS	MCC
HERC2	MCF2L2
HERC4	MCL1
HEY2	MCTP1
HFE2	MCU
HGD	MDFIC
HGF	MDGA2
HHEX	MECOM
HHLA2	MED12L
HIATL2	MEGF10
HIBCH	MEIS2
HIGD1AP12	MEMO1
HIP1	MET
HIPK3	METTL8
HIST1H2BF	MFSD6
HIST1H4D	MGAT4C
HIST1H4PS1	MGC27382
HIST2H2AB	MGC34034
HIVEP1	MIPOL1
HLCS	MIR100HG

HMBOX1
HMGA1
HMGB1P13
HMGB3P2
HMGN1P38
HMOX2
HNMT
HNRNPA1P31
HNRNPC
HOMER1
HOMER2
HORMAD1
HORMAD2
HPD
HPDL
HPGDS
HSD17B13
HSD17B3
HSD17B4
HSD17B6
HSP90AB2P
HSPA14
HSPA4L
HTR2A
HYAL4
HYALP1
IARS
IBSP
ICA1L
IDE
IDH1
IFI16
IFNA6
IFNGR1
IFT57
IFT88
IGF1
IGF2BP1
IGF2BP2
IGF2R
IGFBP2
IGHV1OR15-4
IGHV1OR21-1
IKZF1
IL17F
MIR4477A
MIR4477B
MIR4500HG
MIR4719
MIRLET7BHG
MITF
MKL1
MKL2
MKLN1
MLL3
MLLT3
MMP16
MMS22L
MNAT1
MOB3B
MORC1
MOXD1
MPPED2
MSH3
MSMP
MSRA
MTDH
MTHFD1
MTHFD1L
MTMR2
MTOR
MTSS1
MUC20
MUSK
MUTED-TXNDC5
MYCBP2
MYH15
MYLK
MYO16
MYO1B
MYO1D
MYO3A
MYO3B
MYO5A
MYO5BP3
MYO6
MYOCD
MYOF
MYRIP
MYT1L

IL18RAP
IL19
IL1B
IL1R2
IL1RAPL1
IL1RAPL2
IL23R
IL24
IL31RA
IL6R
IL7
IL9
IMP4
IMPG2
INADL
INO80C
INTS2
INTS4
INTS4L1
INTS6
INTS8
IPCEF1
IPO7
IPO9
IQCA1
IQCB1
IQCF6
IQCH
IQGAP1
IQGAP3
IRS1
ISM1
ISX
ITCH
ITGA4
ITGA6
ITGA8
ITGB3
ITGB3BP
ITGB5
ITGBL1
ITIH5
ITLN2
ITSN2
IVL
NAALADL2
NALCN
NARS2
NAV1
NAV2
NAV3
NBAS
NBEA
NBEAP1
NBEAP3
NBEAP4
NBPF10
NBPF13P
NBPF14
NBPF15
NBPF17P
NCALD
NCAM2
NCK1
NCKAP5
NCOA1
NCOA2
NCOA3
NCOR1
NDST3
NDST4
NDUFAF2
NEBL
NECAB1
NEDD4L
NEDD9
NEGR1
NEK10
NEK11
NEK7
NELL1
NELL2
NEO1
NF1
NF1P6
NFASC
NFIA
NFIB
NFIX
NFKB1

JAG1	NHSL1
JAK1	NID1
JARID2	NIN
JAZF1	NKAIN1P1
JHDM1D	NKAIN2
JKAMP	NKAIN3
JPH1	NLGN1
KANK1	NLK
KAT6A	NMNAT2
KAZN	NOL4
KBTBD12	NOS1AP
KCNB1	NOTCH2
KCNJ16	NOTCH2NL
KCNMB4	NOX4
KCNN3	NPAS3
KCNQ1	NPHP3-AS1
KCNQ3	NR3C1
KCTD8	NR5A2
KDM3A	NRCAM
KDM5A	NRG1
KDR	NRG2
KIAA0087	NRG3
KIAA0232	NRP1
KIAA0247	NRXN1
KIAA0319	NRXN3
KIAA0355	NSL1
KIAA0391	NSMCE2
KIAA0528	NT5C2
KIAA0556	NT5DC1
KIAA0586	NTM
KIAA0748	NTN4
KIAA0922	NTNG1
KIAA1009	NTNG2
KIAA1199	NTRK2
KIAA1267	NTRK3
KIAA1324	NUBPL
KIAA1328	NUP133
KIAA1370	NUP210L
KIAA1377	NVL
KIAA1407	NXPH1
KIAA1429	NXPH2
KIAA1432	OCA2
KIAA1468	ODZ2
KIAA1598	ODZ3
KIAA1731	OFCC1

KIAA1755
KIAA1797
KIAA1826
KIAA2026
KIDINS220
KIF11
KIF15
KIF16B
KIF18A
KIF1B
KIF5B
KIN
KLHL14
KLHL2
KLHL20
KLHL24
KLHL25
KLHL29
KLHL3
KLK13
KLK15
KLK3
KLKP1
KLRD1
KMO
KNTC1
KPNA1
KPNA4
KPNA5
KRAS
KRIT1
KRT18P2
KRT18P29
KRT18P46
KRT20
KRT73
KRTAP4-6
KTN1
LAMA1
LAMA3
LAMA4
LAMB1
LAMC2
LAMP3
LAPTM4B
OLFM3
OMA1
OPCML
OPRM1
OR10G7
OR11H12
OR11H13P
OR11H2
OR2A20P
OR2L13
OR2T5
OR4G6P
OR4T1P
OR6W1P
OR8B12
ORC5
OSBPL10
OSBPL8
OTOGL
OTUD7B
OXCT1
OXR1
PACRG
PAG1
PAICSP4
PAK1
PAK7
PALLD
PALM2-AKAP2
PAM
PAPPA2
PARD3
PARD3B
PARK2
PARP8
PBRM1
PBX1
PBX3
PCCA
PCDH15
PCDH9
PCDHA@
PCDHA1
PCDHA10
PCDHA11

LCN15	PCDHA12
LCOR	PCDHA13
LCORL	PCDHA2
LCP1	PCDHA3
LEMD1	PCDHA4
LEMD3	PCDHA5
LEPROTL1	PCDHA6
LIG3	PCDHA7
LIMA1	PCDHA8
LIMCH1	PCDHA9
LIMS1	PCDHB@
LIN28B	PCGEM1
LIN52	PCMTD1P2
LIN7A	PCNX
LINC00189	PCNXL2
LINC00293	PCSK2
LINC00461	PCSK5
LINC00470	PDE10A
LINC00476	PDE11A
LINC00483	PDE1A
LINC00486	PDE1C
LINC00487	PDE3A
LIPC	PDE3B
LIPI	PDE4B
LIPT1	PDE4D
LMBR1	PDE4DIP
LMBRD2	PDE5A
LMLN	PDE6A
LMNB1	PDE7B
LMO3	PDGFC
LMO7	PDGFD
LMTK2	PDLIM5
LMX1A	PDSS2
LNPEP	PDXDC1
LNX2	PDZD2
LOC100113374	PDZD8
LOC100127905	PDZRN3
LOC100127989	PDZRN4
LOC100128007	PEAK1
LOC100128154	PENK
LOC100128412	PEX5L
LOC100128497	PGCP
LOC100128505	PGM2L1
LOC100128590	PGM5
LOC100128898	PHACTR1

LOC100128909	PHACTR2
LOC100128974	PHC3
LOC100128993	PHLDB2
LOC100129009	PHLPP2
LOC100129195	PHTF2
LOC100129402	PID1
LOC100129413	PIK3AP1
LOC100129422	PIK3C2B
LOC100129734	PIK3C2G
LOC100129827	PIK3CA
LOC100129858	PIK3CB
LOC100129997	PIK3R4
LOC100130018	PION
LOC100130097	PIP4K2A
LOC100130155	PITPNC1
LOC100130299	PKHD1
LOC100130316	PKHD1L1
LOC100130331	PKIB
LOC100130466	PKN2
LOC100130522	PLA2G4A
LOC100130535	PLCB1
LOC100130579	PLCB4
LOC100130691	PLCH1
LOC100130801	PLCL1
LOC100131013	PLCXD2
LOC100131080	PLCXD3
LOC100131131	PLD1
LOC100131234	PLD5
LOC100131284	PLEKHG1
LOC100131520	PLEKHG4B
LOC100131842	PLEKHM3
LOC100131852	PLOD2
LOC100132249	PLXDC2
LOC100132310	PLXNA4
LOC100132352	PNLIPRP3
LOC100132501	POLR3B
LOC100132626	POT1
LOC100132699	POTEG
LOC100132762	POU2F1
LOC100132891	POU6F2
LOC100133023	PPAPDC1B
LOC100133047	PPFIA2
LOC100192378	PPFIBP2
LOC100194426	PPIP5K2
LOC100271702	PPM1D

LOC100271722	PPM1E
LOC100286997	PPM1L
LOC100287049	PPP1R12B
LOC100287063	PPP1R14BP5
LOC100287225	PPP1R14C
LOC100287313	PPP1R15B
LOC100287366	PPP1R1C
LOC100287497	PPP1R9A
LOC100287723	PPP2R2B
LOC100287856	PPP3CA
LOC100287912	PPP6R3
LOC100287966	PPPDE1
LOC100288102	PREX2
LOC100288437	PRH1-PRR4
LOC100288503	PRIM2
LOC100288637	PRKACB
LOC100289144	PRKCA
LOC100289207	PRKCB
LOC100289388	PRKCE
LOC100289416	PRKD1
LOC100289473	PRKG1
LOC100418712	PRKG2
LOC100418822	PRLR
LOC100418888	PROS1
LOC100418983	PROX1
LOC100419324	PRR16
LOC100419553	PRRC2C
LOC100419615	PRSS16
LOC100419616	PRUNE2
LOC100419685	PSD3
LOC100419780	PSMA8
LOC100419911	PSMD4
LOC100420103	PTGER3
LOC100420149	PTH2R
LOC100420216	PTN
LOC100420289	PTPN14
LOC100420424	PTPRC
LOC100420430	PTPRD
LOC100420502	PTPRG
LOC100420580	PTPRJ
LOC100420668	PTPRK
LOC100420926	PTPRM
LOC100420948	PTPRN2
LOC100420968	PTPRO
LOC100421439	PTPRQ

LOC100421509	PTPRR
LOC100421705	PTPRT
LOC100421863	PTPRZ1
LOC100422013	PUM1
LOC100422241	PVT1
LOC100422352	PXDNL
LOC100422564	RAB27B
LOC100422606	RAB3C
LOC100505487	RAB3GAP2
LOC100505498	RABGAP1L
LOC100505515	RABL3
LOC100505547	RAD51B
LOC100505572	RAD54B
LOC100505583	RAI14
LOC100505650	RALGAPA1
LOC100505659	RALGPS1
LOC100505663	RALGPS2
LOC100505668	RALYL
LOC100505678	RANBP17
LOC100505695	RANBP9
LOC100505703	RAPGEF4
LOC100505711	RAPGEF5
LOC100505794	RARB
LOC100505799	RASA1
LOC100505811	RASAL2
LOC100505832	RASGRF2
LOC100505854	RASSF3
LOC100505869	RAVER2
LOC100505870	RB1CC1
LOC100505902	RBBP5
LOC100505918	RBFOX1
LOC100505930	RBM47
LOC100505940	RBMS1
LOC100505964	RBMS3
LOC100505966	RC3H1
LOC100505985	RCOR3
LOC100505987	RCSD1
LOC100505989	REEP1
LOC100506049	RELN
LOC100506051	RERE
LOC100506092	RERG
LOC100506154	REXO1L1
LOC100506196	RFTN1
LOC100506220	RFWD2
LOC100506229	RFX4

LOC100506304	RFX6
LOC100506305	RGL1
LOC100506342	RGNEF
LOC100506354	RGS13
LOC100506368	RGS3
LOC100506380	RGS7
LOC100506393	RGSL1
LOC100506409	RHBDD1
LOC100506422	RHOU
LOC100506478	RHPN2P1
LOC100506528	RICTOR
LOC100506534	RIMS1
LOC100506810	RIMS2
LOC100506860	RIT2
LOC100506929	RNF115
LOC100506994	RNF145
LOC100507043	RNF180
LOC100507059	RNU1-5
LOC100507065	ROBO1
LOC100507127	ROBO2
LOC100507186	ROCK1P1
LOC100507233	ROR1
LOC100507367	RORA
LOC100507377	RORB
LOC100507381	ROS1
LOC100507389	RPAP2
LOC100507429	RPL14P1
LOC100507433	RPL23AP7
LOC100507443	RPL39P11
LOC100507452	RPL7P37
LOC100507474	RPRD2
LOC100507540	RPS6KA2
LOC100507561	RPS6KC1
LOC100507582	RSPO2
LOC100507613	RSPO3
LOC100507616	RSRC1
LOC100508736	RTKN2
LOC100509105	RUNDC3B
LOC100526771	RUNX1T1
LOC100526838	RYR2
LOC100533655	RYR3
LOC100533721	SAMD12
LOC100630917	SAMD12-AS1
LOC100631247	SAMD3
LOC100652734	SATB1

LOC100652735	SATB2
LOC100652766	SBF2
LOC100652767	SCAPER
LOC100652780	SCFD2
LOC100652796	SCHIP1
LOC100652812	SCMH1
LOC100652813	SCML4
LOC100652815	SCN1A
LOC100652823	SCN2A
LOC100652827	SCN9A
LOC100652834	SCYL3
LOC100652838	SDCCAG8
LOC100652843	SDK1
LOC100652863	SDK2
LOC100652908	SEC22B
LOC100652919	SEC23A
LOC120364	SEMA3A
LOC120824	SEMA3C
LOC134997	SEMA3D
LOC136157	SEMA3E
LOC144481	SEMA5A
LOC151658	SEMA6D
LOC152225	SENP5
LOC153469	SENP7
LOC154092	SESTD1
LOC283038	SETD2
LOC283177	SFMBT2
LOC283585	SGCD
LOC283688	SGCE
LOC283711	SGCZ
LOC284788	SGIP1
LOC285419	SGK1
LOC285501	SGK3
LOC285577	SGMS1
LOC285697	SGPP2
LOC285758	SH3GL2
LOC285965	SH3RF2
LOC286135	SH3RF3
LOC286149	SHANK2
LOC338758	SHCBP1L
LOC339685	SHISA6
LOC339894	SHISA9
LOC339975	SIGLEC30P
LOC340515	SKAP1
LOC344887	SKAP2

LOC375190	SLC12A8
LOC388553	SLC13A1
LOC388630	SLC13A3
LOC388734	SLC14A2
LOC389740	SLC16A10
LOC389935	SLC16A12
LOC390306	SLC1A1
LOC390311	SLC1A2
LOC391040	SLC20A2
LOC391600	SLC22A3
LOC392285	SLC24A2
LOC399815	SLC24A3
LOC400654	SLC25A21
LOC400655	SLC26A3
LOC402096	SLC2A12
LOC440067	SLC2A13
LOC440434	SLC30A7
LOC440742	SLC35F1
LOC440993	SLC35F3
LOC441009	SLC35F4
LOC441899	SLC38A6
LOC441907	SLC41A1
LOC441915	SLC41A2
LOC442006	SLC41A3
LOC442320	SLC44A5
LOC442421	SLC4A7
LOC442459	SLC5A11
LOC554201	SLC9A9
LOC613126	SLCO1B1
LOC641379	SLCO5A1
LOC642550	SLIT2
LOC642587	SLIT3
LOC643126	SLMAP
LOC643634	SMAP1
LOC644135	SMC5
LOC644159	SMYD3
LOC644265	SNAP91
LOC644277	SNAR-B1
LOC644335	SNCAIP
LOC644425	SNRPN
LOC644632	SNTB1
LOC644662	SNTG1
LOC644754	SNTG2
LOC644768	SNX13
LOC644838	SNX18P10

LOC645266	SNX18P21
LOC645405	SNX24
LOC645434	SNX27
LOC645503	SNX29
LOC646012	SNX30
LOC646044	SOAT1
LOC646213	SOBP
LOC646324	SORCS1
LOC646388	SORCS3
LOC646555	SOX5
LOC646626	SOX6
LOC646709	SP100
LOC646719	SP140
LOC646730	SPAG16
LOC647012	SPAG17
LOC647051	SPAG9
LOC647150	SPATA1
LOC647253	SPATA17
LOC647532	SPATA6
LOC648809	SPATA9
LOC649024	SPATS2L
LOC649166	SPECC1
LOC652423	SPG20
LOC727924	SPHKAP
LOC728095	SPOCK1
LOC728323	SPOCK3
LOC728377	SPON1
LOC728602	SPRR3
LOC728606	SRFBP1
LOC728723	SRGAP2
LOC728730	SRGAP2P1
LOC728811	SRGAP2P2
LOC728989	SRGAP3
LOC729222	SRP9
LOC729316	SSBP2
LOC729506	SSH2
LOC729658	ST13P19
LOC729817	ST18
LOC729911	ST3GAL3
LOC729941	ST6GAL1
LOC729950	ST6GALNAC3
LOC730021	ST6GALNAC5
LOC730076	ST7
LOC730091	ST8SIA6
LOC731308	STAG1

LOC93463	STAU2
LOH12CR1	STK3
LONP2	STK31
LONRF2	STK38L
LOXHD1	STK39
LPA	STK4
LPAR1	STON2
LPAR3	STRBP
LPIN2	STRN
LPPR4	STRN3
LPPR5	STX7
LRCH1	STX8
LRCH3	STXBP4
LRFN2	STXBP5
LRFN5	STXBP5L
LRIG1	STXBP6
LRIG3	SUCLG2
LRP12	SULF1
LRP2	SULT1E1
LRP6	SUPT3H
LRRC1	SUSD4
LRRC17	SV2B
LRRC28	SV2C
LRRC36	SVEP1
LRRC49	SWAP70
LRRC4C	SWT1
LRRC8B	SYBU
LRRC8D	SYCP2L
LRRCC1	SYN2
LRRFIP1	SYN3
LRRFIP2	SYNDIG1
LRRIQ1	SYNE1
LRRTM3	SYNE2
LTBP1	SYNPR
LTF	SYPL1
LUC7L2	SYT1
LY75	SYT14
LY75-CD302	SZT2
LYN	TACR1
LYNX1	TAF3
LYPD6B	TAG
LYPLA1	TANC2
LYZ	TARBP1
LYZL4	TBC1D22B
MAGOHB	TBC1D3F

MAL2	TBC1D5
MALT1	TBCE
MAML3	TBL1XR1
MANBA	TBPL1
MANBAL	TBX15
MAOA	TBX19
MAP2K1	TBX3
MAP2K4	TCERG1
MAP3K1	TCF12
MAP3K4	TCF4
MAP4	TCF7L2
MAP4K5	TDRD1
MAP6	TDRD5
MAP9	TEKT4P2
MAPK1	TFDP2
MAPK6	TFEC
MAPK8IPP	TFPI
MAPRE2	THEMIS
MAPT	THRAP3
MARCH1	THRB
MARCH10	THSD4
MARCH11	THSD7A
MARCH6	THSD7B
MARCH8	TIMM8AP1
MARK1	TLE4
MARK2P9	TLL1
MAT2B	TMC1
MATN2	TMC5
MATR3	TMCC1
MAX	TMCO1
MBLAC2	TMEFF1
MBNL3	TMEFF2
MBOAT2	TMEM108
MBP	TMEM117
MBTD1	TMEM131
MBTPS1	TMEM132B
MCCC2	TMEM132D
MCM6	TMEM135
MCM9	TMEM161B
MCOLN2	TMEM163
MCPH1	TMEM196
MCTP2	TMEM2
MDFI	TMEM232
ME1	TMEM233
ME2	TMEM45A

ME3	TMEM55A
MED13	TMTC1
MED13L	TMTC2
MED17	TNC
MED23	TNFAIP6
MED27	TNIK
MEF2A	TNNI3K
MEF2C	TNPO3
MEGF11	TNR
MEGF9	TNRC6B
MEIS1	TOM1L2
MELK	TOP2B
MEP1A	TOX
MERTK	TP63
METAP2	TPD52
METTL10	TPK1
METTL15	TPRG1
MFHAS1	TPTE
MFN1	TRA
MGA	TRAF3IP2-AS1
MGAM	TRAK2
MGAT4A	TRANK1
MGC21881	TRB
MGMT	TRDN
MGST1	TREM1
MIA3	TRHDE
MIB1	TRIM33
MICAL2	TRIM36
MICU1	TRIO
MIMT1	TRMT1L
MIPEP	TRNAL10
MIR1202	TRPC4
MIR1263	TRPC6
MIR3118-2	TRPM3
MIR3118-3	TRPM6
MIR3144	TRPM8
MIR31HG	TSHZ2
MIR3648	TSNAX-DISC1
MIR3686	TTC13
MIR3687	TTC21B
MIR3688-1	TTC27
MIR4275	TTC28
MIR4300	TTLL11
MIR4424	TTN
MIR4447	TXNRD1

MIR4514	TYW1B
MIR4523	UAP1
MIR548G	UBE2CBP
MIR876	UBE2E2
MKI67	UBE2H
MKX	UGT2B15
MLANA	UHMK1
MLK7-AS1	UIMC1
MLL5	ULK4
MLLT10	UNC13C
MLLT4	UNC5D
MLPH	UNC79
MME	UNC80
MMP15	UPP2
MMP20	USH2A
MND1	USP17L2
MON1B	UTRN
MON2	UVRAG
MORF4L1	VAV3
MOSC1	VCAN
MOV10L1	VEPH1
MPP7	VGLL4
MR1	VMP1
MRP63P7	VN1R7P
MRPL42	VPS13A
MRPL42P3	VPS13B
MRPS21P2	VPS13D
MRPS27	VPS45
MRPS31P1	VPS8
MRPS33P4	VTA1
MRPS5P3	VTI1A
MRPS6	VWA3B
MRS2P1	WBSCR17
MRVI1	WDR17
MS4A5	WDR49
MS4A6E	WDR64
MS4A7	WDR70
MS4A8B	WDR72
MSH4	WDR78
MSI2	WIF1
MSMB	WWC1
MSR1	WWOX
MSRB3	WWP1
MSTN	WWP2
MT1P2	WWTR1

MTERFD1	XIRP2
MTF2	XKR4
MTHFD2L	XPR1
MTMR12	XRCC4
MTMR3	XRN1
MTMR9	XYLT1
MTR	YEATS2
MTUS1	YWHAE
MTUS2	ZBBX
MTX2	ZBTB1
MUTED	ZBTB16
MYB	ZBTB20
MYBL1	ZBTB38
MYCT1	ZC3H11A
MYH10	ZCCHC7
MYH9	ZCWPW2
MYLK-AS1	ZDHHC14
MYLK4	ZFPM2
MYO10	ZFR
MYO15B	ZFYVE9
MYO1E	ZMAT4
MYO5B	ZMYM4
MYO5BP2	ZNF248
MYO9A	ZNF267
MYOT	ZNF277
MYPN	ZNF33B
MYSM1	ZNF365
N4BP2L2	ZNF385B
NAA35	ZNF385D
NAB1	ZNF407
NAIP	ZNF503-AS1
NANOGP3	ZNF507
NAP1L1	ZNF521
NAP1L4	ZNF595
NAPEPLD	ZNF644
NARG2	ZNF670
NASPP1	ZNF670-ZNF695
NBEAL1	ZNF695
NBEAP6	ZNF704
NBPF9	ZNF717
NCAM1	ZNF804A
NCAPG2	ZNF804B
NCKAP1	ZPBP
NCL	ZSWIM5
NCOR1P2	ZSWIM6

NDFIP1	ZZZ3
NDUFB8P3	
NDUFS4	
NEB	
NEDD4	
NEFHP1	
NEK1	
NEK9	
NET1	
NETO1	
NETO2	
NEU3	
NEURL	
NFAT5	
NFATC3	
NFYC	
NGEF	
NHEJ1	
NHLH2	
NIM1	
NIPBL	
NIPSNAP3A	
NIPSNAP3B	
NKIRAS1	
NLN	
NLRC3	
NLRP14	
NLRP3	
NMD3	
NMD3P1	
NME1-NME2	
NME7	
NME9	
NMNAT3	
NMUR2	
NNT	
NOL10	
NOP58	
NOTUM	
NOX3	
NPAT	
NPBWR1	
NPEPPS	
NPFFR2	
NPHP3-ACAD11	

NPM1P21
NPNT
NR1D2
NR1H4
NR3C2
NR6A1
NRF1
NRG4
NSMAF
NSUN6
NSUN7
NUCKS1
NUDCD1
NUDT3
NUDT4P1
NUDT6
NUDT9
NUMB
NUP153
NUP160
NUP205
NUP93
NUP98
OAZ3
OC90
ODC1
ODZ4
OGFOD1
OLA1
OPN1SW
OPN3
OPRK1
OR10D3
OR10D5P
OR10J8P
OR10J9P
OR13D1
OR14A2
OR14C36
OR14K1
OR2A1
OR2A25
OR2A3P
OR2A42
OR2AJ1

OR2AS2P
OR2B4P
OR2E1P
OR2G3
OR2M5
OR2M7
OR2T12
OR2T33
OR2T7
OR2U2P
OR4A17P
OR4C50P
OR4K8P
OR4V1P
OR51G1
OR51V1
OR52A5
OR56A1
OR5B1P
OR5BK1P
OR5F1
OR5G3
OR5J1P
OR5K1
OR5M4P
OR5P3
OR6C72P
OR6K5P
OR6M1
OR6M3P
OR6V1
OR7E41P
OR8G7P
OR8T1P
OR9A2
OR9Q1
ORMDL1
OSBPL11
OSBPL1A
OSBPL6
OSBPL9
P4HA1
PA2G4P2
PABPC1P13
PACSIN2

PAH
PALM2
PAPD4
PAPPA
PAPSS1
PARG
PARK7
PARN
PARP11
PARP12
PARP15
PAWR
PAX3
PAXIP1
PBX2P1
PC
PCA3
PCBP2
PCBP3
PCDH11X
PCDH7
PCDH8P1
PCDHAC1
PCDHAC2
PCDHACT
PCDHGA1
PCDHGA10
PCDHGA11
PCDHGA12
PCDHGA2
PCDHGA3
PCDHGA4
PCDHGA5
PCDHGA6
PCDHGA7
PCDHGA8
PCDHGA9
PCDHGB1
PCDHGB2
PCDHGB3
PCDHGB4
PCDHGB5
PCDHGB6
PCDHGB7
PCLO

PCM1
PCMTD1
PCOLCE2
PCSK6
PDCD10
PDCL2
PDE7A
PDE8B
PDE9A
PDIA5
PDS5B
PELI2
PER1
PEX1
PEX10
PGAP1
PGBD3P1
PGBD3P4
PGCP1
PHF11
PHF14
PHF15
PHIP
PHKB
PHKBP2
PHLPP1
PHOSPHO2-KLHL23
PHYHIPL
PI15
PIAS1
PIAS2
PICALM
PIEZO2
PIGP
PIK3C2A
PIK3C3
PIK3R1
PIK3R6
PIKFYVE
PIP5K1B
PJA2
PKD2
PKP1
PKP2
PKP4

PLAC1L
PLAGL1
PLB1
PLBD1
PLCG2
PLCL2
PLEKHA5
PLG
PLS1
PLXNA2
PLXNC1
PM20D1
POC1A
POGLUT1
POGZ
POLA2
POLQ
POLR1A
POM121L10P
POMC
POTEKP
POU5F1P2
PPAPDC1A
PPARG
PPARGC1B
PPFIBP1
PPHLN1
PPIAL4D
PPIEL
PPIL3
PPIL6
PPM1A
PPM1H
PPME1
PPP1CB
PPP1R12A
PPP1R13B
PPP1R3D
PPP2CA
PPP2R2A
PPP2R5A
PPP2R5C
PPP2R5E
PPP4R4
PPP6C

PPYR1
PRDM1
PRDM4
PRDM5
PRELID2
PREP
PREX1
PRICKLE1
PRICKLE2
PRIM1
PRKAA2
PRKAG2
PRKAR2A
PRKCH
PRKCQ
PRKRIR
PRMT6
PROSP
PRPF18
PRPF39
PRPF40A
PRR15L
PRR23B
PRR5L
PRRC1
PRSS38
PRTFDC1
PRTG
PSAT1P1
PSD4
PSMA1
PSMB1
PSMB3P2
PSMB7
PSMD14
PSMD5
PSME4
PSMG2
PTBP2
PTCHD3
PTER
PTGDR
PTGFR
PTGIS
PTGR1

PTK2
PTK2B
PTK7
PTMAP8
PTPDC1
PTPLA
PTPN1
PTPN12
PTPN13
PTPN2
PTPN21
PTPN4
PTPRA
PTPRB
PTPRE
PUM2
PURG
PXDN
PYGB
PYHIN1
QKI
QSER1
R3HDM1
R3HDM2
RAB10
RAB11FIP2
RAB31
RAB38
RAB3B
RAB3GAP1
RAB6B
RAB7A
RAD21L1
RAD23B
RAD54L2
RAF1
RALBP1
RALGAPA2
RALGAPB
RAMP1
RANBP3L
RAP1GAP2
RAPGEF6
RARRES1
RARRES2

RARS
RASEF
RASGEF1A
RASGEF1C
RASGRP3
RASSF10
RASSF8
RBBP8
RBFOX2
RBL2
RBM20
RBM33
RBM45
RBM46
RBM6
RBMS2
RBPMS
RBX1
RCAN2
RCBTB1
RCC2P5
RDX
RECK
REEP3
REG1A
REG1B
REG1P
REG3A
REPS1
RERGL
REV1
REV3L
RFC3
RFT1
RFTN2
RFX7
RGS20
RGS22
RGS6
RGS7BP
RGS8
RHAG
RHOBTB1
RHOBTB3
RHOJ

RHPN2
RIC8B
RIN2
RINT1
RIOK3
RLF
RLIMP3
RMI1
RMST
RNASEH1P3
RNASEH2B
RNF103-VPS24
RNF13
RNF130
RNF144A
RNF144B
RNF150
RNF157
RNF169
RNF182
RNF2
RNF20
RNF38
RNU7-2P
RNU7-51P
ROCK1
ROCK2
ROD1
ROR2
RPF2
RPGRIP1L
RPH3A
RPL10P10
RPL13AP14
RPL15P20
RPL17P23
RPL18P2
RPL18P3
RPL19P14
RPL19P17
RPL21P106
RPL21P11
RPL21P116
RPL21P16
RPL21P2

RPL21P25
RPL21P39
RPL21P67
RPL21P75
RPL21P96
RPL22L1
RPL22P7
RPL23AP2
RPL23AP56
RPL23AP68
RPL24P3
RPL26P14
RPL26P23
RPL26P4
RPL29P7
RPL31P11
RPL31P23
RPL31P37
RPL31P56
RPL32
RPL32P35
RPL34P26
RPL34P7
RPL35P1
RPL35P4
RPL36AP28
RPL36AP51
RPL38P3
RPL4P3
RPL4P5
RPL6P22
RPL6P27
RPL7AP12
RPL7AP18
RPL7AP58
RPL7L1P7
RPL7P16
RPL7P6
RPLP0P7
RPLP0P8
RPN1
RPRD1A
RPS10-NUDT3
RPS10P12
RPS10P27

RPS10P7
RPS11P3
RPS15AP22
RPS15P2
RPS17P16
RPS20
RPS20P6
RPS24
RPS24P16
RPS25P4
RPS26P8
RPS27P22
RPS4XP8
RPS6KA5
RPS6KA6
RPS6P13
RPS6P16
RPS7P5
RPS8P8
RPSAP11
RPSAP44
RPSAP47
RPSAP52
RPTOR
RRAS2
RREB1
RRN3
RSBN1L
RSL24D1P9
RTN3
RTN4
RTTN
RUFY2
RXFP3
RYBP
RYK
SAMD4A
SAMD9L
SAP30
SASH1
SAV1
SC5DL
SCAF11
SCAI
SCAP

SCARB2
SCEL
SCG5
SCGB1D1
SCIN
SCLY
SCN10A
SCN11A
SCN7A
SCN8A
SCNN1G
SCOC
SCP2
SCRN1
SCTR
SCUBE2
SDC2
SDC3
SDHC
SDR16C5
SEC14L2
SEC16B
SEC22C
SEC24B
SECISBP2L
SEL1L
SEL1L2
SELE
SEMA6A
SEPHS1P3
SEPHS1P5
SEPT15
SEPT2
SERBP1
SERGEF
SERINC5
SERPINE3
SERPINI1
SETBP1
SETDB1
SETP19
SETX
SEZ6L
SFMBT1
SFT2D2

SGPP1
SH2D6
SH3BP4
SH3D19
SH3GL3
SH3RF1
SH3TC2
SHC4
SHOC2
SHQ1
SHROOM3
SI
SIDT1
SIK2
SIK3
SIL1
SIN3A
SIPA1L1
SIPA1L2
SIPA1L3
SIRPB2
SKINTL
SLA2
SLAMF7
SLC10A7
SLC11A2
SLC12A1
SLC15A2
SLC16A7
SLC16A9
SLC17A1
SLC17A2
SLC19A2
SLC1A3
SLC20A1
SLC22A1
SLC22A15
SLC22A16
SLC22A23
SLC22A24
SLC24A4
SLC25A13
SLC25A15P4
SLC25A26
SLC25A33

SLC25A5P4
SLC25A5P8
SLC26A7
SLC27A6
SLC28A3
SLC2AXP1
SLC30A10
SLC30A8
SLC31A1P1
SLC35B4
SLC35D1
SLC36A1
SLC38A4
SLC38A9
SLC39A10
SLC39A12
SLC39A6
SLC39A9
SLC43A1
SLC44A1
SLC44A3
SLC45A3
SLC4A1AP
SLC4A4
SLC5A12
SLC7A14
SLC7A2
SLC8A1
SLC9A1
SLC9A11
SLC9A3R1
SLC9B1
SLCO1A2
SLCO1B7
SLCO1C1
SLCO2A1
SLCO3A1
SLCO6A1
SLFN11
SLITRK3
SLK
SLTM
SMA5
SMAD2
SMARCA2

SMARCC1
SMARCD2
SMC1B
SMC3
SMC6
SMCHD1
SMCP
SMG7
SMOC1
SMPD3
SMURF1
SMURF2
SNAP25
SND1
SNED1
SNORD56B
SNRK
SNTB2
SNX1
SNX16
SNX18P22
SNX25
SOCS6
SORBS1
SORBS2
SORD
SORD2
SOS2
SOX2-OT
SOX5P
SP3
SPAG1
SPAG6
SPATA16
SPATA22
SPATA5
SPATS2
SPECC1L
SPEF2
SPG20OS
SPICE1
SPINK14
SPINK8
SPIRE1
SPOPL

SPRYD5
SPRYD7
SPTLC1
SRBD1
SRD5A2
SREK1
SRI
SRPK1
SRPK2
SRPRB
SRRM1P1
SRSF1
SRSF5
SSX2IP
ST13P8
ST20-MTHFS
ST3GAL1
ST5
ST7L
ST8SIA1
ST8SIA3
STAB2
STAC
STAG2
STAM
STARD13
STARD9
STARP1
STAT4
STEAP2
STEAP4
STIM1
STIM2
STK17A
STK32A
STK33
STK35
STL
STOX1
STRA8
STX11
STX16-NPEPL1
STX17
STX6
STXBP1

SUMF1
SUMO1
SUPT5H
SUSD5
SUV420H1
SVIL
SVOPL
SYCP1
SYDE2
SYK
SYNCRIP
SYNPO2
SYT10
SYT11
SYT2
SYT9
SYTL2
TAAR4P
TAB2
TACC1
TACC2
TACR3
TAF15
TAF1B
TAF2
TAF4B
TAF5L
TANC1
TANK
TAOK3
TAS2R16
TAS2R2
TATDN1
TAX1BP1
TBC1D1
TBC1D12
TBC1D14
TBC1D19
TBC1D22A
TBC1D4
TBC1D8
TBC1D9
TBCA
TBCK
TBRG1

TBXAS1
TC2N
TCEA1
TCEB1P18
TCERG1L
TCF24
TCF7L1
TDP1
TDRD12
TDRD3
TDRD9
TECPR2
TECRL
TECTA
TEK
TET1P1
TEX10
TEX14
TEX2
TFCP2L1
TG
TGFBR3
TGIF1
TGM6
TGS1
THADA
THEM4
TIGD6
TIPARP
TIPRL
TJP1
TKTL2
TLE1
TLK1
TLK2P2
TLL2
TLN2
TM2D1
TM4SF18
TM6SF1
TMC2
TMC3
TMC6
TMCC3
TMEM110

TMEM110-MUSTN1
TMEM132C
TMEM139
TMEM141
TMEM154
TMEM164
TMEM170B
TMEM182
TMEM183B
TMEM184C
TMEM185A
TMEM200A
TMEM209
TMEM30B
TMEM56-RWDD3
TMEM79
TMEM85
TMEM87A
TMEM8B
TMOD2
TMPO
TMPRSS11E
TMTC3
TMX4
TNFAIP3
TNFAIP8
TNFAIP8L3
TNFRSF9
TNFSF11
TNIP3
TNK2
TNKS
TNKS2
TNN
TNRC6A
TOM1L1
TOMM20
TOPBP1
TOR1AIP1
TOX3
TP53BP1
TP53I3
TP53INP1
TP53TG3
TPH2

TPM3
TPRXL
TPST1
TRA2B
TRAPPC9
TRDMT1
TRERF1
TRG@
TRHR
TRIM14
TRIM2
TRIM21
TRIM24
TRIM31
TRIM37
TRIM40
TRIM42
TRIM67
TRIP12
TRIP4
TRMT11
TRMT12
TRNAA32
TRNAA40
TRNAA8
TRNAC18
TRNAK16
TRNAL41P
TRNAL47P
TRNAM16
TRNAN29
TRNAP25P
TRNAQ10
TRNAQ54P
TRNAS1
TRNAT19
TRPC1
TRPC3
TRPC4AP
TRPM1
TRPM7
TRPS1
TRPV6
TRRAP
TSC22D3

TSG1
TSGA10
TSGA13
TSHR
TSPAN12
TSPAN2
TSPAN5
TSPAN9
TTBK2
TTC14
TTC15
TTC17
TTC23
TTC23L
TTC26
TTC37
TTC39B
TTC39C
TTC6
TTC8
TTC9
TTI1
TTL
TTLL5
TUBD1
TUBGCP3
TUBGCP5
TUFT1
TUT1
TWSG1
TXLNB
TXNDC16
TXNRD3NB
TYW1
UACA
UBAP1
UBAP2L
UBE2D1
UBE2E1
UBE2E3
UBE2F-SCLY
UBE2G1
UBE2R2
UBE2W
UBE3A

UBE3C
UBE4A
UBL3
UBN1
UBN2
UBR1
UBR3
UBXN2B
UBXN4
UBXN7
UCHL5
UCK2
UGGT2
UGT1A10
UGT1A4
UGT1A5
UGT1A6
UGT1A7
UGT1A8
UGT1A9
UHRF1BP1
ULK2
UNC13B
UNC5C
UNGP2
UNQ6494
UQCRC2
USP1
USP10
USP13
USP14
USP15
USP22
USP25
USP28
USP3
USP31
USP33
USP37
USP40
USP45
USP50
USP54
USP6
USP6NL

USP8
UST
UTP20
VAPA
VCL
VDAC1
VEGFC
VEZF1
VGLL2
VHLL
VKORC1L1
VN1R3
VN1R37P
VN1R38P
VN1R68P
VN1R69P
VN2R14P
VN2R1P
VPRBP
VPS13C
VPS37C
VPS41
VRK2
VSNL1
VSTM2A
VWC2L
VWF
WAC
WASF1
WASL
WDFY2
WDFY4
WDR12
WDR25
WDR26
WDR27
WDR35
WDR36
WDR41
WDR47
WDR59
WDR60
WDR66
WDR69
WDR7

WDR76
WDR88
WDR91
WDSUB1
WDYHV1
WFDC2
WHSC1
WHSC1L1
WIBG
WLS
WNK1
WNK2
WNT16
WRN
WT1
WWC2
XCL2
XKR3
XKR9
XPNPEP1
XPO1
XPO4
XRN2
YAF2
YAP1
YARS2
YES1
YIPF7
YLPM1
YPEL1
YSK4
YTHDC2
YTHDF3
YWHAQ
YWHAZ
ZAK
ZBTB24
ZBTB41
ZBTB44
ZC3H11B
ZC3H12C
ZC3H6
ZC3HAV1
ZCCHC11
ZCCHC16

ZCCHC2
ZCCHC6
ZDHHC13
ZDHHC2
ZDHHC20
ZDHHC21
ZEB1
ZFAND3
ZFHX3
ZFHX4
ZFYVE16
ZHX2
ZKSCAN1
ZMYND11
ZMYND8
ZNF100
ZNF107
ZNF124
ZNF131
ZNF143
ZNF148
ZNF253
ZNF280D
ZNF281
ZNF383
ZNF398
ZNF430
ZNF438
ZNF444P1
ZNF451
ZNF461
ZNF529
ZNF536
ZNF559-ZNF177
ZNF563
ZNF566
ZNF608
ZNF618
ZNF624
ZNF638
ZNF652
ZNF664-FAM101A
ZNF667
ZNF678
ZNF69

ZNF709
ZNF720
ZNF727
ZNF730
ZNF735
ZNF767
ZNF777
ZNF780B
ZNF85
ZNF861P
ZNF92
ZNRF1
ZNRF2
ZRANB3
ZSCAN12
ZUFSP
ZWILCH
ZYX
