# Supplementary material for: Comprehensive genomics in androgen receptor-dependent castration-resistant prostate cancer identifies an adaptation pathway mediated by opioid receptor kappa 1
Source: Commun Biol. 2022 Apr 1;5:299. doi: 10.1038/s42003-022-03227-w (PMC8976065; doi:10.1038/s42003-022-03227-w)
Supplement: Supplementary file 5 — Supplementary Data S3 [file 42003_2022_3227_MOESM5_ESM.pdf]

Supplementary data S3. Differentially upregulated genes in KUCaP2 CR tumors compared with KUCaP2 AD tumors in RNA seq (fkpm > 3.0 and fold change > 4.0)

#chrom	start	end	Genesymbol	UCSCid	category	mappedE	exonLen	RefSeqId	re	KUCaP2_	KUCaP2_CR266_fpk	CR/AD
chr18	70203914	70211723	CBLN2+	uc002lkv.	coding	0	2894	NM_182511	1	0	6.83175	**68317.5**
chr2	12856997	12882856	TRIB2+	uc002rbv.	coding	596	4404	NM_021643	1	0.02224	19.72602	**886.9613**
chr8	72933486	72987819	TRPA1+	uc003xza.	coding	298	5189	NM_007332	1	0.00944	3.28514	**348.0021**
chr2	121103718	121109383	INHBB+	uc002tmn.	coding	1790	3203	NM_002193	1	0.09184	15.60934	**169.9623**
chrX	38420730	38548171	TSPAN7+	uc004deg.	coding	299	1805	NM_004615	1	0.02722	3.32053	**121.9886**
chr18	48086483	48258195	MAPK4+	uc002lev.	coding	6112	4735	NM_002747	1	0.21213	20.90885	**98.56621**
chr4	62362838	62938167	LPHN3+ (ADGRL3)	uc010ihh.	coding	4398	6124	NM_015236	1	0.11802	11.50693	**97.49983**
chr14	21467418	21470030	SLC39A2+	uc001vyr.	coding	298	1409	NM_014579	1	0.03476	3.07128	**88.35673**
chr1	861120	879961	SAMD11+	uc001abw	coding	599	2554	NM_152486	1	0.03854	3.1564	**81.89933**
chr1	31652592	31661034	NKAIN1+	uc001bsn.	coding	894	2527	NM_024522	1	0.05814	4.58932	**78.93567**
chr16	1127721	1128731	BC084558+ (SSTR5)	uc002ckp.	nearCodin	298	1010		1	0.04849	3.1638	**65.24644**
chr18	48321490	48351754	MRO+	uc010dpc.	coding	2712	4887	NM_001127174	1	0.0912	5.7885	**63.47039**
chr7	126078651	126892428	GRM8+	uc003vlt.2	coding	4537	3860	NM_001127323	1	0.19316	12.23531	**63.34288**
chr3	112323410	112359977	CCDC80+	uc003dzg.	coding	1494	4521	NM_199511	1	0.05431	3.30644	**60.88087**
chr18	13620866	13652753	C18orf1+ (LDLRAD4)	uc002ksi.	coding	4970	8032	NM_181483	1	0.10169	5.75573	**56.60075**
chr9	104331634	104500862	GRIN3A+	uc004bbp.	coding	14910	7770	NM_133445	1	0.31535	13.73782	**43.56372**
chr3	148890291	148939832	CP+	uc003ewy	coding	13315	4660	NM_000096	1	0.46956	19.67057	**41.89149**
chr9	94484883	94712444	ROR2+	uc004arj.1	coding	9879	4091	NM_004560	1	0.39685	14.66428	**36.95169**
chr4	176554088	176923648	GPM6A+	uc003iuh.	coding	7538	2956	NM_201592	1	0.41907	15.06068	**35.93834**
chr3	139654026	140286917	CLSTN2+	uc003etn.	coding	27200	4880	NM_022131	1	0.91598	31.02376	**33.86947**
chr13	26618734	26625198	SHISA2+	uc001uqm	coding	1787	2889	NM_001007538	1	0.10165	3.30822	**32.5452**
chr6	84743419	84800603	MRAP2+	uc003pkg.	coding	1944	2211	NM_138409	1	0.14449	4.67017	**32.32175**
chr6	130758261	130764208	TMEM200A+	uc003qcb.	coding	9604	5019	NM_052913	1	0.31447	9.43805	**30.01256**
chr6	43044028	43129457	DKFZp434L0319+ (PTK7)	uc003oug.	nearCodin	20844	4053		1	0.84517	24.85299	**29.40591**
chr11	73359734	73373862	PLEKHB1+	uc009ytq.	coding	1471	2146	NM_001130036	1	0.11265	3.2778	**29.0972**
chr4	100227527	100242572	ADH1B+	uc003hus.	coding	8382	2681	NM_000668	1	0.51379	12.86617	**25.04169**
chr3	127407910	127542051	MGLL+	uc003ejx.	coding	6567	4187	NM_001003794	1	0.25775	6.15741	**23.88908**
chr8	54138275	54164194	OPRK1+	uc003xri.1	coding	24176	4959	NM_000912	1	0.80118	18.61689	**23.23684**
chr1	183217378	183248417	KIAA0479+ (NMNAT2)	uc009wye	nearCodin	9097	4913		1	0.30429	6.18975	**20.34161**
chr1	210502263	210849631	HHAT+	uc001hhz.	coding	3995	3620	NM_018194	1	0.18136	3.67298	**20.25243**
chr4	71062243	71070292	ODAM+	uc003hfc.	coding	3449	1287	NM_017855	1	0.44041	8.35548	**18.97205**
chr12	15034826	15038828	MGP+	uc001rcn.	coding	897	661	NM_000900	1	0.22301	4.01925	**18.02273**
chr8	61101422	61193954	CA8+	uc003xtz.	coding	2987	2278	NM_004056	1	0.21549	3.83368	**17.79052**
chr3	112051915	112081656	MOX2-	uc003dyy.	coding	2531	2126		1	0.19564	3.28707	**16.80163**
chr17	62116509	62119203	BC070350+	uc002jdy.	noncoding	61230	2694		1	3.73513	62.16379	**16.64301**
chr11	119179234	119187840	MCAM+	uc001pwf.	coding	24736	3331	NM_006500	1	1.22038	20.26021	**16.60156**
chr3	18389265	18480252	SATB1+	uc003cbj.	coding	17950	3908	NM_001131010	1	0.75483	10.96192	**14.52237**
chr3	4535033	4889286	ITPR1+	uc010hca.	coding	25522	9813	NM_002222	1	0.42742	6.03559	**14.12098**
chr19	6494329	6502330	TUBB4+	uc002mfg.	coding	4920	2287	NM_006087	1	0.35354	4.97948	**14.08463**
chr11	118004093	118023630	SCN4B+	uc010rxv.	coding	9635	4175	NM_001142348	1	0.37926	5.23553	**13.80459**
chr2	69240275	69476457	ANTXR1+	uc002sfg.	coding	19184	5891	NM_032208	1	0.53517	7.29952	**13.63963**
chr1	20301924	20306932	PLA2G2A+	uc010odb.	coding	159561	888	NM_001161728	1	29.52926	401.4359	**13.59451**
chr17	62120389	62207502	ERN1+	uc002jdz.	coding	84655	4005	NM_001433	1	3.47367	44.42845	**12.79006**
chr1	223394162	223537544	UNQ196+ (SUSD4)	uc010puw	coding	15919	3180		1	0.82267	10.50224	**12.76604**
chr8	98881310	99048944	MATN2+	uc003yid.	coding	14826	4049	NM_030583	1	0.60175	7.46781	**12.41015**
chr3	15296356	15382901	SH3BP5+	uc003bzr.	coding	5225	2706	NM_001018009	1	0.31732	3.9018	**12.2961**
chrX	64887510	64961792	MSN+	uc004dwf.	coding	11691	3969	NM_002444	1	0.48407	5.74861	**11.87558**
chr19	55795533	55823901	BRSK1+	uc002qkg.	coding	8392	3079	NM_032430	1	0.44791	5.29529	**11.82222**
chr20	2673523	2740753	EBF4+	uc002wgt.	coding	6589	2910	NM_001110514	1	0.3721	4.35743	**11.71037**
chr2	234826042	234928165	TRPM8+	uc002vvh.	coding	13163	5620	NM_024080	1	0.38491	4.46623	**11.60331**
chr19	18723681	18731843	TMEM59L+	uc002njy.	coding	8945	1613	NM_012109	1	0.91135	10.32176	**11.32579**
chr2	234890417	234928165	LTRPC6+	uc010fyk.	nearCodin	6667	3260		1	0.33609	3.64618	**10.84882**
chr3	46710678	46735171	ALS2CL+	uc003cqb.	coding	23935	4721	NM_147129	1	0.83318	8.93203	**10.72041**
chr7	45002259	45018704	MYO1G+	uc003tmh.	coding	6146	3279	NM_033054	1	0.30803	3.15491	**10.24222**
chr16	1128780	1130142	SSTR5+	uc002ckq.	coding	4174	1362	NM_001053	1	0.50363	5.03252	**9.992494**
chr1	156669409	156675375	CRABP2+	uc001fpr.	coding	2536	962	NM_001878	1	0.43322	4.2991	**9.923595**
chr2	16733900	16847096	FAM49A+	uc002rck.	coding	13329	1490	NM_030797	1	1.47011	14.22051	**9.673092**
chr8	120428551	120436617	NOV+	uc003yoq.	coding	72089	2601	NM_002514	1	4.55478	43.83421	**9.623782**
chr7	89874487	89940375	C7orf63+	uc010lep.	coding	53272	3900	NM_001039706	1	2.24478	21.33437	**9.503991**
chr2	86066270	86116157	ST3GAL5+	uc002sqq.	coding	8618	2382	NM_003896	1	0.59457	5.60529	**9.427469**
chr7	63505820	63538925	ZNF727+	uc011kdm	coding	4499	1677	NM_001159522	1	0.44088	4.0441	**9.172791**
chr12	65444406	65515116	WIF1+	uc001ssk.	coding	5532	2007	NM_007191	1	0.45297	4.13141	**9.120714**
chr22	36585177	36600879	APOL4+	uc003aox.	coding	15634	3223	NM_145660	1	0.79717	7.26749	**9.116613**
chr2	152689287	152955593	CACNB4+	uc010fnz.	coding	16562	7790	NM_001145798	1	0.34939	3.17524	**9.087953**
chr5	39371779	39425335	DAB2+	uc003jlx.2	coding	11740	4597	NM_001343	1	0.41969	3.77175	**8.98699**
chr12	106457124	106533811	NUAK1+	uc001tlj.1	coding	32200	6821	NM_014840	1	0.77579	6.66536	**8.591707**
chr3	100211462	100296285	TMEM45A+	uc003dtz.	coding	14610	1564	NM_018004	1	1.53516	13.11196	**8.541103**
chr3	40428672	40470109	ENTPD3+	uc003ckd.	coding	6729	2792	NM_001248	1	0.39607	3.26507	**8.243669**
chr8	75896842	75946791	CRISPLD1+	uc003yan.	coding	262229	4192	NM_031461	1	10.28012	83.89266	**8.160669**
chr3	158384203	158390482	LXN+	uc003fch.	coding	3437	1115	NM_020169	1	0.50657	4.08567	**8.065361**
chr14	52781015	52795320	PTGER2+	uc001wzr.	coding	7899	2476	NM_000956	1	0.52428	4.11489	**7.84865**
chr1	10057254	10076077	RBP7+	uc001aqq.	coding	6594	654	NM_052960	1	1.65695	12.87504	**7.770325**
chr6	157802556	158094976	ZDHHC14+	uc003qqt.	coding	8613	2786	NM_024630	1	0.50806	3.82076	**7.520293**
chr11	17515442	17565963	USH1C+	uc001mnf.	coding	8374	2236	NM_005709	1	0.61546	4.62615	**7.516573**
chr12	6456010	6486523	SCNN1A+	uc010sfb.	coding	10022	3198	NM_001159575	1	0.51501	3.83555	**7.447525**
chr12	26111968	26232824	RASSF8+	uc001rgz.	coding	9700	2312	NM_007211	1	0.68948	5.11507	**7.418736**
chr7	29959719	30029905	SCRN1+	uc011kaa.	coding	28125	5207	NM_001145514	1	0.88765	6.56584	**7.396879**
chr1	175126122	175162229	KIAA0040+	uc001gko.	coding	31068	4644	NM_001162894	1	1.09941	7.99436	**7.2715**
chr5	9035137	9546233	SEMA5A+	uc003jek.	coding	244911	11808	NM_003966	1	3.40856	24.5159	**7.192451**
chr5	32788944	32791819	C5orf23+	uc003jhw.	coding	42760	2875	NM_024563	1	2.44421	17.47742	**7.150539**
chr2	48796158	48906746	STON1-GTF2A1L+	uc002rwp.	coding	11914	3828	NM_172311	1	0.51148	3.64933	**7.134844**
chr14	23594505	23652849	SLC7A8+	uc001wiz.	coding	50506	4214	NM_012244	1	1.96964	13.95477	**7.084934**

chr6	134490384	134639196	SGK1+	uc003qeo.	coding	13166	3200	NM_001143676	1	0.67615	4.5365	**6.70931**
chr4	57514153	57547872	HOPX+	uc003hcb.	coding	28877	1418	NM_139212	1	3.34668	22.25158	**6.648852**
chr13	25946208	26595419	ATP8A2+	uc001uqk.	coding	24400	5005	NM_016529	1	0.80117	5.31529	**6.63441**
chr2	234526290	234681949	UGT1A8+	uc002vup.	coding	151195	2400	NM_019076	1	10.35297	68.57543	**6.623745**
chr16	21269842	21314417	CRYM+	uc002dil.2	coding	14872	1241	NM_001014444	1	1.96941	13.03927	**6.620902**
chr4	154387497	154557860	KIAA0922+	uc003inm.	coding	42804	5014	NM_015196	1	1.40294	9.2201	**6.571985**
chr13	93879077	95060267	GPC6+	uc001vlt.2	coding	20884	7096	NM_005708	1	0.48366	3.17841	**6.571579**
chr2	234637772	234681949	UGT1A3+	uc002vuy.	coding	208640	2349	NM_019093	1	14.59665	95.59833	**6.549334**
chr2	234601511	234681949	UGT1A6+	uc002vuv.	coding	150471	2482	NM_001072	1	9.96299	65.19094	**6.543311**
chr2	234545122	234681949	UGT1A10+	uc002vur.	coding	149886	2383	NM_019075	1	10.33655	67.47499	**6.527806**
chr2	234621637	234681949	UGT1A5+	uc002vuw	coding	149906	2349	NM_019078	1	10.48757	68.32954	**6.515288**
chr9	4490443	4587469	SLC1A1+	uc003zij.1	coding	48268	3722	NM_004170	1	2.13119	13.87575	**6.510799**
chr2	234580543	234681949	UGT1A9+	uc002vus.	coding	149871	2374	NM_021027	1	10.3747	67.49372	**6.505607**
chr2	234590583	234681949	UGT1A7+	uc002vut.	coding	149865	2337	NM_019077	1	10.53854	68.52166	**6.502007**
chr2	234627437	234681949	UGT1A4+	uc002vux.	coding	149876	2378	NM_007120	1	10.3576	67.32732	**6.500282**
chr3	3841120	3889386	LRRN1+	uc003bpt.	coding	26821	3822	NM_020873	1	1.15325	7.49183	**6.496276**
chr20	39314518	39317876	MAFB+	uc002xji.2	coding	48415	3358	NM_005461	1	2.3694	15.25779	**6.439516**
chr21	43640007	43717352	ABCG1+	uc002zar.	coding	16245	2958	NM_207174	1	0.90253	5.80049	**6.426922**
chrX	46937774	46952712	RGN+	uc004dha.	coding	4782	1615	NM_004683	1	0.4866	3.07693	**6.323325**
chr20	36766352	36793700	TGM2+	uc002xht.	coding	14468	1877	NM_198951	1	1.26673	7.91245	**6.246359**
chr22	25202135	25322813	SGSM1+	uc003abj.	coding	19432	5828	NM_001098498	1	0.54794	3.42238	**6.245903**
chrX	17653412	17754111	NHS+	uc004cxy.	coding	27775	8226	NM_001136024	1	0.55489	3.45681	**6.229721**
chr2	242127923	242164791	ANO7+	uc002wax	coding	21190	4128	NM_001001891	1	0.84359	5.23286	**6.203084**
chr20	36531498	36573745	VSTM2L+	uc002xhk.	coding	36699	1959	NM_080607	1	3.07864	18.76346	**6.094724**
chr4	154505977	154510159	DKFZp586H1322+	uc010ips.1	coding	5037	615		1	1.34597	8.1847	**6.080893**
chr21	28208607	28217728	ADAMTS1+	uc002ymf.	coding	117937	4649	NM_006988	1	4.16898	25.22282	**6.050118**
chr2	234668918	234681949	UGT1A1+	uc002vvb.	coding	193006	2361	NM_000463	1	13.43426	80.73155	**6.009378**
chr12	41086357	41464094	CNTN1+	uc001rmn	coding	77588	3394	NM_175038	1	3.75683	22.49303	**5.987237**
chr6	3722835	3752246	C6orf145+	uc003mvt.	coding	23240	2194	NM_183373	1	1.74076	10.36897	**5.956576**
chr19	17003762	17137625	CPAMD8+	uc002nfb.	coding	46739	5987	NM_015692	1	1.28295	7.60885	**5.930746**
chr2	111490149	111875799	ACOXL+	uc010yxk.	coding	8930	2373	NM_001142807	1	0.61843	3.6246	**5.860971**
chr5	32711664	32787252	NPR3+	uc003jhu.	coding	28450	2642	NM_000908	1	1.76965	10.16221	**5.742497**
chr4	6322305	6474326	PPP2R2C+	uc003gjc.	coding	28883	4437	NM_020416	1	1.06977	6.12947	**5.729708**
chr11	115044345	115375241	CADM1+	uc001ppk.	coding	136345	4222	NM_001098517	1	5.30713	29.97538	**5.648134**
chr9	116918230	117072974	COL27A1+	uc011lxl.1	coding	57559	5582	NM_032888	1	1.69458	9.48635	**5.598054**
chr16	15596122	15682115	C16orf45+	uc002ddp.	coding	52019	2049	NM_001142469	1	4.17214	23.28868	**5.581951**
chr22	45680888	45691754	UPK3A+	uc003bfy.	coding	16862	1024	NM_006953	1	2.70613	14.51702	**5.364495**
chr11	30406039	30607930	MPPED2+	uc001msq	coding	40484	5693	NM_001145399	1	1.16864	6.00738	**5.140488**
chr8	1922043	1955108	KBTBD11+	uc003wpw	coding	24590	6716	NM_014867	1	0.60171	3.07138	**5.104419**
chrX	17818169	17879457	RAI2+	uc010nfa.	coding	20682	2225	NM_021785	1	1.52757	7.71677	**5.051664**
chr11	75897370	75917574	WNT11+	uc001oxe.	coding	268048	1926	NM_004626	1	22.87152	114.8591	**5.021926**
chr7	102220093	102257205	RASA4+	uc003vaf.	coding	66561	5467	NM_001079877	1	2.00083	9.90173	**4.948811**
chr18	616699	650291	CLUL1+	uc002kkq.	coding	14011	1862	NM_199167	1	1.2366	6.11692	**4.946563**
chr10	5005453	5020157	AKR1C1+	uc001ihq.	coding	479319	1378	NM_001353	1	57.16289	277.6811	**4.857717**
chrX	72432154	72434684	NAP1L2+	uc004ebi.	coding	23693	2530	NM_021963	1	1.539	7.44744	**4.839142**
chr6	45389913	45518818	RUNX2+	uc003oxt.	coding	34863	5700	NM_004348	1	1.00514	4.81013	**4.785532**
chr2	157180945	157189287	NR4A2+	uc002tyz.	coding	48090	3529	NM_006186	1	2.23945	10.49493	**4.686387**
chr9	38621084	38623275	C9orf122+	uc004abh.	coding	12232	1725	NR_027294	1	1.16533	5.37652	**4.613732**
chr10	101802064	101841642	CPN1+	uc001kql.	coding	8882	1756	NM_001308	1	0.83124	3.83142	**4.609283**
chr11	121322960	121504471	SORL1+	uc001pxx.	coding	142512	10924	NM_003105	1	2.14392	9.83631	**4.588002**
chr21	46875423	46933633	COL18A1+	uc011afs.	coding	50496	6607	NM_130444	1	1.25601	5.74303	**4.57244**
chr20	62119365	62130505	EEF1A2+	uc002yfe.	coding	209331	1843	NM_001958	1	18.66582	83.15981	**4.455192**
chr2	219919145	219925189	IHH+	uc002vjo.1	coding	57582	2019	NM_002181	1	4.68694	20.73706	**4.424435**
chr5	659977	693510	TPPP+	uc003jbh.	coding	150854	6021	NM_007030	1	4.11744	18.04308	**4.382111**
chr12	15260717	15374304	RERG+	uc001rct.2	coding	10887	2216	NM_032918	1	0.80738	3.51321	**4.351371**
chr22	42372930	42394223	SEPT3+	uc003bbs.	coding	46815	4654	NM_019106	1	1.65309	7.14791	**4.323969**
chr6	168707585	168720402	DACT2+	uc003qwq	coding	39062	2940	NM_214462	1	2.18346	9.35822	**4.285959**
chr20	62065031	62103993	KCNQ2+	uc002yfc.	coding	33005	1425	NM_172109	1	3.80631	16.19854	**4.255707**
chr17	73720773	73753898	ITGB4+	uc002jpj.2	coding	34810	5690	NM_001005619	1	1.00538	4.27631	**4.253427**
chr17	48712217	48769062	ABCC3+	uc002isl.2	coding	116573	5165	NM_003786	1	3.70908	15.77073	**4.251925**
chr8	142138719	142205898	DENND3+	uc003yvy.	coding	38604	5441	NM_014957	1	1.16598	4.93607	**4.233409**
chr13	72012097	72441330	DACH1+	uc010thp.	coding	90904	4627	NM_004392	1	3.22866	13.62814	**4.22099**
chr1	40766162	40782981	COL9A2+	uc001cfh.	coding	63769	2873	NM_001852	1	3.64765	15.35003	**4.208197**
chr7	192968	300711	FAM20C+	uc003sip.	coding	60030	2751	NM_020223	1	3.58605	14.61564	**4.075693**
chr21	15215454	15220685	C21orf15+	uc002yjf.2	noncoding	34955	1278	NR_026755	1	4.49487	18.1202	**4.031307**
chr18	14477955	14498705	CXADRP3+	uc010xai.1	noncoding	116705	1608	NR_024076	1	11.9273	47.90839	**4.0167**
chr21	17442841	17982094	C21orf34+	uc002ykb.	noncoding	17557	3475	NR_027790	1	0.8303	3.33188	**4.012863**
