# Supplementary material for: Comprehensive genomics in androgen receptor-dependent castration-resistant prostate cancer identifies an adaptation pathway mediated by opioid receptor kappa 1
Source: Commun Biol. 2022 Apr 1;5:299. doi: 10.1038/s42003-022-03227-w (PMC8976065; doi:10.1038/s42003-022-03227-w)
Supplement: Supplementary file 6 — Supplementary Data S4 [file 42003_2022_3227_MOESM6_ESM.pdf]

**Supplementary data S4. Differentially downregulated genes in KUCaP2 CR tumors compared with KUCaP2 AD tumors in RNA seq (fkpm > 3.0 and fold change < 0.25)**

| #chrom | start | end | Genesymbol | UCSCid | category | mappedB | exonLeng | RefSeqId | re | KUCaP2_/ | KUCaP2_CR266_fpk | CR/AD |
|---|---|---|---|---|---|---|---|---|---|---|---|---|
| chr1 | 235092978 | 235095736 | XLOC_000627+ | XLOC_00( | noncoding | 38904 | 421 | XLOC_000627 | 1 | 15.18626 | 0.00527 | 0.000347 |
| chr17 | 74620846 | 74639894 | ST6GALNAC1+ | uc002jsh.2 | coding | 762990 | 2542 | NM_018414 | 1 | 49.3267 | 0.41774 | 0.008469 |
| chr4 | 70066052 | 70080449 | UGT2B11+ | uc003heh. | coding | 138243 | 1704 | NM_001073 | 1 | 13.33252 | 0.15599 | 0.0117 |
| chr2 | 162848756 | 162931052 | DPP4+ | uc002ubz. | coding | 1897041 | 3898 | NM_001935 | 1 | 79.97857 | 1.60458 | 0.020063 |
| chr13 | 77454303 | 77460540 | KCTD12+ | uc010aeu. | coding | 254328 | 6237 | NM_138444 | 1 | 6.70127 | 0.14137 | 0.021096 |
| chrX | 151903227 | 151909516 | CSAG1+ | uc004fgf.2 | coding | 60297 | 657 | NM_001102576 | 1 | 15.08235 | 0.33658 | 0.022316 |
| chrX | 43515408 | 43606067 | MAOA+ | uc004dfy.2 | coding | 699614 | 4072 | NM_000240 | 1 | 28.23511 | 0.63151 | 0.022366 |
| chr4 | 42112871 | 42154895 | BEND4+ | uc003gwm | coding | 164503 | 8777 | NM_001159547 | 1 | 3.08011 | 0.07039 | 0.022853 |
| chr6 | 142623055 | 142767401 | GPR126+ (ADGRG6 | uc010khf.2 | coding | 211442 | 6829 | NM_001032395 | 1 | 5.0883 | 0.12816 | 0.025187 |
| chr7 | 38369947 | 38370558 | X06774+ | uc010kxj.1 | coding | 10315 | 486 | | 1 | 3.48796 | 0.0907 | 0.026004 |
| chr3 | 102153858 | 102198685 | ZPLD1+ | uc003dvt.1 | coding | 1313205 | 3619 | NM_175056 | 1 | 59.63246 | 1.56732 | 0.026283 |
| chrX | 151899298 | 151903172 | MAGEA12+ | uc004fgc.2 | coding | 104521 | 1646 | NM_005367 | 1 | 10.43548 | 0.30841 | 0.029554 |
| chr13 | 28536278 | 28543317 | CDX2+ | uc001urv.2 | coding | 92463 | 2089 | NM_001265 | 1 | 7.27392 | 0.22177 | 0.030488 |
| chr4 | 70345883 | 70361626 | UGT2B4+ | uc003hek. | coding | 417239 | 2102 | NM_021139 | 1 | 32.62053 | 1.02035 | 0.031279 |
| chr18 | 40847858 | 40857615 | SYT4+ | uc002law.2 | coding | 1499848 | 4094 | NM_020783 | 1 | 60.20578 | 2.25213 | 0.037407 |
| chr2 | 8996700 | 9143876 | MBOAT2+ | uc002qzg. | coding | 702634 | 3804 | NM_138799 | 1 | 30.3548 | 1.16167 | 0.03827 |
| chr1 | 55505219 | 55530523 | PCSK9+ | uc001cyf.1 | coding | 243812 | 3636 | NM_174936 | 1 | 11.01971 | 0.45571 | 0.041354 |
| chr3 | 58490863 | 58522929 | ACOX2+ | uc003dkl.2 | coding | 105593 | 2308 | NM_003500 | 1 | 7.51862 | 0.31692 | 0.042151 |
| chr9 | 93372113 | 93405108 | DIRAS2+ | uc004aqx. | coding | 98835 | 4107 | NM_017594 | 1 | 3.9548 | 0.19852 | 0.050197 |
| chr10 | 88728187 | 88730666 | C10orf116+ (ADIRF) | uc001ked. | coding | 72305 | 672 | NM_006829 | 1 | 17.68226 | 1.02177 | 0.057785 |
| chr19 | 15751706 | 15771569 | CYP4F3+ | uc002nbj.2 | coding | 90027 | 2987 | NM_000896 | 1 | 4.95309 | 0.32238 | 0.065087 |
| chr11 | 9041934 | 9113155 | SCUBE2+ | uc001mhh | coding | 595684 | 3737 | NM_020974 | 1 | 26.1958 | 1.80694 | 0.068978 |
| chr7 | 38299243 | 38313253 | TARP+ | uc003tgc.1 | coding | 136913 | 1032 | NM_001003806 | 1 | 21.80237 | 1.61346 | 0.074004 |
| chr3 | 78646387 | 79817059 | ROBO1+ | uc003dqe. | coding | 212761 | 6839 | NM_002941 | 1 | 5.11256 | 0.39204 | 0.076682 |
| chr5 | 110834021 | 110848157 | STARD4+ | uc003kph. | coding | 115168 | 2264 | NM_139164 | 1 | 8.35977 | 0.64249 | 0.076855 |
| chr4 | 72607412 | 72649888 | GC+ | uc003hge. | coding | 1135532 | 1778 | NM_000583 | 1 | 104.9558 | 9.47055 | 0.090234 |
| chr1 | 64239689 | 64644707 | ROR1+ | uc001dbj.2 | coding | 70459 | 3382 | NM_005012 | 1 | 3.42375 | 0.33283 | 0.097212 |
| chr4 | 100257650 | 100273917 | ADH1C+ | uc003huu. | coding | 85674 | 1468 | NM_000669 | 1 | 9.59095 | 0.94433 | 0.098461 |
| chr1 | 169075946 | 169101960 | ATP1B1+ | uc001gfr.1 | coding | 350724 | 2200 | NM_001001787 | 1 | 26.19881 | 2.6432 | 0.10089 |
| chr20 | 7863630 | 7921093 | HAO1+ | uc002wmv | coding | 158977 | 1746 | NM_017545 | 1 | 14.96335 | 1.59709 | 0.106733 |
| chr16 | 21245015 | 21263750 | ANKS4B+ | uc010bwp | coding | 192178 | 2906 | NM_145865 | 1 | 10.86793 | 1.24482 | 0.114541 |
| chr1 | 201252579 | 201302115 | PKP1+ | uc001gwe | coding | 1493903 | 5378 | NM_001005337 | 1 | 45.64996 | 5.36627 | 0.117553 |
| chrX | 16141423 | 16171640 | GRPR+ | uc004cxj.2 | coding | 110674 | 2680 | NM_005314 | 1 | 6.78656 | 0.81536 | 0.120143 |
| chr7 | 12629147 | 12693225 | SCIN+ | uc003sso. | coding | 382587 | 2568 | NM_033128 | 1 | 24.48353 | 3.12859 | 0.127783 |
| chr5 | 147204142 | 147211260 | SPINK1+ | uc003los.2 | coding | 163730 | 441 | NM_003122 | 1 | 61.01385 | 8.02806 | 0.131578 |
| chr1 | 57320442 | 57383894 | C8A+ | uc001cyo. | coding | 61689 | 2392 | NM_000562 | 1 | 4.23824 | 0.56514 | 0.133343 |
| chr4 | 87515467 | 87736326 | PTPN13+ | uc003hqb. | coding | 224372 | 7987 | NM_080684 | 1 | 4.61662 | 0.64258 | 0.139188 |
| chr11 | 33037727 | 33055126 | DEPDC7+ | uc001muc | coding | 140235 | 1992 | NM_139160 | 1 | 11.56927 | 1.70965 | 0.147775 |
| chr9 | 90112755 | 90323548 | DAPK1+ | uc004apd. | coding | 176721 | 5937 | NM_004938 | 1 | 4.8917 | 0.73226 | 0.149694 |
| chr11 | 60467046 | 60483282 | MS4A8B+ | uc001npv. | coding | 245372 | 1351 | NM_031457 | 1 | 29.84753 | 4.47745 | 0.150011 |
| chr1 | 121484057 | 121485429 | XLOC_000992- | XLOC_00( | noncoding | 144694 | 1213 | XLOC_000992 | 1 | 19.60327 | 3.10697 | 0.158492 |
| chr20 | 43029923 | 43060029 | HNF4A+ | uc002xma | coding | 95704 | 3238 | NM_000457 | 1 | 4.85727 | 0.80025 | 0.164753 |
| chr5 | 113769226 | 113832196 | KCNN2+ | uc003kqp. | coding | 37210 | 1456 | NM_170775 | 1 | 4.19988 | 0.72251 | 0.172031 |
| chr4 | 69962192 | 69978704 | UGT2B7+ | uc003heg. | coding | 57531 | 1886 | NM_001074 | 1 | 5.01302 | 0.86758 | 0.173065 |
| chr1 | 55315299 | 55352921 | DHCR24+ | uc001cyc. | coding | 4474359 | 4286 | NM_014762 | 1 | 171.5606 | 29.7549 | 0.173437 |
| chr15 | 55495801 | 55582001 | RAB27A+ | uc002acr.2 | coding | 653958 | 2799 | NM_183235 | 1 | 38.39598 | 6.71309 | 0.174838 |
| chr7 | 50526135 | 50633154 | DDC+ | uc003tpg.3 | coding | 311612 | 2068 | NM_001082971 | 1 | 24.76295 | 4.49499 | 0.181521 |
| chr11 | 94823016 | 94865815 | ENDOD1+ | uc001pfh.2 | coding | 282940 | 4650 | NM_015036 | 1 | 9.99954 | 1.83893 | 0.183901 |
| chr17 | 30814104 | 30818270 | CDK5R1+ | uc002hhn. | coding | 477543 | 3853 | NM_003885 | 1 | 20.36818 | 3.75434 | 0.184324 |
| chr16 | 52471917 | 52581714 | TOX3+ | uc010vgt.1 | coding | 123740 | 3123 | NM_001146188 | 1 | 6.51144 | 1.2135 | 0.186364 |
| chr8 | 113235160 | 114449242 | CSMD3+ | uc011lhx.1 | coding | 262707 | 12615 | NM_052900 | 1 | 3.42234 | 0.63799 | 0.186419 |
| chr22 | 37966253 | 37976024 | LGALS2+ | uc003ata.2 | coding | 35746 | 527 | NM_006498 | 1 | 11.14694 | 2.09637 | 0.188067 |
| chr9 | 79115548 | 79122330 | GCNT1+ | uc004akh. | coding | 463485 | 5605 | NM_001097636 | 1 | 13.58935 | 2.67014 | 0.196488 |
| chr2 | 224461659 | 224467121 | SCG2+ | uc002vnm | coding | 51714 | 2474 | NM_003469 | 1 | 3.43516 | 0.68948 | 0.200713 |
| chr7 | 99564351 | 99573687 | AZGP1+ | uc003ush. | coding | 225803 | 1215 | NM_001185 | 1 | 30.54163 | 6.18509 | 0.202513 |
| chr4 | 72204769 | 72437803 | SLC4A4+ | uc003hgc. | coding | 412447 | 7639 | NM_003759 | 1 | 8.873 | 1.82561 | 0.205749 |
| chr6 | 10980994 | 11044624 | ELOVL2+ | uc003mzp | coding | 244917 | 4071 | NM_017770 | 1 | 9.88682 | 2.04372 | 0.206712 |
| chr3 | 167727653 | 167813417 | GOLIM4+ | uc003ffe.2 | coding | 2772638 | 2838 | NM_014498 | 1 | 160.5534 | 33.28662 | 0.207324 |
| chr7 | 157331750 | 158380482 | PTPRN2+ | uc003wnq | coding | 354526 | 4739 | NM_130843 | 1 | 12.2942 | 2.551 | 0.207496 |
| chr18 | 70501276 | 70532934 | NETO1+ | uc002lkz.2 | coding | 133663 | 1846 | NM_138999 | 1 | 11.89921 | 2.49863 | 0.209983 |
| chr19 | 18284578 | 18288927 | IFI30+ | uc002nic.1 | coding | 24040 | 1032 | NM_006332 | 1 | 3.82819 | 0.81483 | 0.21285 |
| chr12 | 12224401 | 12252625 | BCL2L14+ | uc001rae.2 | coding | 41741 | 2035 | NM_030766 | 1 | 3.37083 | 0.72897 | 0.216258 |
| chr7 | 107788083 | 108096826 | NRCAM+ | uc003vfc.2 | coding | 2188917 | 6294 | NM_005010 | 1 | 57.1533 | 12.39016 | 0.216788 |
| chr9 | 101569980 | 101612358 | GALNT12+ | uc004ayz. | coding | 78897 | 2730 | NM_024642 | 1 | 4.74938 | 1.03753 | 0.218456 |
| chr13 | 107142097 | 107187337 | EFNB2+ | uc001vqi.2 | coding | 414891 | 4317 | NM_004093 | 1 | 15.79395 | 3.47571 | 0.220066 |
| chr5 | 13690436 | 13944589 | DNAH5+ | uc003jfd.2 | coding | 510481 | 15573 | NM_001369 | 1 | 5.38699 | 1.18605 | 0.220169 |
| chr21 | 47609037 | 47648738 | LSS+ | uc002zik.2 | coding | 1654085 | 4390 | NM_001145437 | 1 | 61.92016 | 13.77258 | 0.222425 |
| chr1 | 153963238 | 153964632 | RPS27- | uc001fdv.2 | coding | 33347 | 352 | NM_001030 | 1 | 15.56872 | 3.51133 | 0.225537 |
| chr10 | 126085871 | 126107519 | OAT+ | uc001lhp.2 | coding | 542559 | 2066 | NM_000274 | 1 | 43.15742 | 9.90523 | 0.229514 |
| chr6 | 119499266 | 119670926 | MAN1A1+ | uc003pym | coding | 401895 | 4121 | NM_005907 | 1 | 16.02687 | 3.68157 | 0.229712 |
| chr6 | 86159301 | 86205496 | NT5E+ | uc003pko. | coding | 317127 | 4055 | NM_002526 | 1 | 12.85231 | 2.99301 | 0.232877 |
| chr14 | 55595871 | 55612147 | LGALS3+ | uc001xbr.2 | coding | 802001 | 1062 | NM_002306 | 1 | 124.105 | 29.2888 | 0.236 |
| chr6 | 46761093 | 46807519 | MEP1A+ | uc010jzh.1 | coding | 1057243 | 2929 | NM_005588 | 1 | 59.31905 | 14.06724 | 0.237145 |
| chr3 | 169557028 | 169587660 | LRRC31+ | uc003fgc.1 | coding | 122314 | 2465 | NM_024727 | 1 | 8.15451 | 1.96474 | 0.240939 |
| chr8 | 53023391 | 53322439 | ST18+ | uc003xra.2 | coding | 131311 | 6330 | NM_014682 | 1 | 3.40907 | 0.82219 | 0.241177 |
| chr6 | 25652428 | 25702006 | SCGN+ | uc003nfb.2 | coding | 94248 | 1477 | NM_006998 | 1 | 10.4865 | 2.56635 | 0.244729 |
| chr17 | 64961012 | 65029518 | CACNG4+ | uc002jft.1 | coding | 217902 | 3397 | NM_014405 | 1 | 10.54155 | 2.58076 | 0.244818 |
| chr12 | 81191170 | 81331694 | LIN7A+ | uc001szj.1 | coding | 69721 | 1230 | NM_004664 | 1 | 9.31531 | 2.31278 | 0.248277 |
| chr4 | 103182820 | 103266655 | SLC39A8+ | uc003hwc | coding | 73048 | 3170 | NM_001135146 | 1 | 3.78693 | 0.94116 | 0.248528 |
