# Supplementary material for: Comprehensive genomics in androgen receptor-dependent castration-resistant prostate cancer identifies an adaptation pathway mediated by opioid receptor kappa 1
Source: Commun Biol. 2022 Apr 1;5:299. doi: 10.1038/s42003-022-03227-w (PMC8976065; doi:10.1038/s42003-022-03227-w)
Supplement: Supplementary file 9 — Supplementary Data S7 [file 42003_2022_3227_MOESM9_ESM.pdf]

Supplementary data S7. List of antibodies used in the prenset study

Antibody	Company	Reference#	Application	Dilution in Western blotti
AR	Santa Cruz	N-20,sc-816	WB	1:400
AR	Santa Cruz	C-19,sc-815	WB	1:400
PSA	Cell Signaling	D11E1,#2475	WB, IHC	1:1000
β-actin	Abcam	ab6276	WB	1:5000
AR-V7	Precision	AG10008	WB	1:1000
Normal IgG	Cell Signaling	#2729	IP, ChIP	N.A.
Histone H3	Cell Signaling	#4620	ChIP	N.A.
α-tublin	Cell Signaling	#2125	WB	1:1000
AR	Santa Cruz	N-20X,sc-816X	ChIP	N.A.
AR	Cell Signaling	D6F11,#5153	IHC	N.A.
OPRK1	Abcam	ab113533	IHC	N.A.
